# Identification of molecular subtypes of coronary artery disease based on ferroptosis- and necroptosis-related genes

**DOI:** 10.3389/fgene.2022.870222

**Published:** 2022-09-20

**Authors:** Wen-Pan Liu, Peng Li, Xu Zhan, Lai-Hao Qu, Tao Xiong, Fang-Xia Hou, Jun-Kui Wang, Na Wei, Fu-Qiang Liu

**Affiliations:** ^1^ Cardiovascular Department, Shaanxi Provincial People’s Hospital, Xi’an, Shaanxi, China; ^2^ Department of Cardiothoracic Surgery, The First People’s Hospital of Kunming City and Ganmei Affiliated Hospital of Kunming Medical University, Kunming, Yunnan, China; ^3^ Department of Surgery, Nanzhao County People’s Hospital, Nanyang, Henan, China; ^4^ Department of Cardiothoracic Surgery, The First Affiliated Hospital of Chongqing Medical University, Chongqing, China; ^5^ Department of Cardiothoracic Surgery, Yan’an Affiliated Hospital of Kunming Medical University, Kunming, Yunnan, China

**Keywords:** coronary artery disease, ferroptosis, necroptosis, subgroup, single-sample gene set enrichment analysis

## Abstract

**Aim:** Coronary artery disease (CAD) is a heterogeneous disorder with high morbidity, mortality, and healthcare costs, representing a major burden on public health. Here, we aimed to improve our understanding of the genetic drivers of ferroptosis and necroptosis and the clustering of gene expression in CAD in order to develop novel personalized therapies to slow disease progression.

**Methods:** CAD datasets were obtained from the Gene Expression Omnibus. The identification of ferroptosis- and necroptosis-related differentially expressed genes (DEGs) and the consensus clustering method including the classification algorithm used km and distance used spearman were performed to differentiate individuals with CAD into two clusters (cluster A and cluster B) based expression matrix of DEGs. Next, we identified four subgroup-specific genes of significant difference between cluster A and B and again divided individuals with CAD into gene cluster A and gene cluster B with same methods. Additionally, we compared differences in clinical information between the subtypes separately. Finally, principal component analysis algorithms were constructed to calculate the cluster-specific gene score for each sample for quantification of the two clusters.

**Results:** In total, 25 ferroptosis- and necroptosis-related DEGs were screened. The genes in cluster A were mostly related to the neutrophil pathway, whereas those in cluster B were mostly related to the B-cell receptor signaling pathway. Moreover, the subgroup-specific gene scores and CAD indices were higher in cluster A and gene cluster A than in cluster B and gene cluster B. We also identified and validated two genes showing upregulation between clusters A and B in a validation dataset.

**Conclusion:** High expression of *CBS* and *TLR4* was related to more severe disease in patients with CAD, whereas *LONP1* and *HSPB1* expression was associated with delayed CAD progression. The identification of genetic subgroups of patients with CAD may improve clinician knowledge of disease pathogenesis and facilitate the development of methods for disease diagnosis, classification, and prognosis.

## Introduction

Coronary artery disease (CAD) is a common cardiac disease and the primary cause of cardiovascular disease-related death (Agha et al., 2019; McCaffrey et al., 2021). CAD is caused by atherosclerosis or atherosclerotic occlusions of the coronary arteries (Thomas et al., 2018). Numerous environmental and genetic variables, including age, smoking habit, hypertension, dyslipidemia, obesity, diabetes, and family history, contribute to CAD (Chiu et al., 2018; Madhavan et al., 2018; Zhang H. W. et al., 2020). According to the CAD prediction model, approximately 20 million deaths and 16 million cases of reduced worker productivity were attributable to CAD in China between 2010 and 2015. Similar numbers are expected between 2000 and 2029 (Moran et al., 2008). The prevalence of CAD continues to increase, and clinical outcomes remain unsatisfactory. Although several studies have reported remarkable progress in the identification of diagnostic biomarkers for CAD in the blood (including long noncoding RNAs, methylation, and mRNAs) (Wang et al., 2020; Zhang X. et al., 2020; Chen Z. et al., 2021; Yang and Xu, 2021; Zhang L. et al., 2021) only two studies have described clinical heterogeneity in patients with different CAD severities (Zhang X. et al., 2020; Zhang B. et al., 2021). Therefore, scientific classification and targeted treatment may facilitate effective management of CAD. Current categorization approaches are based on pathological characteristics, disease development, and clinical symptoms, and the use of genotypic subgroups is developing slowly (Davies et al., 2021). The development of gene chip makes it possible to study the occurrence and development of CAD at the gene level (Franchini, 2016; Musunuru and Kathiresan, 2019).

At present, a review has reported forms of cell death that could affect CAD risk, such as apoptosis, pyroptosis, parthanatos, and autophagy, which have been previously implicated in CAD pathogenesis (Del Re et al., 2019). Additional, both ferroptosis and necroptosis are also involved in the development of CAD (Del Re et al., 2019). At present, Zhou et al.( 2021) has reported the specific mechanism of ferroptosis combined with pyroptosis in coronary atherosclerosis, and screened important marker genes. However, there is no study on ferroptosis combined with necroptosis in CAD. Ferroptosis, a novel type of cell death discovered in the last few years, is accompanied by accumulation of large amounts of iron and lipid peroxidation during the cell death process (Li et al., 2020). Furthermore, ferroptosis is an independent process triggered by the presence of harmful lipid reactive oxygen species and the consumption of polyunsaturated fatty acids (Li et al., 2020); these features distinguish ferroptosis from apoptosis, necrosis, and autophagy. Bai et al. (2020) fed apolipoprotein E (ApoE)^−/−^ mice a high-fat diet (HFD) to induce atherosclerosis in the presence or absence of the widely utilized ferroptosis inhibitor Ferrostatin-1 (Fer-1) and showed that Fer-1 treatment dramatically reduced atherosclerotic lesions, suggesting that ferroptosis occurs in mice with HFD-induced atherosclerosis. Further *in vitro* investigations using mouse aortic endothelial cells indicated that endothelial dysfunction significantly contributes to ferroptosis in the setting of atherosclerosis. Necroptosis is another newly defined form of cell death similar to necrosis and apoptosis in terms of morphology (e.g., cell swelling and rupture) (Vandenabeele et al., 2010). The necrotic features of necroptosis are marked by cell rupture and the release of immunogenic intracellular components, which activate inflammatory responses, highlighting the pro-inflammatory features of necroptosis. Vandenabeele et al. (2010) found that lipid peroxidation enhances oxidized low-density lipoprotein (ox-LDL) buildup and necroptosis. Moreover, the powerful pro-inflammatory effects of necroptosis can cause atherosclerosis. Therefore, necroptosis and ferroptosis inhibitors are promising strategies for targeting atherosclerosis, oxidative stress, and inflammatory responses. Initially, apoptosis, autophagy, and necrosis were thought to be mutually exclusive. However, recent studies have shown a delicate balance among these modes of death, suggesting that inhibition of one mode of death may increase the sensitivity of cells to activation of the other mode of death (Chen et al., 2018).

As gene chip analysis becomes more affordable, more researchers are using this approach to uncover the molecular pathways underlying the development and progression of CAD. However, most studies have only evaluated differences between CAD cases and normal controls, ignoring variances within CAD cases. Tumor samples are frequently subtyped in cancer research based on the expression patterns of ferroptosis- (Shan et al., 2021), pyroptosis- (Shao et al., 2021), DNA methylation- (Ding et al., 2019), and necroptosis-related genes (Zhao et al., 2021); such analyses can reveal intertumor heterogeneity, predict clinical endpoints, and guide treatment.

Therefore, in this study, we aimed to elucidate the mechanisms through which ferroptosis- and necroptosis-related genes contribute to CAD progression by typing CAD cases according to these genes. We also investigated the correlations between the typing results and clinical characteristics.

## Materials and methods

### Data collection and processing

mRNA expression profiles obtained from whole-blood samples of patients were downloaded from GEO (https://www.ncbi.nlm.nih.gov/geo/). GSE12288 (Sinnaeve et al., 2009) included 110 CAD samples and 112 normal samples based on the GPL96 platform, GSE20680 (Beineke et al., 2012) included 143 CAD samples and 52 normal samples based on the GPL4133 platform, and GSE20681 (Beineke et al., 2012) included 99 CAD samples and 99 normal samples based on the GPL4133 platform; these three datasets were used as training sets. GSE180083dataset by merging GSE180081 and GSE180082 datasets (McCaffrey et al., 2021) included 116 CAD samples and 60 normal samples based on the GPL14761 platform and was used as the validation dataset. Additionally, information regarding age, CAD index, and sex was collected. The Duke Coronary Artery Disease Index (CAD index) (Felker et al., 2002)was used to quantify the severity of lesions and diseased arteries and to assess the presence of lesions in the left anterior descending branch and main stem as an indicator of the severity of CAD.

The “normalizeBetweenArrays” function of limma package (Ritchie et al., 2015) was used to adjust the microarray data to quartiles for expression matrix of GSE12288, GSE20680 and GSE20681 datasets. Each gene was annotated using platform-provided annotation data. If a gene had several probes, the average expression level was determined. Batch effects were eliminated by combining GSE12288, GSE20680, and GSE20681 into a single dataset using the “sva” package in R software. A two-dimensional principal component analysis (PCA) cluster plot was used to illustrate intersample correction.

### Identification of ferroptosis- and necroptosis-related differentially expressed genes between normal and CAD samples

Ferroptosis- and necroptosis-related genes (*n* = 67 for necroptosis, *n* = 259 for ferroptosis) were retrieved from the molecular signature database (MSigDB4) and FerrDb5 (Zhou and Bao, 2020) (Supplementary Table S1). We performed differential analysis of ferroptosis- and necroptosis-related genes between normal and CAD samples using the wilcoxon test. Finally, we extracted 25 ferroptosis- and necroptosis-related DEGs (i.e., *NCF2*, *BNIP3*, *CBS*, *FTL*, *RPL8*, *HSPB1*, *MAP3K5*, *MAPK14*, *ELAVL1*, *HIC1*, *STAT3*, *PGD*, *SCP2*, *SLC38A1*, *MYB*, *TLR4*, *MTDH*, *LONP1*, *FADD*, *ITPK1*, *MYC*, *TNFSF10*, *DNMT1*, *BACH2*, and *LEF1*) from the dataset using difference analysis between normal and CAD samples.

### Construction of subgroups based on consensus clustering

We used consensus clustering (Wilkerson and Hayes, 2010) to categorize patients with CAD into distinct subgroups based on the expressed matrix of ferroptosis- and necroptosis-related DEGs between normal and CAD samples. Clustering was carried out using the Kmeans method and the Spearman distance. The maximum number of clusters was set at nine. The consensus matrix determined the final cluster number.

### Comparing the clinical characteristics of the two subgroups

Clinical data, such as age, sex, and CAD index, were obtained using the series matrix file downloaded from the three gene sets GSE12288, GSE20680, and GSE20681 in the GEO database. The continuous variables were age and CAD index, which were compared using the pairwise Wilcoxon rank sum test (Divine et al., 2013) and shown using box plots. Male patient proportions were examined as a categorical variable (ratios) and shown using histograms.

### Identification of DEGs between the two subgroups and gene ontology functional enrichment analysis

We utilized the R package “limma” to search for DEGs between the two subgroups. The screening criteria were as follows: |log (fold change [FC])| > 0.5 and adjusted *p* < 0.05. Next, we extracted 22 ferroptosis- and necroptosis-related DEGs using difference analysis between the two subgroups. The genes included *NCF2*, *BNIP3*, *CBS*, *FTL*, *RPL8*, *HSPB1*, *MAP3K5*, *MAPK14*, *STAT3*, *PGD*, *SCP2*, *SLC38A1*, *TLR4*, *MTDH*, *LONP1*, *FADD*, *ITPK1*, *MYC*, *TNFSF10*, *DNMT1*, *BACH2*, and *LEF1*. Using the “clusterProfiler” function in R, we investigated DEGs between cluster A and B enrichment in gene ontology (GO). GO functional enrichment analysis was used to determine the mechanisms in which the upregulated DEGs were involved in the two subgroups, and the findings were represented using an enrichment circle diagram. Results of enrichment analysis were considered statistically significant when *p* value was <0.05 and q value was <0.05.

### Estimation of immune cell infiltration

The abundance of immune cells in CAD samples was evaluated utilizing single sample gene set enrichment analysis (ssGSEA) and the “GSVA” R package. Gene set variation analysis (GSVA) is a non-parametric, unsupervised technique for calculating the variation of gene set enrichment across an expression dataset’s samples. Each ssGSEA enrichment score reflects the extent to which the genes in a specific gene set are coordinately up- or down-regulated in a sample. The essential criteria were as follows: abs.ranking = TRUE and kcdf = “Gaussian” and method = “ssgsea”. Based on the preceding analysis, we calculated the amount of immune cells in each sample (Xiao et al., 2020).

### Screening of subgroup‐specific genes and consensus clustering between subtypes

Subgroup‐specific genes (*LONP1*, *TLR4*, *CBS*, and *HSPB1*) were identified by intersection of the 22 ferroptosis- and necroptosis-related DEGs and DEGs between cluster A and B. Additionally, we typed all CAD samples (gene cluster A and B) again based on the four subgroup-specific gene expression matrix using the typing method described above. Finally, we compared the clinical characteristics of gene cluster A and B.

### Estimation of the subgroup‐specific gene signature

Then, using principal component analysis (PCA), a subgroup-specific gene signature was constructed. Components 1 and 2 were both chosen to act as signature scores. This method had the advantage of focusing the score on the set containing the largest block of highly correlated (or anticorrelated) genes, while down-weighting contributions from genes that do not track with other set members in the set. The subgroup-specific gene score was subsequently calculated using the following formula: subgroup-specific gene score = ∑(PC1i + PC2i) where PC1 denotes principal component 1 and PC2 denotes principal component 2, and i reflects the expression of subgroup-specific genes (Zhang B. et al., 2020).

### Validation of 4 subgroup‐specific genes in GSE180083

We used the dataset to validate the differences in 4 subgroup‐specific genes (*HSPB1, LONP1, TLR4* and *CBS*) in normal and CAD samples in the merged dataset and GSE180083 dataset. Results with *P* values less than 0.05 were considered statistically significant using two-sided tests.

## Results

**FIGURE 1:**
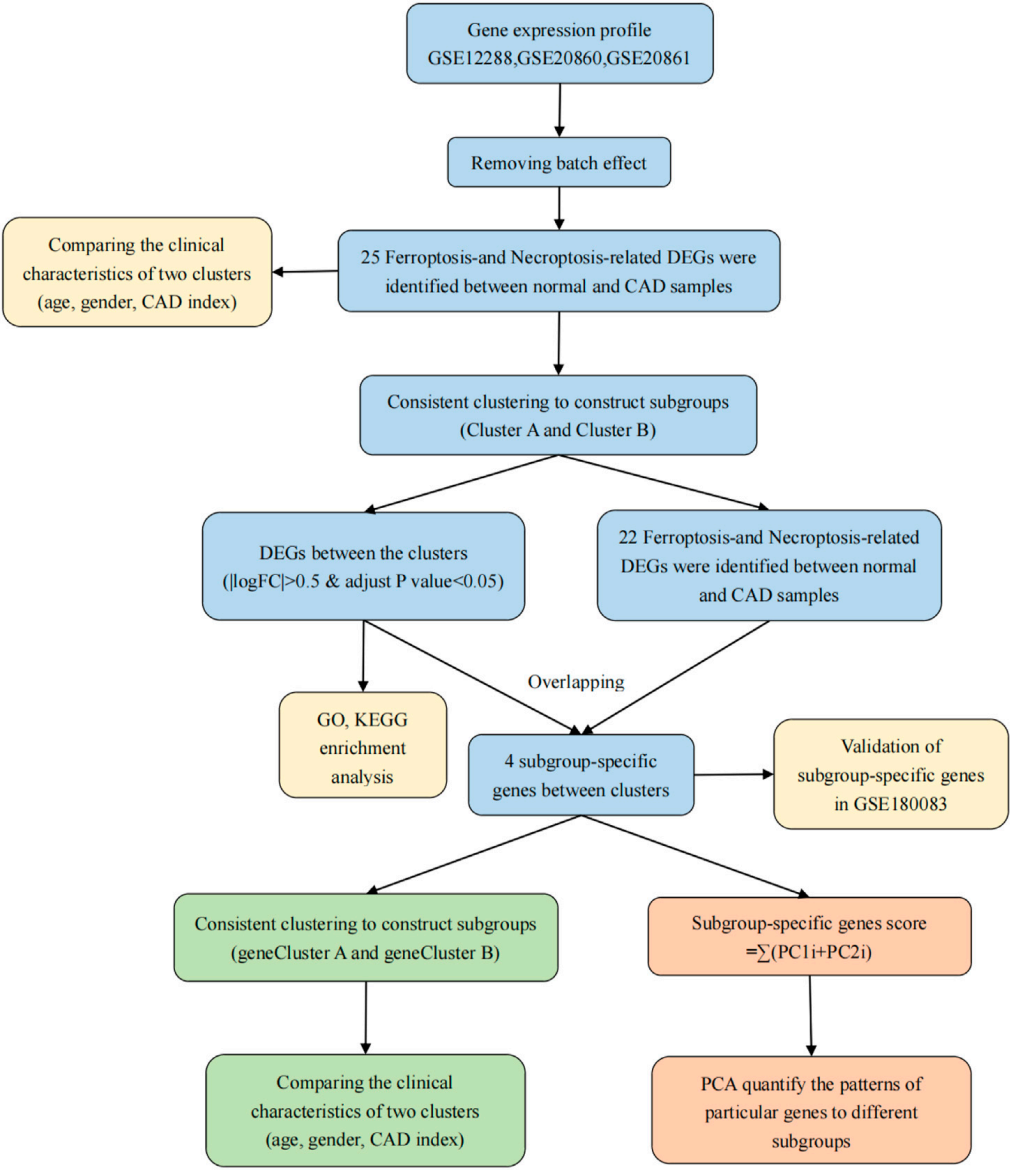
Flow chart of the research process.

Figure 1 depicts the procedures used in our cohort investigation. Supplementary Table S2 lists feature information for the training datasets (GSE12288, GSE20680, and GSE20681) and validation dataset (GSE180083).

### Elimination of batch effects

**FIGURE 2:**
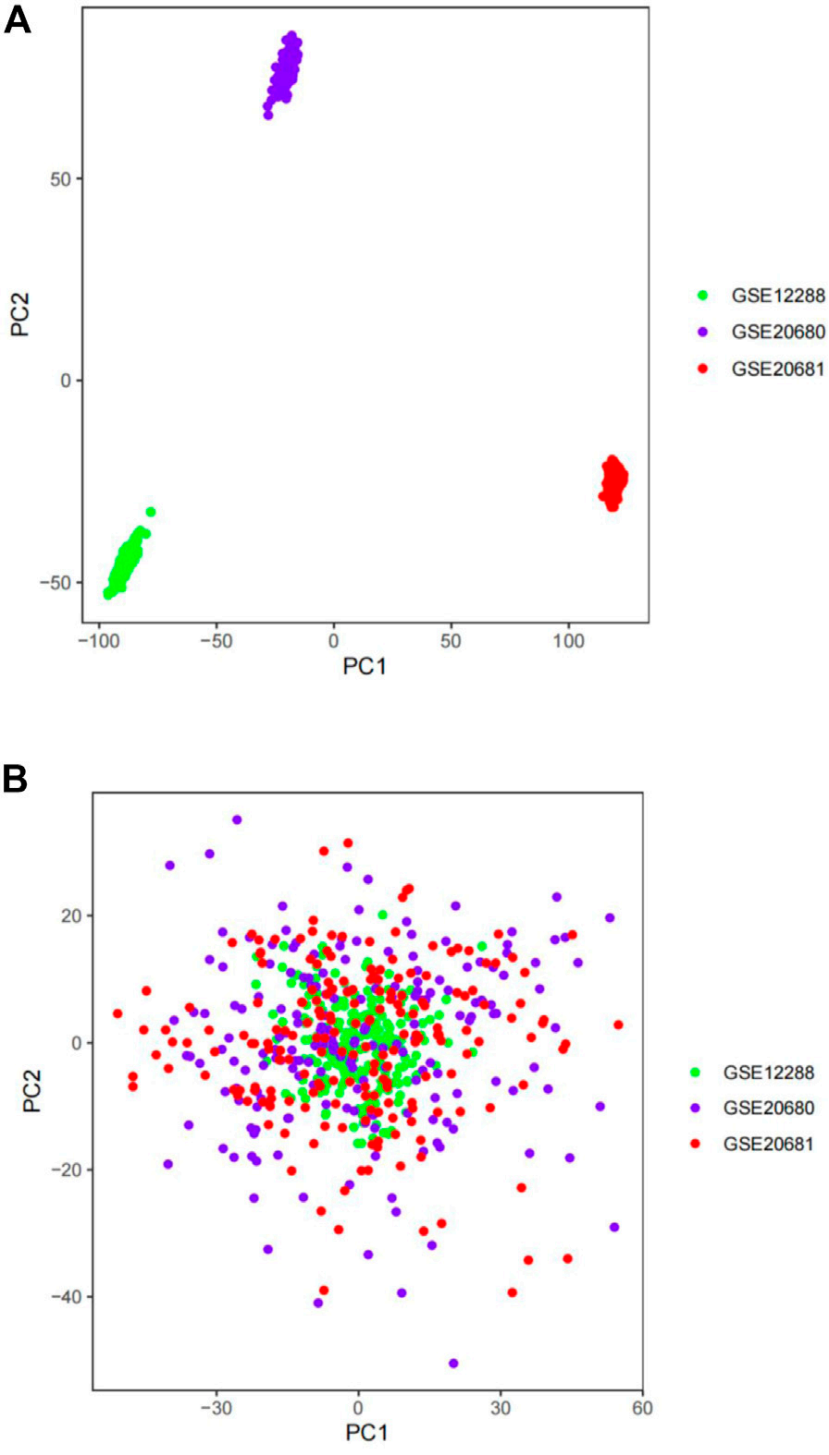
The gene expression datasets were processed using principal component analysis (PCA). The top two principal components (PC1 and PC2) of gene expression profiles were visualized as points on a scatter plot. Samples were based on data visualized without **(A)** and with **(B)** the batch effect removed. The colors indicate samples from three different datasets, which are represented by the dots.

First, we assessed the batch effects among GSE12288, GSE20680, and GSE20681 using a PCA cluster diagram. The results indicated that a batch effect did exist among the datasets (Figure 2A). Thus, using the R package “sva”, these three gene expression matrices were normalized and processed. After normalization and elimination of the batch effect, the data were depicted using a PCA cluster diagram (Figure 2B). The findings demonstrated unequivocally that the batch effect between GSE12288, GSE20680, and GSE20681 had been eliminated.

### Expression of 25 significant ferroptosis- and necroptosis-related DEGs in normal and CAD samples

**FIGURE 3:**
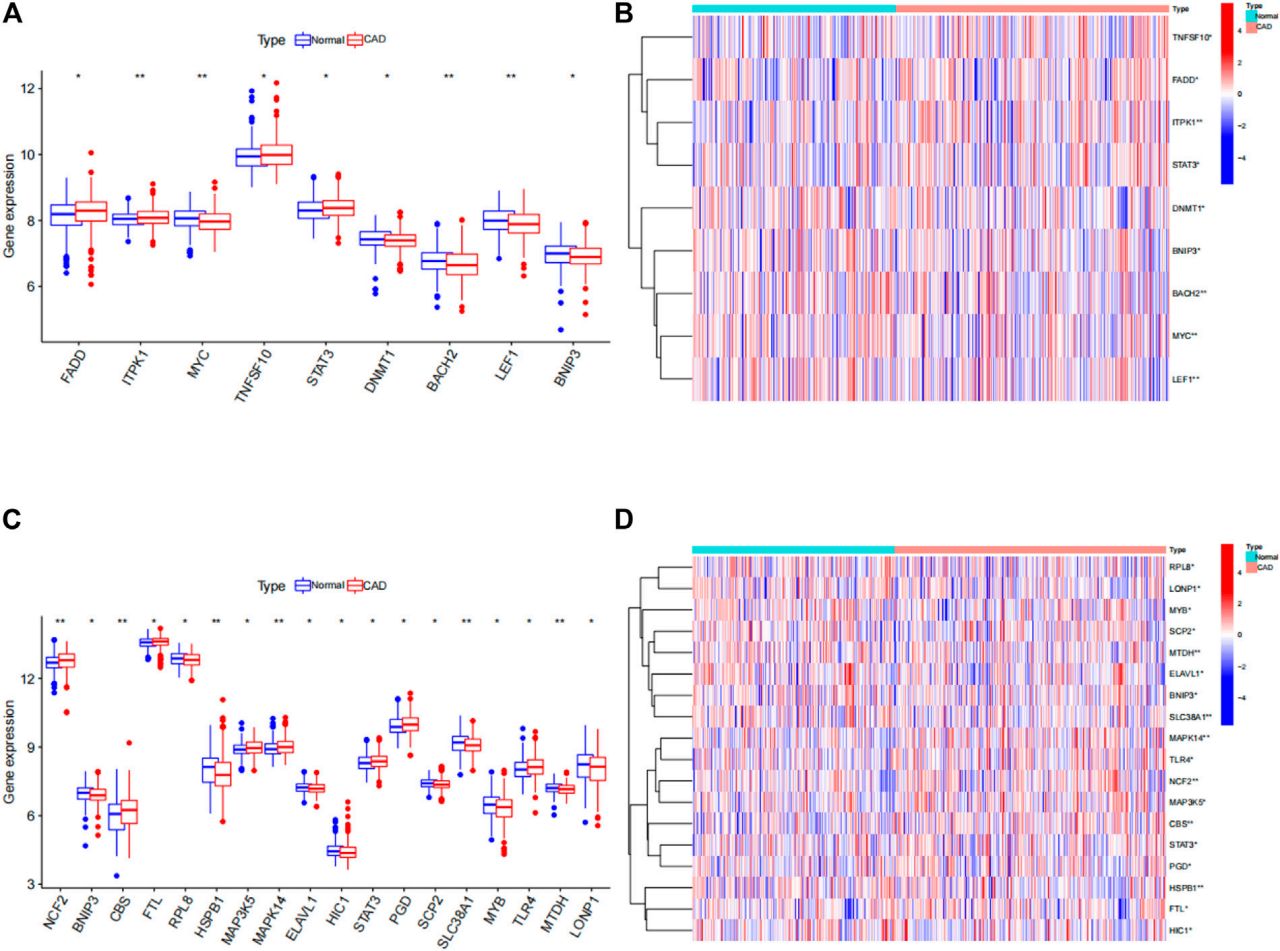
Differences in the expression of 25 ferroptosis- and necroptosis-related genes in control (con) and CAD (treat) groups. **(A)** Differential expression histogram of the nine necroptosis-related genes identified between controls and patients with CAD. **(B)** Expression heat map of the nine necroptosis-related genes in controls and patients with CAD. **(C)** Differential expression histogram of the 18 ferroptosis-related genes identified between controls and patients with CAD. **(D)** Expression heatmap of the 18 ferroptosis-related genes in controls and patients with CAD. *p < 0.05, **p < 0.01, and ***p < 0.001.

The “limma” package in R was utilized to analyze the differential expression levels of ferroptosis- and necroptosis-related genes in normal and CAD samples. Twenty-five significant ferroptosis- and necroptosis-related DEGs (*NCF2*, *BNIP3*, *CBS*, *FTL*, *RPL8*, *HSPB1*, *MAP3K5*, *MAPK14*, *ELAVL1*, *HIC1*, *STAT3*, *PGD*, *SCP2*, *SLC38A1*, *MYB*, *TLR4*, *MTDH*, *LONP1*, *FADD*, *ITPK1*, *MYC*, *TNFSF10*, *DNMT1*, *BACH2*, and *LEF1*) were identified between normal and CAD samples and visualized using a heat map and box plot (Figures 3A–D). We found that the necroptosis-related genes *MYC*, *DNMT1*, *BACH2*, *LEF1*, and *BNIP3* and the ferroptosis-related genes *BNIP3*, *RPL8*, *HSPB1*, *ELAVL1*, *HIC1*, *SCP2*, *SLC38A1*, *MYB*, *MTDH*, and *LONP1* were overexpressed in normal samples compared with CAD samples. By contrast, the necroptosis-related genes *FADD*, *ITPK1*, *TNFSF10*, *STAT3*, and *DNMT1* and the ferroptosis-related genes *NCF2*, *CBS*, *FTL*, *MAP3K5*, *MAPK14*, *STAT3*, *PGD*, *SLC38A1*, *MYB*, *MTDH*, and *LONP1* displayed decreased expression in normal samples compared with those in CAD samples.

### Two subtypes were identified based on the expression of ferroptosis- and necroptosis-related DEGs

**FIGURE 4:**
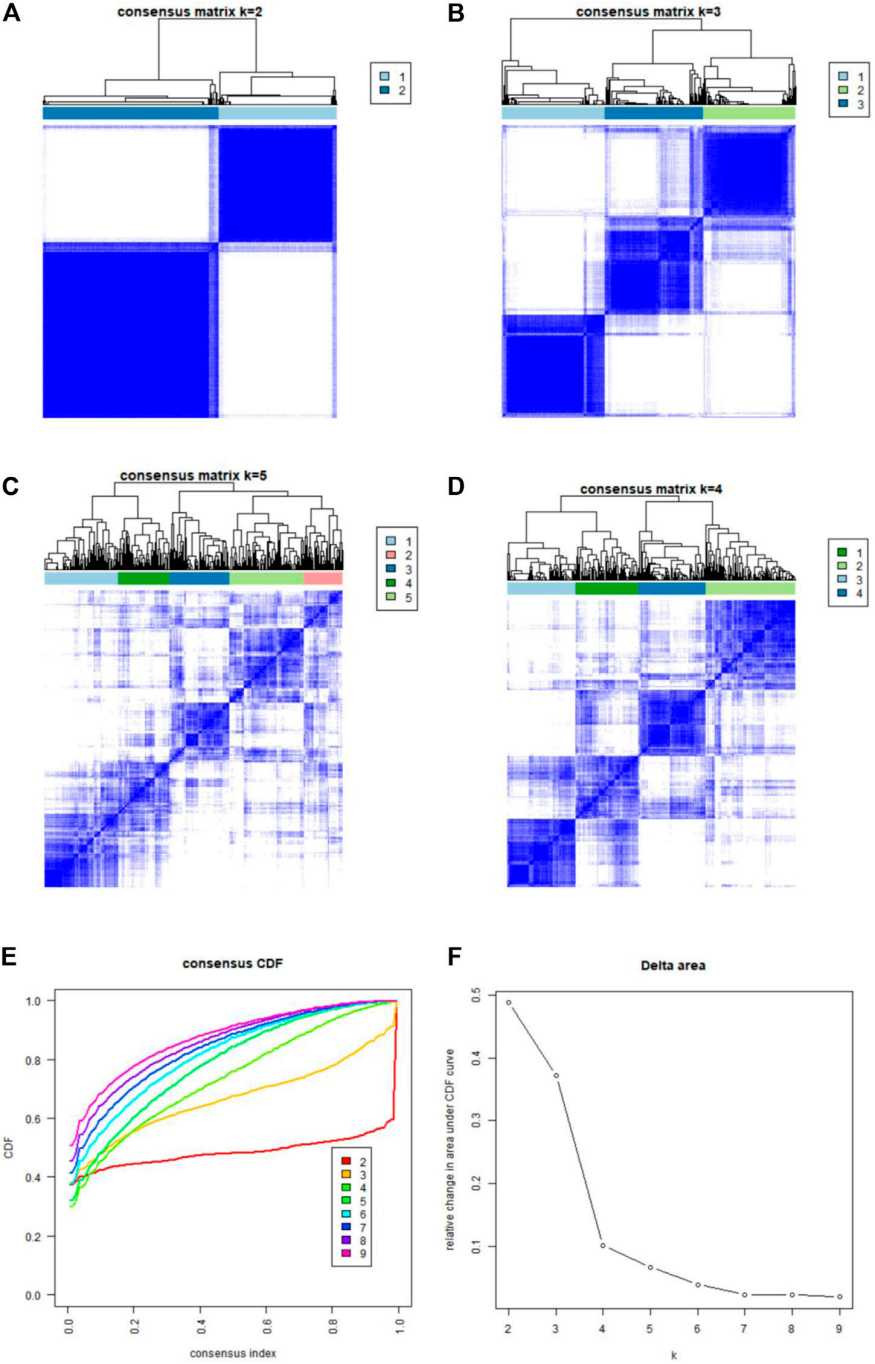
Consensus clustering of the 25 ferroptosis- and necroptosis-related genes in patients with CAD. **(A–D)** Consensus matrices of the 25 ferroptosis- and necroptosis-related genes for k = 2–5. **(E)** CDF curve for k = 2–9. **(F)** Delta area scores of the CDF curve for k = 2–9.

**FIGURE 5:**
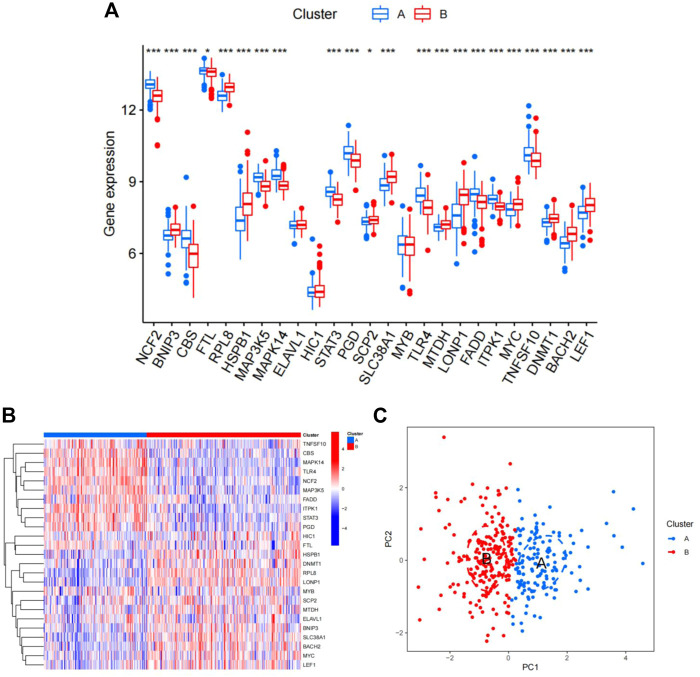
Differences in the expression of 25 ferroptosis- and necroptosis-related genes in cluster A and cluster B. **(A)** Differential expression histogram of the 25 ferroptosis- and necroptosis-related genes in cluster A and cluster B. Red: cluster B; blue: cluster A. **(B)** Expression heat map of the 25 ferroptosis- and necroptosis-related genes in cluster A and cluster B. Red: cluster B; blue: cluster A; red: high expression; blue: low expression. **(C)** Principal component analysis of the expression profiles of the 25 ferroptosis- and necroptosis-related genes, demonstrating marked differences in transcriptomes between cluster A and cluster B. Red: cluster B; blue: cluster A. Each dot represents a sample.

**FIGURE 6:**
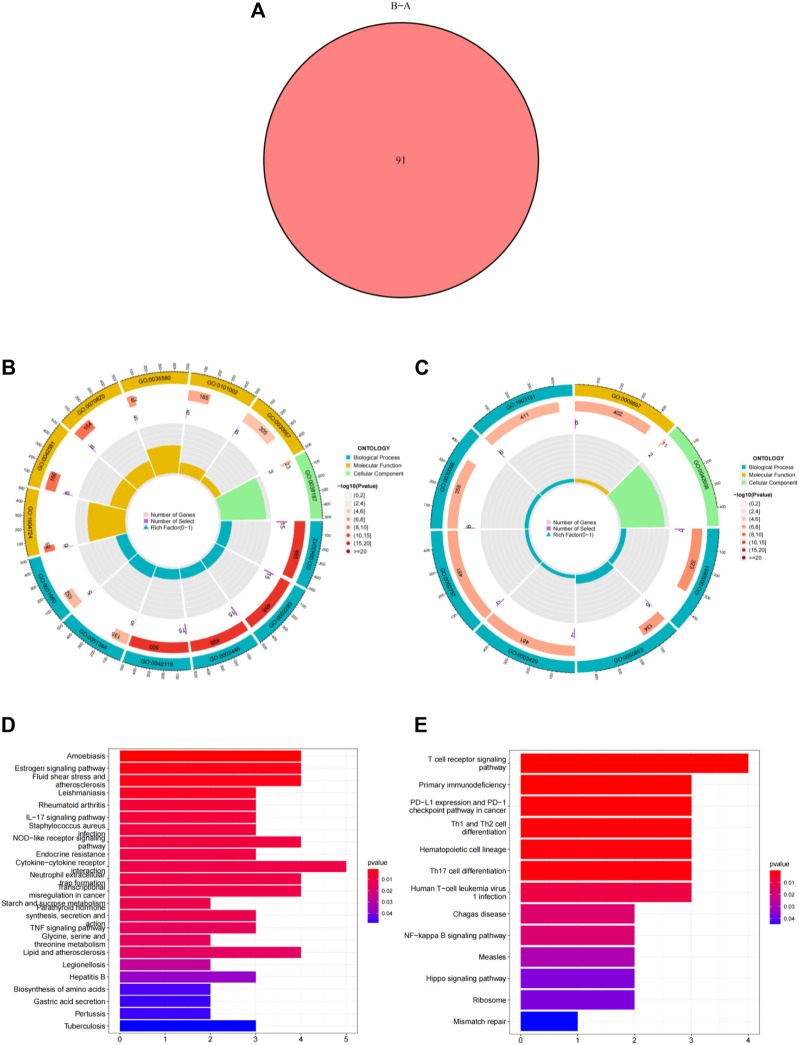
Differentially expressed genes (DEGs) and enrichment analysis between cluster A and cluster B. **(A)** Venn diagram representing the intersection of DEGs between cluster A and cluster B. **(B)** Gene ontology analysis of upregulated DEGs in cluster A. **(C)** Gene ontology analysis of upregulated DEGs in cluster B. **(D)** Kyoto Encyclopedia of Genes and Genomes analysis of the upregulated DEGs in cluster A. **(E)** Kyoto Encyclopedia of Genes and Genomes analysis of upregulated DEGs in cluster B.

Using the “ConsensusClusterPlus” package in R software, we performed consensus clustering to identify two subgroups (cluster A and cluster B) based on the expression of the 25 significant ferroptosis- and necroptosis-related DEGs in all CAD samples (*n* = 352) (Figures 4A–F, Supplementary Table S3). Cluster A included 141 cases, whereas cluster B included 211 cases. The heat map and box plot were then used to depict the differential expression levels of the 25 significant ferroptosis- and necroptosis-related DEGs between the cluster A and B (Figures 5A,B). *NCF2*, *CBS*, *FTL*, *MAP3K5*, *MAPK14*, *STAT3*, *PGD*, *TLR4*, *FADD*, *ITPK1*, and *TNFSF10* were all expressed at higher levels in cluster A than in cluster B, whereas *RPL8*, *HSPB1*, *ELAVL1*, *HIC1*, *SCP2*, *SLC38A1*, *MTDH*, *LONP1*, *MYC*, *DNMT1*, *BACH2*, and *LEF1* showed the opposite trends. There were no significant differences in *ELAVL1*, *HIC1*, or *MYB* expression between cluster A and cluster B. According to PCA, the expression of the 25 significant ferroptosis- and necroptosis-related DEGs could fully separate the cluster A and B (Figure 5C). Between the cluster A and B, 91 DEGs were identified (Figure 6A; Table 1). To elucidate the potential mechanisms through which 91 DEGs contributed to CAD, we used GO functional enrichment analysis and illustrated the findings using an enrichment circle diagram (Figures 6B–E, Supplementary Table S4). We found that the majority of upregulated genes in cluster A were enriched in GO:0043312 (neutrophil degranulation), GO:0002283 (neutrophil activation involved in immune response), GO:0002446 (neutrophil mediated immunity), and GO:0042119 (neutrophil activation), whereas the majority of upregulated genes in cluster B were enriched in GO:0050851 (antigen receptor-mediated signaling pathway), GO:0050853 (B cell receptor signaling pathway), GO:0002429 (immune response-activating cell surface receptor signaling pathway), and GO:0002757 (immune response-activating signal transduction). All of these categories were associated with inflammation.

**TABLE 1:** Differential genes between cluster A and cluster B.

Genes	logFC	P.Value	adj.P.Val
Up-regulated in cluster B			
LCK	0.504546053	1.51E-30	1.56E-27
RPL36	0.521792929	3.14E-30	2.96E-27
LONP1	0.803850527	6.69E-29	3.60E-26
FAIM3	0.509238851	7.80E-27	1.96E-24
KRI1	0.741118014	2.72E-26	5.81E-24
TCF7	0.510489225	2.33E-25	4.18E-23
CD3D	0.507503667	7.36E-25	1.17E-22
CD79B	0.580450466	7.14E-22	6.16E-20
CD52	0.547967917	8.20E-20	4.59E-18
CD3G	0.770664616	2.36E-19	1.26E-17
LIG1	0.503740429	4.83E-17	1.86E-15
PRMT1	0.519897888	5.16E-17	1.95E-15
COQ4	0.554835126	1.74E-16	6.03E-15
ICOS	0.614182382	2.87E-15	7.79E-14
LTBP3	0.569074238	3.58E-15	9.57E-14
NME3	0.577144816	4.22E-15	1.12E-13
BIRC3	0.589328467	4.24E-15	1.12E-13
RPL34	0.564808402	1.17E-14	2.92E-13
HSPB1	0.633625542	1.83E-14	4.41E-13
FBXL15	0.540083039	4.24E-14	9.49E-13
IGHM	0.606806869	4.59E-14	1.02E-12
LSR	0.555946486	6.37E-14	1.38E-12
MS4A1	0.503015635	6.56E-14	1.41E-12
IGHD	0.508656664	9.66E-14	2.04E-12
MRPS33	0.527669416	3.39E-13	6.48E-12
C12orf29	0.573221133	1.48E-12	2.54E-11
EPHX2	0.540691194	2.38E-12	3.89E-11
WDR18	0.503291017	4.48E-12	6.92E-11
LRIG1	0.521258506	1.08E-11	1.56E-10
TCL1A	0.595110216	5.42E-10	5.79E-09
XIST	0.815900198	0.002715629	0.008356416
Up-regulated in cluster A			
NFIL3	−0.579779291	7.80E-37	7.27E-33
FAM129A	−0.581504267	3.59E-32	5.07E-29
REPS2	−0.55999927	5.16E-32	6.48E-29
TNFRSF10C	−0.557569777	1.11E-30	1.26E-27
QPCT	−0.602786665	9.52E-30	7.69E-27
MGAM	−0.753175116	2.11E-29	1.32E-26
DUSP1	−0.640946149	7.76E-29	3.99E-26
TLR4	−0.550786326	1.59E-28	6.41E-26
IL1R2	−0.66514049	1.85E-28	7.21E-26
KCNJ15	−0.624123879	2.26E-28	8.25E-26
MEGF9	−0.506620185	7.62E-28	2.33E-25
RALB	−0.52961537	8.23E-28	2.45E-25
PYGL	−0.516041358	1.25E-27	3.63E-25
ABHD5	−0.510208938	1.30E-26	3.14E-24
ACSL1	−0.644907146	4.05E-26	8.18E-24
AQP9	−0.524605253	8.22E-26	1.58E-23
CLEC4E	−0.749241373	3.08E-24	4.65E-22
FCGR3B	−0.591635756	6.11E-24	8.38E-22
IGF2R	−0.588144324	6.14E-24	8.38E-22
NAMPT	−0.532538173	2.58E-23	3.09E-21
CYP4F3	−0.621164391	6.31E-23	6.61E-21
BCL6	−0.512419287	1.07E-22	1.06E-20
MANSC1	−0.534944844	1.49E-22	1.42E-20
ADM	−0.604059281	1.17E-21	9.63E-20
CBS	−0.718822658	1.47E-21	1.16E-19
KCNJ2	−0.563929088	2.34E-21	1.81E-19
BASP1	−0.502355608	1.10E-20	7.20E-19
FPR2	−0.515456987	3.25E-20	1.93E-18
MMP9	−0.850613784	3.77E-20	2.21E-18
MMP25	−0.510194692	4.06E-20	2.36E-18
BMX	−0.833485855	1.21E-19	6.70E-18
MME	−0.566122322	1.49E-19	8.13E-18
DYSF	−0.508463926	3.42E-19	1.75E-17
ARG1	−0.83609682	3.88E-18	1.78E-16
ANXA3	−0.586668109	3.97E-18	1.81E-16
CXCL1	−0.509163147	4.25E-18	1.92E-16
RNASEL	−0.538518792	6.37E-17	2.37E-15
NSUN7	−0.61003545	8.54E-17	3.12E-15
LIN7A	−0.513847273	9.56E-17	3.45E-15
KRT23	−0.588993838	3.86E-16	1.24E-14
FOS	−0.572751329	1.75E-15	4.93E-14
G0S2	−0.595760463	3.65E-15	9.74E-14
ECHDC3	−0.510962133	3.72E-15	9.89E-14
HIST1H3G	−0.544657894	3.55E-14	8.07E-13
SP1	−0.509464563	5.08E-14	1.12E-12
UPF1	−0.562794433	5.25E-14	1.16E-12
GALNT14	−0.619462095	9.88E-14	2.08E-12
AOC3	−0.501629311	2.05E-13	4.07E-12
TREML2	−0.586877867	2.08E-13	4.11E-12
TNFAIP6	−0.56038306	3.66E-13	6.92E-12
TPST1	−0.617561054	5.07E-13	9.34E-12
OSM	−0.546563912	7.08E-13	1.28E-11
ZNF230	−0.539427697	1.95E-12	3.27E-11
MAK	−0.520492313	2.69E-12	4.34E-11
INHBB	−0.504318482	8.86E-12	1.29E-10
PGLYRP1	−0.602924119	1.37E-11	1.91E-10
HIST3H3	−0.502135761	2.55E-10	2.90E-09
HIST1H2BO	−0.508497062	1.89E-09	1.86E-08
APOBEC3B	−0.600843308	2.14E-09	2.07E-08
ORM1	−0.624701222	2.09E-08	1.69E-07

**FIGURE 7:**
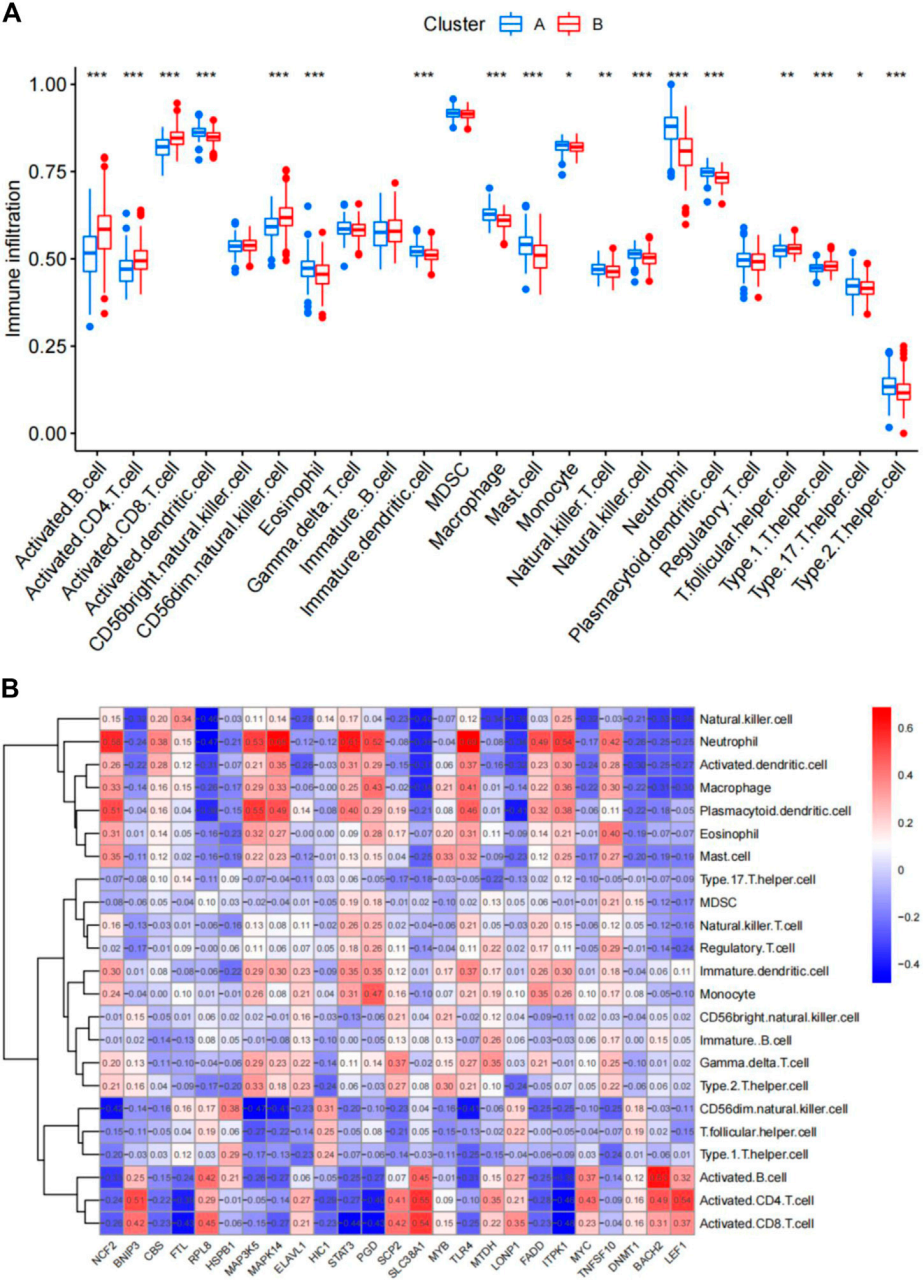
Single-sample gene set enrichment analysis. **(A)** Differential immune cell infiltration between cluster A and cluster B. **(B)** Correlation between infiltrating immune cells and the 25 ferroptosis- and necroptosis-related genes. *p < 0.05, **p < 0.01, and ***p < 0.001.

The abundance of immune cells in CAD samples was then calculated using ssGSEA (Supplementary Table S5), and the associations between the 25 significant ferroptosis- and necroptosis-related DEGs and immune cells were investigated by correlation test with spearman method. We found that *NCF2*, *CBS*, *FTL*, *MAP3K5*, *MAPK14*, *STAT3*, *PGD*, *TLR4*, *FADD*, *ITPK1*, and *TNFSF10* all exhibited positive relationships with various types of immune cells, particularly neutrophils (Figure 7B). We then investigated the differences in immune cell infiltration between patients with high and low expression of *NCF2*, *CBS*, *FTL*, *MAP3K5*, *MAPK14*, *STAT3*, *PGD*, *TLR4*, *FADD*, *ITPK1*, and *TNFSF10*. The findings indicate that patients with elevated levels of *NCF2*, *CBS*, *FTL*, *MAP3K5*, *MAPK14*, *STAT3*, *PGD*, *TLR4*, *FADD*, *ITPK1*, and *TNFSF10* showed greater infiltration of immune cells than those with low levels (Supplementary Figure S1A–K). Finally, we analyzed the differential infiltration of immune cells between the two subgroups (Figure 7A). Cluster A was found to be closely connected with several types of inflammatory cells, suggesting that the infiltration of various immune cells plays a very important role in cluster A.

### Comparison of the clinical characteristics of the two subgroups

**FIGURE 8:**
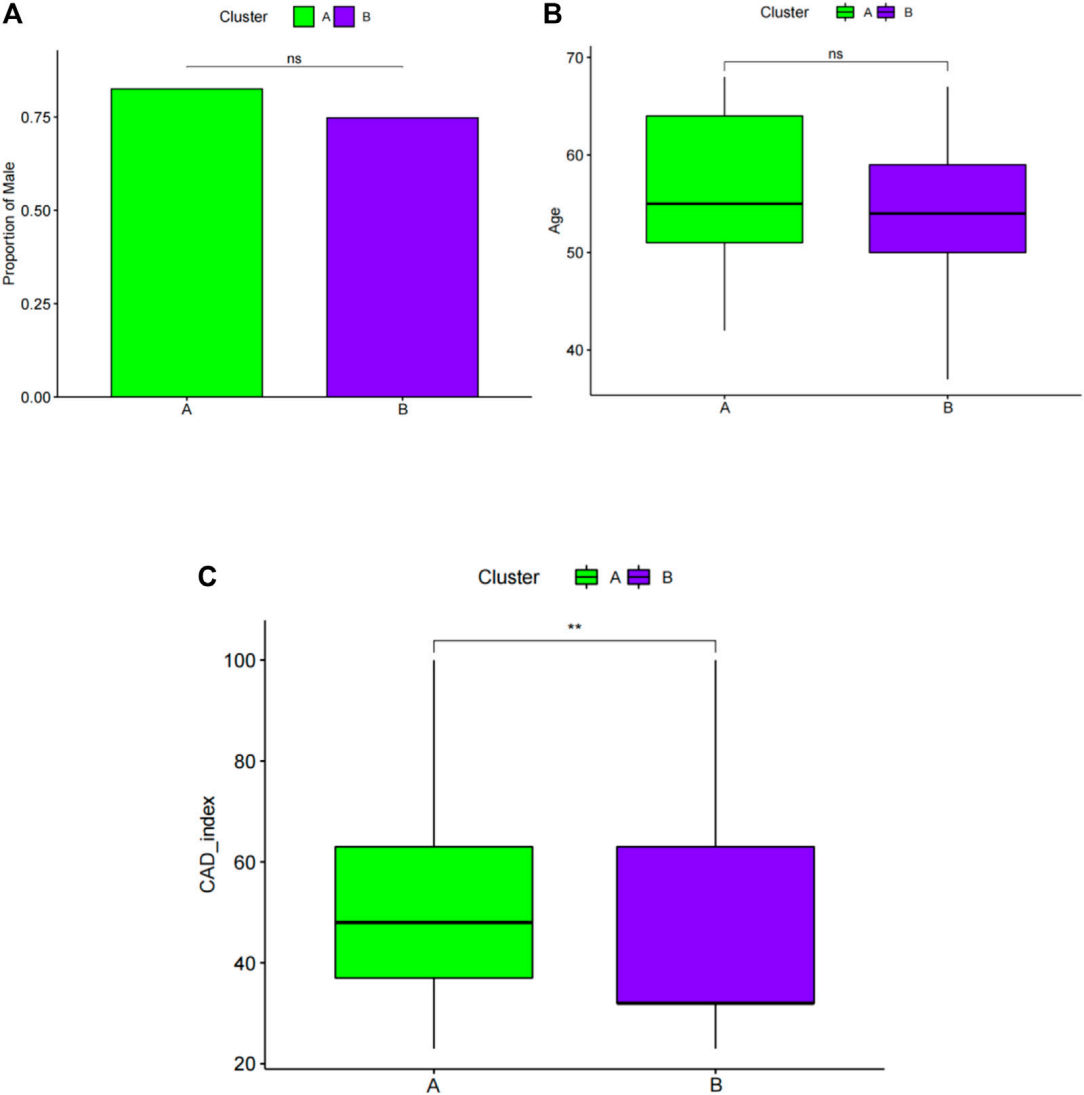
Pairwise comparisons of clinical characteristics between cluster A and cluster B. **(A)** The proportion of men in each subgroup is represented by the bar plot. Boxplots **(B,C)** display ages and CAD indices for subgroups. CAD: coronary artery disease. *p < 0.05, **p < 0.01, and ***p < 0.001.

Next, sex, age, and CAD index were gained in patients with CAD from the GSE12288 dataset and only sex were gained in patients with CAD from the GSE20681 dataset to identify the clinical features of the two subgroups. There are no useful clinical features in GSE20680 dataset. Specifically, there were no significant variations in the proportion of men or in age between clusters A and B (Figures 8A,B). Additionally, cluster A showed a higher CAD index than cluster B, indicating that individuals in cluster A may have greater disease severity (Figure 8C). We then conducted analysis of variance (ANOVA) to compare ages in the two subgroups and found that subgroup served as an age-independent predictor of the extent of CAD (Table 2, *p* < 0.05). These findings suggested that the subgroups based on 25 ferroptosis- and necroptosis-related DEGs may reflect not only the phases of CAD development but also the basic biological aspects of CAD.

**TABLE 2:** The analysis of variance for the transcriptome classification, age, and their interaction.

		Df	Sum square	Mean square	F‐value	Pr (>F)	
Cluster A and cluster B	Transcriptome classification	1	1852	1851.9	4.618	0.0339	*
	Age	1	1318	1317.6	3.286	0.0727	
	Transcriptome classification and age interaction	1	392	391.9	0.977	0.3251	
	Residuals	106	42510	401			
GeneCluster A and geneCluster B	Transcriptome classification	1	4782	4782	12.493	0.000607	***
	Age	1	701	701	1.83	0.179005	
	Transcriptome classification and age interaction	1	11	11	0.028	0.868188	
	Residuals	106	40578	383			

Note. Df: degree of freedom.

Significant codes: 0 “***” 0.001 “**” 0.01 “*” 0.05 “.” 0.1 ‘’ 1.

### Identification of two subgroup‐specific gene patterns and generation of the subgroup‐specific gene signature

**FIGURE 9:**
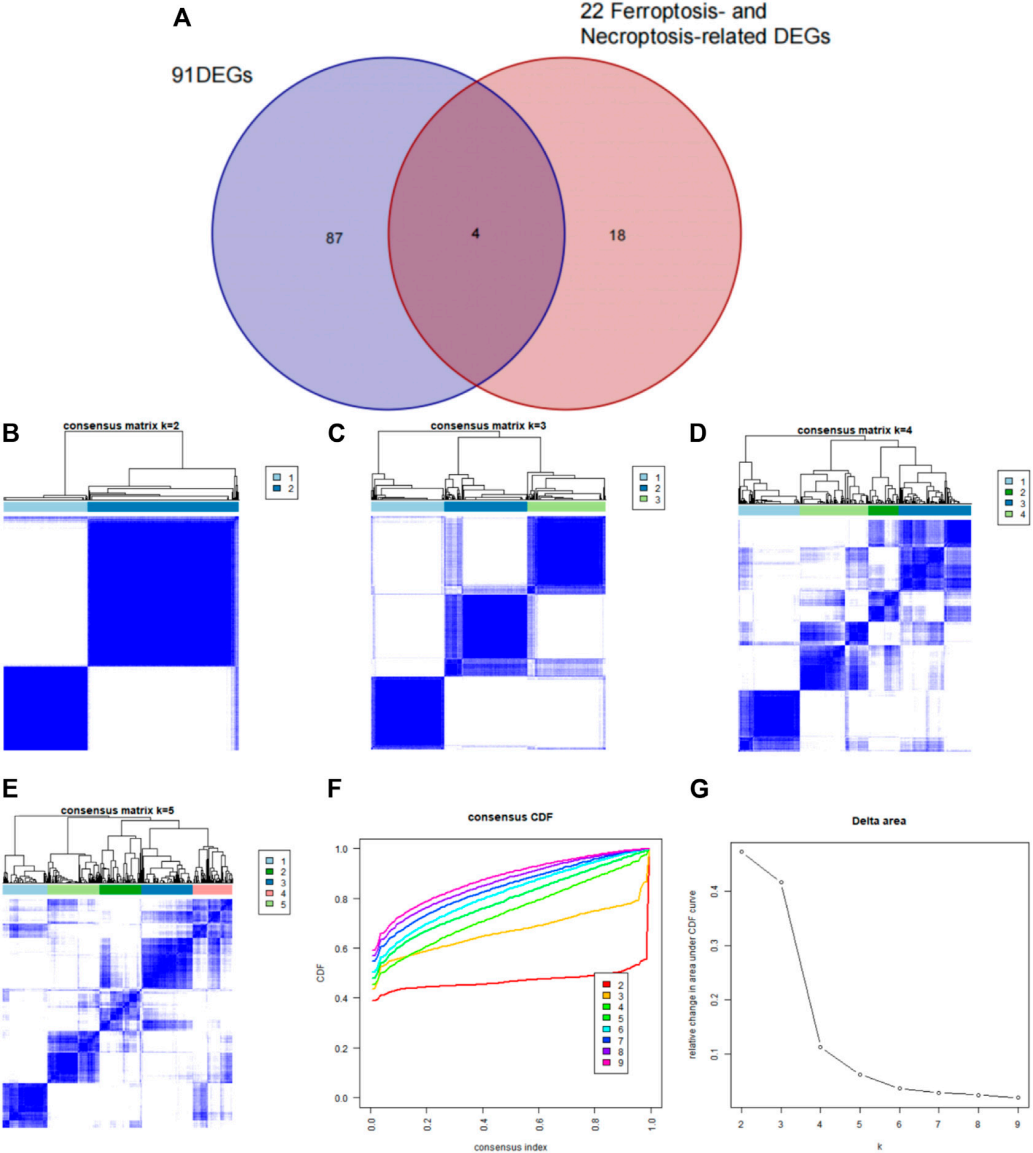
Identification of subgroup-specific genes in the two clusters and consensus clustering of the four subgroup-specific genes in patients with CAD. **(A)** The four subgroup‐specific genes were identified by intersection of 22 ferroptosis- and necroptosis-related DEGs and DEGs between the two clusters. **(B–E)** Consensus matrices of the four subgroup-specific genes for k = 2–5. **(F)** CDF curve for k = 2–9. **(G)** Delta area score of the CDF curve for k = 2–9.

**FIGURE 10:**
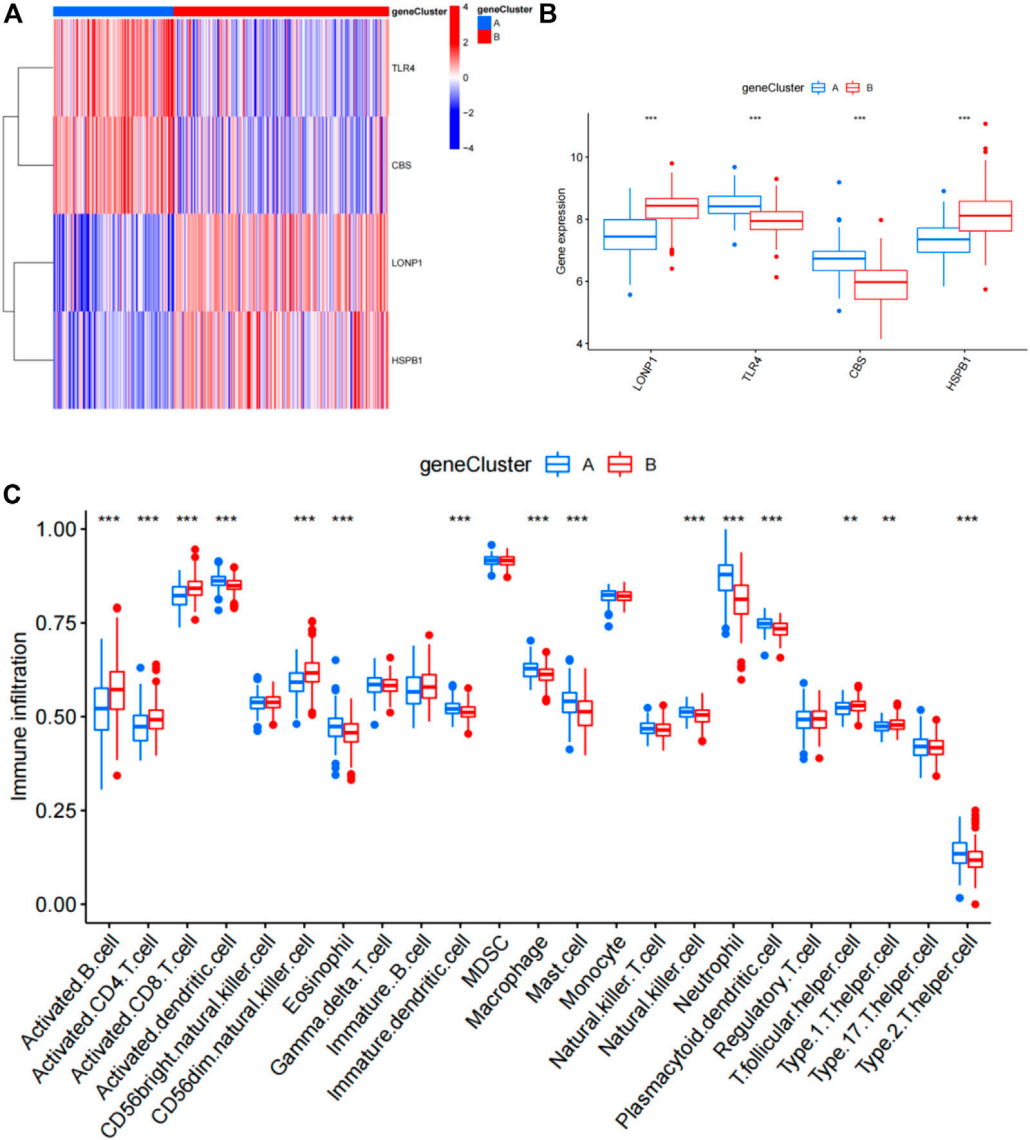
Differential analysis of four subgroup-specific genes and infiltrating immune cells between gene cluster A and gene cluster B. **(A)** Expression heat map of the four subgroup-specific genes in gene cluster A and gene cluster B. Red: gene cluster B; blue: gene cluster A; red: high expression; blue: low expression. **(B)** Differential expression histogram of the four subgroup-specific genes in gene cluster A and gene cluster B. Red: gene cluster B; blue: gene cluster A. **(C)** Differential immune cell infiltration between gene cluster A and gene cluster B. Red: gene cluster B; blue: gene cluster A.

**FIGURE 11:**
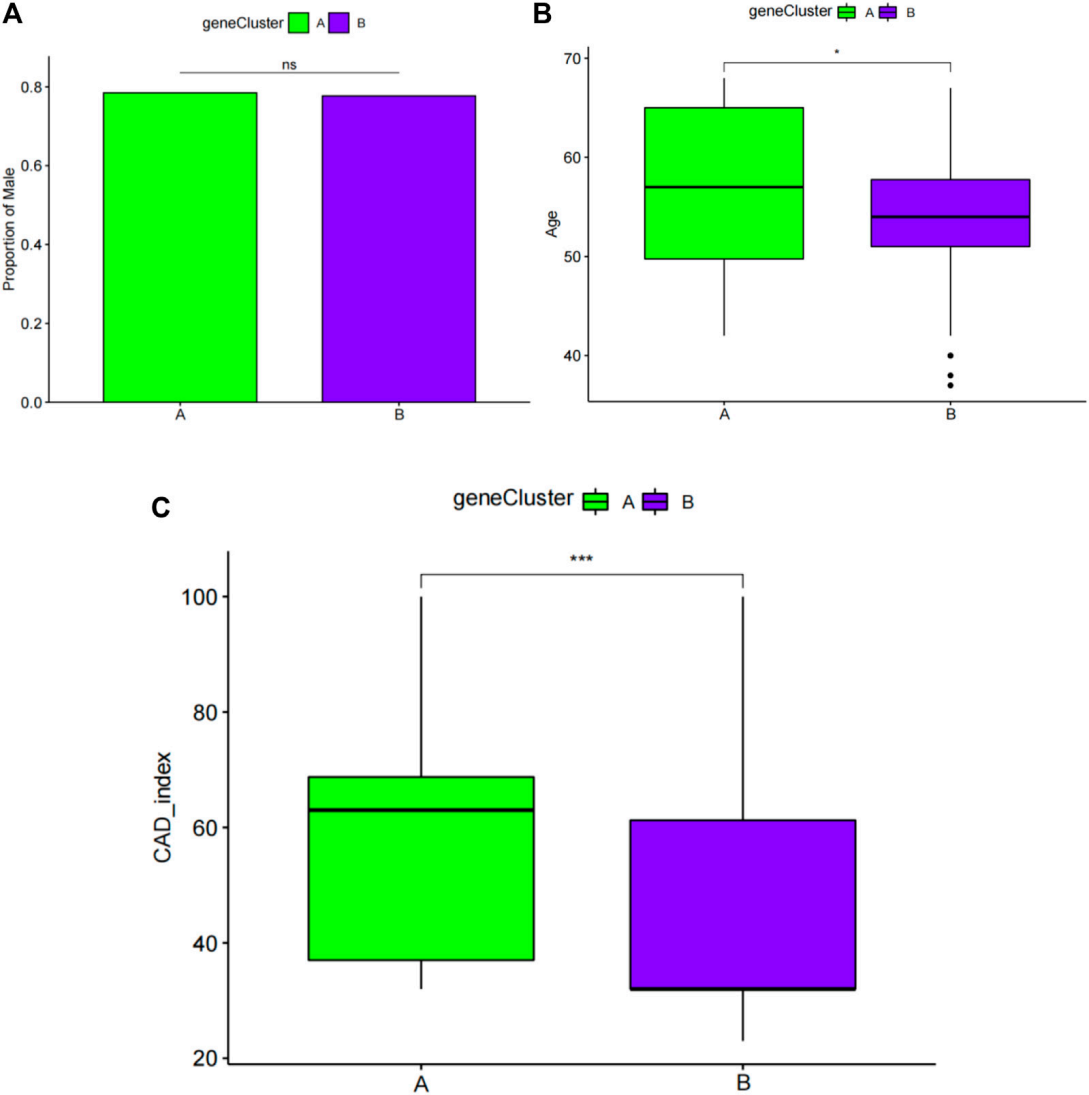
Pairwise comparisons of clinical characteristics between gene cluster A and gene cluster B. **(A)** The proportion of men in each subgroup is represented by the bar plot. Boxplots **(B,C)** display ages and CAD indices for the subgroups. CAD: coronary artery disease. *p < 0.05, **p < 0.01, and ***p < 0.001.

**FIGURE 12:**
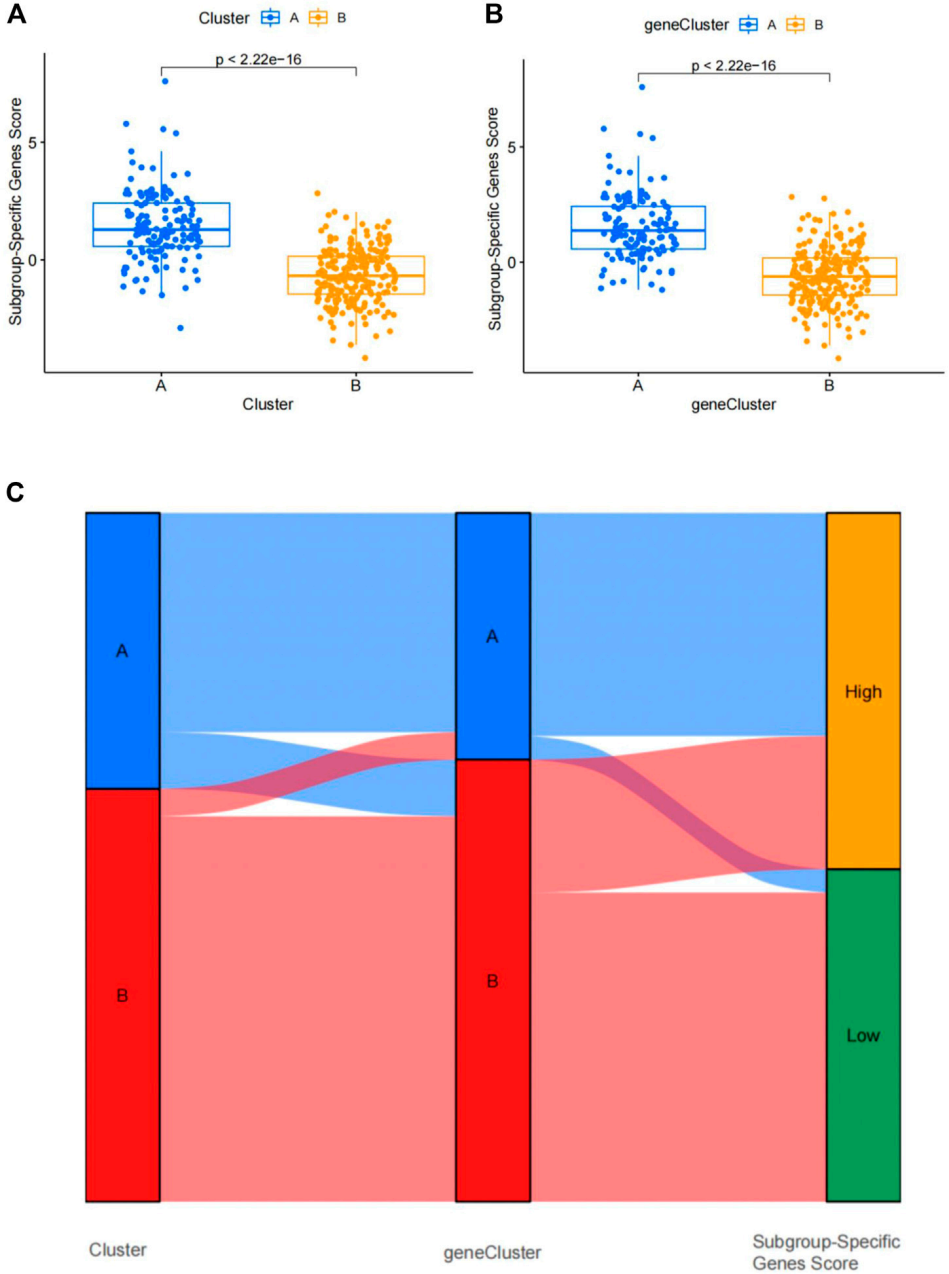
Differential analysis of four subgroup-specific gene scores between the two clusters. **(A)** Differences in four subgroup-specific gene scores between cluster A and cluster B. **(B)** Differences in four subgroup-specific gene scores between gene cluster A and gene cluster. *p* < 0.05 indicates significance. **(C)** Sankey diagram showing the relationships among clusters A and B, gene clusters A and B, and subgroup-specific scores.

To further confirm the four specific genes (Figure 9A) that determine typing, we performed consensus clustering to divide all CAD samples (n = 352) into gene cluster A and B based on the expression of the four subgroup-specific genes. We observed that the two subgroup-specific gene patterns (gene cluster A and gene cluster B; Supplementary Table S6) were consistent with clustering of ferroptosis- and necroptosis-related gene clusters (cluster A and B) (Figures 9B,C). According to our analysis, differences in subgroup-specific gene expression, immune cell infiltration (Figure 10C), and clinical features (Figures 11A–C) between gene cluster A and gene cluster B were likewise similar to above results in the cluster A and B. Additionally, individuals in gene cluster A were older and had higher CAD indices than individuals in gene cluster B (*p* < 0.001), indicating that disease may be more severe in individuals in gene cluster A. Additionally, we conducted ANOVA to compare ages and subgroups and found that subgroup was also an age-independent predictor (*p* < 0.05) (Table 2). This further confirmed the accuracy of our typing using the consensus clustering approach. We used PCA algorithms to calculate the subgroup-specific gene score for each sample in order to quantify the subgroup-specific gene clusters (Supplementary Table S7). The subgroup-specific gene score was then compared between the two separate ferroptosis- and necroptosis-related gene clusters or subgroup-specific gene clusters. The findings indicated that the subgroup-specific gene score was higher in cluster A or gene cluster A than in cluster B or gene cluster B (*p* < 0.05) (Figures 12A,B). Surprisingly, the subgroup-specific gene scores and CAD indices were greater in cluster A than in cluster B, indicating that these four genes may be critical in the progression of CAD. Additionally, all four of these genes were ferroptosis-related genes, indicating that ferroptosis played critical roles in controlling CAD progression. Interestingly, *CBS* and *TLR4* were overexpressed in individuals with CAD and were therefore considered risk genes, whereas *HSPB1* and *LONP1* were overexpressed in normal individuals and were thus considered protective genes. According to the findings of the second typing, *CBS* and *TLR4* were overexpressed in cluster A (*p* < 0.001), whereas *HSPB1* and *LONP1* were overexpressed in cluster B (*p* < 0.001). Thus, *CBS* and *TLR4* may accelerate CAD advancement, whereas *HSPB1* and *LONP1* may suppress CAD progression, underscoring the importance of categorization in determining the degree of CAD. A Sankey diagram was used to depict the association between ferroptosis- and necroptosis-related gene patterns, subgroup-specific gene patterns, and subgroup-specific gene scores (Figure 12C).

### Validation of 4 subgroup‐specific genes in the merged dataset and GSE180083 dataset

**FIGURE 13:**
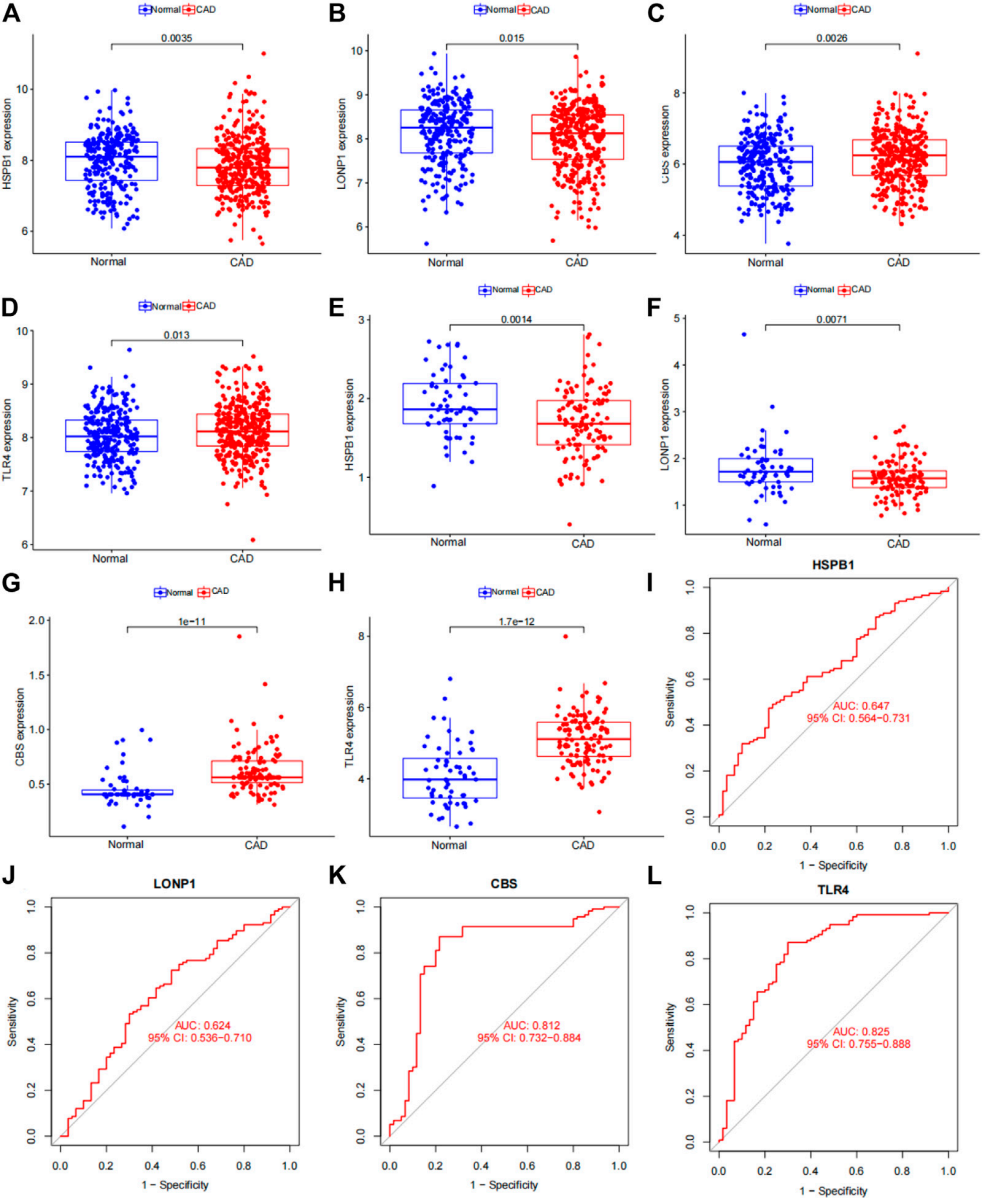
Verification of 4 subgroup-specific genes. **(A)** *HSPB1* expression in patients with CAD samples compared with normal samples in the merged dataset. **(B)** *LONP1* expression in patients with CAD samples compared with normal samples in the merged dataset. **(C)** *CBS* expression in patients with CAD samples compared with normal samples in the merged dataset. **(D)** *TLR4* expression in patients with CAD samples compared with normal samples in the merged dataset. **(E)** *HSPB1* expression in patients with CAD samples compared with normal samples in the GSE180083 dataset. **(F)** *LONP1* expression in patients with CAD samples compared with normal samples in the GSE180083 dataset. **(G)** *CBS* expression in patients with CAD samples compared with normal samples in the GSE180083 dataset. **(H)** *TLR4* expression in patients with CAD samples compared with normal samples in the GSE180083 dataset. **(I)** Performance of *HSPB1* expression in CAD diagnosis in the test dataset. **(J)** Performance of *LONP1* expression in CAD diagnosis in the GSE180083 dataset. **(K)** Performance of *CBS* expression in CAD diagnosis in the GSE180083 dataset. **(L)** Performance of *TLR4* expression in CAD diagnosis in the GSE180083 dataset. The distinction was considered good when the AUC value was between 0.8 and 0.9 and exceptional when the AUC value was greater than 0.9. ROC: receiver operating characteristic; AUC, area under the ROC curve.

We used the dataset to validate differences in the 4 subgroup‐specific genes *HSPB1, LONP1, CBS* and *TLR4* in the normal and CAD samples in the merged dataset and GSE180083 dataset. We found that *CBS* and *TLR4* were significantly upregulated and *HSPB1* and *LONP1* were significantly downregulated in patients with CAD in merged dataset and GSE180083 dataset (Figures 13A–H). To determine the diagnostic accuracy of this model, the area under the receiver operating characteristic curve (ROC) was determined using the “'ROC'” package. The area under the curve (AUC) value of *HSPB1, LONP1, CBS* and *TLR4* in GSE180083 dataset were 0.647, 0.624, 0.812, 0.825, respectively. The distinction was considered good when the area under the curve (AUC) value was between  0.8 and 0.9 and exceptional when the AUC value was greater than 0.9 (Rice and Harris, 2005).

## Discussion

Globally, CAD is a leading cause of death, with mortality predicted to reach 23.6 million by 2030 (Zhang L. et al., 2021). CAD is caused by various genetic and environmental factors, and the cumulative influence of these variables is critical. In this study, we investigated gene expression profiles from three GEO datasets of patients with CAD and normal controls. Additionally, we effectively divided the 352 patients with CAD into two clusters (clusters A and B) for the first time based on the expression of ferroptosis- and necroptosis-related DEGs. Further research identified functional modules or pathways unique to subgroups as a result of the categorization. Subsequently, we correctly categorized the 352 patients with CAD into gene clusters A and B for the second time based on the expression of four subgroup-specific genes. There was a significant correlation between clinical features and categorization. Moreover, when compared with the two gene clusters, patients in gene cluster A had a higher CAD index and were older, suggesting that gene cluster A was associated with more severe CAD. The typing results suggested that ferroptosis and necroptosis may play critical roles in CAD. Taken together, our results showed that categorization of patients with CAD was highly correlated with clinical features and certain functional modules or pathways.

Earlier studies have only explored the heterogeneity between subtypes (Peng et al., 2019; Zhang B. et al., 2021) and have not evaluated the molecular mechanisms responsible for this heterogeneity. The current study is the first to identify differences between subtypes based on ferroptosis- and necroptosis-related genes and to explore the associations between specific pathways and clinical characteristics in individuals with CAD within specific subgroups. We further performed functional enrichment analysis of the two clusters and found that the upregulated genes in cluster A were mainly enriched in neutrophil-related biological processes. According to current research, neutrophils are short-lived phagocytic cells expressing a wide range of physiologically active enzymes, including myeloperoxidases and proteinases. Leukocytosis and neutrophilia are independent risk factors for CAD. Additionally, C-X-C chemokine motif receptor 4 (CXCR4) and its ligand C-X-C chemokine ligand 12 play roles in neutrophil egress from the bone marrow and control neutrophil recruitment to atherosclerotic lesions (Zernecke et al., 2008). Chronic CXCR4 inhibition induces neutrophilia and increases the number of neutrophils in plaques, both of which are linked to apoptosis and a pro-inflammatory phenotype, suggesting that neutrophils may have pro-inflammatory roles in atherosclerosis (Zernecke et al., 2008). Furthermore, we found that cluster A was mainly related to neutrophils by contributing to the inflammatory response in atherosclerosis, suggesting that neutrophil-related pathways, which lead to further inflammatory infiltration, could explain the differences in CAD indices between the clusters. Neutrophil activation is involved in the immunological response, neutrophil-mediated immunity, and atherosclerosis development. In this study, we observed associations among older age, neutrophil pathway activation, and disease severity in cluster A, indicating that an aberrant neutrophil pathway in older patients with CAD may result in severe disease. In addition, upregulated genes in cluster B were mainly enriched in the B-cell receptor signaling pathway and antigen receptor-mediated signaling pathway. B cells were initially detected inside the adventitia in atherosclerotic lesions, and immunoglobulin-positive cells were identified within atherosclerotic plaques (Galkina and Ley, 2007a). The functions of B cells in mediating the immune response in atherosclerosis have recently been investigated. Splenectomy was shown to exacerbate atherosclerosis in ApoE^−/−^ mice, accompanied by a decrease in anti-ox-LDL antibody levels. Adoptive transfer of splenic B cells from atherosclerosis-prone ApoE^−/−^ animals into young ApoE^−/−^ recipients was also shown to prevent atherosclerosis (Caligiuri et al., 2002). These findings suggest that atheroprotective immunity may expand during the development of atherosclerosis and that B cells or their immunoglobulin products may play protective roles. From these results, we inferred that the severity of CAD was lower in individuals in cluster B than in those in cluster A, mainly because of the protective effects of B cells. In addition, the typing results showed that the main genes highly expressed in cluster A were ferroptosis-related genes (e.g., *NCF2*, *CBS*, *FTL*, *MAP3K5*, *MAPK14*, *STAT3*, *PGD*, and *TLR4*); thus, ferroptosis was closely associated with the progression of CAD. Current research indicates that ferroptosis and necroptosis differ in morphological characteristics, developmental steps, and key regulators, inducers, and inhibitors. Nonetheless, accumulating evidence suggests that significant cross-talk exists between ferroptosis and necroptosis (Fearns et al., 2006; Xu et al., 2017; Maher et al., 2018; Wang et al., 2018; Proneth and Conrad, 2019; Zhou et al., 2020; Chen C. et al., 2021). In this research, although the last significant genes identified in the analysis were all related to ferroptosis, it cannot be ruled out that necroptosis plays a similar role. The mechanism of the interaction between necroptosis and ferroptosis in CAD should be further investigated. Elucidating this mechanism could provide new perspectives to support advances in CAD treatment.

In addition, with additional exploration of the mechanisms of CAD, monocytes (Lessner et al., 2002), macrophages (Stoneman et al., 2007), mast cells (Bot et al., 2007), neutrophils (Zernecke et al., 2008), T cells (Galkina and Ley, 2007b), natural killer cells (Schiller et al., 2002), and dendritic cells (Bobryshev and Lord, 1995) have been found to be closely associated with the development and progression of CAD. According to the results of our analysis by ssGSEA, the proportion of most immune cells in cluster A was significantly increased, which further explained the severity of the CAD disease in cluster A and also further confirming that the severity of CAD may be associated with degree of immune cell infiltration. Furthermore, by studying the correlations of ferroptosis- and necroptosis-related DEGs with immune cells, we were surprised to find high expression in cluster A and that all DEGs were positively correlated with the aforementioned immune cells and functioned to promote the infiltration of various immune cells. Therefore, we further confirmed the important roles of ferroptosis and necroptosis in CAD progression as reported in Dominic (Del Re et al., 2019).

In this study, we next evaluated DEGs among different subgroups and found that all four significant genes were ferroptosis-related genes. To further validate the roles of these four genes in CAD progression, we typed the four genes and found that the results of typing, clinical features, and immune cell differences between typing were highly similar to the previous typing results, demonstrating the significant roles of these four genes in CAD progression. *LONP1* is an important mitochondrial target that is regulated by lipid-induced protein kinase R-like endoplasmic reticulum kinase/eukaryotic initiation factor 2α signaling in macrophages and in lesions (Tian et al., 2011; Crewe et al., 2017). Acute cell stresses, such as hypoxia, oxidative stress, food restriction, and the unfolded protein response at the endoplasmic reticulum, have been found to upregulate LonP1 (Hori et al., 2002; Sepuri et al., 2017). Venkatesh et al. (Venkatesh et al., 2019) showed that LonP1 is an endogenous cardioprotective mediator. Furthermore, overexpression of LonP1 protects the heart from injury by limiting oxidative damage to proteins and lipids, maintaining mitochondrial redox equilibrium, and reprogramming bioenergetics by decreasing complex I content and activity. Mechanisms that enhance LonP1 expression may protect the myocardium from cardiac stress and ischemia/reperfusion damage. Thus, the abovementioned results demonstrate that upregulation of LONP1 may inhibit the progression of CAD. HSPB1 is a well-known small heat shock protein that works as an oligomer and phosphorylated dimer (Mohammad et al., 2018). In rat cardiomyocytes, HSPB1 improves the reductive activity of endogenous glutathione (GSH) reductase/GSH/GSH peroxidase and thioredoxin/peroxiredoxin antioxidant systems, confirming its role in protein oxidation resistance (Liu et al., 2019). Kraemer et al. showed that HSPB1 is upregulated and phosphorylated in ST-elevation myocardial infarction platelets. Additionally, expression of HSPB1 in cardiomyocytes is necessary for wound healing after myocardial infarction, suggesting that this protein may be a target for enhancement of repair after myocardial infarction (Wang et al., 2019). Notably, HSPB1 generates homologous oxidized HSPB1 as a result of its own unique cysteine and subsequently acts as an antioxidant *in vitro* (Rajagopal et al., 2015). Thus, the abovementioned results also demonstrate that upregulation of HSPB1 may inhibit the progression of CAD. The results of our study showed that LONP1 and HSPB1 were significantly upregulated in cluster B and that the severity of CAD was lower in cluster B than in cluster A, further demonstrating the protective roles of LONP1 and HSPB1 in the progression of CAD. However, this requires our follow-up further experiments to confirm the specific protective mechanism of these two genes in CAD progression.

Interestingly, our analysis also found two genes that were significantly elevated in CAD samples, CBS and TLR4. CBS is a critical enzyme involved in the trans-sulfuration pathway via catabolism of homocysteine (Hcy). Mutations in CBS have been found in homocystinuric individuals and are related to thrombosis and increased plasma Hcy levels (Gaustadnes et al., 2000). Furthermore, Hcy increases atherogenesis by promoting vascular smooth muscle cell proliferation (Tsai et al., 1996), limiting endothelial cell growth and re-endothelialization after damage, decreasing endothelial relaxation, accelerating neointimal formation (Wang et al., 1997; Wang et al., 2002), and reducing high-density lipoprotein production (Liao et al., 2006). The immunological response elicited by Hcy is intricately associated with cardiovascular disease. Thus, upregulation of CBS promotes the onset and progression of CAD. TLR4 is a member of the Toll-like receptor (TLR) family and is involved in the progression of atherosclerosis, including monocyte activation, endothelial cell injury, vascular smooth muscle cell fibrosis, and macrophage and foam cell production (Xu et al., 2001). TLR4 expression is higher in CAD plaques than in controls (Edfeldt et al., 2002). In hypercholesterolemic ApoE^−/−^ mice, TLR4 knockout reduces lesion size, lipid content, and macrophage infiltration (Michelsen et al., 2004; Choi et al., 2009). Additionally, in diabetic ApoE^−/−^ mice, administration of TLR4 antagonist reduces atherosclerotic lesions, blocks inflammatory molecule production, and decreases monocyte and macrophage content (Lu et al., 2013). Under lipid-rich conditions, endogenous TLR4 ligands activate monocytes and macrophages, causing widespread membrane ruffling, macropinocytosis, lipoprotein uptake, and foam cell production (Howell et al., 2011; Liu et al., 2012). The TLR4 signaling pathway is a potential anti-inflammatory and anti-atherosclerosis target. Importantly, TLR4 expression has been reported to be strongly correlated with the severity of CAD, as reflected by the number of coronary stenoses, and may be a clinically useful biomarker of the risk of cardiovascular disease (Shao et al., 2014). Thus, upregulation of TLR4 is involved in promoting the onset and progression of CAD. Taken together, these findings suggested that TLR4 and CBS play key roles in the development of atherosclerosis. Therefore, blocking TLR4 and CBS signaling may be beneficial in the treatment of CAD. In addition, PCA demonstrated that the subgroup-specific gene scores in cluster A or gene cluster A was higher than that in cluster B or gene cluster B. Finally, we found that the subgroup-specific upregulated genes *CBS* and *TLR4* were significantly upregulated in the disease group in the GSE180083 dataset and had very high diagnostic efficacy. Therefore, regarding the role of CBS and TLR4 in the progression of CAD, combined with the above-mentioned related research reports and the results of this study, it is confirmed that the two play an important role in the progression of CAD. And further study of their mechanism of action will provide favorable therapeutic targets for alleviating CAD progression.

Although our findings identified potential subgroups of patients with CAD based on molecular analyses of ferroptosis- and necroptosis-related genes and revealed the characteristics of each subtype, gene expression changes are not necessarily genetically driven or could be partially genetic and partially environmental. Additional, several limitations should be acknowledged. First, some clinical follow-up information was not available for the samples; therefore, we did not consider several important factors, such as the presence of patient comorbidities, when distinguishing among subgroups. Second, the consistency of the CAD subgroups should be confirmed using further analyses. Finally, the results were acquired only through bioinformatics analysis, and the results should be confirmed in further experiments.

## Conclusion

High expression of *CBS* and *TLR4* in CAD was associated with increased disease severity and may be promising diagnostic markers of CAD. By contrast, *LONP1* and *HSPB1* may delay CAD progression. The identification of genetic subgroups of patients with CAD has improved our understanding of the pathogenesis of CAD and has facilitated the development of potential methods for disease diagnosis, classification, and prognosis evaluation.

## Data Availability

The datasets presented in this study can be found in online repositories. The names of the repository/repositories and accession number(s) can be found in the article/Supplementary Material.

## Glossary

CAD: Coronary artery disease
DEGs: differentially expressed genes
CBS: cystathionine beta-synthase
TLR4: toll-like receptor 4
LONP1: lon peptidase 1, mitochondrial
HSPB1: heat shock protein family B (small) member 1
Fer-1: Ferrostatin-1
NCF2: neutrophil cytosolic factor 2
BNIP3: BCL2 interacting protein 3
FTL: ferritin light chain
RPL8: ribosomal protein L8
MAP3K5: mitogen-activated protein kinase kinase 5
MAPK14: mitogen-activated protein kinase 14
ELAVL1: ELAV-like RNA-binding protein 1
HIC1: HIC ZBTB transcriptional repressor 1
STAT3: signal transducer and activator of transcription 3
PGD: phosphogluconate dehydrogenase
SCP2: sterol carrier protein 2
SLC38A1: solute carrier family 38 member 1
MYB: MYB proto-oncogene, transcription factor
MTDH: metadherin
FADD: Fas associated *via* death domain
ITPK1: inositol-tetrakisphosphate 1-kinase
MYC: MYC proto-oncogene, bHLH transcription factor
TNFSF10: TNF superfamily member 10
DNMT1: DNA methyltransferase 1
BACH2: BTB domain and CNC homolog 2
LEF1: lymphoid enhancer binding factor 1
GO: gene ontology
ssGSEA: single-sample gene set enrichment analysis
PCA: principal component analysis
ANOVA: analysis of variance
ROC: receiver operating characteristic
AUC: area under the curve
CXCR4: C-X-C motif chemokine receptor 4
CXCL12: C-X-C motif chemokine ligand 12
oxLDL: oxidized low-density lipoprotein
Hcy: homocysteine
HHcy: high homocysteine.
