# Supplementary material for: Identification of molecular subtypes of coronary artery disease based on ferroptosis- and necroptosis-related genes
Source: Front Genet. 2022 Sep 20;13:870222. doi: 10.3389/fgene.2022.870222 (PMC9531137; doi:10.3389/fgene.2022.870222)
Supplement: Supplementary file 1 [file Table1.docx]

**Supplement Table1.** Ferroptosis-and Necroptosis-related genes.

| Genes | Cell type |
| --- | --- |
| PTGS2 | Ferroptosis |
| DUSP1 | Ferroptosis |
| NOS2 | Ferroptosis |
| NCF2 | Ferroptosis |
| MT3 | Ferroptosis |
| UBC | Ferroptosis |
| ALB | Ferroptosis |
| TXNRD1 | Ferroptosis |
| SRXN1 | Ferroptosis |
| GPX2 | Ferroptosis |
| BNIP3 | Ferroptosis |
| OXSR1 | Ferroptosis |
| SELENOS | Ferroptosis |
| ANGPTL7 | Ferroptosis |
| CHAC1 | Ferroptosis |
| SLC7A11 | Ferroptosis |
| DDIT4 | Ferroptosis |
| LOC284561 | Ferroptosis |
| ASNS | Ferroptosis |
| TSC22D3 | Ferroptosis |
| DDIT3 | Ferroptosis |
| JDP2 | Ferroptosis |
| SESN2 | Ferroptosis |
| SLC1A4 | Ferroptosis |
| PCK2 | Ferroptosis |
| TXNIP | Ferroptosis |
| VLDLR | Ferroptosis |
| GPT2 | Ferroptosis |
| PSAT1 | Ferroptosis |
| LURAP1L | Ferroptosis |
| SLC7A5 | Ferroptosis |
| HERPUD1 | Ferroptosis |
| XBP1 | Ferroptosis |
| ATF3 | Ferroptosis |
| SLC3A2 | Ferroptosis |
| CBS | Ferroptosis |
| ATF4 | Ferroptosis |
| ZNF419 | Ferroptosis |
| KLHL24 | Ferroptosis |
| TRIB3 | Ferroptosis |
| ZFP69B | Ferroptosis |
| ATP6V1G2 | Ferroptosis |
| VEGFA | Ferroptosis |
| GDF15 | Ferroptosis |
| TUBE1 | Ferroptosis |
| ARRDC3 | Ferroptosis |
| CEBPG | Ferroptosis |
| SNORA16A | Ferroptosis |
| RGS4 | Ferroptosis |
| BLOC1S5-TXNDC5 | Ferroptosis |
| LOC390705 | Ferroptosis |
| EIF2S1 | Ferroptosis |
| KIM-1 | Ferroptosis |
| IL6 | Ferroptosis |
| CXCL2 | Ferroptosis |
| RELA | Ferroptosis |
| HSD17B11 | Ferroptosis |
| AGPAT3 | Ferroptosis |
| SETD1B | Ferroptosis |
| HMOX1 | Ferroptosis |
| TF | Ferroptosis |
| FTL | Ferroptosis |
| RPL8 | Ferroptosis |
| ATP5MC3 | Ferroptosis |
| TFRC | Ferroptosis |
| MAFG | Ferroptosis |
| IL33 | Ferroptosis |
| FTH1 | Ferroptosis |
| SLC40A1 | Ferroptosis |
| GPX4 | Ferroptosis |
| HAMP | Ferroptosis |
| HSPB1 | Ferroptosis |
| NFE2L2 | Ferroptosis |
| STEAP3 | Ferroptosis |
| DRD5 | Ferroptosis |
| DRD4 | Ferroptosis |
| MAP3K5 | Ferroptosis |
| MAPK14 | Ferroptosis |
| SLC2A1 | Ferroptosis |
| SLC2A3 | Ferroptosis |
| SLC2A6 | Ferroptosis |
| SLC2A8 | Ferroptosis |
| SLC2A12 | Ferroptosis |
| GLUT13 | Ferroptosis |
| SLC2A14 | Ferroptosis |
| EIF2AK4 | Ferroptosis |
| ALOX5 | Ferroptosis |
| ALOX12 | Ferroptosis |
| ALOX15 | Ferroptosis |
| ACSF2 | Ferroptosis |
| IREB2 | Ferroptosis |
| HMGB1 | Ferroptosis |
| ELAVL1 | Ferroptosis |
| TFAP2C | Ferroptosis |
| SP1 | Ferroptosis |
| HBA1 | Ferroptosis |
| NNMT | Ferroptosis |
| PLIN4 | Ferroptosis |
| HIC1 | Ferroptosis |
| STMN1 | Ferroptosis |
| RRM2 | Ferroptosis |
| CAPG | Ferroptosis |
| HNF4A | Ferroptosis |
| NGB | Ferroptosis |
| YWHAE | Ferroptosis |
| GABPB1 | Ferroptosis |
| AURKA | Ferroptosis |
| MIR4715 | Ferroptosis |
| RIPK1 | Ferroptosis |
| PRDX1 | Ferroptosis |
| MIR30B | Ferroptosis |
| AKR1C1 | Ferroptosis |
| AKR1C2 | Ferroptosis |
| AKR1C3 | Ferroptosis |
| RB1 | Ferroptosis |
| HSF1 | Ferroptosis |
| GCLC | Ferroptosis |
| SQSTM1 | Ferroptosis |
| NQO1 | Ferroptosis |
| MUC1 | Ferroptosis |
| MT1G | Ferroptosis |
| CISD1 | Ferroptosis |
| FANCD2 | Ferroptosis |
| FTMT | Ferroptosis |
| HSPA5 | Ferroptosis |
| TP53 | Ferroptosis |
| HELLS | Ferroptosis |
| SCD | Ferroptosis |
| FADS2 | Ferroptosis |
| SRC | Ferroptosis |
| STAT3 | Ferroptosis |
| PML | Ferroptosis |
| MTOR | Ferroptosis |
| NFS1 | Ferroptosis |
| TP63 | Ferroptosis |
| CDKN1A | Ferroptosis |
| MIR137 | Ferroptosis |
| ENPP2 | Ferroptosis |
| VDAC2 | Ferroptosis |
| FH | Ferroptosis |
| CISD2 | Ferroptosis |
| MIR9-1 | Ferroptosis |
| MIR9-2 | Ferroptosis |
| MIR9-3 | Ferroptosis |
| ISCU | Ferroptosis |
| ACSL3 | Ferroptosis |
| OTUB1 | Ferroptosis |
| CD44 | Ferroptosis |
| LINC00336 | Ferroptosis |
| BRD4 | Ferroptosis |
| PRDX6 | Ferroptosis |
| MIR17 | Ferroptosis |
| NF2 | Ferroptosis |
| ARNTL | Ferroptosis |
| HIF1A | Ferroptosis |
| JUN | Ferroptosis |
| CA9 | Ferroptosis |
| TMBIM4 | Ferroptosis |
| PLIN2 | Ferroptosis |
| MIR212 | Ferroptosis |
| Fer1HCH | Ferroptosis |
| AIFM2 | Ferroptosis |
| LAMP2 | Ferroptosis |
| ZFP36 | Ferroptosis |
| PROM2 | Ferroptosis |
| CHMP5 | Ferroptosis |
| CHMP6 | Ferroptosis |
| CAV1 | Ferroptosis |
| GCH1 | Ferroptosis |
| CS | Ferroptosis |
| EMC2 | Ferroptosis |
| NOX1 | Ferroptosis |
| CYBB | Ferroptosis |
| NOX3 | Ferroptosis |
| NOX4 | Ferroptosis |
| NOX5 | Ferroptosis |
| DUOX1 | Ferroptosis |
| DUOX2 | Ferroptosis |
| G6PD | Ferroptosis |
| PGD | Ferroptosis |
| PIK3CA | Ferroptosis |
| FLT3 | Ferroptosis |
| SCP2 | Ferroptosis |
| ACSL4 | Ferroptosis |
| LPCAT3 | Ferroptosis |
| NRAS | Ferroptosis |
| KRAS | Ferroptosis |
| HRAS | Ferroptosis |
| TFR2 | Ferroptosis |
| SLC38A1 | Ferroptosis |
| SLC1A5 | Ferroptosis |
| GLS2 | Ferroptosis |
| GOT1 | Ferroptosis |
| CARS1 | Ferroptosis |
| KEAP1 | Ferroptosis |
| ATG5 | Ferroptosis |
| ATG7 | Ferroptosis |
| NCOA4 | Ferroptosis |
| ALOX12B | Ferroptosis |
| ALOX15B | Ferroptosis |
| ALOXE3 | Ferroptosis |
| PHKG2 | Ferroptosis |
| ACO1 | Ferroptosis |
| G6PDX | Ferroptosis |
| ULK1 | Ferroptosis |
| ATG3 | Ferroptosis |
| ATG4D | Ferroptosis |
| BECN1 | Ferroptosis |
| MAP1LC3A | Ferroptosis |
| GABARAPL2 | Ferroptosis |
| GABARAPL1 | Ferroptosis |
| ATG16L1 | Ferroptosis |
| WIPI1 | Ferroptosis |
| WIPI2 | Ferroptosis |
| SNX4 | Ferroptosis |
| ATG13 | Ferroptosis |
| ULK2 | Ferroptosis |
| SAT1 | Ferroptosis |
| EGFR | Ferroptosis |
| MAPK3 | Ferroptosis |
| MAPK1 | Ferroptosis |
| BID | Ferroptosis |
| ZEB1 | Ferroptosis |
| DPP4 | Ferroptosis |
| CDKN2A | Ferroptosis |
| PEBP1 | Ferroptosis |
| SOCS1 | Ferroptosis |
| CDO1 | Ferroptosis |
| MYB | Ferroptosis |
| MAPK8 | Ferroptosis |
| MAPK9 | Ferroptosis |
| LINC00472 | Ferroptosis |
| PRKAA2 | Ferroptosis |
| PRKAA1 | Ferroptosis |
| BAP1 | Ferroptosis |
| ABCC1 | Ferroptosis |
| MIR6852 | Ferroptosis |
| ACVR1B | Ferroptosis |
| TGFBR1 | Ferroptosis |
| EPAS1 | Ferroptosis |
| HILPDA | Ferroptosis |
| IFNG | Ferroptosis |
| ANO6 | Ferroptosis |
| LPIN1 | Ferroptosis |
| TNFAIP3 | Ferroptosis |
| TLR4 | Ferroptosis |
| ATM | Ferroptosis |
| YY1AP1 | Ferroptosis |
| EGLN2 | Ferroptosis |
| MIOX | Ferroptosis |
| TAZ | Ferroptosis |
| MTDH | Ferroptosis |
| IDH1 | Ferroptosis |
| SIRT1 | Ferroptosis |
| FBXW7 | Ferroptosis |
| PANX1 | Ferroptosis |
| DNAJB6 | Ferroptosis |
| BACH1 | Ferroptosis |
| LONP1 | Ferroptosis |
| FADD | Necroptosis |
| FAS | Necroptosis |
| FASLG | Necroptosis |
| MLKL | Necroptosis |
| RIPK1 | Necroptosis |
| RIPK3 | Necroptosis |
| TLR3 | Necroptosis |
| TNF | Necroptosis |
| TSC1 | Necroptosis |
| TRIM11 | Necroptosis |
| CASP8 | Necroptosis |
| ZBP1 | Necroptosis |
| MAPK8 | Necroptosis |
| IPMK | Necroptosis |
| ITPK1 | Necroptosis |
| SIRT3 | Necroptosis |
| MYC | Necroptosis |
| TNFRSF1A | Necroptosis |
| TNFSF10 | Necroptosis |
| TNFRSF1B | Necroptosis |
| TRAF2 | Necroptosis |
| PANX1 | Necroptosis |
| OTULIN | Necroptosis |
| CYLD | Necroptosis |
| USP22 | Necroptosis |
| MAP3K7 | Necroptosis |
| SQSTM1 | Necroptosis |
| STAT3 | Necroptosis |
| DIABLO | Necroptosis |
| DNMT1 | Necroptosis |
| CFLAR | Necroptosis |
| BRAF | Necroptosis |
| AXL | Necroptosis |
| ID1 | Necroptosis |
| CDKN2A | Necroptosis |
| HSPA4 | Necroptosis |
| BCL2 | Necroptosis |
| STUB1 | Necroptosis |
| FLT3 | Necroptosis |
| HAT1 | Necroptosis |
| SIRT2 | Necroptosis |
| SIRT1 | Necroptosis |
| PLK1 | Necroptosis |
| MPG | Necroptosis |
| BACH2 | Necroptosis |
| GATA3 | Necroptosis |
| MYCN | Necroptosis |
| ALK | Necroptosis |
| ATRX | Necroptosis |
| TERT | Necroptosis |
| SLC39A7 | Necroptosis |
| SPATA2 | Necroptosis |
| RNF31 | Necroptosis |
| IDH1 | Necroptosis |
| IDH2 | Necroptosis |
| KLF9 | Necroptosis |
| HDAC9 | Necroptosis |
| HSP90AA1 | Necroptosis |
| LEF1 | Necroptosis |
| BNIP3 | Necroptosis |
| CD40 | Necroptosis |
| BCL2L11 | Necroptosis |
| EGFR | Necroptosis |
| DDX58 | Necroptosis |
| TARDBP | Necroptosis |
| APP | Necroptosis |
| TNFRSF21 | Necroptosis |
