# Supplementary material for: Identification of molecular subtypes of coronary artery disease based on ferroptosis- and necroptosis-related genes
Source: Front Genet. 2022 Sep 20;13:870222. doi: 10.3389/fgene.2022.870222 (PMC9531137; doi:10.3389/fgene.2022.870222)
Supplement: Supplementary file 2 [file Table7.docx]

**Supplement Table7.** We used PCA algorithms to calculate the 4 subgroup-specific genes score for CAD samples.

| Samples | subgroup-specific genes score |
| --- | --- |
| GSM308600 | 2.418335212 |
| GSM308601 | 0.393001707 |
| GSM308603 | 1.842310548 |
| GSM308604 | 0.799846374 |
| GSM308605 | 2.095339034 |
| GSM308606 | 1.03090359 |
| GSM308607 | 3.894167492 |
| GSM308608 | 0.570898623 |
| GSM308609 | 1.212935021 |
| GSM308610 | -0.682786685 |
| GSM308611 | 1.372075618 |
| GSM308613 | -0.538653728 |
| GSM308614 | -2.420150726 |
| GSM308615 | -0.552590787 |
| GSM308616 | -2.335114781 |
| GSM308617 | -1.757967841 |
| GSM308618 | 1.050596451 |
| GSM308620 | -1.499682249 |
| GSM308622 | -0.757124043 |
| GSM308623 | 2.175602756 |
| GSM308625 | -0.771581886 |
| GSM308626 | 1.630740438 |
| GSM308627 | 0.187706246 |
| GSM308629 | -0.930424066 |
| GSM308630 | 0.711484499 |
| GSM308631 | 1.207597853 |
| GSM308632 | 0.06024822 |
| GSM308634 | -1.652949605 |
| GSM308637 | -0.646947191 |
| GSM308639 | 0.018101705 |
| GSM308640 | -2.103766119 |
| GSM308641 | -2.316708858 |
| GSM308642 | -0.012160932 |
| GSM308644 | 3.444483191 |
| GSM308645 | -1.514958875 |
| GSM308646 | 2.421702006 |
| GSM308647 | -1.949398743 |
| GSM308650 | 1.879782261 |
| GSM308652 | -0.677681711 |
| GSM308653 | 0.174086798 |
| GSM308655 | 0.638751547 |
| GSM308657 | -0.681065434 |
| GSM308658 | 0.800701359 |
| GSM308659 | 2.93305007 |
| GSM308664 | -3.242991443 |
| GSM308666 | 0.451423516 |
| GSM308667 | -0.771192865 |
| GSM308668 | 1.158716059 |
| GSM308669 | 0.68832316 |
| GSM308670 | -0.314799514 |
| GSM308671 | -0.396164511 |
| GSM308672 | 0.871430432 |
| GSM308673 | -1.282427071 |
| GSM308674 | 1.622327587 |
| GSM308675 | 0.709382635 |
| GSM308676 | -0.105579205 |
| GSM308678 | 0.83374493 |
| GSM308679 | -2.227350679 |
| GSM308681 | 0.830709652 |
| GSM308685 | -1.497101993 |
| GSM308686 | -0.706644336 |
| GSM308688 | -1.836258504 |
| GSM308689 | -0.582132418 |
| GSM308690 | -2.161245527 |
| GSM308691 | 0.117989731 |
| GSM308692 | 0.538023163 |
| GSM308693 | -1.915996048 |
| GSM308694 | 1.241975812 |
| GSM308695 | -0.00868924 |
| GSM308696 | 0.109075944 |
| GSM308697 | 0.432271449 |
| GSM308699 | -0.882567046 |
| GSM308700 | 0.576919876 |
| GSM308703 | 1.002584348 |
| GSM308704 | 1.234810982 |
| GSM308705 | 2.404563747 |
| GSM308707 | 2.412968125 |
| GSM308708 | -0.462871015 |
| GSM308710 | 0.375098969 |
| GSM308711 | -1.141203397 |
| GSM308715 | 1.17538649 |
| GSM308717 | 0.201394798 |
| GSM308718 | -0.53972557 |
| GSM308719 | 1.343386793 |
| GSM308723 | 1.88234101 |
| GSM308724 | 1.386117698 |
| GSM308725 | 2.922573919 |
| GSM308727 | -0.707390044 |
| GSM308734 | -2.892261811 |
| GSM308736 | -0.791006541 |
| GSM308737 | 0.646877502 |
| GSM308741 | -2.031004259 |
| GSM308742 | 2.931973417 |
| GSM308743 | -0.425234327 |
| GSM308745 | -0.560262245 |
| GSM308747 | 1.923013919 |
| GSM308752 | 0.307785894 |
| GSM308754 | -0.149436956 |
| GSM308755 | -1.140255449 |
| GSM308756 | -0.950910231 |
| GSM308759 | -1.763060169 |
| GSM308773 | 1.904249768 |
| GSM308775 | -0.960985335 |
| GSM308776 | 1.205548816 |
| GSM308778 | 3.099414034 |
| GSM308780 | 0.856984962 |
| GSM308781 | 2.485179945 |
| GSM308782 | 0.934869839 |
| GSM308783 | -0.266412737 |
| GSM308784 | -0.086767615 |
| GSM518690 | -0.636109321 |
| GSM518691 | -1.371189979 |
| GSM518692 | -0.320023435 |
| GSM518693 | -0.375173469 |
| GSM518694 | 0.890116779 |
| GSM518695 | 1.036866505 |
| GSM518696 | -0.597448719 |
| GSM518697 | -1.451476369 |
| GSM518698 | 1.368657483 |
| GSM518699 | 5.382468811 |
| GSM518700 | -1.511136688 |
| GSM518701 | -1.22461027 |
| GSM518702 | 5.556603526 |
| GSM518703 | -2.179007329 |
| GSM518704 | -1.342300199 |
| GSM518705 | -1.195173283 |
| GSM518706 | 1.852545981 |
| GSM518707 | 0.911218241 |
| GSM518708 | -0.323933201 |
| GSM518709 | -0.733892874 |
| GSM518710 | 0.366748093 |
| GSM518711 | -2.219087238 |
| GSM518712 | -1.234030473 |
| GSM518713 | -0.59123082 |
| GSM518714 | -1.372540713 |
| GSM518715 | -1.6352742 |
| GSM518716 | -0.297796025 |
| GSM518717 | 1.124262259 |
| GSM518718 | -1.285129487 |
| GSM518719 | -1.774138279 |
| GSM518720 | 7.592727501 |
| GSM518721 | 0.974947038 |
| GSM518722 | -2.712873018 |
| GSM518723 | 1.16603953 |
| GSM518724 | -0.790758657 |
| GSM518725 | 1.005547029 |
| GSM518726 | 1.770398357 |
| GSM518727 | -1.081429921 |
| GSM518728 | -1.087322294 |
| GSM518729 | -3.425811372 |
| GSM518730 | 0.914084272 |
| GSM518731 | 2.801485574 |
| GSM518732 | -1.473907096 |
| GSM518733 | 2.815193875 |
| GSM518734 | 2.382134369 |
| GSM518735 | 0.751665706 |
| GSM518736 | -0.674021804 |
| GSM518737 | 2.631225109 |
| GSM518738 | -0.264060273 |
| GSM518739 | 0.60849924 |
| GSM518740 | 2.608829777 |
| GSM518741 | 0.701527805 |
| GSM518742 | 0.862435612 |
| GSM518743 | -0.068585309 |
| GSM518744 | 1.205116454 |
| GSM518745 | 1.116470045 |
| GSM518746 | -0.822147262 |
| GSM518747 | 0.123234403 |
| GSM518748 | -1.055882314 |
| GSM518749 | -1.714503339 |
| GSM518750 | 1.809436251 |
| GSM518751 | 2.814109903 |
| GSM518752 | -0.324763364 |
| GSM518753 | -1.644911976 |
| GSM518754 | -3.610451451 |
| GSM518755 | 1.531429437 |
| GSM518756 | -0.824084503 |
| GSM518757 | 2.765686979 |
| GSM518758 | 0.563115611 |
| GSM518759 | 1.885277695 |
| GSM518760 | -1.83441707 |
| GSM518761 | 0.145798791 |
| GSM518762 | -4.168130099 |
| GSM518763 | -1.137505038 |
| GSM518764 | 0.441592373 |
| GSM518765 | -1.275562365 |
| GSM518766 | 0.393718933 |
| GSM518767 | 0.506619385 |
| GSM518768 | 1.066681068 |
| GSM518769 | -1.742247741 |
| GSM518770 | 0.148253437 |
| GSM518771 | 0.204807432 |
| GSM518772 | -2.071249386 |
| GSM518773 | -0.855030838 |
| GSM518774 | 2.704861347 |
| GSM518775 | 1.594565138 |
| GSM518776 | 0.424001429 |
| GSM518777 | -0.928077261 |
| GSM518778 | 0.685429413 |
| GSM518779 | 2.043167494 |
| GSM518780 | 1.280791922 |
| GSM518781 | -1.120039079 |
| GSM518782 | -2.268683918 |
| GSM518783 | 0.333468647 |
| GSM518784 | -0.424340435 |
| GSM518785 | -2.448473149 |
| GSM518786 | -1.645870902 |
| GSM518787 | -1.392248272 |
| GSM518788 | -1.689787857 |
| GSM518789 | -1.54286344 |
| GSM518790 | 1.725711638 |
| GSM518791 | -1.088988572 |
| GSM518792 | -0.310917784 |
| GSM518793 | 2.315649258 |
| GSM518794 | -0.199805318 |
| GSM518795 | 0.702486917 |
| GSM518796 | -1.836836649 |
| GSM518797 | -1.774994195 |
| GSM518798 | -0.368887713 |
| GSM518799 | -0.887565249 |
| GSM518800 | 0.192519376 |
| GSM518801 | -0.321678244 |
| GSM518802 | 1.440264818 |
| GSM518803 | 0.009098745 |
| GSM518804 | 0.100469044 |
| GSM518805 | 1.731681015 |
| GSM518806 | 0.378213522 |
| GSM518807 | -2.007355079 |
| GSM518808 | 0.445441695 |
| GSM518809 | -1.170788959 |
| GSM518810 | 1.582354875 |
| GSM518811 | -0.847761194 |
| GSM518812 | 1.287003892 |
| GSM518813 | 0.178118341 |
| GSM518814 | -1.129860983 |
| GSM518815 | 2.861545206 |
| GSM518816 | 3.657524952 |
| GSM518817 | -0.315403159 |
| GSM518818 | -0.545603168 |
| GSM518819 | 1.423694093 |
| GSM518820 | -0.360560422 |
| GSM518821 | 0.71954621 |
| GSM518822 | -0.041180083 |
| GSM518823 | 1.046202236 |
| GSM518824 | 2.974131857 |
| GSM518825 | 1.654978317 |
| GSM518826 | -0.322869032 |
| GSM518827 | 1.477289408 |
| GSM518828 | 0.105635645 |
| GSM518829 | 0.154711488 |
| GSM518830 | -0.835273444 |
| GSM518831 | -0.890656477 |
| GSM518832 | 3.59951644 |
| GSM518885 | 2.611773502 |
| GSM518887 | -0.76362705 |
| GSM518889 | 1.222862826 |
| GSM518891 | -0.464492282 |
| GSM518893 | 0.179763371 |
| GSM518895 | 1.091714636 |
| GSM518897 | -0.992610647 |
| GSM518899 | -2.241110187 |
| GSM518901 | -0.238907062 |
| GSM518903 | -2.875287276 |
| GSM518905 | 2.997369954 |
| GSM518907 | -0.006516897 |
| GSM518909 | -2.124762464 |
| GSM518911 | -0.140477852 |
| GSM518913 | 2.078497151 |
| GSM518915 | -0.336670156 |
| GSM518917 | -0.805438258 |
| GSM518919 | -3.033968416 |
| GSM518921 | -0.371569069 |
| GSM518923 | -1.935147251 |
| GSM518925 | 1.680844368 |
| GSM518927 | -0.071092037 |
| GSM518929 | -0.635160512 |
| GSM518931 | -1.696666824 |
| GSM518933 | 0.34880227 |
| GSM518935 | -0.251317571 |
| GSM518937 | 0.992603632 |
| GSM518939 | 0.496488727 |
| GSM518941 | 1.522527324 |
| GSM518943 | 2.624704295 |
| GSM518945 | -2.049363416 |
| GSM518947 | 2.82201468 |
| GSM518949 | 2.425302206 |
| GSM518951 | 0.822822568 |
| GSM518953 | 1.913670827 |
| GSM518955 | 0.956639997 |
| GSM518957 | 2.867139984 |
| GSM518959 | 1.376537769 |
| GSM518961 | 0.69959113 |
| GSM518963 | 0.669304292 |
| GSM518965 | -1.452164009 |
| GSM518967 | 0.168295225 |
| GSM518969 | 0.317735354 |
| GSM518971 | 0.317492297 |
| GSM518973 | -0.26814328 |
| GSM518975 | 1.116717789 |
| GSM518977 | 2.100724398 |
| GSM518979 | 1.310741934 |
| GSM518981 | 3.930291243 |
| GSM518983 | 0.847593181 |
| GSM518985 | -2.732963794 |
| GSM518987 | -1.085401204 |
| GSM518989 | -1.374127617 |
| GSM518991 | 1.41662133 |
| GSM518993 | 0.325671988 |
| GSM518995 | -1.470605316 |
| GSM518997 | -0.884789325 |
| GSM518999 | -2.649573946 |
| GSM519001 | 1.509364767 |
| GSM519003 | 2.711464576 |
| GSM519005 | 1.674111796 |
| GSM519007 | -1.339387366 |
| GSM519009 | 1.42716475 |
| GSM519011 | -0.442787634 |
| GSM519013 | -0.522325699 |
| GSM519015 | -1.1110618 |
| GSM519017 | -1.130015115 |
| GSM519019 | -2.469420233 |
| GSM519021 | 0.264214198 |
| GSM519023 | 0.773149807 |
| GSM519025 | 0.36478765 |
| GSM519027 | 2.142166133 |
| GSM519029 | -0.43620804 |
| GSM519031 | -0.24806168 |
| GSM519033 | 5.784576012 |
| GSM519035 | -0.096816687 |
| GSM519037 | -1.992162755 |
| GSM519039 | -0.149105986 |
| GSM519041 | -1.354749296 |
| GSM519043 | -1.2176427 |
| GSM519045 | 0.463821107 |
| GSM519047 | -0.049789215 |
| GSM519049 | 0.429832824 |
| GSM519051 | -0.980345952 |
| GSM519053 | 0.415125372 |
| GSM519055 | 0.330321375 |
| GSM519057 | 4.146130545 |
| GSM519059 | -1.433329594 |
| GSM519061 | -0.290642097 |
| GSM519063 | -1.317777162 |
| GSM519065 | -1.338098038 |
| GSM519067 | 0.100986783 |
| GSM519069 | -2.169677796 |
| GSM519071 | 2.8257979 |
| GSM519073 | 0.126589098 |
| GSM519075 | 4.615095952 |
| GSM519077 | 2.832331292 |
| GSM519079 | -0.295276988 |
| GSM519081 | 0.959300533 |
