# Supplementary material for: Identification of molecular subtypes of coronary artery disease based on ferroptosis- and necroptosis-related genes
Source: Front Genet. 2022 Sep 20;13:870222. doi: 10.3389/fgene.2022.870222 (PMC9531137; doi:10.3389/fgene.2022.870222)
Supplement: Supplementary file 3 [file Table2.docx]

**Supplement Table 2.** Information on the characteristics of the GSE12288, GSE20680, GSE20681 and [GSE68506](https://www.ncbi.nlm.nih.gov/geo/query/acc.cgi?acc=GSE68506).

| Samples | Status | Gender | Age(year) | CAD_index | GPL |
| --- | --- | --- | --- | --- | --- |
| GSE12288 |  |  |  |  | GPL96 |
| GSM308600 | CAD | Male | 47 | 63 | GPL96 |
| GSM308601 | CAD | Male | 61 | 63 | GPL96 |
| GSM308603 | CAD | Male | 61 | 32 | GPL96 |
| GSM308604 | CAD | Male | 63 | 63 | GPL96 |
| GSM308605 | CAD | Male | 65 | 32 | GPL96 |
| GSM308606 | CAD | Male | 66 | 37 | GPL96 |
| GSM308607 | CAD | Male | 56 | 37 | GPL96 |
| GSM308608 | CAD | Male | 67 | 63 | GPL96 |
| GSM308609 | CAD | Male | 54 | 32 | GPL96 |
| GSM308610 | CAD | Male | 53 | 74 | GPL96 |
| GSM308611 | CAD | Male | 50 | 63 | GPL96 |
| GSM308613 | CAD | Male | 55 | 23 | GPL96 |
| GSM308614 | CAD | Male | 54 | 32 | GPL96 |
| GSM308615 | CAD | Male | 50 | 42 | GPL96 |
| GSM308616 | CAD | Male | 57 | 23 | GPL96 |
| GSM308617 | CAD | Male | 59 | 82 | GPL96 |
| GSM308618 | CAD | Male | 42 | 32 | GPL96 |
| GSM308620 | CAD | Male | 52 | 48 | GPL96 |
| GSM308622 | CAD | Male | 59 | 23 | GPL96 |
| GSM308623 | CAD | Male | 44 | 48 | GPL96 |
| GSM308625 | CAD | Male | 49 | 48 | GPL96 |
| GSM308626 | CAD | Male | 46 | 42 | GPL96 |
| GSM308627 | CAD | Male | 49 | 32 | GPL96 |
| GSM308629 | CAD | Male | 38 | 32 | GPL96 |
| GSM308630 | CAD | Male | 45 | 32 | GPL96 |
| GSM308631 | CAD | Male | 48 | 48 | GPL96 |
| GSM308632 | CAD | Male | 40 | 74 | GPL96 |
| GSM308634 | CAD | Male | 51 | 42 | GPL96 |
| GSM308637 | CAD | Male | 50 | 48 | GPL96 |
| GSM308639 | CAD | Male | 48 | 32 | GPL96 |
| GSM308640 | CAD | Male | 54 | 63 | GPL96 |
| GSM308641 | CAD | Male | 48 | 42 | GPL96 |
| GSM308642 | CAD | Male | 45 | 100 | GPL96 |
| GSM308644 | CAD | Male | 42 | 37 | GPL96 |
| GSM308645 | CAD | Male | 61 | 100 | GPL96 |
| GSM308646 | CAD | Male | 57 | 37 | GPL96 |
| GSM308647 | CAD | Male | 46 | 48 | GPL96 |
| GSM308650 | CAD | Male | 63 | 67 | GPL96 |
| GSM308652 | CAD | Male | 54 | 32 | GPL96 |
| GSM308653 | CAD | Male | 53 | 23 | GPL96 |
| GSM308655 | CAD | Male | 63 | 63 | GPL96 |
| GSM308657 | CAD | Male | 52 | 32 | GPL96 |
| GSM308658 | CAD | Male | 55 | 48 | GPL96 |
| GSM308659 | CAD | Male | 57 | 100 | GPL96 |
| GSM308664 | CAD | Male | 56 | 74 | GPL96 |
| GSM308666 | CAD | Male | 63 | 37 | GPL96 |
| GSM308667 | CAD | Male | 62 | 74 | GPL96 |
| GSM308668 | CAD | Male | 44 | 63 | GPL96 |
| GSM308669 | CAD | Male | 54 | 48 | GPL96 |
| GSM308670 | CAD | Male | 60 | 74 | GPL96 |
| GSM308671 | CAD | Male | 56 | 32 | GPL96 |
| GSM308672 | CAD | Male | 51 | 23 | GPL96 |
| GSM308673 | CAD | Male | 55 | 42 | GPL96 |
| GSM308674 | CAD | Male | 40 | 32 | GPL96 |
| GSM308675 | CAD | Male | 52 | 42 | GPL96 |
| GSM308676 | CAD | Male | 52 | 32 | GPL96 |
| GSM308678 | CAD | Male | 67 | 63 | GPL96 |
| GSM308679 | CAD | Male | 55 | 63 | GPL96 |
| GSM308681 | CAD | Male | 54 | 23 | GPL96 |
| GSM308685 | CAD | Male | 50 | 67 | GPL96 |
| GSM308686 | CAD | Male | 51 | 32 | GPL96 |
| GSM308688 | CAD | Male | 60 | 32 | GPL96 |
| GSM308689 | CAD | Male | 59 | 74 | GPL96 |
| GSM308690 | CAD | Male | 52 | 56 | GPL96 |
| GSM308691 | CAD | Male | 57 | 67 | GPL96 |
| GSM308692 | CAD | Male | 54 | 100 | GPL96 |
| GSM308693 | CAD | Male | 52 | 23 | GPL96 |
| GSM308694 | CAD | Male | 48 | 32 | GPL96 |
| GSM308695 | CAD | Male | 54 | 63 | GPL96 |
| GSM308696 | CAD | Male | 60 | 32 | GPL96 |
| GSM308697 | CAD | Male | 55 | 32 | GPL96 |
| GSM308699 | CAD | Male | 52 | 48 | GPL96 |
| GSM308700 | CAD | Male | 67 | 63 | GPL96 |
| GSM308703 | CAD | Male | 63 | 74 | GPL96 |
| GSM308704 | CAD | Female | 60 | 74 | GPL96 |
| GSM308705 | CAD | Female | 43 | 32 | GPL96 |
| GSM308707 | CAD | Male | 67 | 74 | GPL96 |
| GSM308708 | CAD | Male | 67 | 63 | GPL96 |
| GSM308710 | CAD | Male | 58 | 63 | GPL96 |
| GSM308711 | CAD | Male | 52 | 32 | GPL96 |
| GSM308715 | CAD | Female | 48 | 48 | GPL96 |
| GSM308717 | CAD | Male | 62 | 32 | GPL96 |
| GSM308718 | CAD | Female | 67 | 32 | GPL96 |
| GSM308719 | CAD | Male | 52 | 32 | GPL96 |
| GSM308723 | CAD | Male | 53 | 63 | GPL96 |
| GSM308724 | CAD | Female | 54 | 82 | GPL96 |
| GSM308725 | CAD | Male | 68 | 82 | GPL96 |
| GSM308727 | CAD | Male | 52 | 63 | GPL96 |
| GSM308734 | CAD | Male | 54 | 32 | GPL96 |
| GSM308736 | CAD | Female | 50 | 32 | GPL96 |
| GSM308737 | CAD | Female | 55 | 32 | GPL96 |
| GSM308741 | CAD | Female | 58 | 48 | GPL96 |
| GSM308742 | CAD | Female | 51 | 48 | GPL96 |
| GSM308743 | CAD | Male | 67 | 74 | GPL96 |
| GSM308745 | CAD | Female | 53 | 23 | GPL96 |
| GSM308747 | CAD | Male | 64 | 67 | GPL96 |
| GSM308752 | CAD | Female | 54 | 23 | GPL96 |
| GSM308754 | CAD | Female | 60 | 23 | GPL96 |
| GSM308755 | CAD | Female | 62 | 23 | GPL96 |
| GSM308756 | CAD | Female | 44 | 32 | GPL96 |
| GSM308759 | CAD | Male | 52 | 32 | GPL96 |
| GSM308773 | CAD | Female | 53 | 32 | GPL96 |
| GSM308775 | CAD | Female | 49 | 100 | GPL96 |
| GSM308776 | CAD | Female | 62 | 67 | GPL96 |
| GSM308778 | CAD | Female | 65 | 32 | GPL96 |
| GSM308780 | CAD | Female | 37 | 32 | GPL96 |
| GSM308781 | CAD | Female | 64 | 32 | GPL96 |
| GSM308782 | CAD | Female | 52 | 63 | GPL96 |
| GSM308783 | CAD | Female | 59 | 32 | GPL96 |
| GSM308784 | CAD | Male | 54 | 32 | GPL96 |
| GSM308566 | Normal | Male | 40 | 0 | GPL96 |
| GSM308567 | Normal | Male | 47 | 0 | GPL96 |
| GSM308568 | Normal | Male | 55 | 0 | GPL96 |
| GSM308569 | Normal | Male | 49 | 0 | GPL96 |
| GSM308570 | Normal | Male | 51 | 0 | GPL96 |
| GSM308571 | Normal | Male | 52 | 0 | GPL96 |
| GSM308572 | Normal | Male | 50 | 0 | GPL96 |
| GSM308573 | Normal | Male | 47 | 0 | GPL96 |
| GSM308574 | Normal | Male | 47 | 0 | GPL96 |
| GSM308575 | Normal | Male | 61 | 0 | GPL96 |
| GSM308576 | Normal | Male | 46 | 0 | GPL96 |
| GSM308577 | Normal | Male | 50 | 0 | GPL96 |
| GSM308578 | Normal | Male | 53 | 0 | GPL96 |
| GSM308579 | Normal | Male | 56 | 0 | GPL96 |
| GSM308580 | Normal | Male | 42 | 0 | GPL96 |
| GSM308581 | Normal | Male | 49 | 0 | GPL96 |
| GSM308582 | Normal | Male | 47 | 0 | GPL96 |
| GSM308583 | Normal | Male | 63 | 0 | GPL96 |
| GSM308584 | Normal | Male | 53 | 0 | GPL96 |
| GSM308585 | Normal | Male | 67 | 0 | GPL96 |
| GSM308586 | Normal | Male | 46 | 0 | GPL96 |
| GSM308587 | Normal | Male | 50 | 0 | GPL96 |
| GSM308588 | Normal | Male | 51 | 0 | GPL96 |
| GSM308589 | Normal | Male | 45 | 0 | GPL96 |
| GSM308590 | Normal | Male | 58 | 0 | GPL96 |
| GSM308591 | Normal | Male | 59 | 0 | GPL96 |
| GSM308592 | Normal | Male | 37 | 0 | GPL96 |
| GSM308593 | Normal | Male | 56 | 0 | GPL96 |
| GSM308594 | Normal | Male | 53 | 0 | GPL96 |
| GSM308595 | Normal | Male | 61 | 0 | GPL96 |
| GSM308596 | Normal | Male | 51 | 0 | GPL96 |
| GSM308597 | Normal | Male | 42 | 0 | GPL96 |
| GSM308598 | Normal | Male | 67 | 0 | GPL96 |
| GSM308599 | Normal | Male | 52 | 0 | GPL96 |
| GSM308602 | Normal | Male | 40 | 0 | GPL96 |
| GSM308612 | Normal | Male | 57 | 0 | GPL96 |
| GSM308619 | Normal | Male | 50 | 0 | GPL96 |
| GSM308621 | Normal | Male | 60 | 0 | GPL96 |
| GSM308624 | Normal | Male | 65 | 0 | GPL96 |
| GSM308628 | Normal | Male | 51 | 0 | GPL96 |
| GSM308633 | Normal | Male | 51 | 0 | GPL96 |
| GSM308635 | Normal | Male | 52 | 0 | GPL96 |
| GSM308636 | Normal | Male | 46 | 0 | GPL96 |
| GSM308638 | Normal | Male | 57 | 0 | GPL96 |
| GSM308643 | Normal | Male | 52 | 0 | GPL96 |
| GSM308648 | Normal | Male | 63 | 0 | GPL96 |
| GSM308649 | Normal | Male | 58 | 0 | GPL96 |
| GSM308651 | Normal | Male | 63 | 0 | GPL96 |
| GSM308654 | Normal | Male | 57 | 0 | GPL96 |
| GSM308656 | Normal | Male | 42 | 0 | GPL96 |
| GSM308660 | Normal | Male | 62 | 0 | GPL96 |
| GSM308661 | Normal | Male | 44 | 0 | GPL96 |
| GSM308662 | Normal | Male | 47 | 0 | GPL96 |
| GSM308663 | Normal | Male | 54 | 0 | GPL96 |
| GSM308665 | Normal | Male | 47 | 0 | GPL96 |
| GSM308677 | Normal | Male | 50 | 0 | GPL96 |
| GSM308680 | Normal | Male | 39 | 0 | GPL96 |
| GSM308682 | Normal | Male | 54 | 0 | GPL96 |
| GSM308683 | Normal | Male | 61 | 0 | GPL96 |
| GSM308684 | Normal | Male | 60 | 0 | GPL96 |
| GSM308687 | Normal | Male | 46 | 0 | GPL96 |
| GSM308698 | Normal | Male | 39 | 0 | GPL96 |
| GSM308701 | Normal | Male | 57 | 0 | GPL96 |
| GSM308702 | Normal | Male | 50 | 0 | GPL96 |
| GSM308706 | Normal | Female | 68 | 0 | GPL96 |
| GSM308709 | Normal | Male | 55 | 0 | GPL96 |
| GSM308712 | Normal | Female | 43 | 0 | GPL96 |
| GSM308713 | Normal | Female | 54 | 0 | GPL96 |
| GSM308714 | Normal | Male | 40 | 0 | GPL96 |
| GSM308716 | Normal | Female | 59 | 0 | GPL96 |
| GSM308720 | Normal | Female | 46 | 0 | GPL96 |
| GSM308721 | Normal | Female | 48 | 0 | GPL96 |
| GSM308722 | Normal | Female | 54 | 0 | GPL96 |
| GSM308726 | Normal | Male | 54 | 0 | GPL96 |
| GSM308728 | Normal | Female | 44 | 0 | GPL96 |
| GSM308729 | Normal | Female | 55 | 0 | GPL96 |
| GSM308730 | Normal | Male | 63 | 0 | GPL96 |
| GSM308731 | Normal | Female | 52 | 0 | GPL96 |
| GSM308732 | Normal | Male | 50 | 0 | GPL96 |
| GSM308733 | Normal | Female | 56 | 0 | GPL96 |
| GSM308735 | Normal | Male | 42 | 0 | GPL96 |
| GSM308738 | Normal | Female | 50 | 0 | GPL96 |
| GSM308739 | Normal | Male | 61 | 0 | GPL96 |
| GSM308740 | Normal | Male | 60 | 0 | GPL96 |
| GSM308744 | Normal | Female | 45 | 0 | GPL96 |
| GSM308746 | Normal | Male | 56 | 0 | GPL96 |
| GSM308748 | Normal | Male | 44 | 0 | GPL96 |
| GSM308749 | Normal | Male | 59 | 0 | GPL96 |
| GSM308750 | Normal | Female | 47 | 0 | GPL96 |
| GSM308751 | Normal | Male | 54 | 0 | GPL96 |
| GSM308753 | Normal | Female | 56 | 0 | GPL96 |
| GSM308757 | Normal | Male | 49 | 0 | GPL96 |
| GSM308758 | Normal | Female | 47 | 0 | GPL96 |
| GSM308760 | Normal | Male | 56 | 0 | GPL96 |
| GSM308761 | Normal | Female | 50 | 0 | GPL96 |
| GSM308762 | Normal | Female | 50 | 0 | GPL96 |
| GSM308763 | Normal | Female | 37 | 0 | GPL96 |
| GSM308764 | Normal | Female | 64 | 0 | GPL96 |
| GSM308765 | Normal | Male | 51 | 0 | GPL96 |
| GSM308766 | Normal | Male | 42 | 0 | GPL96 |
| GSM308767 | Normal | Female | 43 | 0 | GPL96 |
| GSM308768 | Normal | Male | 37 | 0 | GPL96 |
| GSM308769 | Normal | Female | 52 | 0 | GPL96 |
| GSM308770 | Normal | Male | 42 | 0 | GPL96 |
| GSM308771 | Normal | Male | 66 | 0 | GPL96 |
| GSM308772 | Normal | Female | 58 | 0 | GPL96 |
| GSM308774 | Normal | Female | 62 | 0 | GPL96 |
| GSM308777 | Normal | Female | 47 | 0 | GPL96 |
| GSM308779 | Normal | Female | 49 | 0 | GPL96 |
| GSM308785 | Normal | Female | 59 | 0 | GPL96 |
| GSM308786 | Normal | Female | 62 | 0 | GPL96 |
| GSM308787 | Normal | Male | 52 | 0 | GPL96 |
| GSE20681 |  |  |  |  | GPL4133 |
| GSM518885 | CAD | Male | NA | NA | GPL4133 |
| GSM518886 | Normal | Male | NA | NA | GPL4133 |
| GSM518887 | CAD | Male | NA | NA | GPL4133 |
| GSM518888 | Normal | Male | NA | NA | GPL4133 |
| GSM518889 | CAD | Female | NA | NA | GPL4133 |
| GSM518890 | Normal | Female | NA | NA | GPL4133 |
| GSM518891 | CAD | Male | NA | NA | GPL4133 |
| GSM518892 | Normal | Male | NA | NA | GPL4133 |
| GSM518893 | CAD | Female | NA | NA | GPL4133 |
| GSM518894 | Normal | Female | NA | NA | GPL4133 |
| GSM518895 | CAD | Male | NA | NA | GPL4133 |
| GSM518896 | Normal | Male | NA | NA | GPL4133 |
| GSM518897 | CAD | Male | NA | NA | GPL4133 |
| GSM518898 | Normal | Male | NA | NA | GPL4133 |
| GSM518899 | CAD | Male | NA | NA | GPL4133 |
| GSM518900 | Normal | Male | NA | NA | GPL4133 |
| GSM518901 | CAD | Female | NA | NA | GPL4133 |
| GSM518902 | Normal | Female | NA | NA | GPL4133 |
| GSM518903 | CAD | Male | NA | NA | GPL4133 |
| GSM518904 | Normal | Male | NA | NA | GPL4133 |
| GSM518905 | CAD | Male | NA | NA | GPL4133 |
| GSM518906 | Normal | Male | NA | NA | GPL4133 |
| GSM518907 | CAD | Male | NA | NA | GPL4133 |
| GSM518908 | Normal | Male | NA | NA | GPL4133 |
| GSM518909 | CAD | Male | NA | NA | GPL4133 |
| GSM518910 | Normal | Male | NA | NA | GPL4133 |
| GSM518911 | CAD | Male | NA | NA | GPL4133 |
| GSM518912 | Normal | Male | NA | NA | GPL4133 |
| GSM518913 | CAD | Male | NA | NA | GPL4133 |
| GSM518914 | Normal | Male | NA | NA | GPL4133 |
| GSM518915 | CAD | Male | NA | NA | GPL4133 |
| GSM518916 | Normal | Male | NA | NA | GPL4133 |
| GSM518917 | CAD | Female | NA | NA | GPL4133 |
| GSM518918 | Normal | Female | NA | NA | GPL4133 |
| GSM518919 | CAD | Male | NA | NA | GPL4133 |
| GSM518920 | Normal | Male | NA | NA | GPL4133 |
| GSM518921 | CAD | Male | NA | NA | GPL4133 |
| GSM518922 | Normal | Male | NA | NA | GPL4133 |
| GSM518923 | CAD | Male | NA | NA | GPL4133 |
| GSM518924 | Normal | Male | NA | NA | GPL4133 |
| GSM518925 | CAD | Male | NA | NA | GPL4133 |
| GSM518926 | Normal | Male | NA | NA | GPL4133 |
| GSM518927 | CAD | Male | NA | NA | GPL4133 |
| GSM518928 | Normal | Male | NA | NA | GPL4133 |
| GSM518929 | CAD | Male | NA | NA | GPL4133 |
| GSM518930 | Normal | Male | NA | NA | GPL4133 |
| GSM518931 | CAD | Male | NA | NA | GPL4133 |
| GSM518932 | Normal | Male | NA | NA | GPL4133 |
| GSM518933 | CAD | Female | NA | NA | GPL4133 |
| GSM518934 | Normal | Female | NA | NA | GPL4133 |
| GSM518935 | CAD | Male | NA | NA | GPL4133 |
| GSM518936 | Normal | Male | NA | NA | GPL4133 |
| GSM518937 | CAD | Male | NA | NA | GPL4133 |
| GSM518938 | Normal | Male | NA | NA | GPL4133 |
| GSM518939 | CAD | Female | NA | NA | GPL4133 |
| GSM518940 | Normal | Female | NA | NA | GPL4133 |
| GSM518941 | CAD | Male | NA | NA | GPL4133 |
| GSM518942 | Normal | Male | NA | NA | GPL4133 |
| GSM518943 | CAD | Male | NA | NA | GPL4133 |
| GSM518944 | Normal | Male | NA | NA | GPL4133 |
| GSM518945 | CAD | Male | NA | NA | GPL4133 |
| GSM518946 | Normal | Male | NA | NA | GPL4133 |
| GSM518947 | CAD | Female | NA | NA | GPL4133 |
| GSM518948 | Normal | Female | NA | NA | GPL4133 |
| GSM518949 | CAD | Male | NA | NA | GPL4133 |
| GSM518950 | Normal | Male | NA | NA | GPL4133 |
| GSM518951 | CAD | Male | NA | NA | GPL4133 |
| GSM518952 | Normal | Male | NA | NA | GPL4133 |
| GSM518953 | CAD | Male | NA | NA | GPL4133 |
| GSM518954 | Normal | Male | NA | NA | GPL4133 |
| GSM518955 | CAD | Male | NA | NA | GPL4133 |
| GSM518956 | Normal | Male | NA | NA | GPL4133 |
| GSM518957 | CAD | Male | NA | NA | GPL4133 |
| GSM518958 | Normal | Male | NA | NA | GPL4133 |
| GSM518959 | CAD | Male | NA | NA | GPL4133 |
| GSM518960 | Normal | Male | NA | NA | GPL4133 |
| GSM518961 | CAD | Male | NA | NA | GPL4133 |
| GSM518962 | Normal | Male | NA | NA | GPL4133 |
| GSM518963 | CAD | Female | NA | NA | GPL4133 |
| GSM518964 | Normal | Female | NA | NA | GPL4133 |
| GSM518965 | CAD | Male | NA | NA | GPL4133 |
| GSM518966 | Normal | Male | NA | NA | GPL4133 |
| GSM518967 | CAD | Male | NA | NA | GPL4133 |
| GSM518968 | Normal | Male | NA | NA | GPL4133 |
| GSM518969 | CAD | Male | NA | NA | GPL4133 |
| GSM518970 | Normal | Male | NA | NA | GPL4133 |
| GSM518971 | CAD | Male | NA | NA | GPL4133 |
| GSM518972 | Normal | Male | NA | NA | GPL4133 |
| GSM518973 | CAD | Male | NA | NA | GPL4133 |
| GSM518974 | Normal | Male | NA | NA | GPL4133 |
| GSM518975 | CAD | Female | NA | NA | GPL4133 |
| GSM518976 | Normal | Female | NA | NA | GPL4133 |
| GSM518977 | CAD | Male | NA | NA | GPL4133 |
| GSM518978 | Normal | Male | NA | NA | GPL4133 |
| GSM518979 | CAD | Male | NA | NA | GPL4133 |
| GSM518980 | Normal | Male | NA | NA | GPL4133 |
| GSM518981 | CAD | Male | NA | NA | GPL4133 |
| GSM518982 | Normal | Male | NA | NA | GPL4133 |
| GSM518983 | CAD | Male | NA | NA | GPL4133 |
| GSM518984 | Normal | Male | NA | NA | GPL4133 |
| GSM518985 | CAD | Female | NA | NA | GPL4133 |
| GSM518986 | Normal | Female | NA | NA | GPL4133 |
| GSM518987 | CAD | Male | NA | NA | GPL4133 |
| GSM518988 | Normal | Male | NA | NA | GPL4133 |
| GSM518989 | CAD | Male | NA | NA | GPL4133 |
| GSM518990 | Normal | Male | NA | NA | GPL4133 |
| GSM518991 | CAD | Female | NA | NA | GPL4133 |
| GSM518992 | Normal | Female | NA | NA | GPL4133 |
| GSM518993 | CAD | Male | NA | NA | GPL4133 |
| GSM518994 | Normal | Male | NA | NA | GPL4133 |
| GSM518995 | CAD | Male | NA | NA | GPL4133 |
| GSM518996 | Normal | Male | NA | NA | GPL4133 |
| GSM518997 | CAD | Male | NA | NA | GPL4133 |
| GSM518998 | Normal | Male | NA | NA | GPL4133 |
| GSM518999 | CAD | Male | NA | NA | GPL4133 |
| GSM519000 | Normal | Male | NA | NA | GPL4133 |
| GSM519001 | CAD | Male | NA | NA | GPL4133 |
| GSM519002 | Normal | Male | NA | NA | GPL4133 |
| GSM519003 | CAD | Male | NA | NA | GPL4133 |
| GSM519004 | Normal | Male | NA | NA | GPL4133 |
| GSM519005 | CAD | Female | NA | NA | GPL4133 |
| GSM519006 | Normal | Female | NA | NA | GPL4133 |
| GSM519007 | CAD | Male | NA | NA | GPL4133 |
| GSM519008 | Normal | Male | NA | NA | GPL4133 |
| GSM519009 | CAD | Female | NA | NA | GPL4133 |
| GSM519010 | Normal | Female | NA | NA | GPL4133 |
| GSM519011 | CAD | Male | NA | NA | GPL4133 |
| GSM519012 | Normal | Male | NA | NA | GPL4133 |
| GSM519013 | CAD | Male | NA | NA | GPL4133 |
| GSM519014 | Normal | Male | NA | NA | GPL4133 |
| GSM519015 | CAD | Male | NA | NA | GPL4133 |
| GSM519016 | Normal | Male | NA | NA | GPL4133 |
| GSM519017 | CAD | Male | NA | NA | GPL4133 |
| GSM519018 | Normal | Male | NA | NA | GPL4133 |
| GSM519019 | CAD | Female | NA | NA | GPL4133 |
| GSM519020 | Normal | Female | NA | NA | GPL4133 |
| GSM519021 | CAD | Male | NA | NA | GPL4133 |
| GSM519022 | Normal | Male | NA | NA | GPL4133 |
| GSM519023 | CAD | Male | NA | NA | GPL4133 |
| GSM519024 | Normal | Male | NA | NA | GPL4133 |
| GSM519025 | CAD | Male | NA | NA | GPL4133 |
| GSM519026 | Normal | Male | NA | NA | GPL4133 |
| GSM519027 | CAD | Male | NA | NA | GPL4133 |
| GSM519028 | Normal | Male | NA | NA | GPL4133 |
| GSM519029 | CAD | Female | NA | NA | GPL4133 |
| GSM519030 | Normal | Female | NA | NA | GPL4133 |
| GSM519031 | CAD | Male | NA | NA | GPL4133 |
| GSM519032 | Normal | Male | NA | NA | GPL4133 |
| GSM519033 | CAD | Female | NA | NA | GPL4133 |
| GSM519034 | Normal | Female | NA | NA | GPL4133 |
| GSM519035 | CAD | Male | NA | NA | GPL4133 |
| GSM519036 | Normal | Male | NA | NA | GPL4133 |
| GSM519037 | CAD | Male | NA | NA | GPL4133 |
| GSM519038 | Normal | Male | NA | NA | GPL4133 |
| GSM519039 | CAD | Female | NA | NA | GPL4133 |
| GSM519040 | Normal | Female | NA | NA | GPL4133 |
| GSM519041 | CAD | Female | NA | NA | GPL4133 |
| GSM519042 | Normal | Female | NA | NA | GPL4133 |
| GSM519043 | CAD | Female | NA | NA | GPL4133 |
| GSM519044 | Normal | Female | NA | NA | GPL4133 |
| GSM519045 | CAD | Male | NA | NA | GPL4133 |
| GSM519046 | Normal | Male | NA | NA | GPL4133 |
| GSM519047 | CAD | Female | NA | NA | GPL4133 |
| GSM519048 | Normal | Female | NA | NA | GPL4133 |
| GSM519049 | CAD | Male | NA | NA | GPL4133 |
| GSM519050 | Normal | Male | NA | NA | GPL4133 |
| GSM519051 | CAD | Male | NA | NA | GPL4133 |
| GSM519052 | Normal | Male | NA | NA | GPL4133 |
| GSM519053 | CAD | Female | NA | NA | GPL4133 |
| GSM519054 | Normal | Female | NA | NA | GPL4133 |
| GSM519055 | CAD | Male | NA | NA | GPL4133 |
| GSM519056 | Normal | Male | NA | NA | GPL4133 |
| GSM519057 | CAD | Male | NA | NA | GPL4133 |
| GSM519058 | Normal | Male | NA | NA | GPL4133 |
| GSM519059 | CAD | Male | NA | NA | GPL4133 |
| GSM519060 | Normal | Male | NA | NA | GPL4133 |
| GSM519061 | CAD | Female | NA | NA | GPL4133 |
| GSM519062 | Normal | Female | NA | NA | GPL4133 |
| GSM519063 | CAD | Male | NA | NA | GPL4133 |
| GSM519064 | Normal | Male | NA | NA | GPL4133 |
| GSM519065 | CAD | Female | NA | NA | GPL4133 |
| GSM519066 | Normal | Female | NA | NA | GPL4133 |
| GSM519067 | CAD | Female | NA | NA | GPL4133 |
| GSM519068 | Normal | Female | NA | NA | GPL4133 |
| GSM519069 | CAD | Male | NA | NA | GPL4133 |
| GSM519070 | Normal | Male | NA | NA | GPL4133 |
| GSM519071 | CAD | Male | NA | NA | GPL4133 |
| GSM519072 | Normal | Male | NA | NA | GPL4133 |
| GSM519073 | CAD | Male | NA | NA | GPL4133 |
| GSM519074 | Normal | Male | NA | NA | GPL4133 |
| GSM519075 | CAD | Male | NA | NA | GPL4133 |
| GSM519076 | Normal | Male | NA | NA | GPL4133 |
| GSM519077 | CAD | Male | NA | NA | GPL4133 |
| GSM519078 | Normal | Male | NA | NA | GPL4133 |
| GSM519079 | CAD | Male | NA | NA | GPL4133 |
| GSM519080 | Normal | Male | NA | NA | GPL4133 |
| GSM519081 | CAD | Male | NA | NA | GPL4133 |
| GSM519082 | Normal | Male | NA | NA | GPL4133 |
| GSE20860 |  |  |  |  | [GPL4133](https://www.ncbi.nlm.nih.gov/geo/query/acc.cgi?acc=GPL4133) |
| GSM518638 | Normal | NA | NA | NA | [GPL4133](https://www.ncbi.nlm.nih.gov/geo/query/acc.cgi?acc=GPL4133) |
| GSM518639 | Normal | NA | NA | NA | [GPL4133](https://www.ncbi.nlm.nih.gov/geo/query/acc.cgi?acc=GPL4133) |
| GSM518640 | Normal | NA | NA | NA | [GPL4133](https://www.ncbi.nlm.nih.gov/geo/query/acc.cgi?acc=GPL4133) |
| GSM518641 | Normal | NA | NA | NA | [GPL4133](https://www.ncbi.nlm.nih.gov/geo/query/acc.cgi?acc=GPL4133) |
| GSM518642 | Normal | NA | NA | NA | [GPL4133](https://www.ncbi.nlm.nih.gov/geo/query/acc.cgi?acc=GPL4133) |
| GSM518643 | Normal | NA | NA | NA | [GPL4133](https://www.ncbi.nlm.nih.gov/geo/query/acc.cgi?acc=GPL4133) |
| GSM518644 | Normal | NA | NA | NA | [GPL4133](https://www.ncbi.nlm.nih.gov/geo/query/acc.cgi?acc=GPL4133) |
| GSM518645 | Normal | NA | NA | NA | [GPL4133](https://www.ncbi.nlm.nih.gov/geo/query/acc.cgi?acc=GPL4133) |
| GSM518646 | Normal | NA | NA | NA | [GPL4133](https://www.ncbi.nlm.nih.gov/geo/query/acc.cgi?acc=GPL4133) |
| GSM518647 | Normal | NA | NA | NA | [GPL4133](https://www.ncbi.nlm.nih.gov/geo/query/acc.cgi?acc=GPL4133) |
| GSM518648 | Normal | NA | NA | NA | [GPL4133](https://www.ncbi.nlm.nih.gov/geo/query/acc.cgi?acc=GPL4133) |
| GSM518649 | Normal | NA | NA | NA | [GPL4133](https://www.ncbi.nlm.nih.gov/geo/query/acc.cgi?acc=GPL4133) |
| GSM518650 | Normal | NA | NA | NA | [GPL4133](https://www.ncbi.nlm.nih.gov/geo/query/acc.cgi?acc=GPL4133) |
| GSM518651 | Normal | NA | NA | NA | [GPL4133](https://www.ncbi.nlm.nih.gov/geo/query/acc.cgi?acc=GPL4133) |
| GSM518652 | Normal | NA | NA | NA | [GPL4133](https://www.ncbi.nlm.nih.gov/geo/query/acc.cgi?acc=GPL4133) |
| GSM518653 | Normal | NA | NA | NA | [GPL4133](https://www.ncbi.nlm.nih.gov/geo/query/acc.cgi?acc=GPL4133) |
| GSM518654 | Normal | NA | NA | NA | [GPL4133](https://www.ncbi.nlm.nih.gov/geo/query/acc.cgi?acc=GPL4133) |
| GSM518655 | Normal | NA | NA | NA | [GPL4133](https://www.ncbi.nlm.nih.gov/geo/query/acc.cgi?acc=GPL4133) |
| GSM518656 | Normal | NA | NA | NA | [GPL4133](https://www.ncbi.nlm.nih.gov/geo/query/acc.cgi?acc=GPL4133) |
| GSM518657 | Normal | NA | NA | NA | [GPL4133](https://www.ncbi.nlm.nih.gov/geo/query/acc.cgi?acc=GPL4133) |
| GSM518658 | Normal | NA | NA | NA | [GPL4133](https://www.ncbi.nlm.nih.gov/geo/query/acc.cgi?acc=GPL4133) |
| GSM518659 | Normal | NA | NA | NA | [GPL4133](https://www.ncbi.nlm.nih.gov/geo/query/acc.cgi?acc=GPL4133) |
| GSM518660 | Normal | NA | NA | NA | [GPL4133](https://www.ncbi.nlm.nih.gov/geo/query/acc.cgi?acc=GPL4133) |
| GSM518661 | Normal | NA | NA | NA | [GPL4133](https://www.ncbi.nlm.nih.gov/geo/query/acc.cgi?acc=GPL4133) |
| GSM518662 | Normal | NA | NA | NA | [GPL4133](https://www.ncbi.nlm.nih.gov/geo/query/acc.cgi?acc=GPL4133) |
| GSM518663 | Normal | NA | NA | NA | [GPL4133](https://www.ncbi.nlm.nih.gov/geo/query/acc.cgi?acc=GPL4133) |
| GSM518664 | Normal | NA | NA | NA | [GPL4133](https://www.ncbi.nlm.nih.gov/geo/query/acc.cgi?acc=GPL4133) |
| GSM518665 | Normal | NA | NA | NA | [GPL4133](https://www.ncbi.nlm.nih.gov/geo/query/acc.cgi?acc=GPL4133) |
| GSM518666 | Normal | NA | NA | NA | [GPL4133](https://www.ncbi.nlm.nih.gov/geo/query/acc.cgi?acc=GPL4133) |
| GSM518667 | Normal | NA | NA | NA | [GPL4133](https://www.ncbi.nlm.nih.gov/geo/query/acc.cgi?acc=GPL4133) |
| GSM518668 | Normal | NA | NA | NA | [GPL4133](https://www.ncbi.nlm.nih.gov/geo/query/acc.cgi?acc=GPL4133) |
| GSM518669 | Normal | NA | NA | NA | [GPL4133](https://www.ncbi.nlm.nih.gov/geo/query/acc.cgi?acc=GPL4133) |
| GSM518670 | Normal | NA | NA | NA | [GPL4133](https://www.ncbi.nlm.nih.gov/geo/query/acc.cgi?acc=GPL4133) |
| GSM518671 | Normal | NA | NA | NA | [GPL4133](https://www.ncbi.nlm.nih.gov/geo/query/acc.cgi?acc=GPL4133) |
| GSM518672 | Normal | NA | NA | NA | [GPL4133](https://www.ncbi.nlm.nih.gov/geo/query/acc.cgi?acc=GPL4133) |
| GSM518673 | Normal | NA | NA | NA | [GPL4133](https://www.ncbi.nlm.nih.gov/geo/query/acc.cgi?acc=GPL4133) |
| GSM518674 | Normal | NA | NA | NA | [GPL4133](https://www.ncbi.nlm.nih.gov/geo/query/acc.cgi?acc=GPL4133) |
| GSM518675 | Normal | NA | NA | NA | [GPL4133](https://www.ncbi.nlm.nih.gov/geo/query/acc.cgi?acc=GPL4133) |
| GSM518676 | Normal | NA | NA | NA | [GPL4133](https://www.ncbi.nlm.nih.gov/geo/query/acc.cgi?acc=GPL4133) |
| GSM518677 | Normal | NA | NA | NA | [GPL4133](https://www.ncbi.nlm.nih.gov/geo/query/acc.cgi?acc=GPL4133) |
| GSM518678 | Normal | NA | NA | NA | [GPL4133](https://www.ncbi.nlm.nih.gov/geo/query/acc.cgi?acc=GPL4133) |
| GSM518679 | Normal | NA | NA | NA | [GPL4133](https://www.ncbi.nlm.nih.gov/geo/query/acc.cgi?acc=GPL4133) |
| GSM518680 | Normal | NA | NA | NA | [GPL4133](https://www.ncbi.nlm.nih.gov/geo/query/acc.cgi?acc=GPL4133) |
| GSM518681 | Normal | NA | NA | NA | [GPL4133](https://www.ncbi.nlm.nih.gov/geo/query/acc.cgi?acc=GPL4133) |
| GSM518682 | Normal | NA | NA | NA | [GPL4133](https://www.ncbi.nlm.nih.gov/geo/query/acc.cgi?acc=GPL4133) |
| GSM518683 | Normal | NA | NA | NA | [GPL4133](https://www.ncbi.nlm.nih.gov/geo/query/acc.cgi?acc=GPL4133) |
| GSM518684 | Normal | NA | NA | NA | [GPL4133](https://www.ncbi.nlm.nih.gov/geo/query/acc.cgi?acc=GPL4133) |
| GSM518685 | Normal | NA | NA | NA | [GPL4133](https://www.ncbi.nlm.nih.gov/geo/query/acc.cgi?acc=GPL4133) |
| GSM518686 | Normal | NA | NA | NA | [GPL4133](https://www.ncbi.nlm.nih.gov/geo/query/acc.cgi?acc=GPL4133) |
| GSM518687 | Normal | NA | NA | NA | [GPL4133](https://www.ncbi.nlm.nih.gov/geo/query/acc.cgi?acc=GPL4133) |
| GSM518688 | Normal | NA | NA | NA | [GPL4133](https://www.ncbi.nlm.nih.gov/geo/query/acc.cgi?acc=GPL4133) |
| GSM518689 | Normal | NA | NA | NA | [GPL4133](https://www.ncbi.nlm.nih.gov/geo/query/acc.cgi?acc=GPL4133) |
| GSM518690 | CAD | NA | NA | NA | [GPL4133](https://www.ncbi.nlm.nih.gov/geo/query/acc.cgi?acc=GPL4133) |
| GSM518691 | CAD | NA | NA | NA | [GPL4133](https://www.ncbi.nlm.nih.gov/geo/query/acc.cgi?acc=GPL4133) |
| GSM518692 | CAD | NA | NA | NA | [GPL4133](https://www.ncbi.nlm.nih.gov/geo/query/acc.cgi?acc=GPL4133) |
| GSM518693 | CAD | NA | NA | NA | [GPL4133](https://www.ncbi.nlm.nih.gov/geo/query/acc.cgi?acc=GPL4133) |
| GSM518694 | CAD | NA | NA | NA | [GPL4133](https://www.ncbi.nlm.nih.gov/geo/query/acc.cgi?acc=GPL4133) |
| GSM518695 | CAD | NA | NA | NA | [GPL4133](https://www.ncbi.nlm.nih.gov/geo/query/acc.cgi?acc=GPL4133) |
| GSM518696 | CAD | NA | NA | NA | [GPL4133](https://www.ncbi.nlm.nih.gov/geo/query/acc.cgi?acc=GPL4133) |
| GSM518697 | CAD | NA | NA | NA | [GPL4133](https://www.ncbi.nlm.nih.gov/geo/query/acc.cgi?acc=GPL4133) |
| GSM518698 | CAD | NA | NA | NA | [GPL4133](https://www.ncbi.nlm.nih.gov/geo/query/acc.cgi?acc=GPL4133) |
| GSM518699 | CAD | NA | NA | NA | [GPL4133](https://www.ncbi.nlm.nih.gov/geo/query/acc.cgi?acc=GPL4133) |
| GSM518700 | CAD | NA | NA | NA | [GPL4133](https://www.ncbi.nlm.nih.gov/geo/query/acc.cgi?acc=GPL4133) |
| GSM518701 | CAD | NA | NA | NA | [GPL4133](https://www.ncbi.nlm.nih.gov/geo/query/acc.cgi?acc=GPL4133) |
| GSM518702 | CAD | NA | NA | NA | [GPL4133](https://www.ncbi.nlm.nih.gov/geo/query/acc.cgi?acc=GPL4133) |
| GSM518703 | CAD | NA | NA | NA | [GPL4133](https://www.ncbi.nlm.nih.gov/geo/query/acc.cgi?acc=GPL4133) |
| GSM518704 | CAD | NA | NA | NA | [GPL4133](https://www.ncbi.nlm.nih.gov/geo/query/acc.cgi?acc=GPL4133) |
| GSM518705 | CAD | NA | NA | NA | [GPL4133](https://www.ncbi.nlm.nih.gov/geo/query/acc.cgi?acc=GPL4133) |
| GSM518706 | CAD | NA | NA | NA | [GPL4133](https://www.ncbi.nlm.nih.gov/geo/query/acc.cgi?acc=GPL4133) |
| GSM518707 | CAD | NA | NA | NA | [GPL4133](https://www.ncbi.nlm.nih.gov/geo/query/acc.cgi?acc=GPL4133) |
| GSM518708 | CAD | NA | NA | NA | [GPL4133](https://www.ncbi.nlm.nih.gov/geo/query/acc.cgi?acc=GPL4133) |
| GSM518709 | CAD | NA | NA | NA | [GPL4133](https://www.ncbi.nlm.nih.gov/geo/query/acc.cgi?acc=GPL4133) |
| GSM518710 | CAD | NA | NA | NA | [GPL4133](https://www.ncbi.nlm.nih.gov/geo/query/acc.cgi?acc=GPL4133) |
| GSM518711 | CAD | NA | NA | NA | [GPL4133](https://www.ncbi.nlm.nih.gov/geo/query/acc.cgi?acc=GPL4133) |
| GSM518712 | CAD | NA | NA | NA | [GPL4133](https://www.ncbi.nlm.nih.gov/geo/query/acc.cgi?acc=GPL4133) |
| GSM518713 | CAD | NA | NA | NA | [GPL4133](https://www.ncbi.nlm.nih.gov/geo/query/acc.cgi?acc=GPL4133) |
| GSM518714 | CAD | NA | NA | NA | [GPL4133](https://www.ncbi.nlm.nih.gov/geo/query/acc.cgi?acc=GPL4133) |
| GSM518715 | CAD | NA | NA | NA | [GPL4133](https://www.ncbi.nlm.nih.gov/geo/query/acc.cgi?acc=GPL4133) |
| GSM518716 | CAD | NA | NA | NA | [GPL4133](https://www.ncbi.nlm.nih.gov/geo/query/acc.cgi?acc=GPL4133) |
| GSM518717 | CAD | NA | NA | NA | [GPL4133](https://www.ncbi.nlm.nih.gov/geo/query/acc.cgi?acc=GPL4133) |
| GSM518718 | CAD | NA | NA | NA | [GPL4133](https://www.ncbi.nlm.nih.gov/geo/query/acc.cgi?acc=GPL4133) |
| GSM518719 | CAD | NA | NA | NA | [GPL4133](https://www.ncbi.nlm.nih.gov/geo/query/acc.cgi?acc=GPL4133) |
| GSM518720 | CAD | NA | NA | NA | [GPL4133](https://www.ncbi.nlm.nih.gov/geo/query/acc.cgi?acc=GPL4133) |
| GSM518721 | CAD | NA | NA | NA | [GPL4133](https://www.ncbi.nlm.nih.gov/geo/query/acc.cgi?acc=GPL4133) |
| GSM518722 | CAD | NA | NA | NA | [GPL4133](https://www.ncbi.nlm.nih.gov/geo/query/acc.cgi?acc=GPL4133) |
| GSM518723 | CAD | NA | NA | NA | [GPL4133](https://www.ncbi.nlm.nih.gov/geo/query/acc.cgi?acc=GPL4133) |
| GSM518724 | CAD | NA | NA | NA | [GPL4133](https://www.ncbi.nlm.nih.gov/geo/query/acc.cgi?acc=GPL4133) |
| GSM518725 | CAD | NA | NA | NA | [GPL4133](https://www.ncbi.nlm.nih.gov/geo/query/acc.cgi?acc=GPL4133) |
| GSM518726 | CAD | NA | NA | NA | [GPL4133](https://www.ncbi.nlm.nih.gov/geo/query/acc.cgi?acc=GPL4133) |
| GSM518727 | CAD | NA | NA | NA | [GPL4133](https://www.ncbi.nlm.nih.gov/geo/query/acc.cgi?acc=GPL4133) |
| GSM518728 | CAD | NA | NA | NA | [GPL4133](https://www.ncbi.nlm.nih.gov/geo/query/acc.cgi?acc=GPL4133) |
| GSM518729 | CAD | NA | NA | NA | [GPL4133](https://www.ncbi.nlm.nih.gov/geo/query/acc.cgi?acc=GPL4133) |
| GSM518730 | CAD | NA | NA | NA | [GPL4133](https://www.ncbi.nlm.nih.gov/geo/query/acc.cgi?acc=GPL4133) |
| GSM518731 | CAD | NA | NA | NA | [GPL4133](https://www.ncbi.nlm.nih.gov/geo/query/acc.cgi?acc=GPL4133) |
| GSM518732 | CAD | NA | NA | NA | [GPL4133](https://www.ncbi.nlm.nih.gov/geo/query/acc.cgi?acc=GPL4133) |
| GSM518733 | CAD | NA | NA | NA | [GPL4133](https://www.ncbi.nlm.nih.gov/geo/query/acc.cgi?acc=GPL4133) |
| GSM518734 | CAD | NA | NA | NA | [GPL4133](https://www.ncbi.nlm.nih.gov/geo/query/acc.cgi?acc=GPL4133) |
| GSM518735 | CAD | NA | NA | NA | [GPL4133](https://www.ncbi.nlm.nih.gov/geo/query/acc.cgi?acc=GPL4133) |
| GSM518736 | CAD | NA | NA | NA | [GPL4133](https://www.ncbi.nlm.nih.gov/geo/query/acc.cgi?acc=GPL4133) |
| GSM518737 | CAD | NA | NA | NA | [GPL4133](https://www.ncbi.nlm.nih.gov/geo/query/acc.cgi?acc=GPL4133) |
| GSM518738 | CAD | NA | NA | NA | [GPL4133](https://www.ncbi.nlm.nih.gov/geo/query/acc.cgi?acc=GPL4133) |
| GSM518739 | CAD | NA | NA | NA | [GPL4133](https://www.ncbi.nlm.nih.gov/geo/query/acc.cgi?acc=GPL4133) |
| GSM518740 | CAD | NA | NA | NA | [GPL4133](https://www.ncbi.nlm.nih.gov/geo/query/acc.cgi?acc=GPL4133) |
| GSM518741 | CAD | NA | NA | NA | [GPL4133](https://www.ncbi.nlm.nih.gov/geo/query/acc.cgi?acc=GPL4133) |
| GSM518742 | CAD | NA | NA | NA | [GPL4133](https://www.ncbi.nlm.nih.gov/geo/query/acc.cgi?acc=GPL4133) |
| GSM518743 | CAD | NA | NA | NA | [GPL4133](https://www.ncbi.nlm.nih.gov/geo/query/acc.cgi?acc=GPL4133) |
| GSM518744 | CAD | NA | NA | NA | [GPL4133](https://www.ncbi.nlm.nih.gov/geo/query/acc.cgi?acc=GPL4133) |
| GSM518745 | CAD | NA | NA | NA | [GPL4133](https://www.ncbi.nlm.nih.gov/geo/query/acc.cgi?acc=GPL4133) |
| GSM518746 | CAD | NA | NA | NA | [GPL4133](https://www.ncbi.nlm.nih.gov/geo/query/acc.cgi?acc=GPL4133) |
| GSM518747 | CAD | NA | NA | NA | [GPL4133](https://www.ncbi.nlm.nih.gov/geo/query/acc.cgi?acc=GPL4133) |
| GSM518748 | CAD | NA | NA | NA | [GPL4133](https://www.ncbi.nlm.nih.gov/geo/query/acc.cgi?acc=GPL4133) |
| GSM518749 | CAD | NA | NA | NA | [GPL4133](https://www.ncbi.nlm.nih.gov/geo/query/acc.cgi?acc=GPL4133) |
| GSM518750 | CAD | NA | NA | NA | [GPL4133](https://www.ncbi.nlm.nih.gov/geo/query/acc.cgi?acc=GPL4133) |
| GSM518751 | CAD | NA | NA | NA | [GPL4133](https://www.ncbi.nlm.nih.gov/geo/query/acc.cgi?acc=GPL4133) |
| GSM518752 | CAD | NA | NA | NA | [GPL4133](https://www.ncbi.nlm.nih.gov/geo/query/acc.cgi?acc=GPL4133) |
| GSM518753 | CAD | NA | NA | NA | [GPL4133](https://www.ncbi.nlm.nih.gov/geo/query/acc.cgi?acc=GPL4133) |
| GSM518754 | CAD | NA | NA | NA | [GPL4133](https://www.ncbi.nlm.nih.gov/geo/query/acc.cgi?acc=GPL4133) |
| GSM518755 | CAD | NA | NA | NA | [GPL4133](https://www.ncbi.nlm.nih.gov/geo/query/acc.cgi?acc=GPL4133) |
| GSM518756 | CAD | NA | NA | NA | [GPL4133](https://www.ncbi.nlm.nih.gov/geo/query/acc.cgi?acc=GPL4133) |
| GSM518757 | CAD | NA | NA | NA | [GPL4133](https://www.ncbi.nlm.nih.gov/geo/query/acc.cgi?acc=GPL4133) |
| GSM518758 | CAD | NA | NA | NA | [GPL4133](https://www.ncbi.nlm.nih.gov/geo/query/acc.cgi?acc=GPL4133) |
| GSM518759 | CAD | NA | NA | NA | [GPL4133](https://www.ncbi.nlm.nih.gov/geo/query/acc.cgi?acc=GPL4133) |
| GSM518760 | CAD | NA | NA | NA | [GPL4133](https://www.ncbi.nlm.nih.gov/geo/query/acc.cgi?acc=GPL4133) |
| GSM518761 | CAD | NA | NA | NA | [GPL4133](https://www.ncbi.nlm.nih.gov/geo/query/acc.cgi?acc=GPL4133) |
| GSM518762 | CAD | NA | NA | NA | [GPL4133](https://www.ncbi.nlm.nih.gov/geo/query/acc.cgi?acc=GPL4133) |
| GSM518763 | CAD | NA | NA | NA | [GPL4133](https://www.ncbi.nlm.nih.gov/geo/query/acc.cgi?acc=GPL4133) |
| GSM518764 | CAD | NA | NA | NA | [GPL4133](https://www.ncbi.nlm.nih.gov/geo/query/acc.cgi?acc=GPL4133) |
| GSM518765 | CAD | NA | NA | NA | [GPL4133](https://www.ncbi.nlm.nih.gov/geo/query/acc.cgi?acc=GPL4133) |
| GSM518766 | CAD | NA | NA | NA | [GPL4133](https://www.ncbi.nlm.nih.gov/geo/query/acc.cgi?acc=GPL4133) |
| GSM518767 | CAD | NA | NA | NA | [GPL4133](https://www.ncbi.nlm.nih.gov/geo/query/acc.cgi?acc=GPL4133) |
| GSM518768 | CAD | NA | NA | NA | [GPL4133](https://www.ncbi.nlm.nih.gov/geo/query/acc.cgi?acc=GPL4133) |
| GSM518769 | CAD | NA | NA | NA | [GPL4133](https://www.ncbi.nlm.nih.gov/geo/query/acc.cgi?acc=GPL4133) |
| GSM518770 | CAD | NA | NA | NA | [GPL4133](https://www.ncbi.nlm.nih.gov/geo/query/acc.cgi?acc=GPL4133) |
| GSM518771 | CAD | NA | NA | NA | [GPL4133](https://www.ncbi.nlm.nih.gov/geo/query/acc.cgi?acc=GPL4133) |
| GSM518772 | CAD | NA | NA | NA | [GPL4133](https://www.ncbi.nlm.nih.gov/geo/query/acc.cgi?acc=GPL4133) |
| GSM518773 | CAD | NA | NA | NA | [GPL4133](https://www.ncbi.nlm.nih.gov/geo/query/acc.cgi?acc=GPL4133) |
| GSM518774 | CAD | NA | NA | NA | [GPL4133](https://www.ncbi.nlm.nih.gov/geo/query/acc.cgi?acc=GPL4133) |
| GSM518775 | CAD | NA | NA | NA | [GPL4133](https://www.ncbi.nlm.nih.gov/geo/query/acc.cgi?acc=GPL4133) |
| GSM518776 | CAD | NA | NA | NA | [GPL4133](https://www.ncbi.nlm.nih.gov/geo/query/acc.cgi?acc=GPL4133) |
| GSM518777 | CAD | NA | NA | NA | [GPL4133](https://www.ncbi.nlm.nih.gov/geo/query/acc.cgi?acc=GPL4133) |
| GSM518778 | CAD | NA | NA | NA | [GPL4133](https://www.ncbi.nlm.nih.gov/geo/query/acc.cgi?acc=GPL4133) |
| GSM518779 | CAD | NA | NA | NA | [GPL4133](https://www.ncbi.nlm.nih.gov/geo/query/acc.cgi?acc=GPL4133) |
| GSM518780 | CAD | NA | NA | NA | [GPL4133](https://www.ncbi.nlm.nih.gov/geo/query/acc.cgi?acc=GPL4133) |
| GSM518781 | CAD | NA | NA | NA | [GPL4133](https://www.ncbi.nlm.nih.gov/geo/query/acc.cgi?acc=GPL4133) |
| GSM518782 | CAD | NA | NA | NA | [GPL4133](https://www.ncbi.nlm.nih.gov/geo/query/acc.cgi?acc=GPL4133) |
| GSM518783 | CAD | NA | NA | NA | [GPL4133](https://www.ncbi.nlm.nih.gov/geo/query/acc.cgi?acc=GPL4133) |
| GSM518784 | CAD | NA | NA | NA | [GPL4133](https://www.ncbi.nlm.nih.gov/geo/query/acc.cgi?acc=GPL4133) |
| GSM518785 | CAD | NA | NA | NA | [GPL4133](https://www.ncbi.nlm.nih.gov/geo/query/acc.cgi?acc=GPL4133) |
| GSM518786 | CAD | NA | NA | NA | [GPL4133](https://www.ncbi.nlm.nih.gov/geo/query/acc.cgi?acc=GPL4133) |
| GSM518787 | CAD | NA | NA | NA | [GPL4133](https://www.ncbi.nlm.nih.gov/geo/query/acc.cgi?acc=GPL4133) |
| GSM518788 | CAD | NA | NA | NA | [GPL4133](https://www.ncbi.nlm.nih.gov/geo/query/acc.cgi?acc=GPL4133) |
| GSM518789 | CAD | NA | NA | NA | [GPL4133](https://www.ncbi.nlm.nih.gov/geo/query/acc.cgi?acc=GPL4133) |
| GSM518790 | CAD | NA | NA | NA | [GPL4133](https://www.ncbi.nlm.nih.gov/geo/query/acc.cgi?acc=GPL4133) |
| GSM518791 | CAD | NA | NA | NA | [GPL4133](https://www.ncbi.nlm.nih.gov/geo/query/acc.cgi?acc=GPL4133) |
| GSM518792 | CAD | NA | NA | NA | [GPL4133](https://www.ncbi.nlm.nih.gov/geo/query/acc.cgi?acc=GPL4133) |
| GSM518793 | CAD | NA | NA | NA | [GPL4133](https://www.ncbi.nlm.nih.gov/geo/query/acc.cgi?acc=GPL4133) |
| GSM518794 | CAD | NA | NA | NA | [GPL4133](https://www.ncbi.nlm.nih.gov/geo/query/acc.cgi?acc=GPL4133) |
| GSM518795 | CAD | NA | NA | NA | [GPL4133](https://www.ncbi.nlm.nih.gov/geo/query/acc.cgi?acc=GPL4133) |
| GSM518796 | CAD | NA | NA | NA | [GPL4133](https://www.ncbi.nlm.nih.gov/geo/query/acc.cgi?acc=GPL4133) |
| GSM518797 | CAD | NA | NA | NA | [GPL4133](https://www.ncbi.nlm.nih.gov/geo/query/acc.cgi?acc=GPL4133) |
| GSM518798 | CAD | NA | NA | NA | [GPL4133](https://www.ncbi.nlm.nih.gov/geo/query/acc.cgi?acc=GPL4133) |
| GSM518799 | CAD | NA | NA | NA | [GPL4133](https://www.ncbi.nlm.nih.gov/geo/query/acc.cgi?acc=GPL4133) |
| GSM518800 | CAD | NA | NA | NA | [GPL4133](https://www.ncbi.nlm.nih.gov/geo/query/acc.cgi?acc=GPL4133) |
| GSM518801 | CAD | NA | NA | NA | [GPL4133](https://www.ncbi.nlm.nih.gov/geo/query/acc.cgi?acc=GPL4133) |
| GSM518802 | CAD | NA | NA | NA | [GPL4133](https://www.ncbi.nlm.nih.gov/geo/query/acc.cgi?acc=GPL4133) |
| GSM518803 | CAD | NA | NA | NA | [GPL4133](https://www.ncbi.nlm.nih.gov/geo/query/acc.cgi?acc=GPL4133) |
| GSM518804 | CAD | NA | NA | NA | [GPL4133](https://www.ncbi.nlm.nih.gov/geo/query/acc.cgi?acc=GPL4133) |
| GSM518805 | CAD | NA | NA | NA | [GPL4133](https://www.ncbi.nlm.nih.gov/geo/query/acc.cgi?acc=GPL4133) |
| GSM518806 | CAD | NA | NA | NA | [GPL4133](https://www.ncbi.nlm.nih.gov/geo/query/acc.cgi?acc=GPL4133) |
| GSM518807 | CAD | NA | NA | NA | [GPL4133](https://www.ncbi.nlm.nih.gov/geo/query/acc.cgi?acc=GPL4133) |
| GSM518808 | CAD | NA | NA | NA | [GPL4133](https://www.ncbi.nlm.nih.gov/geo/query/acc.cgi?acc=GPL4133) |
| GSM518809 | CAD | NA | NA | NA | [GPL4133](https://www.ncbi.nlm.nih.gov/geo/query/acc.cgi?acc=GPL4133) |
| GSM518810 | CAD | NA | NA | NA | [GPL4133](https://www.ncbi.nlm.nih.gov/geo/query/acc.cgi?acc=GPL4133) |
| GSM518811 | CAD | NA | NA | NA | [GPL4133](https://www.ncbi.nlm.nih.gov/geo/query/acc.cgi?acc=GPL4133) |
| GSM518812 | CAD | NA | NA | NA | [GPL4133](https://www.ncbi.nlm.nih.gov/geo/query/acc.cgi?acc=GPL4133) |
| GSM518813 | CAD | NA | NA | NA | [GPL4133](https://www.ncbi.nlm.nih.gov/geo/query/acc.cgi?acc=GPL4133) |
| GSM518814 | CAD | NA | NA | NA | [GPL4133](https://www.ncbi.nlm.nih.gov/geo/query/acc.cgi?acc=GPL4133) |
| GSM518815 | CAD | NA | NA | NA | [GPL4133](https://www.ncbi.nlm.nih.gov/geo/query/acc.cgi?acc=GPL4133) |
| GSM518816 | CAD | NA | NA | NA | [GPL4133](https://www.ncbi.nlm.nih.gov/geo/query/acc.cgi?acc=GPL4133) |
| GSM518817 | CAD | NA | NA | NA | [GPL4133](https://www.ncbi.nlm.nih.gov/geo/query/acc.cgi?acc=GPL4133) |
| GSM518818 | CAD | NA | NA | NA | [GPL4133](https://www.ncbi.nlm.nih.gov/geo/query/acc.cgi?acc=GPL4133) |
| GSM518819 | CAD | NA | NA | NA | [GPL4133](https://www.ncbi.nlm.nih.gov/geo/query/acc.cgi?acc=GPL4133) |
| GSM518820 | CAD | NA | NA | NA | [GPL4133](https://www.ncbi.nlm.nih.gov/geo/query/acc.cgi?acc=GPL4133) |
| GSM518821 | CAD | NA | NA | NA | [GPL4133](https://www.ncbi.nlm.nih.gov/geo/query/acc.cgi?acc=GPL4133) |
| GSM518822 | CAD | NA | NA | NA | [GPL4133](https://www.ncbi.nlm.nih.gov/geo/query/acc.cgi?acc=GPL4133) |
| GSM518823 | CAD | NA | NA | NA | [GPL4133](https://www.ncbi.nlm.nih.gov/geo/query/acc.cgi?acc=GPL4133) |
| GSM518824 | CAD | NA | NA | NA | [GPL4133](https://www.ncbi.nlm.nih.gov/geo/query/acc.cgi?acc=GPL4133) |
| GSM518825 | CAD | NA | NA | NA | [GPL4133](https://www.ncbi.nlm.nih.gov/geo/query/acc.cgi?acc=GPL4133) |
| GSM518826 | CAD | NA | NA | NA | [GPL4133](https://www.ncbi.nlm.nih.gov/geo/query/acc.cgi?acc=GPL4133) |
| GSM518827 | CAD | NA | NA | NA | [GPL4133](https://www.ncbi.nlm.nih.gov/geo/query/acc.cgi?acc=GPL4133) |
| GSM518828 | CAD | NA | NA | NA | [GPL4133](https://www.ncbi.nlm.nih.gov/geo/query/acc.cgi?acc=GPL4133) |
| GSM518829 | CAD | NA | NA | NA | [GPL4133](https://www.ncbi.nlm.nih.gov/geo/query/acc.cgi?acc=GPL4133) |
| GSM518830 | CAD | NA | NA | NA | [GPL4133](https://www.ncbi.nlm.nih.gov/geo/query/acc.cgi?acc=GPL4133) |
| GSM518831 | CAD | NA | NA | NA | [GPL4133](https://www.ncbi.nlm.nih.gov/geo/query/acc.cgi?acc=GPL4133) |
| GSM518832 | CAD | NA | NA | NA | [GPL4133](https://www.ncbi.nlm.nih.gov/geo/query/acc.cgi?acc=GPL4133) |
| GSE180083 |  |  |  |  | GPL14761 |
| GSM5452345 | CAD | Male | 63 | NA | GPL6884 |
| GSM5452346 | CAD | Female | 62 | NA | GPL6884 |
| GSM5452347 | CAD | Female | 49 | NA | GPL6884 |
| GSM5452348 | CAD | Male | 68 | NA | GPL6884 |
| GSM5452349 | Normal | Female | 60 | NA | GPL6884 |
| GSM5452350 | CAD | Female | 86 | NA | GPL6884 |
| GSM5452351 | Normal | Female | 62 | NA | GPL6884 |
| GSM5452352 | Normal | Female | 56 | NA | GPL6884 |
| GSM5452353 | Normal | Female | 52 | NA | GPL6884 |
| GSM5452354 | CAD | Male | 68 | NA | GPL6884 |
| GSM5452355 | CAD | Male | 59 | NA | GPL6884 |
| GSM5452356 | Normal | Male | 70 | NA | GPL6884 |
| GSM5452357 | CAD | Female | 55 | NA | GPL6884 |
| GSM5452358 | CAD | Female | 68 | NA | GPL6884 |
| GSM5452359 | CAD | Male | 54 | NA | GPL6884 |
| GSM5452360 | CAD | Male | 57 | NA | GPL6884 |
| GSM5452361 | CAD | Male | 66 | NA | GPL6884 |
| GSM5452362 | CAD | Male | 59 | NA | GPL6884 |
| GSM5452363 | Normal | Female | 43 | NA | GPL6884 |
| GSM5452364 | CAD | Female | 58 | NA | GPL6884 |
| GSM5452365 | Normal | Male | 58 | NA | GPL6884 |
| GSM5452366 | CAD | Male | 56 | NA | GPL6884 |
| GSM5452367 | CAD | Male | 53 | NA | GPL6884 |
| GSM5452368 | CAD | Male | 72 | NA | GPL6884 |
| GSM5452369 | CAD | Male | 41 | NA | GPL6884 |
| GSM5452370 | CAD | Male | 40 | NA | GPL6884 |
| GSM5452371 | CAD | Male | 61 | NA | GPL6884 |
| GSM5452372 | CAD | Female | 54 | NA | GPL6884 |
| GSM5452373 | CAD | Female | 75 | NA | GPL6884 |
| GSM5452374 | Normal | Male | 42 | NA | GPL6884 |
| GSM5452375 | CAD | Female | 56 | NA | GPL6884 |
| GSM5452376 | CAD | Male | 52 | NA | GPL6884 |
| GSM5452377 | CAD | Male | 71 | NA | GPL6884 |
| GSM5452378 | CAD | Female | 53 | NA | GPL6884 |
| GSM5452379 | CAD | Male | 54 | NA | GPL6884 |
| GSM5452380 | CAD | Female | 63 | NA | GPL6884 |
| GSM5452381 | CAD | Male | 66 | NA | GPL6884 |
| GSM5452382 | CAD | Female | 58 | NA | GPL6884 |
| GSM5452383 | CAD | Male | 69 | NA | GPL6884 |
| GSM5452384 | CAD | Female | 53 | NA | GPL6884 |
| GSM5452385 | CAD | Female | 81 | NA | GPL6884 |
| GSM5452386 | CAD | Male | 59 | NA | GPL6884 |
| GSM5452387 | CAD | Male | 61 | NA | GPL6884 |
| GSM5452388 | CAD | Female | 73 | NA | GPL6884 |
| GSM5452389 | CAD | Male | 54 | NA | GPL6884 |
| GSM5452390 | Normal | Male | 64 | NA | GPL6884 |
| GSM5452391 | CAD | Female | 61 | NA | GPL6884 |
| GSM5452392 | CAD | Male | 63 | NA | GPL6884 |
| GSM5452393 | CAD | Female | 56 | NA | GPL6884 |
| GSM5452394 | CAD | Female | 67 | NA | GPL6884 |
| GSM5452395 | CAD | Male | 68 | NA | GPL6884 |
| GSM5452396 | CAD | Male | 61 | NA | GPL6884 |
| GSM5452397 | CAD | Male | 65 | NA | GPL6884 |
| GSM5452398 | CAD | Female | 86 | NA | GPL6884 |
| GSM5452399 | CAD | Male | 43 | NA | GPL6884 |
| GSM5452400 | CAD | Female | 47 | NA | GPL6884 |
| GSM5452401 | CAD | Male | 59 | NA | GPL6884 |
| GSM5452402 | CAD | Male | 60 | NA | GPL6884 |
| GSM5452403 | Normal | Male | 43 | NA | GPL6884 |
| GSM5452404 | CAD | Male | 69 | NA | GPL6884 |
| GSM5452405 | Normal | Female | 55 | NA | GPL6884 |
| GSM5452406 | Normal | Female | 54 | NA | GPL6884 |
| GSM5452407 | CAD | Female | 51 | NA | GPL6884 |
| GSM5452408 | Normal | Female | 47 | NA | GPL6884 |
| GSM5452409 | CAD | Female | 76 | NA | GPL6884 |
| GSM5452410 | Normal | Male | 45 | NA | GPL6884 |
| GSM5452411 | CAD | Male | 62 | NA | GPL6884 |
| GSM5452412 | CAD | Male | 61 | NA | GPL6884 |
| GSM5452413 | CAD | Female | 77 | NA | GPL6884 |
| GSM5452414 | CAD | Female | 74 | NA | GPL6884 |
| GSM5452415 | CAD | Female | 50 | NA | GPL6884 |
| GSM5452416 | CAD | Male | 61 | NA | GPL6884 |
| GSM5452417 | Normal | Female | 48 | NA | GPL6884 |
| GSM5452418 | Normal | Female | 50 | NA | GPL6884 |
| GSM5452419 | CAD | Male | 69 | NA | GPL6884 |
| GSM5452420 | CAD | Female | 48 | NA | GPL6884 |
| GSM5452421 | CAD | Male | 69 | NA | GPL6884 |
| GSM5452422 | Normal | Female | 63 | NA | GPL6884 |
| GSM5452423 | CAD | Female | 53 | NA | GPL6884 |
| GSM5452424 | Normal | Male | 48 | NA | GPL6884 |
| GSM5452425 | CAD | Male | 63 | NA | GPL6884 |
| GSM5452426 | CAD | Male | 66 | NA | GPL6884 |
| GSM5452427 | Normal | Female | 68 | NA | GPL6884 |
| GSM5452428 | CAD | Female | 70 | NA | GPL6884 |
| GSM5452429 | CAD | Female | 79 | NA | GPL6884 |
| GSM5452430 | CAD | Female | 73 | NA | GPL6884 |
| GSM5452431 | Normal | Male | 57 | NA | GPL6884 |
| GSM5452432 | CAD | Female | 74 | NA | GPL6884 |
| GSM5452433 | CAD | Female | 40 | NA | GPL6884 |
| GSM5452434 | Normal | Male | 51 | NA | GPL6884 |
| GSM5452435 | CAD | Female | 62 | NA | GPL6884 |
| GSM5452436 | Normal | Male | 48 | NA | GPL6884 |
| GSM5452437 | CAD | Male | 70 | NA | GPL6884 |
| GSM5452438 | CAD | Female | 57 | NA | GPL6884 |
| GSM5452439 | Normal | Female | 47 | NA | GPL6884 |
| GSM5452440 | CAD | Male | 71 | NA | GPL6884 |
| GSM5452441 | Normal | Male | NA | NA | GPL6884 |
| GSM5452442 | Normal | Male | NA | NA | GPL6884 |
| GSM5452443 | Normal | Female | NA | NA | GPL6884 |
| GSM5452444 | Normal | Male | NA | NA | GPL6884 |
| GSM5452445 | Normal | Male | NA | NA | GPL6884 |
| GSM5452446 | Normal | Male | NA | NA | GPL6884 |
| GSM5452447 | Normal | Male | NA | NA | GPL6884 |
| GSM5452448 | Normal | Male | NA | NA | GPL6884 |
| GSM5452449 | Normal | Female | NA | NA | GPL6884 |
| GSM5452450 | Normal | Male | NA | NA | GPL6884 |
| GSM5452451 | Normal | Female | NA | NA | GPL6884 |
| GSM5452452 | Normal | Male | NA | NA | GPL6884 |
| GSM5452453 | Normal | Male | NA | NA | GPL6884 |
| GSM5452454 | Normal | Male | NA | NA | GPL6884 |
| GSM5452455 | Normal | Female | NA | NA | GPL6884 |
| GSM5452456 | Normal | Male | NA | NA | GPL6884 |
| GSM5452457 | Normal | Male | NA | NA | GPL6884 |
| GSM5452458 | Normal | Female | NA | NA | GPL6884 |
| GSM5452459 | Normal | Female | NA | NA | GPL6884 |
| GSM5452460 | Normal | Male | NA | NA | GPL6884 |
| GSM5452461 | Normal | Male | NA | NA | GPL6884 |
| GSM5452462 | Normal | Male | NA | NA | GPL6884 |
| GSM5452463 | Normal | Female | NA | NA | GPL6884 |
| GSM5452464 | Normal | Female | NA | NA | GPL6884 |
| GSM5452465 | Normal | Female | NA | NA | GPL6884 |
| GSM5452466 | Normal | Male | NA | NA | GPL6884 |
| GSM5452467 | Normal | Male | NA | NA | GPL6884 |
| GSM5452468 | Normal | Female | NA | NA | GPL6884 |
| GSM5452469 | Normal | Female | NA | NA | GPL6884 |
| GSM5452470 | Normal | Male | NA | NA | GPL6884 |
| GSM5452471 | Normal | Female | NA | NA | GPL6884 |
| GSM5452472 | Normal | Male | NA | NA | GPL6884 |
| GSM5452473 | Normal | Female | NA | NA | GPL6884 |
| GSM5452474 | Normal | Male | NA | NA | GPL6884 |
| GSM5452475 | CAD | Male | NA | NA | GPL6884 |
| GSM5452476 | Normal | Male | NA | NA | GPL6884 |
| GSM5452477 | Normal | Male | NA | NA | GPL6884 |
| GSM5452478 | Normal | Female | NA | NA | GPL6884 |
| GSM5452479 | CAD | Female | NA | NA | GPL6884 |
| GSM5452480 | CAD | Female | NA | NA | GPL6884 |
| GSM5452481 | CAD | Female | NA | NA | GPL6884 |
| GSM5452482 | CAD | Male | NA | NA | GPL6884 |
| GSM5452483 | CAD | Female | NA | NA | GPL6884 |
| GSM5452484 | CAD | Male | NA | NA | GPL6884 |
| GSM5452485 | CAD | Male | NA | NA | GPL6884 |
| GSM5452486 | CAD | Male | NA | NA | GPL6884 |
| GSM5452487 | CAD | Male | NA | NA | GPL6884 |
| GSM5452488 | CAD | Female | NA | NA | GPL6884 |
| GSM5452489 | CAD | Male | NA | NA | GPL6884 |
| GSM5452490 | CAD | Male | NA | NA | GPL6884 |
| GSM5452491 | CAD | Male | NA | NA | GPL6884 |
| GSM5452492 | CAD | Male | NA | NA | GPL6884 |
| GSM5452493 | CAD | Male | NA | NA | GPL6884 |
| GSM5452494 | CAD | Female | NA | NA | GPL6884 |
| GSM5452495 | CAD | Female | NA | NA | GPL6884 |
| GSM5452496 | CAD | Female | NA | NA | GPL6884 |
| GSM5452497 | CAD | Male | NA | NA | GPL6884 |
| GSM5452498 | CAD | Female | NA | NA | GPL6884 |
| GSM5452499 | CAD | Male | NA | NA | GPL6884 |
| GSM5452500 | CAD | Female | NA | NA | GPL6884 |
| GSM5452501 | CAD | Female | NA | NA | GPL6884 |
| GSM5452502 | CAD | Male | NA | NA | GPL6884 |
| GSM5452503 | CAD | Female | NA | NA | GPL6884 |
| GSM5452504 | CAD | Male | NA | NA | GPL6884 |
| GSM5452505 | CAD | Female | NA | NA | GPL6884 |
| GSM5452506 | CAD | Female | NA | NA | GPL6884 |
| GSM5452507 | CAD | Female | NA | NA | GPL6884 |
| GSM5452508 | CAD | Female | NA | NA | GPL6884 |
| GSM5452509 | CAD | Male | NA | NA | GPL6884 |
| GSM5452510 | CAD | Female | NA | NA | GPL6884 |
| GSM5452511 | CAD | Female | NA | NA | GPL6884 |
| GSM5452512 | CAD | Female | NA | NA | GPL6884 |
| GSM5452513 | CAD | Male | NA | NA | GPL6884 |
| GSM5452514 | CAD | Male | NA | NA | GPL6884 |
| GSM5452515 | CAD | Male | NA | NA | GPL6884 |
| GSM5452516 | CAD | Female | NA | NA | GPL6884 |
| GSM5452517 | CAD | Male | NA | NA | GPL6884 |
| GSM5452518 | CAD | Male | NA | NA | GPL6884 |
| GSM5452519 | CAD | Male | NA | NA | GPL6884 |
| GSM5452520 | CAD | Female | NA | NA | GPL6884 |

CAD:Coronary artery diseases; NA:Missing value; GPL:Genetic platforms
