# Supplementary material for: Identification of molecular subtypes of coronary artery disease based on ferroptosis- and necroptosis-related genes
Source: Front Genet. 2022 Sep 20;13:870222. doi: 10.3389/fgene.2022.870222 (PMC9531137; doi:10.3389/fgene.2022.870222)
Supplement: Supplementary file 4 [file Table5.docx]

**Supplement Table5.** The abundance of immune cells in CAD samples was calculated using ssGSEA.

| Samples | Activated.B.cell | Activated.CD4.T.cell | Activated.CD8.T.cell | Activated.dendritic.cell | CD56bright.natural.killer.cell | CD56dim.natural.killer.cell | Eosinophil | Gamma.delta.T.cell | Immature..B.cell | Immature.dendritic.cell | MDSC |
| --- | --- | --- | --- | --- | --- | --- | --- | --- | --- | --- | --- |
| GSM308566_con | 0.612952577 | 0.511036799 | 0.86054307 | 0.848041035 | 0.51791757 | 0.56985675 | 0.414262608 | 0.559012197 | 0.602027276 | 0.487957152 | 0.88935978 |
| GSM308567_con | 0.659889468 | 0.510705364 | 0.832977207 | 0.842713052 | 0.540611661 | 0.655952695 | 0.491366636 | 0.601516703 | 0.636534339 | 0.512005512 | 0.915047903 |
| GSM308568_con | 0.598694741 | 0.472118601 | 0.871447918 | 0.805678765 | 0.523276048 | 0.719333792 | 0.449214781 | 0.613434845 | 0.649730479 | 0.465410965 | 0.895280337 |
| GSM308569_con | 0.583459907 | 0.550161385 | 0.859509533 | 0.838164717 | 0.531371298 | 0.609218056 | 0.472481287 | 0.602300044 | 0.624026922 | 0.518878503 | 0.923618214 |
| GSM308570_con | 0.658334782 | 0.409828606 | 0.809123235 | 0.877288185 | 0.562071498 | 0.58312492 | 0.446313719 | 0.561076179 | 0.578231873 | 0.524728849 | 0.898526454 |
| GSM308571_con | 0.545366717 | 0.483740447 | 0.849210086 | 0.891188179 | 0.507724367 | 0.630151221 | 0.468516236 | 0.550793078 | 0.553952241 | 0.511982999 | 0.906414507 |
| GSM308572_con | 0.57863127 | 0.526114402 | 0.847999312 | 0.843829126 | 0.511039506 | 0.613097833 | 0.442777123 | 0.590659338 | 0.54380314 | 0.515398461 | 0.925858981 |
| GSM308573_con | 0.541838238 | 0.455256976 | 0.816189593 | 0.845042886 | 0.558280486 | 0.59991483 | 0.480326897 | 0.595109678 | 0.529311573 | 0.498619141 | 0.917804318 |
| GSM308574_con | 0.696674177 | 0.484463535 | 0.832053665 | 0.833626757 | 0.560528013 | 0.642253679 | 0.528146882 | 0.557874264 | 0.584785368 | 0.539131991 | 0.917935872 |
| GSM308575_con | 0.491622126 | 0.439538902 | 0.805428437 | 0.858063453 | 0.578853523 | 0.586364625 | 0.467871041 | 0.567769573 | 0.517921291 | 0.509326265 | 0.892377893 |
| GSM308576_con | 0.616002392 | 0.418441308 | 0.822672959 | 0.864113686 | 0.542391248 | 0.548102337 | 0.481593005 | 0.609415712 | 0.66335686 | 0.536268289 | 0.891879915 |
| GSM308577_con | 0.557979091 | 0.512735931 | 0.861925371 | 0.873170552 | 0.510816606 | 0.619097596 | 0.484556084 | 0.563931351 | 0.532197554 | 0.5080065 | 0.918116704 |
| GSM308578_con | 0.621958225 | 0.448301857 | 0.837475445 | 0.852158708 | 0.535106706 | 0.637780281 | 0.514345116 | 0.579234113 | 0.546536808 | 0.508815166 | 0.895850266 |
| GSM308579_con | 0.580248057 | 0.509648626 | 0.861062638 | 0.855819843 | 0.528090779 | 0.595508665 | 0.430478542 | 0.607411063 | 0.567528782 | 0.493681517 | 0.915517894 |
| GSM308580_con | 0.477729114 | 0.483443012 | 0.848927645 | 0.877189446 | 0.53681796 | 0.605784663 | 0.441047261 | 0.566398963 | 0.568764166 | 0.518843396 | 0.918232717 |
| GSM308581_con | 0.358844186 | 0.460997954 | 0.861625821 | 0.872377998 | 0.572969742 | 0.607005958 | 0.478668519 | 0.589948703 | 0.646998637 | 0.544472877 | 0.932560519 |
| GSM308582_con | 0.634467254 | 0.554182261 | 0.870775306 | 0.835973442 | 0.520286389 | 0.607523499 | 0.417411666 | 0.597489124 | 0.564951708 | 0.513136452 | 0.914403298 |
| GSM308583_con | 0.664216369 | 0.542864782 | 0.837884716 | 0.849289705 | 0.513854709 | 0.634489463 | 0.475994832 | 0.624779896 | 0.562799435 | 0.487880015 | 0.908590418 |
| GSM308584_con | 0.519899546 | 0.487134506 | 0.841917671 | 0.891971908 | 0.533039807 | 0.658732722 | 0.438778361 | 0.576029964 | 0.532403732 | 0.513255685 | 0.912371462 |
| GSM308585_con | 0.448370154 | 0.437373629 | 0.853601636 | 0.869536001 | 0.528287416 | 0.654042293 | 0.405166821 | 0.59738628 | 0.492931382 | 0.523168672 | 0.926884617 |
| GSM308586_con | 0.510370906 | 0.460381495 | 0.830314523 | 0.862908605 | 0.493284022 | 0.655273045 | 0.449501347 | 0.606137213 | 0.576859091 | 0.528495249 | 0.909211339 |
| GSM308587_con | 0.581287736 | 0.514148276 | 0.855913758 | 0.860876357 | 0.511824774 | 0.61017589 | 0.450054345 | 0.615699054 | 0.606066593 | 0.517303935 | 0.935228763 |
| GSM308588_con | 0.514716162 | 0.418753154 | 0.802417508 | 0.848125305 | 0.522570727 | 0.66757128 | 0.454118771 | 0.574474173 | 0.620231457 | 0.50356073 | 0.887821356 |
| GSM308589_con | 0.572763051 | 0.501686912 | 0.834167502 | 0.849419348 | 0.561163862 | 0.57982604 | 0.457711076 | 0.603468127 | 0.527169481 | 0.525551942 | 0.917620925 |
| GSM308590_con | 0.576057433 | 0.516415299 | 0.845970723 | 0.880618346 | 0.518642328 | 0.592042088 | 0.471914652 | 0.563841346 | 0.587607008 | 0.512904528 | 0.914090135 |
| GSM308591_con | 0.588186728 | 0.508835883 | 0.840402854 | 0.852164843 | 0.507741785 | 0.662220954 | 0.489972025 | 0.568697227 | 0.626096415 | 0.501724937 | 0.908674563 |
| GSM308592_con | 0.524987466 | 0.503697476 | 0.833802193 | 0.877653423 | 0.549314778 | 0.576323737 | 0.466936359 | 0.553476226 | 0.607329624 | 0.516895121 | 0.90302298 |
| GSM308593_con | 0.405115943 | 0.45399911 | 0.779144718 | 0.862433449 | 0.537966083 | 0.574334964 | 0.500785974 | 0.587690085 | 0.598573335 | 0.548986804 | 0.914025494 |
| GSM308594_con | 0.542591682 | 0.449912546 | 0.835225852 | 0.84575579 | 0.529519898 | 0.591394649 | 0.410107863 | 0.58289697 | 0.523763196 | 0.500323994 | 0.916478709 |
| GSM308595_con | 0.589666084 | 0.484710548 | 0.823384744 | 0.876410525 | 0.533730556 | 0.603389209 | 0.481236063 | 0.546618966 | 0.559063985 | 0.53236252 | 0.913538214 |
| GSM308596_con | 0.626605388 | 0.552723849 | 0.846760974 | 0.838125588 | 0.5360807 | 0.688685076 | 0.498806973 | 0.59736219 | 0.567879973 | 0.549113387 | 0.919093185 |
| GSM308597_con | 0.674339949 | 0.512138697 | 0.849721648 | 0.839129298 | 0.529735026 | 0.57977493 | 0.44953856 | 0.628615558 | 0.651915797 | 0.508033052 | 0.89082808 |
| GSM308598_con | 0.599220049 | 0.484614888 | 0.857867237 | 0.837821749 | 0.534652713 | 0.648719872 | 0.475883243 | 0.591440084 | 0.545025925 | 0.49910071 | 0.899124908 |
| GSM308599_con | 0.608455307 | 0.499462 | 0.838469816 | 0.851210686 | 0.553174941 | 0.674825538 | 0.33214385 | 0.543245506 | 0.536053521 | 0.498613397 | 0.926466021 |
| GSM308602_con | 0.58004255 | 0.510356848 | 0.848182438 | 0.875596492 | 0.506765075 | 0.538751121 | 0.368842212 | 0.531339574 | 0.536869883 | 0.515414934 | 0.892411586 |
| GSM308612_con | 0.654195375 | 0.502498943 | 0.824197653 | 0.845545997 | 0.559386963 | 0.560867719 | 0.451738772 | 0.603366149 | 0.521503992 | 0.498688383 | 0.898328947 |
| GSM308619_con | 0.581050545 | 0.506547519 | 0.855607231 | 0.854715241 | 0.551844024 | 0.618780997 | 0.458910369 | 0.581347277 | 0.538726924 | 0.518557686 | 0.907825451 |
| GSM308621_con | 0.547396383 | 0.484468689 | 0.846523578 | 0.848717994 | 0.516284017 | 0.610882129 | 0.41893036 | 0.583449331 | 0.590262886 | 0.489587547 | 0.925599086 |
| GSM308624_con | 0.512514292 | 0.431787628 | 0.818493449 | 0.850436194 | 0.537920934 | 0.656619299 | 0.404277584 | 0.562002766 | 0.541321675 | 0.490311798 | 0.919030645 |
| GSM308628_con | 0.56116407 | 0.524306923 | 0.869275125 | 0.877597515 | 0.547960341 | 0.614580391 | 0.46146139 | 0.566979703 | 0.607194562 | 0.492086866 | 0.913182304 |
| GSM308633_con | 0.467241831 | 0.522616845 | 0.859478032 | 0.867473143 | 0.557625971 | 0.610896449 | 0.413359762 | 0.601736941 | 0.570074024 | 0.532240142 | 0.870315564 |
| GSM308635_con | 0.584999694 | 0.506580155 | 0.85710777 | 0.865465544 | 0.516886035 | 0.573245823 | 0.40032529 | 0.592714599 | 0.569349121 | 0.515060504 | 0.903365168 |
| GSM308636_con | 0.536919709 | 0.515410949 | 0.831182929 | 0.857039058 | 0.555161065 | 0.666559681 | 0.426979494 | 0.586538245 | 0.559049668 | 0.519951295 | 0.908755561 |
| GSM308638_con | 0.492350573 | 0.485334414 | 0.78718528 | 0.861899976 | 0.508792458 | 0.597821284 | 0.423256272 | 0.59391152 | 0.539302529 | 0.489354207 | 0.928741229 |
| GSM308643_con | 0.538770756 | 0.522650349 | 0.807993866 | 0.870735719 | 0.541016945 | 0.581701708 | 0.438760416 | 0.586368403 | 0.611969393 | 0.507313385 | 0.910555983 |
| GSM308648_con | 0.480394476 | 0.461116856 | 0.848301837 | 0.889550625 | 0.530070729 | 0.681607144 | 0.459557117 | 0.598231905 | 0.561229059 | 0.497592951 | 0.918726148 |
| GSM308649_con | 0.489098398 | 0.525337639 | 0.865451735 | 0.856209108 | 0.53561265 | 0.629278728 | 0.467045413 | 0.603131355 | 0.606029883 | 0.514253055 | 0.930256431 |
| GSM308651_con | 0.551732142 | 0.560581206 | 0.854492639 | 0.853328947 | 0.50739423 | 0.599306083 | 0.448983026 | 0.59142675 | 0.587195512 | 0.48897014 | 0.902709785 |
| GSM308654_con | 0.608424282 | 0.506949766 | 0.827736065 | 0.860905611 | 0.552178482 | 0.59338289 | 0.43942234 | 0.61216258 | 0.644625487 | 0.515600972 | 0.90224701 |
| GSM308656_con | 0.652749365 | 0.519739161 | 0.864826658 | 0.866412534 | 0.55539204 | 0.582816851 | 0.398806229 | 0.597643099 | 0.556698422 | 0.486443873 | 0.909723543 |
| GSM308660_con | 0.63264043 | 0.519241486 | 0.851582184 | 0.84983122 | 0.540006321 | 0.64372332 | 0.522534803 | 0.581833825 | 0.62735878 | 0.496368165 | 0.917584189 |
| GSM308661_con | 0.642797456 | 0.493306899 | 0.851519728 | 0.85534369 | 0.506554237 | 0.618455494 | 0.42929261 | 0.556146901 | 0.561515878 | 0.521640516 | 0.901034877 |
| GSM308662_con | 0.604518648 | 0.480486134 | 0.866824948 | 0.82758278 | 0.517304954 | 0.59325715 | 0.372042755 | 0.518552696 | 0.649260163 | 0.523540785 | 0.904185028 |
| GSM308663_con | 0.5032466 | 0.492085544 | 0.829355645 | 0.865591619 | 0.536720142 | 0.577122289 | 0.51656224 | 0.63391133 | 0.540804314 | 0.498538108 | 0.91033746 |
| GSM308665_con | 0.478005968 | 0.483895308 | 0.863289409 | 0.859118447 | 0.547153803 | 0.616186914 | 0.499111018 | 0.601505087 | 0.536821048 | 0.54351253 | 0.930326914 |
| GSM308677_con | 0.607540606 | 0.539053756 | 0.858254578 | 0.864258622 | 0.526777608 | 0.576237823 | 0.436592618 | 0.584670615 | 0.634337034 | 0.506010035 | 0.915970556 |
| GSM308680_con | 0.561430131 | 0.466602347 | 0.835127035 | 0.835977894 | 0.499430042 | 0.62470456 | 0.470229599 | 0.573778744 | 0.503693874 | 0.513739151 | 0.933519153 |
| GSM308682_con | 0.52462524 | 0.460711786 | 0.757963775 | 0.872125779 | 0.518473562 | 0.57294804 | 0.462482464 | 0.56975157 | 0.531757991 | 0.557142774 | 0.907336709 |
| GSM308683_con | 0.652243994 | 0.523562916 | 0.884271976 | 0.850163598 | 0.517195396 | 0.640311129 | 0.427561841 | 0.59924191 | 0.556132846 | 0.539016251 | 0.913325096 |
| GSM308684_con | 0.515926173 | 0.453475914 | 0.84861697 | 0.840281303 | 0.491462583 | 0.61931843 | 0.408740591 | 0.487434509 | 0.600722737 | 0.556632659 | 0.904607399 |
| GSM308687_con | 0.588039618 | 0.427514364 | 0.823240935 | 0.853184955 | 0.533195909 | 0.623165557 | 0.480696224 | 0.580028287 | 0.583506753 | 0.520007161 | 0.924970995 |
| GSM308698_con | 0.489504778 | 0.465939659 | 0.790090465 | 0.841632519 | 0.549177789 | 0.658652985 | 0.389510404 | 0.5790409 | 0.524728993 | 0.4789117 | 0.936531902 |
| GSM308701_con | 0.602364006 | 0.53506381 | 0.863075202 | 0.841547422 | 0.527006167 | 0.554410695 | 0.452693781 | 0.577116544 | 0.606566114 | 0.51684572 | 0.920848494 |
| GSM308702_con | 0.659874985 | 0.462562171 | 0.859195229 | 0.880526805 | 0.524582271 | 0.564270972 | 0.449097576 | 0.531797245 | 0.644056246 | 0.511140882 | 0.915216481 |
| GSM308706_con | 0.613934025 | 0.542445786 | 0.875327607 | 0.856667925 | 0.571894843 | 0.63266888 | 0.440218444 | 0.588605957 | 0.595323035 | 0.485029544 | 0.916147565 |
| GSM308709_con | 0.424873055 | 0.469962959 | 0.779861843 | 0.871516301 | 0.515695828 | 0.665998255 | 0.431613915 | 0.61707487 | 0.642596332 | 0.48883462 | 0.929860361 |
| GSM308712_con | 0.623231576 | 0.56917525 | 0.893427233 | 0.839681649 | 0.518377834 | 0.571834756 | 0.475668865 | 0.590574069 | 0.592133219 | 0.536683439 | 0.926310782 |
| GSM308713_con | 0.492806545 | 0.530029964 | 0.836872387 | 0.855260446 | 0.535197821 | 0.652429069 | 0.491426823 | 0.589252058 | 0.513649449 | 0.472443949 | 0.904800344 |
| GSM308714_con | 0.541346668 | 0.502906726 | 0.827398092 | 0.870645905 | 0.540744008 | 0.621663577 | 0.36090121 | 0.56212979 | 0.545797989 | 0.52109983 | 0.894167172 |
| GSM308716_con | 0.705858972 | 0.50031003 | 0.871539663 | 0.848098256 | 0.509411728 | 0.658167923 | 0.447014348 | 0.613194078 | 0.57904519 | 0.531669046 | 0.913552233 |
| GSM308720_con | 0.580138415 | 0.475722468 | 0.831451483 | 0.840895527 | 0.546856197 | 0.5486286 | 0.505371682 | 0.6159957 | 0.578226079 | 0.536093959 | 0.906488841 |
| GSM308721_con | 0.582483182 | 0.476185712 | 0.827783703 | 0.854082228 | 0.538638927 | 0.614835969 | 0.464253365 | 0.5907967 | 0.589911976 | 0.516941393 | 0.925929255 |
| GSM308722_con | 0.615404141 | 0.510126908 | 0.880693455 | 0.860865975 | 0.532169721 | 0.644135415 | 0.507768361 | 0.564033955 | 0.570175806 | 0.51536582 | 0.89013607 |
| GSM308726_con | 0.519714081 | 0.554463465 | 0.882488105 | 0.85670381 | 0.511611163 | 0.700445864 | 0.496808082 | 0.61725653 | 0.644467131 | 0.51510672 | 0.92473033 |
| GSM308728_con | 0.641485018 | 0.541168385 | 0.895877756 | 0.835128264 | 0.491540803 | 0.677762801 | 0.445606969 | 0.598154073 | 0.608435302 | 0.4826478 | 0.892488858 |
| GSM308729_con | 0.569363877 | 0.567108743 | 0.891459319 | 0.822304059 | 0.538575629 | 0.654276546 | 0.460798651 | 0.605816565 | 0.6062635 | 0.509301129 | 0.907783843 |
| GSM308730_con | 0.513903257 | 0.436021709 | 0.814498012 | 0.845592928 | 0.541794477 | 0.648629457 | 0.458403312 | 0.572869215 | 0.569489747 | 0.521399753 | 0.927998776 |
| GSM308731_con | 0.633374624 | 0.504918828 | 0.86760827 | 0.842332568 | 0.549575427 | 0.57951825 | 0.442332639 | 0.601817345 | 0.633350622 | 0.520387843 | 0.927063809 |
| GSM308732_con | 0.514668274 | 0.514707395 | 0.860942289 | 0.867067335 | 0.583149058 | 0.633595905 | 0.431049526 | 0.559242697 | 0.518602262 | 0.539696168 | 0.892554093 |
| GSM308733_con | 0.591307043 | 0.497985849 | 0.843203703 | 0.850600987 | 0.568213584 | 0.622749272 | 0.445812656 | 0.555170857 | 0.626020768 | 0.517211246 | 0.911422779 |
| GSM308735_con | 0.629720071 | 0.471257048 | 0.82304999 | 0.846116912 | 0.528229012 | 0.579937005 | 0.467020805 | 0.596651184 | 0.58641645 | 0.514530869 | 0.926540715 |
| GSM308738_con | 0.553688739 | 0.496736814 | 0.826092848 | 0.87772106 | 0.560508415 | 0.629502917 | 0.478896245 | 0.557462848 | 0.536841405 | 0.51162553 | 0.914043375 |
| GSM308739_con | 0.556933786 | 0.514575188 | 0.836025524 | 0.844092165 | 0.550800073 | 0.591142109 | 0.465633423 | 0.61267327 | 0.599902685 | 0.515113463 | 0.920685371 |
| GSM308740_con | 0.506276984 | 0.498648086 | 0.843229267 | 0.868757942 | 0.522495736 | 0.587009971 | 0.490356639 | 0.554132956 | 0.545342437 | 0.54773215 | 0.911692029 |
| GSM308744_con | 0.575985527 | 0.498883455 | 0.807793963 | 0.890371045 | 0.565679312 | 0.613205443 | 0.435613758 | 0.581068402 | 0.564190397 | 0.533621621 | 0.92408271 |
| GSM308746_con | 0.572917358 | 0.513482251 | 0.838746747 | 0.827151996 | 0.490360957 | 0.564980003 | 0.448803863 | 0.570314742 | 0.559200609 | 0.498830812 | 0.921982108 |
| GSM308748_con | 0.589139185 | 0.510743135 | 0.862485907 | 0.866619286 | 0.52965347 | 0.629729021 | 0.404336913 | 0.550786164 | 0.612934329 | 0.496365002 | 0.919428408 |
| GSM308749_con | 0.395066468 | 0.555245437 | 0.843259405 | 0.889638057 | 0.538098287 | 0.587573296 | 0.44954919 | 0.562725446 | 0.612674513 | 0.522905086 | 0.926786482 |
| GSM308750_con | 0.606782499 | 0.516252291 | 0.877049365 | 0.841618304 | 0.533324029 | 0.6553503 | 0.432622777 | 0.623702824 | 0.617696338 | 0.543614421 | 0.902823354 |
| GSM308751_con | 0.56555655 | 0.422445754 | 0.856316202 | 0.852114136 | 0.508139278 | 0.6380305 | 0.407257598 | 0.560718878 | 0.525019998 | 0.518226342 | 0.921643788 |
| GSM308753_con | 0.524633245 | 0.505944822 | 0.836459459 | 0.8238023 | 0.579558135 | 0.596782594 | 0.461446108 | 0.588218483 | 0.5519642 | 0.514587073 | 0.921794046 |
| GSM308757_con | 0.390279332 | 0.513434835 | 0.838207652 | 0.863802283 | 0.529333054 | 0.618551299 | 0.457878797 | 0.573737628 | 0.514940935 | 0.502637698 | 0.897842211 |
| GSM308758_con | 0.527762524 | 0.517803364 | 0.834117736 | 0.858475404 | 0.554461201 | 0.576025776 | 0.546666142 | 0.627687606 | 0.617945398 | 0.524026109 | 0.921854971 |
| GSM308760_con | 0.543346377 | 0.45035847 | 0.835599378 | 0.87887054 | 0.540465449 | 0.621590147 | 0.449292091 | 0.545572202 | 0.646673862 | 0.519824465 | 0.921642498 |
| GSM308761_con | 0.406543281 | 0.485745555 | 0.835858697 | 0.847878632 | 0.539637343 | 0.604598406 | 0.473150012 | 0.553833452 | 0.515868779 | 0.522727297 | 0.899326022 |
| GSM308762_con | 0.427842177 | 0.461431157 | 0.788776055 | 0.871477192 | 0.530155228 | 0.574882269 | 0.475382396 | 0.598897357 | 0.569004717 | 0.489868676 | 0.910753441 |
| GSM308763_con | 0.539760848 | 0.526767494 | 0.764845221 | 0.82972305 | 0.551173657 | 0.604346287 | 0.486652639 | 0.585089035 | 0.604683529 | 0.5124956 | 0.904405241 |
| GSM308764_con | 0.584045753 | 0.476503293 | 0.823117529 | 0.868941677 | 0.490450022 | 0.608081661 | 0.434881869 | 0.530523935 | 0.601791301 | 0.486832736 | 0.924807562 |
| GSM308765_con | 0.595626761 | 0.552400953 | 0.883528351 | 0.862109325 | 0.519843166 | 0.575866552 | 0.443292263 | 0.601149246 | 0.659908311 | 0.531864382 | 0.92230181 |
| GSM308766_con | 0.449489229 | 0.451819532 | 0.838754388 | 0.887314458 | 0.538814127 | 0.632966523 | 0.424431926 | 0.595693649 | 0.509010811 | 0.516307571 | 0.924152261 |
| GSM308767_con | 0.59744222 | 0.489227918 | 0.828793821 | 0.868892828 | 0.533007614 | 0.596860263 | 0.436720711 | 0.600635396 | 0.510365891 | 0.540697879 | 0.909162018 |
| GSM308768_con | 0.579113422 | 0.482533694 | 0.845523586 | 0.890990041 | 0.546390563 | 0.629040359 | 0.477151443 | 0.539331734 | 0.55659436 | 0.528384942 | 0.901825399 |
| GSM308769_con | 0.581296423 | 0.483577713 | 0.854022392 | 0.884716011 | 0.543220254 | 0.608996416 | 0.436903113 | 0.586753828 | 0.571146611 | 0.516219774 | 0.881540114 |
| GSM308770_con | 0.472275527 | 0.472897826 | 0.821934589 | 0.882532166 | 0.557632855 | 0.593357038 | 0.435652492 | 0.563550531 | 0.63138357 | 0.484518369 | 0.888157009 |
| GSM308771_con | 0.482615943 | 0.447357517 | 0.79269394 | 0.883488575 | 0.492805249 | 0.63369668 | 0.413522201 | 0.581705028 | 0.514762654 | 0.518881082 | 0.886663188 |
| GSM308772_con | 0.524524688 | 0.385459032 | 0.800987584 | 0.837353051 | 0.557325485 | 0.622645786 | 0.434566767 | 0.509261571 | 0.462185474 | 0.507471374 | 0.902307284 |
| GSM308774_con | 0.537669735 | 0.536346795 | 0.867796663 | 0.850036267 | 0.530068366 | 0.609510703 | 0.435850329 | 0.596031929 | 0.627285346 | 0.516485367 | 0.915457157 |
| GSM308777_con | 0.517850541 | 0.503083481 | 0.832667697 | 0.873229377 | 0.555956465 | 0.593304336 | 0.507852845 | 0.596744421 | 0.607287229 | 0.52797878 | 0.900519225 |
| GSM308779_con | 0.479772174 | 0.44869696 | 0.822205237 | 0.882781121 | 0.571911755 | 0.574166489 | 0.393149836 | 0.589901882 | 0.587633519 | 0.502000501 | 0.928028827 |
| GSM308785_con | 0.527350741 | 0.449762692 | 0.8317117 | 0.834830585 | 0.512549529 | 0.674071945 | 0.460465464 | 0.533378576 | 0.53685173 | 0.516939895 | 0.928124793 |
| GSM308786_con | 0.601587434 | 0.505862108 | 0.828997059 | 0.876463039 | 0.513011234 | 0.595219758 | 0.414177169 | 0.583354458 | 0.613853794 | 0.507710236 | 0.925378366 |
| GSM308787_con | 0.564083808 | 0.513988794 | 0.822663819 | 0.874685606 | 0.559872978 | 0.620377038 | 0.457866883 | 0.577759374 | 0.558074171 | 0.528657848 | 0.894948977 |
| GSM518638_con | 0.583056667 | 0.52582688 | 0.851537538 | 0.858378023 | 0.530457479 | 0.631774685 | 0.438372915 | 0.532379666 | 0.567792505 | 0.506295484 | 0.913060631 |
| GSM518639_con | 0.561059328 | 0.493441056 | 0.841660564 | 0.832408252 | 0.576059607 | 0.64838597 | 0.442972369 | 0.614860702 | 0.569936559 | 0.49754687 | 0.918934579 |
| GSM518640_con | 0.496828015 | 0.430128608 | 0.786916273 | 0.888601108 | 0.549040406 | 0.60670754 | 0.521040557 | 0.591454798 | 0.555589079 | 0.507249003 | 0.897714091 |
| GSM518641_con | 0.611099903 | 0.520093097 | 0.843510805 | 0.842988948 | 0.528912589 | 0.620165952 | 0.464616077 | 0.553333928 | 0.509912075 | 0.540058981 | 0.903510847 |
| GSM518642_con | 0.419608633 | 0.462048964 | 0.849038712 | 0.855988774 | 0.536352897 | 0.600193545 | 0.480556533 | 0.58607989 | 0.614857535 | 0.536512597 | 0.928779531 |
| GSM518643_con | 0.488326737 | 0.567439417 | 0.790514182 | 0.862125051 | 0.539192209 | 0.542412611 | 0.430587711 | 0.587874794 | 0.619798593 | 0.525622991 | 0.949240716 |
| GSM518644_con | 0.624605882 | 0.552524116 | 0.864959984 | 0.835565014 | 0.566145063 | 0.598111714 | 0.482048697 | 0.590725931 | 0.573859483 | 0.51814641 | 0.915508319 |
| GSM518645_con | 0.66343171 | 0.50761967 | 0.877334435 | 0.83582101 | 0.564642377 | 0.645638224 | 0.443985806 | 0.581494622 | 0.644892863 | 0.500577989 | 0.917417079 |
| GSM518646_con | 0.568434809 | 0.474079388 | 0.82524522 | 0.853739036 | 0.554341965 | 0.652041259 | 0.434017169 | 0.56468484 | 0.581624992 | 0.478187234 | 0.908918141 |
| GSM518647_con | 0.782313562 | 0.522494504 | 0.891146754 | 0.818051742 | 0.550921639 | 0.590057747 | 0.395264641 | 0.575649435 | 0.656122414 | 0.514367848 | 0.926740942 |
| GSM518648_con | 0.581952169 | 0.471892506 | 0.861647771 | 0.873720632 | 0.562640922 | 0.611212311 | 0.461794385 | 0.58957247 | 0.609795409 | 0.501666445 | 0.904625554 |
| GSM518649_con | 0.580750611 | 0.479807788 | 0.821805489 | 0.846701203 | 0.531050293 | 0.628564822 | 0.460370531 | 0.563571268 | 0.676169744 | 0.517168782 | 0.925142179 |
| GSM518650_con | 0.629808785 | 0.513069592 | 0.834028135 | 0.834399524 | 0.516906955 | 0.595935718 | 0.46750805 | 0.558792645 | 0.57612231 | 0.501624863 | 0.91354672 |
| GSM518651_con | 0.775419158 | 0.400615875 | 0.786089678 | 0.878353278 | 0.535024695 | 0.592084841 | 0.429627831 | 0.517498498 | 0.572550933 | 0.487587961 | 0.894160205 |
| GSM518652_con | 0.598106528 | 0.472655916 | 0.83843338 | 0.853243156 | 0.49523089 | 0.667167396 | 0.433916621 | 0.575403819 | 0.489062401 | 0.465269767 | 0.898661151 |
| GSM518653_con | 0.52028545 | 0.475873634 | 0.804794784 | 0.855815916 | 0.523743419 | 0.651838382 | 0.401018749 | 0.5462078 | 0.593002603 | 0.497065018 | 0.915189155 |
| GSM518654_con | 0.498924027 | 0.507107251 | 0.823960847 | 0.889663562 | 0.511421873 | 0.569449919 | 0.454255499 | 0.558620755 | 0.542982534 | 0.473942707 | 0.891645827 |
| GSM518655_con | 0.570844282 | 0.474637116 | 0.802376691 | 0.887396846 | 0.483311694 | 0.54793215 | 0.497132102 | 0.611468361 | 0.562959805 | 0.552105347 | 0.920830928 |
| GSM518656_con | 0.61821523 | 0.509782581 | 0.833259932 | 0.898976732 | 0.536119874 | 0.676616542 | 0.447404354 | 0.561319985 | 0.583141146 | 0.514692127 | 0.900405742 |
| GSM518657_con | 0.627328566 | 0.431003915 | 0.769717742 | 0.848790183 | 0.538397973 | 0.641356543 | 0.444280422 | 0.543923866 | 0.597567255 | 0.497059571 | 0.925177177 |
| GSM518658_con | 0.4762777 | 0.488414064 | 0.837846733 | 0.857365397 | 0.527854643 | 0.655094734 | 0.425365112 | 0.583031044 | 0.548235669 | 0.534911429 | 0.90641918 |
| GSM518659_con | 0.494801462 | 0.528526743 | 0.847983644 | 0.882305388 | 0.54701147 | 0.633894704 | 0.529380283 | 0.58099508 | 0.548882723 | 0.49232604 | 0.904603847 |
| GSM518660_con | 0.729660548 | 0.488260321 | 0.847915559 | 0.85493867 | 0.566373411 | 0.621018947 | 0.443268906 | 0.580789074 | 0.62462587 | 0.497554987 | 0.907122929 |
| GSM518661_con | 0.503168651 | 0.535343539 | 0.786879587 | 0.888669218 | 0.505452424 | 0.67831421 | 0.450907536 | 0.567474784 | 0.528963874 | 0.482088446 | 0.905911188 |
| GSM518662_con | 0.517916579 | 0.389198797 | 0.738993819 | 0.901109547 | 0.517850219 | 0.60123311 | 0.458070038 | 0.559860628 | 0.561511845 | 0.495927133 | 0.891277004 |
| GSM518663_con | 0.56331468 | 0.54433432 | 0.887327292 | 0.844816007 | 0.566824384 | 0.557247081 | 0.548233422 | 0.635527128 | 0.625022816 | 0.519148745 | 0.893990469 |
| GSM518664_con | 0.683816867 | 0.538894362 | 0.876969213 | 0.810874961 | 0.521472116 | 0.592518784 | 0.43917541 | 0.549627529 | 0.553819662 | 0.531938173 | 0.903697116 |
| GSM518665_con | 0.557179411 | 0.447662648 | 0.800107262 | 0.882881395 | 0.516963914 | 0.611172436 | 0.530958158 | 0.593172975 | 0.579902611 | 0.525369859 | 0.898201561 |
| GSM518666_con | 0.582127141 | 0.454307953 | 0.790785079 | 0.845034823 | 0.561648012 | 0.683468782 | 0.454420228 | 0.621000202 | 0.57105939 | 0.532665297 | 0.930455269 |
| GSM518667_con | 0.652880821 | 0.57524339 | 0.885125398 | 0.825088705 | 0.514509868 | 0.621590648 | 0.442384449 | 0.537535718 | 0.544640935 | 0.509255886 | 0.916349224 |
| GSM518668_con | 0.553994588 | 0.506486838 | 0.82187328 | 0.85053758 | 0.525421256 | 0.682268924 | 0.447332921 | 0.553527828 | 0.500820537 | 0.484293533 | 0.913819102 |
| GSM518669_con | 0.56293281 | 0.547421966 | 0.865452543 | 0.83013653 | 0.505213088 | 0.553790662 | 0.499436639 | 0.601139376 | 0.541975792 | 0.554538548 | 0.913643678 |
| GSM518670_con | 0.576929569 | 0.532900893 | 0.827364941 | 0.834249695 | 0.511202612 | 0.530182068 | 0.54957086 | 0.581077201 | 0.565174691 | 0.531560486 | 0.909391247 |
| GSM518671_con | 0.618827273 | 0.456082288 | 0.805181912 | 0.868375073 | 0.578646099 | 0.617614606 | 0.452332765 | 0.55762117 | 0.669479451 | 0.499357693 | 0.919992535 |
| GSM518672_con | 0.583912553 | 0.442248589 | 0.772474678 | 0.856049883 | 0.550477691 | 0.624271393 | 0.476803324 | 0.565469725 | 0.633324213 | 0.487518204 | 0.912650932 |
| GSM518673_con | 0.579045256 | 0.53629931 | 0.872600214 | 0.835134143 | 0.515787153 | 0.642680081 | 0.449240301 | 0.559380847 | 0.557836255 | 0.493776654 | 0.90135535 |
| GSM518674_con | 0.662436613 | 0.564369402 | 0.870478907 | 0.825457281 | 0.544231229 | 0.633644704 | 0.399341745 | 0.570755751 | 0.586888492 | 0.472576268 | 0.929161864 |
| GSM518675_con | 0.461032186 | 0.509569417 | 0.809391436 | 0.877118559 | 0.526517539 | 0.444524926 | 0.612640438 | 0.585604905 | 0.638995352 | 0.568579317 | 0.887461136 |
| GSM518676_con | 0.54085436 | 0.385780144 | 0.750760299 | 0.915717817 | 0.545618572 | 0.689601638 | 0.377959724 | 0.591774622 | 0.604471873 | 0.488208158 | 0.934422949 |
| GSM518677_con | 0.564788683 | 0.531210061 | 0.861844368 | 0.879541152 | 0.523640347 | 0.609612528 | 0.443889936 | 0.56217958 | 0.548445131 | 0.522807355 | 0.901064701 |
| GSM518678_con | 0.602576007 | 0.491763201 | 0.854800565 | 0.843304789 | 0.528738013 | 0.620105499 | 0.453112753 | 0.577936236 | 0.574171963 | 0.540329051 | 0.953906069 |
| GSM518679_con | 0.617209592 | 0.342462982 | 0.792606257 | 0.821394278 | 0.543597786 | 0.661678853 | 0.480984404 | 0.565895294 | 0.643128837 | 0.527304124 | 0.926265307 |
| GSM518680_con | 0.654927325 | 0.537165116 | 0.887887233 | 0.830744539 | 0.542817274 | 0.630375681 | 0.419986456 | 0.587942721 | 0.65023417 | 0.529636021 | 0.936743241 |
| GSM518681_con | 0.490935109 | 0.46170184 | 0.79679011 | 0.867338481 | 0.52945952 | 0.569833958 | 0.417208984 | 0.557435883 | 0.57100547 | 0.525333814 | 0.916112704 |
| GSM518682_con | 0.669162947 | 0.558812501 | 0.896810621 | 0.828182627 | 0.53198837 | 0.670451384 | 0.391696317 | 0.552755043 | 0.53083973 | 0.50767001 | 0.922911554 |
| GSM518683_con | 0.752994558 | 0.55798451 | 0.857836348 | 0.84670636 | 0.550363717 | 0.584232947 | 0.448709544 | 0.55787072 | 0.647709085 | 0.507361573 | 0.912619697 |
| GSM518684_con | 0.628958354 | 0.498808296 | 0.871545721 | 0.833800348 | 0.56283384 | 0.664591412 | 0.458118867 | 0.568920611 | 0.618726394 | 0.501317929 | 0.910672597 |
| GSM518685_con | 0.554400144 | 0.527969261 | 0.864417418 | 0.860581262 | 0.527484909 | 0.626716951 | 0.495346306 | 0.591137758 | 0.579006914 | 0.52129311 | 0.904657966 |
| GSM518686_con | 0.548630197 | 0.549099765 | 0.852018996 | 0.883610625 | 0.482095976 | 0.64279042 | 0.417484255 | 0.576664565 | 0.527493442 | 0.492243377 | 0.914905455 |
| GSM518687_con | 0.526391731 | 0.485112766 | 0.831547675 | 0.834813404 | 0.522809847 | 0.641678076 | 0.399938427 | 0.536157716 | 0.532694272 | 0.475033347 | 0.92237093 |
| GSM518688_con | 0.594022788 | 0.456996588 | 0.840422173 | 0.845221964 | 0.556924458 | 0.646685925 | 0.467994012 | 0.595224399 | 0.565483439 | 0.506990208 | 0.905208571 |
| GSM518689_con | 0.436565546 | 0.374581307 | 0.736985221 | 0.891588037 | 0.53665892 | 0.552965791 | 0.485613652 | 0.586397025 | 0.631362382 | 0.543864794 | 0.921648332 |
| GSM518886_con | 0.672841332 | 0.504377376 | 0.825739866 | 0.848425209 | 0.528112247 | 0.65454075 | 0.434024296 | 0.555919071 | 0.560798778 | 0.488605621 | 0.90507513 |
| GSM518888_con | 0.523089677 | 0.522150726 | 0.88804763 | 0.82369135 | 0.566153062 | 0.684544621 | 0.420571109 | 0.580524598 | 0.55309665 | 0.482880079 | 0.897721931 |
| GSM518890_con | 0.598129721 | 0.549047563 | 0.875384784 | 0.843971519 | 0.489692329 | 0.621794508 | 0.419388109 | 0.55767413 | 0.630303429 | 0.508524303 | 0.926492952 |
| GSM518892_con | 0.560525095 | 0.404541093 | 0.788625569 | 0.855372008 | 0.569696077 | 0.592183074 | 0.549564842 | 0.567072362 | 0.559788482 | 0.526323214 | 0.902659541 |
| GSM518894_con | 0.629418985 | 0.567540767 | 0.895158119 | 0.838079685 | 0.56514031 | 0.651992104 | 0.522137717 | 0.560236469 | 0.575004006 | 0.500388558 | 0.91139894 |
| GSM518896_con | 0.597985072 | 0.502024376 | 0.847870031 | 0.842406579 | 0.544352403 | 0.617915806 | 0.489534264 | 0.597736953 | 0.542341036 | 0.502317983 | 0.889254925 |
| GSM518898_con | 0.446135715 | 0.520987742 | 0.880122312 | 0.843528304 | 0.551222753 | 0.599977824 | 0.48714458 | 0.616940146 | 0.557469821 | 0.504617761 | 0.900782293 |
| GSM518900_con | 0.583174352 | 0.461912005 | 0.853585954 | 0.842552024 | 0.574636489 | 0.614099828 | 0.505694706 | 0.588237865 | 0.603983698 | 0.535287002 | 0.935120262 |
| GSM518902_con | 0.494835625 | 0.435007148 | 0.78292728 | 0.855293594 | 0.566394845 | 0.573813609 | 0.537254041 | 0.596176193 | 0.569069856 | 0.52580627 | 0.91070361 |
| GSM518904_con | 0.640270601 | 0.477957278 | 0.807105279 | 0.862142429 | 0.540530205 | 0.579879946 | 0.481546581 | 0.563677297 | 0.558683144 | 0.521510125 | 0.907802339 |
| GSM518906_con | 0.654617529 | 0.557091425 | 0.881485145 | 0.818944386 | 0.562452349 | 0.565423702 | 0.484241739 | 0.592156055 | 0.565987769 | 0.504735859 | 0.898508343 |
| GSM518908_con | 0.580096699 | 0.466633433 | 0.838042314 | 0.851010818 | 0.531334412 | 0.631977656 | 0.468869417 | 0.571670671 | 0.561044186 | 0.494439434 | 0.907789801 |
| GSM518910_con | 0.604856805 | 0.492070674 | 0.805684821 | 0.848278051 | 0.545014931 | 0.642970087 | 0.44605968 | 0.574925886 | 0.648876021 | 0.483872308 | 0.906227359 |
| GSM518912_con | 0.520097609 | 0.509751749 | 0.858936676 | 0.830658848 | 0.54452424 | 0.636744038 | 0.477719675 | 0.554623987 | 0.566919411 | 0.518329099 | 0.925980127 |
| GSM518914_con | 0.593291029 | 0.484178115 | 0.858044866 | 0.850949754 | 0.524796386 | 0.626963693 | 0.485021937 | 0.587434691 | 0.563107121 | 0.502947998 | 0.920027502 |
| GSM518916_con | 0.535092415 | 0.47305846 | 0.874661361 | 0.849153317 | 0.557797593 | 0.640367662 | 0.439803635 | 0.603009387 | 0.654332155 | 0.506156162 | 0.935923123 |
| GSM518918_con | 0.575780259 | 0.523334064 | 0.85863759 | 0.881923908 | 0.56175219 | 0.617806523 | 0.488451473 | 0.572633044 | 0.551720841 | 0.506648377 | 0.910766826 |
| GSM518920_con | 0.552209251 | 0.447143234 | 0.788803878 | 0.846853894 | 0.498705941 | 0.655907911 | 0.462237353 | 0.562456509 | 0.506252615 | 0.5120356 | 0.896449514 |
| GSM518922_con | 0.595571691 | 0.481667446 | 0.830840288 | 0.835852025 | 0.542316483 | 0.634680278 | 0.480271294 | 0.55952689 | 0.570760103 | 0.508098501 | 0.89245302 |
| GSM518924_con | 0.607439686 | 0.563992371 | 0.884545287 | 0.827777015 | 0.544476619 | 0.571247032 | 0.424161919 | 0.606743309 | 0.602981274 | 0.48366224 | 0.87729185 |
| GSM518926_con | 0.731599959 | 0.550912527 | 0.888073316 | 0.80571821 | 0.54798825 | 0.641063544 | 0.401928763 | 0.614612662 | 0.665124509 | 0.482531117 | 0.895846793 |
| GSM518928_con | 0.482784904 | 0.489058579 | 0.862673266 | 0.834870852 | 0.591139884 | 0.625298257 | 0.515366103 | 0.622282026 | 0.574684054 | 0.517284767 | 0.881211628 |
| GSM518930_con | 0.459150093 | 0.409828353 | 0.795765799 | 0.858114174 | 0.539459215 | 0.664421784 | 0.515178478 | 0.581000859 | 0.550107172 | 0.520400587 | 0.930373518 |
| GSM518932_con | 0.53183244 | 0.479050778 | 0.823928292 | 0.847659853 | 0.584102422 | 0.625267977 | 0.484052584 | 0.60247051 | 0.508625005 | 0.494201472 | 0.884697019 |
| GSM518934_con | 0.483486961 | 0.487342601 | 0.855120463 | 0.850903829 | 0.538037889 | 0.564251249 | 0.413413631 | 0.619593996 | 0.542865574 | 0.529999351 | 0.925519456 |
| GSM518936_con | 0.59074089 | 0.476003684 | 0.811335698 | 0.850451952 | 0.541962382 | 0.581741086 | 0.444755678 | 0.591000205 | 0.603996349 | 0.546670224 | 0.913977607 |
| GSM518938_con | 0.535936387 | 0.411325803 | 0.800815427 | 0.86449834 | 0.540581775 | 0.593404489 | 0.456204308 | 0.531677681 | 0.616516088 | 0.460110558 | 0.904704874 |
| GSM518940_con | 0.571463999 | 0.496048464 | 0.844393017 | 0.840144663 | 0.527258962 | 0.589419325 | 0.401666085 | 0.556409022 | 0.575883258 | 0.502870278 | 0.919266372 |
| GSM518942_con | 0.55363784 | 0.429804366 | 0.850926073 | 0.848201906 | 0.536516628 | 0.568399849 | 0.414596184 | 0.592215402 | 0.571319201 | 0.522660884 | 0.94563677 |
| GSM518944_con | 0.524641763 | 0.485650576 | 0.825288791 | 0.885879675 | 0.520854475 | 0.55127575 | 0.43151156 | 0.585459437 | 0.524326803 | 0.530260021 | 0.914657593 |
| GSM518946_con | 0.496103158 | 0.526926039 | 0.811198912 | 0.847701141 | 0.51329669 | 0.664820152 | 0.425860559 | 0.607679688 | 0.539954088 | 0.506050448 | 0.935213041 |
| GSM518948_con | 0.545732837 | 0.425320473 | 0.818725509 | 0.877641832 | 0.564711649 | 0.56470259 | 0.446346185 | 0.617302382 | 0.60669304 | 0.50831494 | 0.92915922 |
| GSM518950_con | 0.45474497 | 0.624393435 | 0.847964217 | 0.844173521 | 0.532429663 | 0.608283029 | 0.404407922 | 0.584357064 | 0.521690394 | 0.500335929 | 0.886948595 |
| GSM518952_con | 0.433442501 | 0.459545315 | 0.774434883 | 0.889171089 | 0.521254473 | 0.63642226 | 0.435151498 | 0.545686843 | 0.489759797 | 0.492825549 | 0.901001642 |
| GSM518954_con | 0.566437972 | 0.526016581 | 0.864685592 | 0.872148003 | 0.540126523 | 0.522457967 | 0.485559464 | 0.634506189 | 0.540955843 | 0.548280295 | 0.918954494 |
| GSM518956_con | 0.61771613 | 0.562157661 | 0.886384592 | 0.831188488 | 0.513558432 | 0.604057135 | 0.411102258 | 0.602151687 | 0.602131186 | 0.517426419 | 0.900225899 |
| GSM518958_con | 0.576943637 | 0.565979076 | 0.878691263 | 0.818197204 | 0.520799363 | 0.655995691 | 0.39702906 | 0.5822358 | 0.566303656 | 0.500494616 | 0.928343746 |
| GSM518960_con | 0.703253036 | 0.540173026 | 0.835300514 | 0.887185727 | 0.555238507 | 0.567739843 | 0.429069141 | 0.593317684 | 0.67416485 | 0.510030918 | 0.912642552 |
| GSM518962_con | 0.595474359 | 0.486649389 | 0.840051305 | 0.855024938 | 0.520111772 | 0.557520321 | 0.415276719 | 0.59980445 | 0.601438795 | 0.567043615 | 0.924189148 |
| GSM518964_con | 0.529347321 | 0.485828884 | 0.79955627 | 0.908219873 | 0.52615465 | 0.582521309 | 0.482158281 | 0.584104629 | 0.547297223 | 0.557587092 | 0.923496457 |
| GSM518966_con | 0.553453848 | 0.529919538 | 0.879700516 | 0.842518371 | 0.544166538 | 0.547181107 | 0.464302653 | 0.601849027 | 0.658345887 | 0.542230902 | 0.909799061 |
| GSM518968_con | 0.643271281 | 0.519934326 | 0.856640282 | 0.876251773 | 0.525892198 | 0.510287665 | 0.412005302 | 0.620158656 | 0.618645288 | 0.557173646 | 0.920777078 |
| GSM518970_con | 0.57586265 | 0.50110885 | 0.807310843 | 0.880828486 | 0.54836189 | 0.574549926 | 0.467906922 | 0.581222126 | 0.56980976 | 0.580340822 | 0.920069539 |
| GSM518972_con | 0.566226204 | 0.569686951 | 0.875150416 | 0.861473704 | 0.569833796 | 0.551187759 | 0.487865631 | 0.638289683 | 0.602109698 | 0.549521423 | 0.915344743 |
| GSM518974_con | 0.598316734 | 0.578861452 | 0.878386548 | 0.816065127 | 0.50492899 | 0.65905231 | 0.371305966 | 0.584284372 | 0.592187619 | 0.495703958 | 0.906664591 |
| GSM518976_con | 0.742648726 | 0.519067966 | 0.857219914 | 0.83500043 | 0.554554843 | 0.530387253 | 0.530507218 | 0.598249706 | 0.669603037 | 0.52816674 | 0.931784103 |
| GSM518978_con | 0.597897104 | 0.486382827 | 0.796644206 | 0.872682259 | 0.485244939 | 0.635052437 | 0.437240295 | 0.561996522 | 0.561439136 | 0.516186764 | 0.901719054 |
| GSM518980_con | 0.446184671 | 0.514442302 | 0.863594672 | 0.838687621 | 0.548830803 | 0.638452148 | 0.372986537 | 0.578368249 | 0.519082051 | 0.498884978 | 0.909903904 |
| GSM518982_con | 0.466025159 | 0.425092492 | 0.754003055 | 0.864624103 | 0.512815716 | 0.616801705 | 0.454097205 | 0.556823519 | 0.526342973 | 0.501463495 | 0.892887658 |
| GSM518984_con | 0.526115726 | 0.517259127 | 0.834007324 | 0.857430217 | 0.521033276 | 0.600021579 | 0.422825734 | 0.536257277 | 0.526047605 | 0.509874655 | 0.905302094 |
| GSM518986_con | 0.668378448 | 0.447371497 | 0.831666038 | 0.891070298 | 0.569794015 | 0.579576674 | 0.497599994 | 0.592154911 | 0.643618817 | 0.533231477 | 0.922649036 |
| GSM518988_con | 0.4823984 | 0.511354678 | 0.875660961 | 0.86476803 | 0.516916806 | 0.62026223 | 0.466336315 | 0.526902417 | 0.546443756 | 0.512683121 | 0.904341291 |
| GSM518990_con | 0.527059919 | 0.577533865 | 0.884637996 | 0.830574098 | 0.586261175 | 0.627375615 | 0.447558333 | 0.582589718 | 0.554115182 | 0.494733939 | 0.891635911 |
| GSM518992_con | 0.62092681 | 0.498189344 | 0.854852071 | 0.828806834 | 0.536624555 | 0.598877579 | 0.539083471 | 0.570504696 | 0.667494395 | 0.559569631 | 0.919896418 |
| GSM518994_con | 0.396282975 | 0.421798086 | 0.815379912 | 0.853536714 | 0.533156383 | 0.619240631 | 0.450200697 | 0.554926158 | 0.564675481 | 0.495773493 | 0.884563017 |
| GSM518996_con | 0.598683211 | 0.508488888 | 0.836783803 | 0.862522434 | 0.525924479 | 0.596138452 | 0.485529289 | 0.602698983 | 0.579202068 | 0.518233319 | 0.920845444 |
| GSM518998_con | 0.498873819 | 0.487120279 | 0.831786656 | 0.856497034 | 0.534269008 | 0.58609409 | 0.515743194 | 0.577503337 | 0.545981925 | 0.521692647 | 0.946711544 |
| GSM519000_con | 0.602799758 | 0.468827451 | 0.826611557 | 0.859624239 | 0.521559513 | 0.653614475 | 0.464012951 | 0.589796638 | 0.684782114 | 0.493059692 | 0.913009364 |
| GSM519002_con | 0.634735859 | 0.486065079 | 0.844520204 | 0.843097061 | 0.543131969 | 0.60088064 | 0.510867557 | 0.5681193 | 0.619098833 | 0.550017381 | 0.948456033 |
| GSM519004_con | 0.476798451 | 0.432965148 | 0.7775091 | 0.854857052 | 0.530032426 | 0.621095095 | 0.466670457 | 0.560058571 | 0.599337924 | 0.469375 | 0.905364943 |
| GSM519006_con | 0.708478257 | 0.569972147 | 0.872475504 | 0.821121245 | 0.544076848 | 0.650583065 | 0.423288203 | 0.567531513 | 0.650581362 | 0.500143778 | 0.916162931 |
| GSM519008_con | 0.534479516 | 0.427825414 | 0.803070937 | 0.866093764 | 0.521053763 | 0.612388308 | 0.494886398 | 0.575820933 | 0.559024416 | 0.513083132 | 0.933173365 |
| GSM519010_con | 0.660224288 | 0.505529476 | 0.821589084 | 0.851993856 | 0.55927246 | 0.645736938 | 0.485644714 | 0.588250814 | 0.647366643 | 0.52255582 | 0.928005408 |
| GSM519012_con | 0.64686206 | 0.474161166 | 0.782579658 | 0.856472774 | 0.505222553 | 0.63009939 | 0.481387561 | 0.564007328 | 0.70613541 | 0.552906176 | 0.946914539 |
| GSM519014_con | 0.513135519 | 0.501165198 | 0.859181286 | 0.850820036 | 0.536258187 | 0.642705072 | 0.446605501 | 0.577836965 | 0.58684543 | 0.519477018 | 0.90251249 |
| GSM519016_con | 0.45405169 | 0.524954494 | 0.85915463 | 0.881049343 | 0.535107932 | 0.60217658 | 0.449188192 | 0.594064976 | 0.513672428 | 0.531758383 | 0.889122684 |
| GSM519018_con | 0.419545734 | 0.40647269 | 0.806795457 | 0.830863225 | 0.525924256 | 0.68783293 | 0.475227288 | 0.563799772 | 0.52309221 | 0.488693925 | 0.925788889 |
| GSM519020_con | 0.536824137 | 0.478290085 | 0.809207247 | 0.850437324 | 0.534257783 | 0.638778285 | 0.4658682 | 0.603581846 | 0.567867279 | 0.504751083 | 0.935553937 |
| GSM519022_con | 0.689111254 | 0.472035055 | 0.860142776 | 0.838574569 | 0.524924283 | 0.656325861 | 0.423517153 | 0.572191506 | 0.547198308 | 0.482423442 | 0.890007128 |
| GSM519024_con | 0.511583377 | 0.561240481 | 0.866369473 | 0.865323898 | 0.563566857 | 0.616127105 | 0.450128322 | 0.633461705 | 0.514031288 | 0.527589323 | 0.887342786 |
| GSM519026_con | 0.643782252 | 0.488620378 | 0.869337873 | 0.862175521 | 0.605891907 | 0.619843337 | 0.477936019 | 0.625059343 | 0.543693829 | 0.495102841 | 0.907641978 |
| GSM519028_con | 0.675833395 | 0.488395123 | 0.82244772 | 0.828431452 | 0.571230802 | 0.616982336 | 0.468685391 | 0.567390342 | 0.622980263 | 0.477232777 | 0.888234001 |
| GSM519030_con | 0.538161415 | 0.521929409 | 0.88298337 | 0.834993018 | 0.536246362 | 0.653843066 | 0.458034234 | 0.569385863 | 0.59620071 | 0.493284903 | 0.898277159 |
| GSM519032_con | 0.57949149 | 0.358462394 | 0.785440113 | 0.858217191 | 0.521873897 | 0.607973136 | 0.499980576 | 0.587252856 | 0.607033354 | 0.526222456 | 0.915299985 |
| GSM519034_con | 0.455968335 | 0.478919705 | 0.786607536 | 0.863407038 | 0.539700893 | 0.655565804 | 0.488763696 | 0.620145161 | 0.553145663 | 0.493505679 | 0.927603439 |
| GSM519036_con | 0.581670085 | 0.576582905 | 0.878111854 | 0.851972144 | 0.588049639 | 0.590809672 | 0.528104172 | 0.599648424 | 0.608569161 | 0.498565946 | 0.898455649 |
| GSM519038_con | 0.557058273 | 0.494673865 | 0.813416799 | 0.870683697 | 0.526381526 | 0.636573448 | 0.476525773 | 0.589571996 | 0.532851982 | 0.530131613 | 0.925233525 |
| GSM519040_con | 0.699117549 | 0.493682655 | 0.852384642 | 0.848323793 | 0.5471998 | 0.638204406 | 0.511158349 | 0.608764262 | 0.6377419 | 0.527384938 | 0.927052068 |
| GSM519042_con | 0.572872854 | 0.488078458 | 0.804182277 | 0.885764405 | 0.505386955 | 0.605432638 | 0.464955539 | 0.62702598 | 0.547029439 | 0.565077786 | 0.933087618 |
| GSM519044_con | 0.465163203 | 0.484256784 | 0.852609595 | 0.844872385 | 0.545882486 | 0.594295389 | 0.45180923 | 0.602071109 | 0.578097328 | 0.531711695 | 0.907817611 |
| GSM519046_con | 0.63985433 | 0.462129535 | 0.819188196 | 0.879732406 | 0.555860774 | 0.667993824 | 0.422835905 | 0.576817839 | 0.579929537 | 0.535692588 | 0.913937021 |
| GSM519048_con | 0.657512431 | 0.440198449 | 0.819195348 | 0.849733986 | 0.549605676 | 0.624682534 | 0.486780269 | 0.573132609 | 0.551376171 | 0.540361507 | 0.942164552 |
| GSM519050_con | 0.567249177 | 0.516944025 | 0.8636174 | 0.862439579 | 0.578221034 | 0.627253223 | 0.490257759 | 0.567097755 | 0.618236495 | 0.509413562 | 0.890842374 |
| GSM519052_con | 0.542804089 | 0.486730982 | 0.85000502 | 0.843363651 | 0.553170817 | 0.612124936 | 0.484307454 | 0.559593341 | 0.560132269 | 0.521220979 | 0.909711884 |
| GSM519054_con | 0.646251051 | 0.583233317 | 0.851856969 | 0.854475532 | 0.532136733 | 0.63255873 | 0.462309332 | 0.588872545 | 0.607817996 | 0.519142268 | 0.95830707 |
| GSM519056_con | 0.495018597 | 0.546224618 | 0.88108642 | 0.822634877 | 0.563134963 | 0.582634069 | 0.419729106 | 0.573570633 | 0.572462592 | 0.508358435 | 0.917159871 |
| GSM519058_con | 0.588544378 | 0.430815921 | 0.826215456 | 0.828018659 | 0.539229753 | 0.711189924 | 0.438173664 | 0.530468685 | 0.539517176 | 0.48858131 | 0.898218024 |
| GSM519060_con | 0.667714008 | 0.469231575 | 0.865769851 | 0.847115022 | 0.554988405 | 0.649857378 | 0.451101453 | 0.554893383 | 0.591179356 | 0.523663306 | 0.911368303 |
| GSM519062_con | 0.676460415 | 0.440315409 | 0.831191237 | 0.858779167 | 0.539957623 | 0.668404207 | 0.460027721 | 0.56230537 | 0.59863392 | 0.520281694 | 0.927063921 |
| GSM519064_con | 0.72274499 | 0.484923097 | 0.8136121 | 0.826070948 | 0.556535002 | 0.596086014 | 0.451920526 | 0.567113355 | 0.691838997 | 0.538591119 | 0.907454865 |
| GSM519066_con | 0.546786072 | 0.357712857 | 0.778758407 | 0.854687596 | 0.564226166 | 0.611730084 | 0.516997669 | 0.572099348 | 0.558525168 | 0.531911176 | 0.894613937 |
| GSM519068_con | 0.473093394 | 0.472189948 | 0.851722235 | 0.841696798 | 0.568607083 | 0.604860613 | 0.520095917 | 0.583225501 | 0.646797713 | 0.529800063 | 0.941779059 |
| GSM519070_con | 0.585912452 | 0.595333486 | 0.877048697 | 0.80626076 | 0.568283398 | 0.704743537 | 0.459128981 | 0.537090606 | 0.56141991 | 0.4715348 | 0.916892326 |
| GSM519072_con | 0.527872083 | 0.529919632 | 0.879124827 | 0.844754469 | 0.522233507 | 0.614938186 | 0.451522751 | 0.588612274 | 0.521372483 | 0.502849151 | 0.922431847 |
| GSM519074_con | 0.589556744 | 0.485826164 | 0.85307308 | 0.839274307 | 0.554072622 | 0.682165886 | 0.459197185 | 0.533990049 | 0.582365529 | 0.50268732 | 0.902450177 |
| GSM519076_con | 0.606598939 | 0.467893023 | 0.788732509 | 0.849810746 | 0.496605421 | 0.597178466 | 0.398109747 | 0.5077174 | 0.560595498 | 0.49384101 | 0.899044546 |
| GSM519078_con | 0.624029496 | 0.496234734 | 0.847347642 | 0.869912604 | 0.533003303 | 0.63537143 | 0.441275288 | 0.587066725 | 0.550863946 | 0.521595775 | 0.897955408 |
| GSM519080_con | 0.598083061 | 0.434111525 | 0.79668074 | 0.842840834 | 0.524127089 | 0.645940059 | 0.47619145 | 0.559338551 | 0.494022603 | 0.496285928 | 0.912978062 |
| GSM519082_con | 0.621219379 | 0.536537673 | 0.876963702 | 0.820372559 | 0.5437063 | 0.602173439 | 0.414678717 | 0.593116735 | 0.53527337 | 0.513633975 | 0.886284083 |
| GSM308600_treat | 0.645802024 | 0.489410172 | 0.866455279 | 0.783809575 | 0.504568135 | 0.615396105 | 0.461835054 | 0.478482522 | 0.650627322 | 0.546412404 | 0.940811989 |
| GSM308601_treat | 0.563395688 | 0.469843798 | 0.816008802 | 0.85623958 | 0.501523975 | 0.675895984 | 0.450307701 | 0.570455572 | 0.572794877 | 0.505068072 | 0.911079553 |
| GSM308603_treat | 0.507431663 | 0.479393501 | 0.81488123 | 0.860910545 | 0.519538421 | 0.624841254 | 0.425140131 | 0.590926133 | 0.53315376 | 0.531731614 | 0.916338952 |
| GSM308604_treat | 0.558791763 | 0.46364406 | 0.860667905 | 0.867999792 | 0.571960668 | 0.58211468 | 0.482484706 | 0.606419699 | 0.553609672 | 0.513650841 | 0.897210686 |
| GSM308605_treat | 0.563475006 | 0.429872276 | 0.800114156 | 0.866487288 | 0.579451542 | 0.587893965 | 0.462469777 | 0.587361053 | 0.52317847 | 0.511583159 | 0.926478085 |
| GSM308606_treat | 0.420760521 | 0.452166358 | 0.806749013 | 0.880016899 | 0.511921146 | 0.626224281 | 0.496154192 | 0.551715361 | 0.523089368 | 0.529420954 | 0.916181948 |
| GSM308607_treat | 0.541696649 | 0.498427217 | 0.846384286 | 0.85793845 | 0.5642969 | 0.578474639 | 0.488742703 | 0.60931182 | 0.526913896 | 0.509864206 | 0.915620116 |
| GSM308608_treat | 0.489423931 | 0.537205984 | 0.854559254 | 0.877219461 | 0.524191814 | 0.608687656 | 0.474067387 | 0.570050053 | 0.605459219 | 0.49809964 | 0.918177686 |
| GSM308609_treat | 0.574302073 | 0.495643437 | 0.838550287 | 0.849432198 | 0.554398111 | 0.627969483 | 0.454678078 | 0.5908225 | 0.62985643 | 0.517358929 | 0.947693164 |
| GSM308610_treat | 0.669667676 | 0.470116283 | 0.858040721 | 0.827355422 | 0.516075537 | 0.642001228 | 0.410010319 | 0.595476213 | 0.566551798 | 0.525262004 | 0.900764304 |
| GSM308611_treat | 0.473031249 | 0.460135005 | 0.833693414 | 0.854837807 | 0.572185137 | 0.60496249 | 0.43803869 | 0.593185082 | 0.516817851 | 0.509129539 | 0.935149025 |
| GSM308613_treat | 0.649740952 | 0.496413646 | 0.840903848 | 0.856162497 | 0.509376716 | 0.5734602 | 0.481273572 | 0.587456855 | 0.537696013 | 0.487157135 | 0.905087539 |
| GSM308614_treat | 0.492218973 | 0.482715872 | 0.858055794 | 0.857884483 | 0.478494895 | 0.60660893 | 0.423431102 | 0.598802968 | 0.555035044 | 0.514516732 | 0.926577349 |
| GSM308615_treat | 0.610260106 | 0.448210119 | 0.852303114 | 0.846650975 | 0.521069781 | 0.646847654 | 0.505464902 | 0.623648416 | 0.559269001 | 0.496521941 | 0.922352479 |
| GSM308616_treat | 0.668734061 | 0.507929399 | 0.839297481 | 0.873361617 | 0.537087628 | 0.606010613 | 0.460279547 | 0.613752242 | 0.651971385 | 0.539082515 | 0.908235166 |
| GSM308617_treat | 0.6429339 | 0.487301062 | 0.830566494 | 0.839572142 | 0.50284317 | 0.563098639 | 0.469457146 | 0.590089096 | 0.554028748 | 0.494504837 | 0.913881832 |
| GSM308618_treat | 0.535974879 | 0.449171099 | 0.820015134 | 0.87295801 | 0.537719789 | 0.560088665 | 0.479375033 | 0.608615736 | 0.570564957 | 0.500872651 | 0.926498032 |
| GSM308620_treat | 0.468023427 | 0.498405394 | 0.811969849 | 0.85558122 | 0.548883728 | 0.573398908 | 0.472374915 | 0.584432905 | 0.492184098 | 0.53012433 | 0.890438515 |
| GSM308622_treat | 0.495726607 | 0.503360517 | 0.862908926 | 0.845195069 | 0.535718058 | 0.590386555 | 0.471203079 | 0.583003777 | 0.57244156 | 0.540306849 | 0.899244237 |
| GSM308623_treat | 0.601709044 | 0.484300564 | 0.82557245 | 0.861397828 | 0.529220038 | 0.643250522 | 0.347400394 | 0.575759052 | 0.604765609 | 0.508022834 | 0.925259763 |
| GSM308625_treat | 0.523634755 | 0.469242796 | 0.830235604 | 0.869401349 | 0.538133869 | 0.658822054 | 0.513581763 | 0.579544849 | 0.587074793 | 0.504991399 | 0.927262674 |
| GSM308626_treat | 0.62456918 | 0.463483142 | 0.823463991 | 0.860454466 | 0.565034702 | 0.641360016 | 0.474468573 | 0.533574045 | 0.54503141 | 0.540454557 | 0.927722893 |
| GSM308627_treat | 0.544325139 | 0.494303649 | 0.836110597 | 0.874636153 | 0.506807638 | 0.603969575 | 0.453933788 | 0.546276176 | 0.607847066 | 0.51052511 | 0.904424109 |
| GSM308629_treat | 0.660703014 | 0.538926962 | 0.835648408 | 0.847325379 | 0.532736086 | 0.577438915 | 0.435832328 | 0.598182075 | 0.574202821 | 0.507117781 | 0.910283449 |
| GSM308630_treat | 0.653895376 | 0.584939402 | 0.86949395 | 0.860906812 | 0.5387137 | 0.585524033 | 0.494553078 | 0.62276614 | 0.540047403 | 0.514278153 | 0.904163732 |
| GSM308631_treat | 0.623772387 | 0.511352833 | 0.793352378 | 0.861857582 | 0.57294101 | 0.590465087 | 0.473097105 | 0.580574295 | 0.600107601 | 0.511983043 | 0.936911342 |
| GSM308632_treat | 0.657263342 | 0.494362149 | 0.841482447 | 0.849014782 | 0.542774751 | 0.627423269 | 0.470600368 | 0.617560225 | 0.58572036 | 0.512809425 | 0.912907436 |
| GSM308634_treat | 0.619024001 | 0.50237174 | 0.827857908 | 0.879870708 | 0.565312469 | 0.613542767 | 0.425959467 | 0.561380455 | 0.662509498 | 0.50887391 | 0.907293933 |
| GSM308637_treat | 0.559545702 | 0.496603422 | 0.839754029 | 0.852363081 | 0.551426327 | 0.62485423 | 0.462696563 | 0.566737006 | 0.526478793 | 0.504139076 | 0.908609687 |
| GSM308639_treat | 0.525655377 | 0.47481502 | 0.809476861 | 0.877286583 | 0.510085652 | 0.592355776 | 0.488784417 | 0.584058288 | 0.605449334 | 0.527279683 | 0.910166634 |
| GSM308640_treat | 0.530055311 | 0.466083181 | 0.837607924 | 0.836484552 | 0.564572085 | 0.590413712 | 0.508427326 | 0.566356161 | 0.594337615 | 0.481521483 | 0.920613151 |
| GSM308641_treat | 0.78810635 | 0.512107933 | 0.816295821 | 0.837731282 | 0.534105518 | 0.616684616 | 0.477429273 | 0.578836945 | 0.643977257 | 0.482926245 | 0.922602869 |
| GSM308642_treat | 0.606532871 | 0.506850272 | 0.83684573 | 0.842444417 | 0.556306339 | 0.628770533 | 0.466453605 | 0.591743387 | 0.621480318 | 0.508990903 | 0.917662491 |
| GSM308644_treat | 0.531672879 | 0.462853224 | 0.797818741 | 0.878127152 | 0.548009711 | 0.626051839 | 0.485187304 | 0.557582399 | 0.628685537 | 0.501453216 | 0.924745801 |
| GSM308645_treat | 0.514824105 | 0.509737776 | 0.818224021 | 0.844008839 | 0.537998612 | 0.543259539 | 0.417918529 | 0.601642929 | 0.592324164 | 0.515031386 | 0.908594547 |
| GSM308646_treat | 0.505628495 | 0.443017098 | 0.807565998 | 0.879236145 | 0.539279873 | 0.599342598 | 0.513298555 | 0.56619188 | 0.558179638 | 0.516961239 | 0.90685004 |
| GSM308647_treat | 0.619432394 | 0.461375907 | 0.848407543 | 0.84853616 | 0.527735784 | 0.642333555 | 0.428202487 | 0.539743157 | 0.543371608 | 0.53428831 | 0.88782728 |
| GSM308650_treat | 0.592465809 | 0.500414222 | 0.818102654 | 0.857924209 | 0.548341368 | 0.599366305 | 0.492031204 | 0.583286326 | 0.660798805 | 0.505076413 | 0.930287973 |
| GSM308652_treat | 0.609590667 | 0.507608172 | 0.828143907 | 0.844550726 | 0.531695697 | 0.59556602 | 0.439568941 | 0.607514447 | 0.64585692 | 0.516877886 | 0.927380322 |
| GSM308653_treat | 0.599060523 | 0.474806343 | 0.827071957 | 0.859732426 | 0.533751372 | 0.588165313 | 0.464707198 | 0.590685768 | 0.552435121 | 0.542953487 | 0.930194941 |
| GSM308655_treat | 0.604479617 | 0.504307132 | 0.800803964 | 0.873739574 | 0.542536821 | 0.638413631 | 0.363074633 | 0.549424974 | 0.617842923 | 0.534845979 | 0.93763166 |
| GSM308657_treat | 0.501658477 | 0.485836565 | 0.827125957 | 0.844081182 | 0.553609686 | 0.59036659 | 0.382499648 | 0.567894447 | 0.603327112 | 0.48849711 | 0.929278671 |
| GSM308658_treat | 0.419136159 | 0.479903711 | 0.804643513 | 0.864478031 | 0.520657266 | 0.50467074 | 0.427830949 | 0.558781032 | 0.549239344 | 0.547089397 | 0.886946255 |
| GSM308659_treat | 0.574859219 | 0.428656368 | 0.780420793 | 0.877462382 | 0.526699903 | 0.592210892 | 0.469849919 | 0.53552772 | 0.675974391 | 0.510215802 | 0.921404777 |
| GSM308664_treat | 0.55473114 | 0.496419492 | 0.830490066 | 0.875428164 | 0.579729752 | 0.572424809 | 0.514782646 | 0.609581466 | 0.546095217 | 0.527412748 | 0.895486313 |
| GSM308666_treat | 0.47627927 | 0.476667947 | 0.803696392 | 0.837532156 | 0.496704548 | 0.729423905 | 0.446098878 | 0.566430797 | 0.527621108 | 0.515049162 | 0.930597341 |
| GSM308667_treat | 0.556356954 | 0.483129134 | 0.811130471 | 0.851526497 | 0.54192889 | 0.596791885 | 0.427040937 | 0.603507556 | 0.521221569 | 0.516252323 | 0.926221827 |
| GSM308668_treat | 0.498362372 | 0.43098025 | 0.79804003 | 0.895467793 | 0.5488353 | 0.644281409 | 0.474741638 | 0.592888606 | 0.54658072 | 0.496704742 | 0.921733235 |
| GSM308669_treat | 0.551151777 | 0.40745249 | 0.823350983 | 0.865943765 | 0.478186044 | 0.568276126 | 0.455666159 | 0.619070819 | 0.527898066 | 0.518638711 | 0.943459852 |
| GSM308670_treat | 0.619847059 | 0.491809651 | 0.865012038 | 0.852690979 | 0.514490033 | 0.665387744 | 0.454666209 | 0.602501509 | 0.640215464 | 0.539380336 | 0.909417209 |
| GSM308671_treat | 0.534184716 | 0.398098218 | 0.824614042 | 0.859477224 | 0.52713715 | 0.593793441 | 0.504173246 | 0.602296291 | 0.613263184 | 0.502124603 | 0.926511973 |
| GSM308672_treat | 0.5103432 | 0.439907372 | 0.834676393 | 0.860382945 | 0.495326453 | 0.641383086 | 0.473246505 | 0.612402488 | 0.666892586 | 0.534698529 | 0.91102089 |
| GSM308673_treat | 0.532351417 | 0.523362288 | 0.879196189 | 0.838648416 | 0.547384156 | 0.583334716 | 0.452433727 | 0.593689133 | 0.52698683 | 0.50916919 | 0.916184164 |
| GSM308674_treat | 0.604339219 | 0.561511442 | 0.857578244 | 0.875759038 | 0.534723227 | 0.559126321 | 0.453407503 | 0.607951511 | 0.538281337 | 0.511716154 | 0.905028522 |
| GSM308675_treat | 0.518751767 | 0.488615575 | 0.835178532 | 0.862125032 | 0.564627554 | 0.577494313 | 0.444549208 | 0.60532038 | 0.650220924 | 0.544397738 | 0.918247907 |
| GSM308676_treat | 0.466024502 | 0.53970879 | 0.868809789 | 0.843998874 | 0.495233917 | 0.59378963 | 0.441128895 | 0.56512741 | 0.532519749 | 0.523366315 | 0.920297129 |
| GSM308678_treat | 0.531331977 | 0.42577823 | 0.77478753 | 0.871403249 | 0.556123134 | 0.519799513 | 0.521459695 | 0.604422202 | 0.542041672 | 0.545446786 | 0.905996054 |
| GSM308679_treat | 0.591735802 | 0.4642476 | 0.855027517 | 0.851947454 | 0.543852142 | 0.645780346 | 0.340552049 | 0.560360471 | 0.499180643 | 0.515379018 | 0.929250543 |
| GSM308681_treat | 0.651796222 | 0.516170291 | 0.854371968 | 0.854027369 | 0.545564622 | 0.613754615 | 0.470967785 | 0.573065601 | 0.588656322 | 0.525836849 | 0.919748022 |
| GSM308685_treat | 0.551768797 | 0.480294827 | 0.829095069 | 0.856242073 | 0.496683759 | 0.631108041 | 0.371209071 | 0.56717758 | 0.644214352 | 0.525304618 | 0.9448478 |
| GSM308686_treat | 0.613729033 | 0.464459375 | 0.832783203 | 0.830046246 | 0.594188662 | 0.639689585 | 0.366969313 | 0.585206723 | 0.597776791 | 0.507889923 | 0.918605305 |
| GSM308688_treat | 0.637671095 | 0.44500454 | 0.804877323 | 0.795491837 | 0.541691347 | 0.64651552 | 0.446259603 | 0.582501615 | 0.622797438 | 0.522208819 | 0.889029191 |
| GSM308689_treat | 0.460471527 | 0.45268137 | 0.78424242 | 0.83955236 | 0.552138817 | 0.567909558 | 0.429204639 | 0.622778604 | 0.562296753 | 0.499283319 | 0.907020655 |
| GSM308690_treat | 0.671469034 | 0.483617394 | 0.899696338 | 0.819611205 | 0.540204989 | 0.585383961 | 0.471619355 | 0.595000095 | 0.648831187 | 0.495367331 | 0.914597789 |
| GSM308691_treat | 0.51929818 | 0.415912109 | 0.801138241 | 0.845393951 | 0.538171138 | 0.555668627 | 0.423760101 | 0.610717633 | 0.608920482 | 0.499557639 | 0.920485532 |
| GSM308692_treat | 0.595854408 | 0.476806375 | 0.857013492 | 0.87383582 | 0.546117166 | 0.612510435 | 0.431282773 | 0.549507343 | 0.525111727 | 0.513885789 | 0.926100826 |
| GSM308693_treat | 0.791321893 | 0.543528103 | 0.813641073 | 0.836411174 | 0.544555034 | 0.59995199 | 0.451476357 | 0.575185746 | 0.693129743 | 0.541345264 | 0.898154596 |
| GSM308694_treat | 0.594244803 | 0.468460126 | 0.839986197 | 0.867150804 | 0.529580676 | 0.62986792 | 0.425846805 | 0.592423838 | 0.535438061 | 0.503664772 | 0.91493633 |
| GSM308695_treat | 0.515624472 | 0.46786692 | 0.873809832 | 0.838880258 | 0.533025433 | 0.60401201 | 0.467500658 | 0.609386514 | 0.517369676 | 0.530080733 | 0.920861588 |
| GSM308696_treat | 0.604587727 | 0.494235084 | 0.806153728 | 0.864349354 | 0.540272359 | 0.588612306 | 0.456386143 | 0.631972879 | 0.564125784 | 0.479641069 | 0.917244847 |
| GSM308697_treat | 0.574315732 | 0.546235642 | 0.809387935 | 0.853615153 | 0.536645633 | 0.556633974 | 0.489775224 | 0.593938808 | 0.64011742 | 0.551761279 | 0.915511735 |
| GSM308699_treat | 0.530288088 | 0.43665066 | 0.785390098 | 0.847836447 | 0.518743126 | 0.617745934 | 0.462273872 | 0.57961162 | 0.553607398 | 0.519284282 | 0.932404779 |
| GSM308700_treat | 0.478722807 | 0.500038337 | 0.834770429 | 0.864116342 | 0.510064623 | 0.572617119 | 0.429133113 | 0.596618566 | 0.544807756 | 0.547664974 | 0.91801078 |
| GSM308703_treat | 0.399898582 | 0.477493563 | 0.818859832 | 0.838291413 | 0.505617555 | 0.52180097 | 0.43129527 | 0.557267815 | 0.614652258 | 0.512925136 | 0.875708203 |
| GSM308704_treat | 0.600795528 | 0.549700055 | 0.875862764 | 0.86231333 | 0.549483822 | 0.602078933 | 0.478915496 | 0.627384152 | 0.606491479 | 0.509327704 | 0.931631883 |
| GSM308705_treat | 0.57709918 | 0.406105062 | 0.821370507 | 0.880010345 | 0.526697353 | 0.602001807 | 0.374901644 | 0.618363975 | 0.55575215 | 0.500101869 | 0.925170599 |
| GSM308707_treat | 0.494013615 | 0.50193455 | 0.823024591 | 0.867486839 | 0.517252102 | 0.645731891 | 0.493743198 | 0.561490809 | 0.469367889 | 0.491347285 | 0.917204076 |
| GSM308708_treat | 0.607030721 | 0.484183605 | 0.816845695 | 0.877006316 | 0.461753325 | 0.558573984 | 0.474220257 | 0.595408136 | 0.494855011 | 0.535701257 | 0.898608144 |
| GSM308710_treat | 0.456319898 | 0.447480455 | 0.812158454 | 0.844931958 | 0.572632357 | 0.614986691 | 0.543477819 | 0.578808719 | 0.518976468 | 0.535100222 | 0.916835701 |
| GSM308711_treat | 0.543733247 | 0.494002241 | 0.857200457 | 0.85552693 | 0.567452394 | 0.653635707 | 0.425352495 | 0.563099089 | 0.557990834 | 0.513379587 | 0.918095874 |
| GSM308715_treat | 0.490590387 | 0.43146915 | 0.811037084 | 0.846490817 | 0.551074717 | 0.561995672 | 0.485910022 | 0.623339851 | 0.603879928 | 0.482544941 | 0.957776682 |
| GSM308717_treat | 0.466929383 | 0.435190086 | 0.832865752 | 0.868938518 | 0.546004601 | 0.569623611 | 0.432564643 | 0.562366162 | 0.525637374 | 0.525255945 | 0.880208476 |
| GSM308718_treat | 0.587551277 | 0.550180554 | 0.858598555 | 0.839933389 | 0.507358949 | 0.60698055 | 0.442752894 | 0.585929521 | 0.622999173 | 0.529118276 | 0.934525712 |
| GSM308719_treat | 0.562581686 | 0.443983536 | 0.792321135 | 0.888553565 | 0.547069727 | 0.59397907 | 0.458171746 | 0.559966764 | 0.511227054 | 0.504875959 | 0.913818748 |
| GSM308723_treat | 0.608697519 | 0.485122083 | 0.830402701 | 0.83806455 | 0.544415356 | 0.590806435 | 0.443652954 | 0.57959551 | 0.631080434 | 0.533301032 | 0.915490023 |
| GSM308724_treat | 0.65441148 | 0.544215955 | 0.884748553 | 0.830568622 | 0.538457353 | 0.612086116 | 0.398433051 | 0.570451904 | 0.625456367 | 0.4726893 | 0.906719778 |
| GSM308725_treat | 0.597936351 | 0.435659124 | 0.783095425 | 0.914109908 | 0.49826362 | 0.612007931 | 0.448132705 | 0.533362192 | 0.525016317 | 0.559938747 | 0.920674855 |
| GSM308727_treat | 0.582852094 | 0.495884834 | 0.857381921 | 0.835369699 | 0.560379344 | 0.685448871 | 0.496215648 | 0.594612223 | 0.578936214 | 0.503352292 | 0.928441485 |
| GSM308734_treat | 0.535426455 | 0.472158358 | 0.84004121 | 0.867500982 | 0.514836229 | 0.594309391 | 0.459117549 | 0.538591248 | 0.591239091 | 0.538274321 | 0.90347851 |
| GSM308736_treat | 0.749773654 | 0.500669026 | 0.823869363 | 0.872688391 | 0.553060365 | 0.607572232 | 0.455254752 | 0.571787299 | 0.552046558 | 0.481811697 | 0.898343918 |
| GSM308737_treat | 0.592634651 | 0.521586471 | 0.867589052 | 0.876417714 | 0.503216856 | 0.616981989 | 0.47275642 | 0.557008004 | 0.656162541 | 0.514405209 | 0.916944584 |
| GSM308741_treat | 0.669341424 | 0.550975385 | 0.91158683 | 0.84763238 | 0.486713437 | 0.613083637 | 0.430781618 | 0.57634648 | 0.565568621 | 0.50240669 | 0.922276271 |
| GSM308742_treat | 0.42867781 | 0.454323901 | 0.840049049 | 0.852026053 | 0.50037051 | 0.62492639 | 0.479132771 | 0.618067337 | 0.564489932 | 0.547776135 | 0.924115525 |
| GSM308743_treat | 0.39331517 | 0.390050317 | 0.843548536 | 0.914091546 | 0.543193776 | 0.590953677 | 0.344526518 | 0.581474071 | 0.525307726 | 0.52594846 | 0.907862021 |
| GSM308745_treat | 0.460868513 | 0.504706288 | 0.841930083 | 0.855908401 | 0.53842711 | 0.596333996 | 0.456022001 | 0.564232312 | 0.542788867 | 0.506519285 | 0.932469565 |
| GSM308747_treat | 0.305842201 | 0.455580069 | 0.813571827 | 0.897274769 | 0.527943819 | 0.582484161 | 0.48754042 | 0.576557925 | 0.559096431 | 0.522383389 | 0.903724175 |
| GSM308752_treat | 0.601608318 | 0.500425602 | 0.858931841 | 0.828412853 | 0.562724373 | 0.620458239 | 0.501908253 | 0.59765881 | 0.51267954 | 0.523124124 | 0.8962149 |
| GSM308754_treat | 0.550310504 | 0.523585688 | 0.863942886 | 0.844975018 | 0.55187506 | 0.571101692 | 0.505181593 | 0.615901113 | 0.555256176 | 0.535662966 | 0.923740474 |
| GSM308755_treat | 0.590999299 | 0.506934124 | 0.860209823 | 0.8490273 | 0.545933171 | 0.69606488 | 0.527203662 | 0.557987565 | 0.543140342 | 0.507620079 | 0.925765404 |
| GSM308756_treat | 0.622487594 | 0.546000515 | 0.890752374 | 0.854291331 | 0.552608992 | 0.563833589 | 0.506230897 | 0.593875961 | 0.606003952 | 0.517651032 | 0.930064193 |
| GSM308759_treat | 0.562476031 | 0.48494393 | 0.847318273 | 0.86062481 | 0.523110492 | 0.625335245 | 0.383206518 | 0.597370489 | 0.623214812 | 0.538446021 | 0.936876374 |
| GSM308773_treat | 0.63630755 | 0.547491847 | 0.877709909 | 0.876558804 | 0.573060632 | 0.60469033 | 0.492889015 | 0.578744575 | 0.563512431 | 0.505793962 | 0.928872305 |
| GSM308775_treat | 0.578958719 | 0.518463552 | 0.841504787 | 0.844440245 | 0.545955511 | 0.666892949 | 0.475584145 | 0.515111368 | 0.581788979 | 0.509556174 | 0.919720275 |
| GSM308776_treat | 0.683619215 | 0.523845565 | 0.851730584 | 0.847645233 | 0.543409945 | 0.625660674 | 0.462934348 | 0.544028681 | 0.717470962 | 0.466374551 | 0.912697292 |
| GSM308778_treat | 0.516657729 | 0.495843069 | 0.818790523 | 0.84810468 | 0.524826154 | 0.5855469 | 0.421379742 | 0.553274787 | 0.490287693 | 0.512244622 | 0.896563092 |
| GSM308780_treat | 0.586896362 | 0.51767027 | 0.855043041 | 0.848971644 | 0.541733101 | 0.616682611 | 0.456216787 | 0.56754003 | 0.553720371 | 0.524660529 | 0.881886202 |
| GSM308781_treat | 0.427238138 | 0.491892059 | 0.77014218 | 0.886064719 | 0.541776044 | 0.616964453 | 0.397329758 | 0.559302978 | 0.526609346 | 0.499621886 | 0.923745534 |
| GSM308782_treat | 0.46500952 | 0.425217841 | 0.845837102 | 0.848795319 | 0.521834524 | 0.616253966 | 0.44087714 | 0.581358813 | 0.641390955 | 0.530326298 | 0.930547851 |
| GSM308783_treat | 0.611158165 | 0.565947817 | 0.896354325 | 0.878581246 | 0.56854697 | 0.613415065 | 0.455249285 | 0.657424435 | 0.655268396 | 0.498672162 | 0.91379022 |
| GSM308784_treat | 0.612918439 | 0.537934806 | 0.88039534 | 0.897693716 | 0.516419246 | 0.566154817 | 0.454564523 | 0.594396737 | 0.644740405 | 0.509523825 | 0.899158826 |
| GSM518690_treat | 0.655426347 | 0.464139126 | 0.831448634 | 0.870687136 | 0.525343782 | 0.63515582 | 0.427648811 | 0.542631103 | 0.616775285 | 0.454406643 | 0.89262952 |
| GSM518691_treat | 0.551810994 | 0.543525172 | 0.853092397 | 0.832637797 | 0.519644156 | 0.571935147 | 0.479982997 | 0.596805802 | 0.509818705 | 0.537150343 | 0.922881561 |
| GSM518692_treat | 0.64606173 | 0.456451241 | 0.83662159 | 0.860781095 | 0.557856587 | 0.601798649 | 0.447092537 | 0.58978086 | 0.680914677 | 0.509657612 | 0.912098928 |
| GSM518693_treat | 0.600339962 | 0.455127181 | 0.837384011 | 0.862675487 | 0.52739597 | 0.615746847 | 0.420325793 | 0.622997636 | 0.538187001 | 0.513850113 | 0.909241125 |
| GSM518694_treat | 0.585526552 | 0.623940049 | 0.798948328 | 0.833774324 | 0.528212051 | 0.661679861 | 0.444683414 | 0.608639728 | 0.656292129 | 0.551827058 | 0.940075298 |
| GSM518695_treat | 0.516121357 | 0.489114432 | 0.81578578 | 0.870752364 | 0.569495043 | 0.681402799 | 0.435284275 | 0.55896906 | 0.615057059 | 0.535401054 | 0.915122292 |
| GSM518696_treat | 0.658631418 | 0.589551329 | 0.849530418 | 0.831689755 | 0.507810933 | 0.627011251 | 0.417192613 | 0.61559425 | 0.576971408 | 0.541867231 | 0.936407199 |
| GSM518697_treat | 0.75521678 | 0.630967824 | 0.836720647 | 0.88813154 | 0.515797555 | 0.601022426 | 0.392520568 | 0.575499212 | 0.58776335 | 0.549165013 | 0.933984881 |
| GSM518698_treat | 0.514984251 | 0.395967601 | 0.758304252 | 0.877421949 | 0.549616098 | 0.583819765 | 0.412897496 | 0.626854095 | 0.61689381 | 0.512849317 | 0.900843843 |
| GSM518699_treat | 0.485909762 | 0.471184775 | 0.782084427 | 0.862723754 | 0.501897406 | 0.575785731 | 0.445064464 | 0.550822423 | 0.52252562 | 0.513222005 | 0.902604467 |
| GSM518700_treat | 0.623173972 | 0.542053588 | 0.832664676 | 0.789129695 | 0.539298839 | 0.511727089 | 0.519857494 | 0.619279706 | 0.681425956 | 0.53334788 | 0.915547751 |
| GSM518701_treat | 0.5907486 | 0.501693512 | 0.840534821 | 0.874875131 | 0.566347348 | 0.641212577 | 0.44467992 | 0.551849687 | 0.575612508 | 0.51419791 | 0.913709554 |
| GSM518702_treat | 0.339873534 | 0.46367679 | 0.781715132 | 0.877992035 | 0.50960309 | 0.546584696 | 0.492908347 | 0.546841938 | 0.551143669 | 0.512630438 | 0.881111524 |
| GSM518703_treat | 0.628558372 | 0.579570404 | 0.945955993 | 0.796619545 | 0.514716457 | 0.574918636 | 0.412491845 | 0.562081375 | 0.524184505 | 0.504801302 | 0.886895482 |
| GSM518704_treat | 0.698755513 | 0.491193077 | 0.810888383 | 0.853275584 | 0.506241692 | 0.659905037 | 0.402135787 | 0.525279262 | 0.52753444 | 0.498580534 | 0.911280871 |
| GSM518705_treat | 0.547589022 | 0.630395456 | 0.878136762 | 0.812550204 | 0.551275607 | 0.576401507 | 0.457603331 | 0.618541801 | 0.620516281 | 0.525602852 | 0.937969472 |
| GSM518706_treat | 0.530160879 | 0.45807924 | 0.822952523 | 0.898718107 | 0.548137349 | 0.614753672 | 0.423376621 | 0.548085621 | 0.612909151 | 0.478525668 | 0.922518046 |
| GSM518707_treat | 0.732964022 | 0.512091325 | 0.807684592 | 0.839019409 | 0.543856315 | 0.7324946 | 0.407723907 | 0.636664032 | 0.61189281 | 0.515046278 | 0.946875108 |
| GSM518708_treat | 0.557630059 | 0.464284263 | 0.858179783 | 0.831736271 | 0.537306154 | 0.63023765 | 0.500330086 | 0.595838469 | 0.585473193 | 0.505950716 | 0.908967879 |
| GSM518709_treat | 0.684282524 | 0.563756167 | 0.830295137 | 0.843090901 | 0.556473074 | 0.755068312 | 0.400552767 | 0.60452484 | 0.560489539 | 0.506579263 | 0.926375329 |
| GSM518710_treat | 0.512900792 | 0.490876733 | 0.840024643 | 0.83931635 | 0.522250739 | 0.635644163 | 0.481465373 | 0.565653464 | 0.581870256 | 0.504208199 | 0.917916319 |
| GSM518711_treat | 0.4298803 | 0.487721266 | 0.874304118 | 0.821732211 | 0.536378118 | 0.590867341 | 0.366926603 | 0.583020528 | 0.611953874 | 0.509117462 | 0.927949682 |
| GSM518712_treat | 0.664186909 | 0.493920825 | 0.844941836 | 0.83040065 | 0.538248389 | 0.615899407 | 0.46735456 | 0.53861664 | 0.587094789 | 0.495137107 | 0.896647947 |
| GSM518713_treat | 0.570382222 | 0.494759894 | 0.867449861 | 0.830734216 | 0.537594479 | 0.6409188 | 0.436562781 | 0.583447588 | 0.574241636 | 0.501940018 | 0.915177131 |
| GSM518714_treat | 0.508170381 | 0.494260619 | 0.810114857 | 0.8249063 | 0.513298525 | 0.67842693 | 0.422655431 | 0.569990708 | 0.5306443 | 0.480634473 | 0.916042027 |
| GSM518715_treat | 0.553520809 | 0.498853154 | 0.885765054 | 0.848579149 | 0.508549201 | 0.605251988 | 0.428363913 | 0.599325118 | 0.598630266 | 0.495596137 | 0.920979409 |
| GSM518716_treat | 0.592380109 | 0.522805921 | 0.833143997 | 0.85139801 | 0.528830059 | 0.575635847 | 0.443772006 | 0.614923198 | 0.584525941 | 0.528555099 | 0.917138422 |
| GSM518717_treat | 0.434658697 | 0.531564379 | 0.829056791 | 0.837697227 | 0.549480875 | 0.48070318 | 0.554114241 | 0.653835246 | 0.653434399 | 0.550690914 | 0.900198541 |
| GSM518718_treat | 0.592857748 | 0.564003147 | 0.841363974 | 0.844573976 | 0.568919241 | 0.654036253 | 0.481850555 | 0.568757599 | 0.58130635 | 0.516595562 | 0.921888642 |
| GSM518719_treat | 0.60223777 | 0.468384118 | 0.839623447 | 0.83663822 | 0.562790302 | 0.677155264 | 0.442448316 | 0.561614099 | 0.629697308 | 0.496358265 | 0.922274126 |
| GSM518720_treat | 0.529774763 | 0.4171959 | 0.770157803 | 0.89804254 | 0.524718465 | 0.544438349 | 0.518972938 | 0.55486654 | 0.52078285 | 0.51450891 | 0.909706172 |
| GSM518721_treat | 0.552568798 | 0.55603995 | 0.855798746 | 0.843747439 | 0.552280924 | 0.494838347 | 0.576065814 | 0.601683839 | 0.524990022 | 0.544818654 | 0.896346374 |
| GSM518722_treat | 0.536917327 | 0.540673519 | 0.882033991 | 0.811679973 | 0.538151295 | 0.555787278 | 0.501638109 | 0.587326362 | 0.582078986 | 0.510010069 | 0.898746678 |
| GSM518723_treat | 0.433026832 | 0.467813821 | 0.876224575 | 0.843915935 | 0.539238758 | 0.580511655 | 0.468463583 | 0.593385561 | 0.583664861 | 0.51742419 | 0.934212231 |
| GSM518724_treat | 0.620239523 | 0.548794902 | 0.85275034 | 0.826129976 | 0.54129325 | 0.651566476 | 0.416530252 | 0.598376354 | 0.54872876 | 0.490631039 | 0.931387487 |
| GSM518725_treat | 0.433942435 | 0.491113866 | 0.83468455 | 0.844118206 | 0.562209135 | 0.638854766 | 0.427098703 | 0.579165585 | 0.574850046 | 0.515319378 | 0.931361085 |
| GSM518726_treat | 0.444177473 | 0.494948304 | 0.763623724 | 0.876545327 | 0.524835963 | 0.551348765 | 0.533980444 | 0.618242327 | 0.567308489 | 0.571530507 | 0.916785213 |
| GSM518727_treat | 0.609062155 | 0.488810018 | 0.887006761 | 0.839286871 | 0.562636634 | 0.636385051 | 0.49511676 | 0.594789127 | 0.561345574 | 0.536079067 | 0.919377816 |
| GSM518728_treat | 0.531964379 | 0.517738205 | 0.847840987 | 0.840967147 | 0.527285105 | 0.655150613 | 0.453318187 | 0.511073274 | 0.520014934 | 0.463030384 | 0.877950822 |
| GSM518729_treat | 0.579470409 | 0.59018523 | 0.925370981 | 0.795127916 | 0.508452065 | 0.61156852 | 0.331233537 | 0.569905092 | 0.55598824 | 0.490097512 | 0.900565038 |
| GSM518730_treat | 0.566926621 | 0.462917365 | 0.801914155 | 0.872994342 | 0.524208751 | 0.647742135 | 0.449821161 | 0.581492981 | 0.608680518 | 0.521415194 | 0.928897698 |
| GSM518731_treat | 0.568893391 | 0.392974355 | 0.752131312 | 0.870182882 | 0.524635257 | 0.6207496 | 0.491530331 | 0.568974461 | 0.634333973 | 0.485979262 | 0.916925122 |
| GSM518732_treat | 0.620585896 | 0.486872287 | 0.849607795 | 0.830945116 | 0.532370888 | 0.685660035 | 0.375055894 | 0.57882244 | 0.633716065 | 0.51191541 | 0.898761237 |
| GSM518733_treat | 0.428274036 | 0.423026007 | 0.806515179 | 0.873395545 | 0.605404342 | 0.603303989 | 0.5052867 | 0.574960898 | 0.55793173 | 0.503469153 | 0.891280378 |
| GSM518734_treat | 0.50806092 | 0.40167945 | 0.779783717 | 0.869471428 | 0.530352086 | 0.569074377 | 0.513236976 | 0.580315796 | 0.564161585 | 0.521143421 | 0.901289507 |
| GSM518735_treat | 0.425841569 | 0.473435329 | 0.824881136 | 0.841170898 | 0.563966424 | 0.625563402 | 0.465229394 | 0.578645277 | 0.54546056 | 0.535191626 | 0.903848927 |
| GSM518736_treat | 0.467811305 | 0.483310339 | 0.836694535 | 0.847185598 | 0.52533078 | 0.687665214 | 0.452126496 | 0.590493703 | 0.515517199 | 0.491477229 | 0.908611967 |
| GSM518737_treat | 0.485187527 | 0.463685253 | 0.802717077 | 0.853455899 | 0.541038278 | 0.573533184 | 0.503970693 | 0.592167355 | 0.555425069 | 0.525608681 | 0.907813785 |
| GSM518738_treat | 0.519750462 | 0.477268437 | 0.885233156 | 0.864837489 | 0.533218314 | 0.622790096 | 0.484231221 | 0.598646954 | 0.532544168 | 0.502776566 | 0.908014115 |
| GSM518739_treat | 0.623834758 | 0.427507556 | 0.787373224 | 0.855110643 | 0.54841233 | 0.579722765 | 0.553277191 | 0.579832842 | 0.608385222 | 0.584335195 | 0.922447887 |
| GSM518740_treat | 0.470561759 | 0.399459878 | 0.740224075 | 0.869370117 | 0.526714547 | 0.612585676 | 0.433119093 | 0.596184137 | 0.598359933 | 0.530047801 | 0.907444511 |
| GSM518741_treat | 0.574427312 | 0.42751871 | 0.819646474 | 0.869905162 | 0.548932712 | 0.623455402 | 0.425726927 | 0.580034037 | 0.565443925 | 0.49844362 | 0.917438856 |
| GSM518742_treat | 0.435960776 | 0.432841965 | 0.791159344 | 0.851712971 | 0.539860206 | 0.591837088 | 0.472925144 | 0.588159949 | 0.551085615 | 0.513271364 | 0.902675495 |
| GSM518743_treat | 0.601663028 | 0.440452811 | 0.778930299 | 0.867987937 | 0.545515368 | 0.600339285 | 0.466878464 | 0.558848263 | 0.57821244 | 0.484637874 | 0.887269522 |
| GSM518744_treat | 0.487653332 | 0.426169348 | 0.794699502 | 0.855492557 | 0.574915033 | 0.597915444 | 0.508877201 | 0.582827585 | 0.589633328 | 0.501823656 | 0.913508189 |
| GSM518745_treat | 0.45363826 | 0.485208856 | 0.847121483 | 0.871132502 | 0.53261809 | 0.603455571 | 0.465596326 | 0.60229662 | 0.529121542 | 0.52268059 | 0.916951888 |
| GSM518746_treat | 0.596964892 | 0.425595075 | 0.831956032 | 0.857425314 | 0.538931971 | 0.643792728 | 0.450906577 | 0.60171018 | 0.68959175 | 0.502137048 | 0.922135053 |
| GSM518747_treat | 0.517488468 | 0.558775013 | 0.865383491 | 0.871585095 | 0.519028783 | 0.589075467 | 0.428851269 | 0.604447154 | 0.641434172 | 0.513197034 | 0.938157334 |
| GSM518748_treat | 0.489894353 | 0.464678268 | 0.844296699 | 0.839243499 | 0.556657505 | 0.600954781 | 0.48980903 | 0.595249759 | 0.547266801 | 0.576087549 | 0.913115248 |
| GSM518749_treat | 0.527610558 | 0.502871201 | 0.848753652 | 0.854957351 | 0.485105395 | 0.655102436 | 0.432817795 | 0.571228838 | 0.509090193 | 0.496184814 | 0.934923465 |
| GSM518750_treat | 0.47106628 | 0.433705756 | 0.798700247 | 0.849471065 | 0.530203794 | 0.660529362 | 0.460872972 | 0.600238859 | 0.566393032 | 0.513777706 | 0.893216711 |
| GSM518751_treat | 0.419082384 | 0.557389561 | 0.832452478 | 0.858995623 | 0.559944952 | 0.62724877 | 0.438176915 | 0.59726535 | 0.576580447 | 0.49226729 | 0.915282891 |
| GSM518752_treat | 0.468927671 | 0.506235895 | 0.858658066 | 0.849379871 | 0.52421915 | 0.675358377 | 0.498020868 | 0.606366191 | 0.562421796 | 0.523703063 | 0.910712916 |
| GSM518753_treat | 0.547790631 | 0.519007307 | 0.855622344 | 0.890419991 | 0.546754789 | 0.555273372 | 0.477827792 | 0.622698393 | 0.593508214 | 0.506510709 | 0.912854051 |
| GSM518754_treat | 0.730067156 | 0.567934807 | 0.914606804 | 0.819929818 | 0.537562602 | 0.582904986 | 0.371210383 | 0.596831205 | 0.659974984 | 0.510776946 | 0.9131025 |
| GSM518755_treat | 0.541391388 | 0.389011435 | 0.8082456 | 0.88506374 | 0.5552639 | 0.588378783 | 0.455714367 | 0.598184099 | 0.504155325 | 0.532441341 | 0.915174319 |
| GSM518756_treat | 0.40235563 | 0.535156587 | 0.866002582 | 0.832543829 | 0.531111657 | 0.54550461 | 0.54893878 | 0.595258254 | 0.590953478 | 0.521378854 | 0.916423996 |
| GSM518757_treat | 0.563876297 | 0.529545639 | 0.856413821 | 0.865005577 | 0.569984172 | 0.590439179 | 0.463894444 | 0.634432526 | 0.665893179 | 0.560131327 | 0.926058008 |
| GSM518758_treat | 0.455483349 | 0.436219849 | 0.834079108 | 0.865929547 | 0.552868316 | 0.610040023 | 0.513086969 | 0.624161855 | 0.549966347 | 0.5372873 | 0.915542177 |
| GSM518759_treat | 0.487390249 | 0.391782792 | 0.772363052 | 0.857035235 | 0.522648103 | 0.619481224 | 0.446281989 | 0.561687802 | 0.612786075 | 0.522584522 | 0.915051965 |
| GSM518760_treat | 0.343156262 | 0.530481082 | 0.885042668 | 0.863650905 | 0.546150858 | 0.636074973 | 0.451286919 | 0.570164774 | 0.554383773 | 0.496767577 | 0.900581177 |
| GSM518761_treat | 0.528429402 | 0.472332435 | 0.844619924 | 0.848326699 | 0.530548952 | 0.622716073 | 0.461211566 | 0.579115957 | 0.570989774 | 0.515210737 | 0.900063926 |
| GSM518762_treat | 0.626007041 | 0.495365808 | 0.877969171 | 0.830095949 | 0.529588345 | 0.646090967 | 0.364052605 | 0.573414739 | 0.539024646 | 0.462342509 | 0.909485874 |
| GSM518763_treat | 0.561590734 | 0.491917821 | 0.835833784 | 0.852623706 | 0.572097343 | 0.649887957 | 0.541588461 | 0.584095321 | 0.548665814 | 0.55597461 | 0.921304363 |
| GSM518764_treat | 0.590422365 | 0.454830964 | 0.835218221 | 0.841368012 | 0.543015708 | 0.62551164 | 0.464733521 | 0.540512529 | 0.635179289 | 0.487403807 | 0.910394188 |
| GSM518765_treat | 0.504720273 | 0.451574951 | 0.827959194 | 0.855098693 | 0.531576936 | 0.685568828 | 0.488290127 | 0.571701499 | 0.632448349 | 0.491976116 | 0.9338544 |
| GSM518766_treat | 0.67492512 | 0.544501381 | 0.856751681 | 0.841998948 | 0.528956388 | 0.584799766 | 0.468353796 | 0.565900101 | 0.580513709 | 0.565694399 | 0.921405532 |
| GSM518767_treat | 0.576122814 | 0.450348426 | 0.835304455 | 0.863883111 | 0.510722005 | 0.614663577 | 0.511075837 | 0.592189613 | 0.546889408 | 0.522233961 | 0.934914721 |
| GSM518768_treat | 0.485002469 | 0.48018054 | 0.818846299 | 0.862967912 | 0.539147745 | 0.609794197 | 0.453290179 | 0.588821298 | 0.586523358 | 0.526498723 | 0.91734834 |
| GSM518769_treat | 0.667714027 | 0.518515174 | 0.825624334 | 0.829970745 | 0.525828883 | 0.588425254 | 0.450535302 | 0.558979936 | 0.579255673 | 0.537627154 | 0.900628569 |
| GSM518770_treat | 0.496883486 | 0.460409647 | 0.852812434 | 0.843959186 | 0.542365034 | 0.614587362 | 0.475740682 | 0.582945443 | 0.593212625 | 0.509404795 | 0.940564721 |
| GSM518771_treat | 0.560703065 | 0.479310187 | 0.815637745 | 0.870754932 | 0.546407094 | 0.599282073 | 0.444216786 | 0.583306598 | 0.541826108 | 0.509815451 | 0.90753027 |
| GSM518772_treat | 0.531577659 | 0.475346383 | 0.864700607 | 0.840056954 | 0.546493915 | 0.752212304 | 0.394295597 | 0.567166931 | 0.54364299 | 0.462520293 | 0.924501143 |
| GSM518773_treat | 0.442854892 | 0.502096102 | 0.849085538 | 0.85428705 | 0.551893631 | 0.625785546 | 0.485327148 | 0.572666518 | 0.595760829 | 0.527011689 | 0.932383933 |
| GSM518774_treat | 0.41925816 | 0.466493454 | 0.838157827 | 0.843379217 | 0.531035106 | 0.593836396 | 0.444608291 | 0.596648367 | 0.54706823 | 0.512106792 | 0.899727871 |
| GSM518775_treat | 0.614318389 | 0.544896344 | 0.846159346 | 0.868521302 | 0.53604409 | 0.536169654 | 0.492691539 | 0.634843838 | 0.595635083 | 0.489372261 | 0.92633259 |
| GSM518776_treat | 0.549136147 | 0.518293613 | 0.867870904 | 0.834460059 | 0.517365728 | 0.531387243 | 0.497232713 | 0.606205985 | 0.605466921 | 0.510028183 | 0.902867344 |
| GSM518777_treat | 0.592631755 | 0.453318873 | 0.823347572 | 0.849933472 | 0.496013389 | 0.632748136 | 0.442614677 | 0.578252211 | 0.526225131 | 0.497077414 | 0.889222171 |
| GSM518778_treat | 0.538570875 | 0.475227069 | 0.841351234 | 0.855461408 | 0.558695359 | 0.583224279 | 0.458641777 | 0.571702955 | 0.576640656 | 0.512828754 | 0.92619791 |
| GSM518779_treat | 0.721723491 | 0.479868516 | 0.794270323 | 0.849393142 | 0.538511486 | 0.61956146 | 0.460396368 | 0.586711763 | 0.648297121 | 0.525326994 | 0.898834807 |
| GSM518780_treat | 0.633070571 | 0.516756633 | 0.861950508 | 0.843218206 | 0.513021377 | 0.569669454 | 0.511340202 | 0.577186599 | 0.659489096 | 0.491987016 | 0.931222457 |
| GSM518781_treat | 0.590724998 | 0.458668649 | 0.795733919 | 0.875179931 | 0.559608326 | 0.621419454 | 0.479951414 | 0.572319611 | 0.588413568 | 0.539818821 | 0.915875782 |
| GSM518782_treat | 0.598461724 | 0.563458301 | 0.904061434 | 0.866198097 | 0.553155475 | 0.534211495 | 0.498942492 | 0.604271272 | 0.550767555 | 0.519308095 | 0.89727456 |
| GSM518783_treat | 0.543167801 | 0.435579713 | 0.802860209 | 0.874136537 | 0.511699496 | 0.604893655 | 0.482073925 | 0.559560517 | 0.627890957 | 0.491779903 | 0.886518383 |
| GSM518784_treat | 0.560399261 | 0.492270905 | 0.836118526 | 0.858923778 | 0.531374314 | 0.627892423 | 0.479005441 | 0.58749543 | 0.592571546 | 0.487568399 | 0.914775074 |
| GSM518785_treat | 0.63750169 | 0.555361721 | 0.871488666 | 0.818342136 | 0.515974462 | 0.597084379 | 0.487235411 | 0.564164843 | 0.549665079 | 0.505658235 | 0.90332079 |
| GSM518786_treat | 0.679336429 | 0.518708772 | 0.876425306 | 0.856462267 | 0.556260066 | 0.610322118 | 0.427888279 | 0.582527626 | 0.609894412 | 0.500284459 | 0.91610874 |
| GSM518787_treat | 0.690718022 | 0.522136911 | 0.852183649 | 0.865445445 | 0.517259949 | 0.602997718 | 0.446833522 | 0.543874167 | 0.567541193 | 0.485183564 | 0.890848679 |
| GSM518788_treat | 0.464354951 | 0.494385327 | 0.848214831 | 0.8425287 | 0.558658975 | 0.574169438 | 0.422703027 | 0.611270472 | 0.579104753 | 0.527380016 | 0.932106943 |
| GSM518789_treat | 0.490476008 | 0.491788795 | 0.849564147 | 0.863858847 | 0.495421413 | 0.634120371 | 0.408199254 | 0.560628329 | 0.486975131 | 0.485694605 | 0.910289082 |
| GSM518790_treat | 0.455954922 | 0.454517096 | 0.850274875 | 0.84993411 | 0.556363386 | 0.571415011 | 0.486486749 | 0.624700839 | 0.595344876 | 0.509030021 | 0.927436527 |
| GSM518791_treat | 0.60293655 | 0.551677519 | 0.879304353 | 0.854868948 | 0.531758152 | 0.57235439 | 0.506689932 | 0.622162812 | 0.567119558 | 0.564710533 | 0.922267517 |
| GSM518792_treat | 0.563126129 | 0.484199032 | 0.887725917 | 0.826782448 | 0.55849177 | 0.603691236 | 0.46941757 | 0.596764453 | 0.606515785 | 0.498159055 | 0.884028453 |
| GSM518793_treat | 0.688093856 | 0.545661086 | 0.819649463 | 0.847480952 | 0.488491595 | 0.679737836 | 0.461710259 | 0.544413195 | 0.531749432 | 0.474330688 | 0.891575583 |
| GSM518794_treat | 0.655473427 | 0.550000468 | 0.842403255 | 0.83930325 | 0.542996466 | 0.597549096 | 0.504984331 | 0.560338111 | 0.609999491 | 0.511810043 | 0.93556081 |
| GSM518795_treat | 0.608585733 | 0.478857813 | 0.804556306 | 0.858399803 | 0.548250321 | 0.592550356 | 0.493908011 | 0.577278703 | 0.562236609 | 0.508601352 | 0.893802041 |
| GSM518796_treat | 0.565267744 | 0.491973695 | 0.806278419 | 0.841411246 | 0.524543918 | 0.651230415 | 0.425711353 | 0.586159054 | 0.609061874 | 0.557848506 | 0.912508508 |
| GSM518797_treat | 0.520202321 | 0.495533521 | 0.864194212 | 0.843219237 | 0.5640601 | 0.628911444 | 0.514469902 | 0.594977626 | 0.50452275 | 0.527534514 | 0.914985106 |
| GSM518798_treat | 0.633553386 | 0.486318066 | 0.853874121 | 0.838151742 | 0.540295504 | 0.635837473 | 0.422667506 | 0.558544118 | 0.564443872 | 0.505879291 | 0.900995193 |
| GSM518799_treat | 0.606939308 | 0.524649125 | 0.867781339 | 0.863410233 | 0.552450418 | 0.598483883 | 0.413655248 | 0.559769009 | 0.56398938 | 0.473554668 | 0.928466437 |
| GSM518800_treat | 0.581004142 | 0.438273708 | 0.816150613 | 0.871968941 | 0.521617302 | 0.639679193 | 0.455932606 | 0.59967857 | 0.637746307 | 0.485340713 | 0.926632513 |
| GSM518801_treat | 0.536087202 | 0.433697205 | 0.821049816 | 0.866601942 | 0.565284193 | 0.604905128 | 0.474219412 | 0.596748527 | 0.57839084 | 0.523100433 | 0.92283955 |
| GSM518802_treat | 0.382030541 | 0.46564215 | 0.825706896 | 0.860322492 | 0.548117945 | 0.638259263 | 0.467460037 | 0.574872293 | 0.604278796 | 0.502359147 | 0.906981665 |
| GSM518803_treat | 0.553478338 | 0.462331093 | 0.792070687 | 0.863366455 | 0.522514055 | 0.630943509 | 0.464410323 | 0.536024892 | 0.556403736 | 0.475700288 | 0.901796084 |
| GSM518804_treat | 0.537337392 | 0.49815648 | 0.814274021 | 0.857616909 | 0.556438581 | 0.640317356 | 0.465909907 | 0.609280975 | 0.588742221 | 0.503286058 | 0.90967891 |
| GSM518805_treat | 0.388221617 | 0.507676656 | 0.802677243 | 0.867605319 | 0.508807748 | 0.558300716 | 0.537347828 | 0.561591488 | 0.520833695 | 0.569562373 | 0.919071263 |
| GSM518806_treat | 0.623849464 | 0.488566204 | 0.843424275 | 0.860038549 | 0.564986037 | 0.630809457 | 0.447457944 | 0.584893011 | 0.530888746 | 0.508632905 | 0.896010451 |
| GSM518807_treat | 0.516378536 | 0.497263747 | 0.847272446 | 0.845545178 | 0.53415464 | 0.619186209 | 0.538798516 | 0.570908107 | 0.657656273 | 0.551232885 | 0.919018127 |
| GSM518808_treat | 0.446358472 | 0.568616693 | 0.881248358 | 0.845741993 | 0.545649334 | 0.622123791 | 0.482634348 | 0.565278578 | 0.519607603 | 0.529051715 | 0.924545925 |
| GSM518809_treat | 0.546525956 | 0.469783383 | 0.863033507 | 0.856356373 | 0.563941603 | 0.606035924 | 0.497788148 | 0.579219673 | 0.6363566 | 0.527844044 | 0.920149022 |
| GSM518810_treat | 0.631643143 | 0.509215108 | 0.865940414 | 0.871262347 | 0.582254486 | 0.567582357 | 0.459365475 | 0.623692396 | 0.590797695 | 0.564700593 | 0.932442389 |
| GSM518811_treat | 0.5981924 | 0.476764924 | 0.809426126 | 0.834308058 | 0.536609798 | 0.561057876 | 0.517619737 | 0.605844362 | 0.58445421 | 0.547866904 | 0.927424726 |
| GSM518812_treat | 0.555688624 | 0.470385496 | 0.843721216 | 0.847601387 | 0.55929975 | 0.627238405 | 0.520241505 | 0.585597386 | 0.663199694 | 0.551641726 | 0.911651426 |
| GSM518813_treat | 0.662143953 | 0.486215951 | 0.857674183 | 0.866005948 | 0.563532204 | 0.650273042 | 0.461223024 | 0.572595205 | 0.592011073 | 0.512352485 | 0.927123615 |
| GSM518814_treat | 0.455221018 | 0.436912531 | 0.821923784 | 0.85090331 | 0.570304286 | 0.643089784 | 0.501264617 | 0.578629969 | 0.527910826 | 0.50567403 | 0.888056434 |
| GSM518815_treat | 0.392378616 | 0.472326941 | 0.813142148 | 0.842010263 | 0.555002132 | 0.592167928 | 0.450447763 | 0.577861848 | 0.598326495 | 0.512242344 | 0.926747906 |
| GSM518816_treat | 0.614677253 | 0.483779583 | 0.794237458 | 0.876518127 | 0.548365213 | 0.496819236 | 0.650736254 | 0.60377161 | 0.610910288 | 0.550431216 | 0.885545865 |
| GSM518817_treat | 0.569838252 | 0.383239319 | 0.838524335 | 0.858699765 | 0.524421296 | 0.568755763 | 0.463411281 | 0.6279298 | 0.607730258 | 0.527001841 | 0.929393984 |
| GSM518818_treat | 0.550784572 | 0.537991207 | 0.870215195 | 0.834324938 | 0.496035389 | 0.595223733 | 0.526210891 | 0.619723901 | 0.562118703 | 0.535371441 | 0.914292745 |
| GSM518819_treat | 0.565000421 | 0.494068406 | 0.805386506 | 0.846844663 | 0.51206474 | 0.623773523 | 0.443027879 | 0.599996616 | 0.593250996 | 0.50210305 | 0.910326854 |
| GSM518820_treat | 0.703244541 | 0.491111947 | 0.825857387 | 0.847615602 | 0.555452039 | 0.636009583 | 0.447663872 | 0.551472314 | 0.571369843 | 0.503234049 | 0.918564487 |
| GSM518821_treat | 0.545442768 | 0.492127699 | 0.849413046 | 0.870746475 | 0.524667472 | 0.559842069 | 0.469991826 | 0.609977642 | 0.670415549 | 0.506410024 | 0.930282755 |
| GSM518822_treat | 0.587543356 | 0.515181486 | 0.865751601 | 0.853055416 | 0.514905869 | 0.671934532 | 0.460896748 | 0.591417182 | 0.518097263 | 0.476491119 | 0.938991925 |
| GSM518823_treat | 0.601306802 | 0.473439379 | 0.849846696 | 0.863923777 | 0.568419493 | 0.570532126 | 0.536288381 | 0.618399378 | 0.59413473 | 0.539423775 | 0.924067449 |
| GSM518824_treat | 0.427828573 | 0.487149889 | 0.811976895 | 0.86207214 | 0.541136278 | 0.612359229 | 0.518172397 | 0.629760501 | 0.554234272 | 0.541762362 | 0.934416405 |
| GSM518825_treat | 0.524469694 | 0.396140145 | 0.791931797 | 0.854559021 | 0.521431325 | 0.563059245 | 0.482513188 | 0.610870733 | 0.595870413 | 0.547343383 | 0.908601637 |
| GSM518826_treat | 0.472165194 | 0.484214314 | 0.857253434 | 0.866144244 | 0.534390997 | 0.616189033 | 0.485478506 | 0.58252794 | 0.594767136 | 0.494318353 | 0.898067071 |
| GSM518827_treat | 0.555700817 | 0.473776741 | 0.822563471 | 0.858482009 | 0.520478045 | 0.590843113 | 0.513168651 | 0.570550427 | 0.529605268 | 0.518503783 | 0.941573838 |
| GSM518828_treat | 0.385820458 | 0.462853104 | 0.818002701 | 0.888118761 | 0.557683407 | 0.618233531 | 0.418169285 | 0.572848355 | 0.531189842 | 0.478905222 | 0.914766168 |
| GSM518829_treat | 0.584841386 | 0.512270367 | 0.84387581 | 0.852557593 | 0.542303301 | 0.567718414 | 0.502042109 | 0.563252149 | 0.568375477 | 0.519237452 | 0.906667159 |
| GSM518830_treat | 0.641363057 | 0.461067962 | 0.840932683 | 0.838631897 | 0.506920047 | 0.640929727 | 0.416939494 | 0.582602474 | 0.641473238 | 0.514270171 | 0.926013247 |
| GSM518831_treat | 0.594268232 | 0.502322685 | 0.85857987 | 0.835343746 | 0.536149073 | 0.636875054 | 0.409878429 | 0.590259297 | 0.573534748 | 0.488232507 | 0.920172045 |
| GSM518832_treat | 0.466185173 | 0.409537984 | 0.837411717 | 0.904179301 | 0.521842215 | 0.527509466 | 0.493284898 | 0.580409874 | 0.553634601 | 0.526158311 | 0.914546756 |
| GSM518885_treat | 0.556441469 | 0.408274723 | 0.760687587 | 0.859238977 | 0.553201335 | 0.619829815 | 0.504125341 | 0.582523017 | 0.576643154 | 0.514144573 | 0.911497809 |
| GSM518887_treat | 0.544243509 | 0.470472274 | 0.814386909 | 0.858712168 | 0.536724435 | 0.637171303 | 0.469536667 | 0.552860119 | 0.578004834 | 0.520543738 | 0.915168 |
| GSM518889_treat | 0.549929811 | 0.558696418 | 0.876447641 | 0.835598698 | 0.512366512 | 0.547950612 | 0.472216916 | 0.60860811 | 0.561178089 | 0.515415998 | 0.928424861 |
| GSM518891_treat | 0.528430613 | 0.466485439 | 0.821197778 | 0.879635087 | 0.535004708 | 0.666872061 | 0.46951101 | 0.5840212 | 0.531861762 | 0.505571388 | 0.908574788 |
| GSM518893_treat | 0.537180762 | 0.488115704 | 0.852070588 | 0.859546496 | 0.539792824 | 0.610281316 | 0.463406057 | 0.554668536 | 0.607889442 | 0.529970284 | 0.906083797 |
| GSM518895_treat | 0.500165974 | 0.521775534 | 0.867016028 | 0.852856089 | 0.524636671 | 0.59450876 | 0.46247147 | 0.586274161 | 0.512631897 | 0.4957473 | 0.900579225 |
| GSM518897_treat | 0.684871685 | 0.452676757 | 0.851112516 | 0.853305171 | 0.54209928 | 0.584156965 | 0.483920565 | 0.555281181 | 0.624027906 | 0.51842933 | 0.908784861 |
| GSM518899_treat | 0.513331093 | 0.47519288 | 0.852246847 | 0.850462983 | 0.5336287 | 0.597832508 | 0.541542741 | 0.592151063 | 0.629914156 | 0.544544487 | 0.931614023 |
| GSM518901_treat | 0.623662925 | 0.497237317 | 0.875163074 | 0.845368835 | 0.507356541 | 0.603642832 | 0.48595364 | 0.583139276 | 0.583800108 | 0.550606385 | 0.918978908 |
| GSM518903_treat | 0.53601066 | 0.548364279 | 0.870740881 | 0.840902736 | 0.550438335 | 0.578640784 | 0.483627966 | 0.593641025 | 0.643278544 | 0.522127111 | 0.90825367 |
| GSM518905_treat | 0.526143613 | 0.546598972 | 0.868136347 | 0.850960119 | 0.544623353 | 0.580840896 | 0.449770655 | 0.59700055 | 0.521735127 | 0.491161398 | 0.910337352 |
| GSM518907_treat | 0.544127177 | 0.467459109 | 0.82612046 | 0.852541345 | 0.536849681 | 0.634014122 | 0.424208239 | 0.562900665 | 0.578978529 | 0.476327881 | 0.913113092 |
| GSM518909_treat | 0.48852362 | 0.418028325 | 0.828952043 | 0.849508821 | 0.522872475 | 0.61674005 | 0.519711299 | 0.56441785 | 0.577607977 | 0.529570769 | 0.919321658 |
| GSM518911_treat | 0.418549305 | 0.433747178 | 0.805373272 | 0.851670492 | 0.569792934 | 0.617495264 | 0.486352375 | 0.581887157 | 0.527431876 | 0.535974533 | 0.917538433 |
| GSM518913_treat | 0.475051374 | 0.540371733 | 0.860416093 | 0.813646377 | 0.551320191 | 0.582373927 | 0.541724246 | 0.565450332 | 0.476808564 | 0.519197053 | 0.898040103 |
| GSM518915_treat | 0.447987327 | 0.47694573 | 0.834322093 | 0.863750599 | 0.558759358 | 0.609336415 | 0.473779501 | 0.590148575 | 0.625622261 | 0.528665998 | 0.911587306 |
| GSM518917_treat | 0.456250252 | 0.495301274 | 0.86654342 | 0.840818236 | 0.571511439 | 0.611528961 | 0.465716274 | 0.591291533 | 0.573299302 | 0.511633305 | 0.9169621 |
| GSM518919_treat | 0.534952803 | 0.468993872 | 0.846038199 | 0.831962608 | 0.545869431 | 0.653006202 | 0.437270342 | 0.570804535 | 0.566468165 | 0.49493219 | 0.871699458 |
| GSM518921_treat | 0.650994956 | 0.508271013 | 0.851781586 | 0.822121447 | 0.584796412 | 0.636195229 | 0.484851186 | 0.569286067 | 0.610457787 | 0.544177786 | 0.905303013 |
| GSM518923_treat | 0.552510071 | 0.445434662 | 0.802760817 | 0.846985089 | 0.501478273 | 0.66284421 | 0.393396969 | 0.59755449 | 0.542923219 | 0.464159662 | 0.891767867 |
| GSM518925_treat | 0.547931679 | 0.44968305 | 0.835214718 | 0.84759194 | 0.544175662 | 0.602914256 | 0.510629868 | 0.596509862 | 0.600763245 | 0.520197429 | 0.898200416 |
| GSM518927_treat | 0.709581632 | 0.527284834 | 0.850456512 | 0.824958431 | 0.510103483 | 0.712521985 | 0.430744011 | 0.590336938 | 0.539839578 | 0.462308879 | 0.907346488 |
| GSM518929_treat | 0.594536845 | 0.487951395 | 0.829096419 | 0.844814205 | 0.510133403 | 0.617859439 | 0.478251534 | 0.542351917 | 0.579377323 | 0.497728882 | 0.889112563 |
| GSM518931_treat | 0.521825035 | 0.483742251 | 0.861146856 | 0.854585302 | 0.540942456 | 0.695584766 | 0.467254303 | 0.562975349 | 0.522532696 | 0.494214622 | 0.902479184 |
| GSM518933_treat | 0.483426087 | 0.53127617 | 0.876608961 | 0.886782424 | 0.533343461 | 0.548307187 | 0.46541436 | 0.620544011 | 0.622047747 | 0.531434364 | 0.937975783 |
| GSM518935_treat | 0.550521976 | 0.497561814 | 0.820930369 | 0.886171426 | 0.564328061 | 0.608368523 | 0.421136136 | 0.565400846 | 0.598866394 | 0.529808624 | 0.92589515 |
| GSM518937_treat | 0.622844795 | 0.407077025 | 0.764422455 | 0.862190959 | 0.550415135 | 0.602591577 | 0.446939127 | 0.608082215 | 0.604889796 | 0.520681732 | 0.927993434 |
| GSM518939_treat | 0.576689542 | 0.48700161 | 0.82456105 | 0.862050607 | 0.563500551 | 0.6306642 | 0.475718293 | 0.609437211 | 0.669918215 | 0.507023002 | 0.920455713 |
| GSM518941_treat | 0.457909122 | 0.463777344 | 0.817910265 | 0.866298395 | 0.544322731 | 0.611932694 | 0.399753905 | 0.554502775 | 0.579157349 | 0.518922486 | 0.921236274 |
| GSM518943_treat | 0.463904258 | 0.471865494 | 0.779292873 | 0.872412861 | 0.523844722 | 0.57290974 | 0.439217571 | 0.586102001 | 0.582514265 | 0.537742058 | 0.909545236 |
| GSM518945_treat | 0.698916415 | 0.594151307 | 0.860860331 | 0.814468998 | 0.544461352 | 0.548991677 | 0.459364327 | 0.600933316 | 0.578995299 | 0.545272344 | 0.914099181 |
| GSM518947_treat | 0.468724957 | 0.490836972 | 0.797585002 | 0.873575175 | 0.54471409 | 0.524309645 | 0.496810072 | 0.615795405 | 0.567274052 | 0.566720155 | 0.92472111 |
| GSM518949_treat | 0.479623441 | 0.520178508 | 0.830833557 | 0.869617942 | 0.513914244 | 0.542147126 | 0.42415196 | 0.591020542 | 0.517034436 | 0.553186077 | 0.923746397 |
| GSM518951_treat | 0.448231804 | 0.489902894 | 0.804516383 | 0.850799937 | 0.521408914 | 0.562396793 | 0.465327141 | 0.578500628 | 0.560955843 | 0.517024454 | 0.890932543 |
| GSM518953_treat | 0.48929051 | 0.484009626 | 0.872645875 | 0.858240399 | 0.524843867 | 0.617175878 | 0.485116474 | 0.601177445 | 0.529461172 | 0.541663114 | 0.914944044 |
| GSM518955_treat | 0.470489516 | 0.487259607 | 0.850409194 | 0.852095508 | 0.550462117 | 0.554800468 | 0.464380779 | 0.599141986 | 0.559137533 | 0.553900176 | 0.935114506 |
| GSM518957_treat | 0.537603018 | 0.480990232 | 0.785294187 | 0.87084061 | 0.526519143 | 0.60161561 | 0.422069703 | 0.599183682 | 0.605196471 | 0.543431564 | 0.897677094 |
| GSM518959_treat | 0.625037659 | 0.568185357 | 0.84910511 | 0.871673511 | 0.552920753 | 0.524916446 | 0.426819172 | 0.606059441 | 0.58261489 | 0.579998074 | 0.902853362 |
| GSM518961_treat | 0.43965381 | 0.444198829 | 0.822833853 | 0.901323677 | 0.557865711 | 0.604804522 | 0.438684087 | 0.593282778 | 0.538035969 | 0.527280243 | 0.919431736 |
| GSM518963_treat | 0.707752124 | 0.574695672 | 0.879282691 | 0.824855586 | 0.540459038 | 0.618287623 | 0.417457551 | 0.565622417 | 0.675927376 | 0.492272902 | 0.92208367 |
| GSM518965_treat | 0.594037809 | 0.523966466 | 0.868193727 | 0.837354505 | 0.53051054 | 0.519282135 | 0.458160439 | 0.601883596 | 0.603315263 | 0.54465302 | 0.903220342 |
| GSM518967_treat | 0.597440119 | 0.497180464 | 0.823531793 | 0.901477583 | 0.518283627 | 0.543034632 | 0.449873277 | 0.593106889 | 0.644527481 | 0.537157451 | 0.925377662 |
| GSM518969_treat | 0.513264119 | 0.58472759 | 0.860928811 | 0.861780298 | 0.547891462 | 0.57754258 | 0.467091797 | 0.62416533 | 0.619185135 | 0.553660578 | 0.922065242 |
| GSM518971_treat | 0.493552295 | 0.538093731 | 0.86822205 | 0.880012922 | 0.577811911 | 0.54839345 | 0.472867471 | 0.614695668 | 0.666896497 | 0.511260776 | 0.921349327 |
| GSM518973_treat | 0.604381222 | 0.505509548 | 0.834261082 | 0.830608861 | 0.516706374 | 0.663312681 | 0.436285622 | 0.57579618 | 0.574792776 | 0.499440234 | 0.927911138 |
| GSM518975_treat | 0.544656033 | 0.587797736 | 0.874000832 | 0.869508561 | 0.53649551 | 0.588244243 | 0.461992673 | 0.57825376 | 0.558921985 | 0.549093582 | 0.933202087 |
| GSM518977_treat | 0.38421233 | 0.440070236 | 0.795665456 | 0.867776541 | 0.534625502 | 0.545341386 | 0.488868573 | 0.590076206 | 0.497461664 | 0.540201002 | 0.921336102 |
| GSM518979_treat | 0.700939426 | 0.500810656 | 0.820349962 | 0.886972342 | 0.529508061 | 0.588459825 | 0.481856129 | 0.563480913 | 0.59302593 | 0.525093767 | 0.938448439 |
| GSM518981_treat | 0.526212193 | 0.398962193 | 0.737835102 | 0.867011706 | 0.52232669 | 0.546620952 | 0.481181096 | 0.578226372 | 0.56538796 | 0.515817921 | 0.912781173 |
| GSM518983_treat | 0.404678219 | 0.42172272 | 0.79355976 | 0.88423703 | 0.504297057 | 0.548173581 | 0.443527299 | 0.585562732 | 0.61015437 | 0.531078421 | 0.90727386 |
| GSM518985_treat | 0.622849696 | 0.568694671 | 0.862200357 | 0.825665441 | 0.522426655 | 0.67586551 | 0.409672072 | 0.536297653 | 0.518690612 | 0.49380864 | 0.92904208 |
| GSM518987_treat | 0.494695052 | 0.555017967 | 0.870209287 | 0.872300468 | 0.540258835 | 0.618143583 | 0.411555272 | 0.587707722 | 0.616685889 | 0.506593198 | 0.926676128 |
| GSM518989_treat | 0.63139786 | 0.494342269 | 0.843282549 | 0.849101951 | 0.524801985 | 0.637430481 | 0.466956407 | 0.586368552 | 0.59878814 | 0.510551428 | 0.893585103 |
| GSM518991_treat | 0.475610093 | 0.48505171 | 0.806495157 | 0.871465983 | 0.536792273 | 0.631520541 | 0.450269359 | 0.572205851 | 0.61115693 | 0.481499485 | 0.936567059 |
| GSM518993_treat | 0.613263574 | 0.453257022 | 0.775114392 | 0.849461882 | 0.50401988 | 0.620966441 | 0.480761002 | 0.560612658 | 0.534525384 | 0.513942026 | 0.90272057 |
| GSM518995_treat | 0.445684743 | 0.424359074 | 0.823880481 | 0.846311441 | 0.540810074 | 0.657982155 | 0.470821604 | 0.62978685 | 0.565744167 | 0.527101724 | 0.920402131 |
| GSM518997_treat | 0.480579249 | 0.483805348 | 0.823801949 | 0.84275631 | 0.527475627 | 0.668156328 | 0.478168762 | 0.606125405 | 0.577864144 | 0.495709813 | 0.894456649 |
| GSM518999_treat | 0.513281284 | 0.508647349 | 0.878965397 | 0.838567384 | 0.537991348 | 0.592528257 | 0.490545063 | 0.619125247 | 0.59463771 | 0.519076814 | 0.919809276 |
| GSM519001_treat | 0.575323171 | 0.452011434 | 0.775476655 | 0.869544893 | 0.52963781 | 0.593243753 | 0.499428271 | 0.551212943 | 0.558951718 | 0.541073947 | 0.900763722 |
| GSM519003_treat | 0.466221387 | 0.444837258 | 0.821476298 | 0.895162286 | 0.52053326 | 0.617878439 | 0.487253824 | 0.583574775 | 0.552803989 | 0.517132937 | 0.92829413 |
| GSM519005_treat | 0.666549036 | 0.504360537 | 0.806239548 | 0.911852658 | 0.495624341 | 0.579222181 | 0.44726532 | 0.585335228 | 0.599752211 | 0.48560261 | 0.933348575 |
| GSM519007_treat | 0.637930164 | 0.506683567 | 0.851702328 | 0.816801056 | 0.559007388 | 0.642344017 | 0.412052404 | 0.595005581 | 0.580240994 | 0.50742716 | 0.911112888 |
| GSM519009_treat | 0.539188833 | 0.504165618 | 0.85784055 | 0.855665383 | 0.533485112 | 0.571257689 | 0.570238874 | 0.606402752 | 0.590468548 | 0.522638142 | 0.930893142 |
| GSM519011_treat | 0.468717177 | 0.515879048 | 0.87452678 | 0.848823855 | 0.525512652 | 0.556910922 | 0.456433373 | 0.623473419 | 0.624185587 | 0.51168887 | 0.909616749 |
| GSM519013_treat | 0.582461127 | 0.496537936 | 0.869904327 | 0.847303406 | 0.588951696 | 0.694092679 | 0.466577309 | 0.57723436 | 0.618638934 | 0.493360792 | 0.90678658 |
| GSM519015_treat | 0.479343347 | 0.46981579 | 0.847171508 | 0.852799659 | 0.574808362 | 0.645362469 | 0.491585051 | 0.615394649 | 0.514276041 | 0.516093467 | 0.895457738 |
| GSM519017_treat | 0.421790965 | 0.440508702 | 0.833680098 | 0.852520701 | 0.599865846 | 0.574108716 | 0.556340297 | 0.576824661 | 0.576045802 | 0.53142039 | 0.927880126 |
| GSM519019_treat | 0.764691838 | 0.46562035 | 0.861173092 | 0.802202262 | 0.553817412 | 0.71531176 | 0.434143934 | 0.576831947 | 0.585465407 | 0.492270229 | 0.945231165 |
| GSM519021_treat | 0.621309064 | 0.550666479 | 0.858537605 | 0.853483064 | 0.543858456 | 0.591608245 | 0.532486408 | 0.601829988 | 0.617363237 | 0.525317466 | 0.905078436 |
| GSM519023_treat | 0.430048762 | 0.466422732 | 0.852241816 | 0.838667524 | 0.553485479 | 0.557300762 | 0.469285139 | 0.656406028 | 0.596156631 | 0.518156401 | 0.937104905 |
| GSM519025_treat | 0.498529345 | 0.555491804 | 0.891124362 | 0.843339605 | 0.571464003 | 0.536707176 | 0.498574219 | 0.620678994 | 0.503450823 | 0.528076261 | 0.893520578 |
| GSM519027_treat | 0.53524325 | 0.528693773 | 0.840697655 | 0.846934991 | 0.535225315 | 0.514900599 | 0.472517472 | 0.625707837 | 0.609421878 | 0.528411672 | 0.897372879 |
| GSM519029_treat | 0.653549533 | 0.543228743 | 0.843629131 | 0.849744141 | 0.570149858 | 0.625653299 | 0.46449781 | 0.593645488 | 0.670038018 | 0.524494633 | 0.893670163 |
| GSM519031_treat | 0.584688149 | 0.445541394 | 0.800445858 | 0.843446674 | 0.523862574 | 0.655969547 | 0.416926076 | 0.55788555 | 0.597954191 | 0.505083899 | 0.91954086 |
| GSM519033_treat | 0.468194779 | 0.423307486 | 0.742464573 | 0.892360794 | 0.499310299 | 0.660297854 | 0.497190758 | 0.551338004 | 0.510465612 | 0.499340342 | 0.894789145 |
| GSM519035_treat | 0.549280372 | 0.639476401 | 0.90231294 | 0.85199332 | 0.562261975 | 0.529463831 | 0.484430441 | 0.614900879 | 0.580935283 | 0.511415944 | 0.885221099 |
| GSM519037_treat | 0.670161479 | 0.452207069 | 0.803343718 | 0.84343527 | 0.52504313 | 0.660144537 | 0.455534655 | 0.575989273 | 0.68246049 | 0.526819723 | 0.921811751 |
| GSM519039_treat | 0.517351261 | 0.476073405 | 0.839337245 | 0.868677937 | 0.546323861 | 0.614409935 | 0.428748687 | 0.608537044 | 0.59972265 | 0.502538967 | 0.924834425 |
| GSM519041_treat | 0.586411441 | 0.44371563 | 0.848586383 | 0.835037232 | 0.560003169 | 0.648433512 | 0.438248872 | 0.576191799 | 0.618123362 | 0.499376216 | 0.932755025 |
| GSM519043_treat | 0.655423044 | 0.476987773 | 0.822122742 | 0.831300513 | 0.549258487 | 0.602322907 | 0.453258206 | 0.595949368 | 0.565280738 | 0.532235181 | 0.949623469 |
| GSM519045_treat | 0.684402239 | 0.453777278 | 0.814699263 | 0.843126317 | 0.55328861 | 0.644557124 | 0.451525146 | 0.579290426 | 0.60807653 | 0.506654875 | 0.898061351 |
| GSM519047_treat | 0.575336985 | 0.502902721 | 0.81168669 | 0.826914222 | 0.493267811 | 0.664509744 | 0.476097863 | 0.559016956 | 0.49665458 | 0.481948579 | 0.936184129 |
| GSM519049_treat | 0.464389006 | 0.43268799 | 0.810973712 | 0.8614992 | 0.554446805 | 0.669175039 | 0.478013632 | 0.599984848 | 0.540303532 | 0.501988064 | 0.90884211 |
| GSM519051_treat | 0.51841045 | 0.491036026 | 0.886640161 | 0.849172055 | 0.520724595 | 0.597474442 | 0.456055086 | 0.585186581 | 0.560088373 | 0.528915528 | 0.926055575 |
| GSM519053_treat | 0.515706992 | 0.463886863 | 0.831113751 | 0.874909272 | 0.520921659 | 0.615906882 | 0.455116691 | 0.59399584 | 0.600206671 | 0.516419857 | 0.923411046 |
| GSM519055_treat | 0.501247549 | 0.493607715 | 0.847555743 | 0.846881029 | 0.549593386 | 0.631375394 | 0.426477436 | 0.562087782 | 0.537096741 | 0.509253603 | 0.923475412 |
| GSM519057_treat | 0.558713566 | 0.448939598 | 0.818412688 | 0.857170841 | 0.472222063 | 0.572576608 | 0.438377271 | 0.53688603 | 0.517123952 | 0.477179548 | 0.912434235 |
| GSM519059_treat | 0.597818592 | 0.493307269 | 0.890380358 | 0.825682108 | 0.569782366 | 0.607639126 | 0.486078187 | 0.540757928 | 0.563141852 | 0.506875727 | 0.915475457 |
| GSM519061_treat | 0.603844576 | 0.471013048 | 0.840406096 | 0.87594367 | 0.548478269 | 0.698196236 | 0.446261858 | 0.543652608 | 0.503116092 | 0.508723009 | 0.92939178 |
| GSM519063_treat | 0.61897405 | 0.456816904 | 0.827259117 | 0.853307525 | 0.548939409 | 0.648501614 | 0.464189547 | 0.561502645 | 0.570967106 | 0.507650869 | 0.895585074 |
| GSM519065_treat | 0.61164284 | 0.517235049 | 0.855823456 | 0.847033043 | 0.571599291 | 0.613075327 | 0.513265406 | 0.582139255 | 0.636739708 | 0.527624054 | 0.919720051 |
| GSM519067_treat | 0.484600796 | 0.465747098 | 0.835455602 | 0.870146498 | 0.517622109 | 0.720110141 | 0.450015648 | 0.576138981 | 0.630088927 | 0.527012023 | 0.924633217 |
| GSM519069_treat | 0.610169908 | 0.439268727 | 0.834351885 | 0.855351935 | 0.570225035 | 0.704936845 | 0.507435933 | 0.581044882 | 0.653156495 | 0.518712644 | 0.920248703 |
| GSM519071_treat | 0.413394963 | 0.457497855 | 0.847564664 | 0.861645952 | 0.575880501 | 0.545172449 | 0.498418052 | 0.608814405 | 0.502722767 | 0.525078461 | 0.932976409 |
| GSM519073_treat | 0.526266251 | 0.504714134 | 0.849731762 | 0.852866641 | 0.529128514 | 0.680762882 | 0.497056289 | 0.591434364 | 0.511676008 | 0.487173444 | 0.933657603 |
| GSM519075_treat | 0.539199099 | 0.435551612 | 0.757203936 | 0.871624612 | 0.472960462 | 0.645793706 | 0.47834513 | 0.531299774 | 0.524568344 | 0.515809784 | 0.898395601 |
| GSM519077_treat | 0.565233868 | 0.472701081 | 0.81839391 | 0.872772104 | 0.518812728 | 0.668592557 | 0.400276721 | 0.549964398 | 0.527102608 | 0.505753402 | 0.899058938 |
| GSM519079_treat | 0.50770584 | 0.438449673 | 0.834131794 | 0.871620999 | 0.527451794 | 0.614733766 | 0.488746738 | 0.579228367 | 0.623619812 | 0.546098825 | 0.918702455 |
| GSM519081_treat | 0.591093996 | 0.559597517 | 0.850092555 | 0.84221075 | 0.517260446 | 0.603926094 | 0.434823667 | 0.54279426 | 0.599075674 | 0.518619289 | 0.908129657 |

| Macrophage | Mast.cell | Monocyte | Natural.killer.T.cell | Natural.killer.cell | Neutrophil | Plasmacytoid.dendritic.cell | Regulatory.T.cell | T.follicular.helper.cell | Type.1.T.helper.cell | Type.17.T.helper.cell | Type.2.T.helper.cell |
| --- | --- | --- | --- | --- | --- | --- | --- | --- | --- | --- | --- |
| 0.615510196 | 0.529486237 | 0.81379262 | 0.441262734 | 0.502106243 | 0.773456958 | 0.70780879 | 0.442640214 | 0.528447854 | 0.452278626 | 0.363296392 | 0.165696399 |
| 0.621555328 | 0.552277024 | 0.83661173 | 0.440412588 | 0.473903169 | 0.701325557 | 0.731103726 | 0.496484373 | 0.517637777 | 0.498026299 | 0.439662873 | 0.118168366 |
| 0.574288274 | 0.428133771 | 0.830687281 | 0.471917641 | 0.489961546 | 0.547338304 | 0.702669544 | 0.515271556 | 0.55736806 | 0.479233815 | 0.453028349 | 0.083277462 |
| 0.615309111 | 0.542804728 | 0.815866648 | 0.476811831 | 0.473127141 | 0.891343191 | 0.758743975 | 0.515204565 | 0.532246805 | 0.471394554 | 0.397074815 | 0.097102117 |
| 0.6178815 | 0.546907848 | 0.825368573 | 0.466230362 | 0.546388093 | 0.830783379 | 0.721451481 | 0.451711588 | 0.508503545 | 0.474682748 | 0.462603823 | 0.148956254 |
| 0.610565331 | 0.568561102 | 0.801021668 | 0.448868657 | 0.489162603 | 0.799167708 | 0.750163792 | 0.502694822 | 0.50894896 | 0.477031283 | 0.434024878 | 0.142704692 |
| 0.605616905 | 0.475060749 | 0.823843497 | 0.456993226 | 0.51869682 | 0.905496149 | 0.73663407 | 0.480109333 | 0.521828767 | 0.456328032 | 0.419392501 | 0.107155963 |
| 0.626907956 | 0.515688712 | 0.836900219 | 0.46642209 | 0.522770257 | 0.909946446 | 0.775158558 | 0.534850452 | 0.53588275 | 0.482951208 | 0.374935644 | 0.102656377 |
| 0.610328939 | 0.487160351 | 0.806681368 | 0.460089714 | 0.487645943 | 0.813067948 | 0.725865843 | 0.503588853 | 0.533137606 | 0.505859288 | 0.400158472 | 0.126899886 |
| 0.600376013 | 0.483834575 | 0.787602491 | 0.481180767 | 0.561712369 | 0.731414848 | 0.732270776 | 0.461430579 | 0.487486437 | 0.475017332 | 0.394483182 | 0.10795858 |
| 0.624544922 | 0.588777647 | 0.840650501 | 0.442657673 | 0.50269079 | 0.886570779 | 0.743260563 | 0.532209735 | 0.5222641 | 0.476773319 | 0.347908615 | 0.152067755 |
| 0.581727271 | 0.447160279 | 0.80578255 | 0.434765057 | 0.487989086 | 0.801004906 | 0.727436507 | 0.509354581 | 0.54595913 | 0.47506305 | 0.374645641 | 0.088402874 |
| 0.61922868 | 0.54758875 | 0.815786836 | 0.471156484 | 0.540467257 | 0.824866673 | 0.725350592 | 0.537912585 | 0.524346665 | 0.486996521 | 0.406448066 | 0.153023713 |
| 0.604192212 | 0.494625919 | 0.830044968 | 0.461263719 | 0.49431427 | 0.834954464 | 0.73986183 | 0.49090101 | 0.525622199 | 0.461665377 | 0.378545412 | 0.11639851 |
| 0.609120346 | 0.578194856 | 0.810381674 | 0.486994738 | 0.539520165 | 0.846398977 | 0.750422881 | 0.439056784 | 0.513301332 | 0.499438829 | 0.448217316 | 0.115381699 |
| 0.62500517 | 0.518462577 | 0.84254866 | 0.45771708 | 0.512653562 | 0.847774483 | 0.767692614 | 0.500244869 | 0.524530901 | 0.475969125 | 0.363988152 | 0.08685938 |
| 0.576635054 | 0.496578171 | 0.792687209 | 0.445977005 | 0.454858704 | 0.77736654 | 0.706685848 | 0.485123058 | 0.504147385 | 0.47734085 | 0.36787385 | 0.091514393 |
| 0.61812956 | 0.489456723 | 0.832438673 | 0.450340851 | 0.501346081 | 0.907803626 | 0.765531359 | 0.514261312 | 0.528492193 | 0.476288405 | 0.379956843 | 0.111644859 |
| 0.595438459 | 0.517269138 | 0.824886322 | 0.468957126 | 0.507322261 | 0.838331345 | 0.708664997 | 0.500328322 | 0.505326513 | 0.456654285 | 0.387097778 | 0.127359215 |
| 0.635572422 | 0.546700216 | 0.823517122 | 0.507343423 | 0.531498455 | 0.872448822 | 0.752754675 | 0.502002233 | 0.512838611 | 0.431318121 | 0.434222096 | 0.133643403 |
| 0.623168078 | 0.463788842 | 0.830601699 | 0.503736633 | 0.455844007 | 0.787642864 | 0.736837471 | 0.55102018 | 0.569262274 | 0.464228124 | 0.409553321 | 0.123560676 |
| 0.602897525 | 0.566591867 | 0.822529966 | 0.460098085 | 0.525396607 | 0.876312309 | 0.730896083 | 0.541376928 | 0.543709757 | 0.482806679 | 0.371945185 | 0.156387089 |
| 0.610847171 | 0.591726894 | 0.84945183 | 0.464453876 | 0.535907045 | 0.811279925 | 0.749814189 | 0.53149377 | 0.537027126 | 0.49575699 | 0.446664717 | 0.083811694 |
| 0.617207436 | 0.554924302 | 0.841747274 | 0.469852842 | 0.496419022 | 0.800009347 | 0.739523484 | 0.537825361 | 0.504201349 | 0.461894975 | 0.419133717 | 0.080837802 |
| 0.629486117 | 0.586232232 | 0.803005227 | 0.449558245 | 0.484261582 | 0.915675672 | 0.758511394 | 0.538365087 | 0.524455879 | 0.469592422 | 0.451050001 | 0.075435919 |
| 0.578675804 | 0.53717452 | 0.805119678 | 0.479831479 | 0.502490935 | 0.747420252 | 0.722411851 | 0.504198415 | 0.530301157 | 0.476583581 | 0.35465356 | 0.097938076 |
| 0.606961221 | 0.512259299 | 0.798945901 | 0.482145772 | 0.54169193 | 0.866736981 | 0.722631377 | 0.432575674 | 0.542124837 | 0.465112845 | 0.404552645 | 0.158618398 |
| 0.622338774 | 0.580855429 | 0.829061844 | 0.4691731 | 0.527929012 | 0.867478122 | 0.732828248 | 0.529269106 | 0.544457406 | 0.46647659 | 0.428571367 | 0.132911144 |
| 0.619663531 | 0.467458126 | 0.850029347 | 0.458682164 | 0.487183756 | 0.79491807 | 0.739860927 | 0.48363354 | 0.499531825 | 0.471345915 | 0.443977286 | 0.110945024 |
| 0.617840616 | 0.527112963 | 0.831029088 | 0.480277049 | 0.521306386 | 0.824121032 | 0.751949043 | 0.464557731 | 0.512337371 | 0.472169241 | 0.432012779 | 0.131765454 |
| 0.612031121 | 0.506996674 | 0.823466045 | 0.502860639 | 0.486627146 | 0.786407211 | 0.756419993 | 0.528246197 | 0.576761769 | 0.476183904 | 0.367307091 | 0.12795596 |
| 0.637241735 | 0.526439864 | 0.821539917 | 0.462663431 | 0.494530313 | 0.884694795 | 0.722815254 | 0.534540603 | 0.54538109 | 0.458684046 | 0.382934481 | 0.131654797 |
| 0.593919203 | 0.484521515 | 0.81438244 | 0.428160264 | 0.515614965 | 0.83648127 | 0.743909278 | 0.461578589 | 0.521025041 | 0.477421149 | 0.434231878 | 0.133259345 |
| 0.591839603 | 0.47097256 | 0.821181784 | 0.450622536 | 0.524075579 | 0.7688172 | 0.719504486 | 0.513258725 | 0.533027203 | 0.483645742 | 0.3785838 | 0.124340837 |
| 0.529535808 | 0.550744494 | 0.822339353 | 0.461423854 | 0.51947144 | 0.746555721 | 0.727828281 | 0.469872913 | 0.45487688 | 0.485470408 | 0.488630406 | 0.12728574 |
| 0.624921596 | 0.594881139 | 0.82101869 | 0.464880973 | 0.53493269 | 0.770827432 | 0.759316575 | 0.448953353 | 0.544662308 | 0.487439458 | 0.402962784 | 0.118349457 |
| 0.612267281 | 0.591920022 | 0.812878058 | 0.477760572 | 0.51249575 | 0.846895668 | 0.708216539 | 0.489785225 | 0.519600506 | 0.468407424 | 0.448104214 | 0.136412733 |
| 0.626690945 | 0.509232263 | 0.844361457 | 0.506031126 | 0.505922793 | 0.726430079 | 0.765784672 | 0.526313985 | 0.539027336 | 0.489801856 | 0.387324436 | 0.133369883 |
| 0.603318047 | 0.565129287 | 0.827358281 | 0.5100785 | 0.509770774 | 0.873818104 | 0.72479934 | 0.470719589 | 0.518286328 | 0.501955905 | 0.405199777 | 0.101558294 |
| 0.628131895 | 0.514262078 | 0.82880626 | 0.474592345 | 0.483409151 | 0.826752829 | 0.7478401 | 0.522716999 | 0.534661868 | 0.478026706 | 0.370648771 | 0.138497156 |
| 0.620432056 | 0.546642717 | 0.825212356 | 0.448583028 | 0.491020581 | 0.828580076 | 0.736519548 | 0.483061538 | 0.534028716 | 0.466948517 | 0.356721246 | 0.138596764 |
| 0.60758272 | 0.520705201 | 0.822643075 | 0.441776588 | 0.493260745 | 0.849030786 | 0.754588854 | 0.496603493 | 0.53234211 | 0.475651996 | 0.417671632 | 0.128798516 |
| 0.644618964 | 0.509706974 | 0.840807315 | 0.463895491 | 0.479108555 | 0.894542713 | 0.747846894 | 0.482603972 | 0.544181081 | 0.461189582 | 0.393883189 | 0.118052203 |
| 0.635376271 | 0.487517033 | 0.840613036 | 0.466609465 | 0.532457596 | 0.932052294 | 0.767879784 | 0.504933792 | 0.507610674 | 0.492387693 | 0.393250086 | 0.114958295 |
| 0.606982071 | 0.505063672 | 0.834961164 | 0.471750848 | 0.481346927 | 0.901852629 | 0.761917915 | 0.457050258 | 0.511659717 | 0.475791261 | 0.410221202 | 0.136985724 |
| 0.626609985 | 0.550958804 | 0.844589512 | 0.492325083 | 0.49854162 | 0.806254104 | 0.725204905 | 0.480281501 | 0.532008331 | 0.460763403 | 0.39097055 | 0.103470249 |
| 0.630247774 | 0.478361399 | 0.813105427 | 0.469632754 | 0.486550953 | 0.829660098 | 0.740395836 | 0.513935091 | 0.536771636 | 0.466833475 | 0.406088258 | 0.137380552 |
| 0.58744889 | 0.482337963 | 0.806832318 | 0.465900974 | 0.494955234 | 0.752787759 | 0.720358068 | 0.467176971 | 0.495565746 | 0.499931218 | 0.423226391 | 0.169716542 |
| 0.620370902 | 0.497402434 | 0.835905915 | 0.473064095 | 0.516409948 | 0.858470984 | 0.734146031 | 0.50091542 | 0.513266393 | 0.465511912 | 0.379845708 | 0.135908923 |
| 0.607833704 | 0.580009871 | 0.825247584 | 0.449729067 | 0.473531418 | 0.762343132 | 0.740246081 | 0.477848884 | 0.533093742 | 0.488858984 | 0.358906504 | 0.149400238 |
| 0.655432542 | 0.575561953 | 0.832031934 | 0.440626999 | 0.487729731 | 0.833791675 | 0.754329093 | 0.467248704 | 0.541335999 | 0.488027766 | 0.394210023 | 0.146626189 |
| 0.603998432 | 0.451832938 | 0.834410989 | 0.476316816 | 0.493601946 | 0.703400246 | 0.72998097 | 0.456882956 | 0.526658838 | 0.473172521 | 0.412905007 | 0.090217604 |
| 0.602130072 | 0.42088489 | 0.810195528 | 0.469028289 | 0.500307496 | 0.672884029 | 0.746889895 | 0.475255905 | 0.481704859 | 0.482396209 | 0.410164314 | 0.145451507 |
| 0.633159144 | 0.499234216 | 0.83464786 | 0.415509995 | 0.478542966 | 0.873480059 | 0.75658441 | 0.440192083 | 0.531653608 | 0.481819201 | 0.45519721 | 0.099748582 |
| 0.642267504 | 0.536720484 | 0.828246544 | 0.477189336 | 0.442937796 | 0.802419476 | 0.734216194 | 0.496882902 | 0.52642955 | 0.48253026 | 0.391594428 | 0.140823759 |
| 0.585455107 | 0.49313967 | 0.821080966 | 0.443033227 | 0.511221555 | 0.862855091 | 0.75154459 | 0.50413696 | 0.527203375 | 0.489635146 | 0.407834898 | 0.072280257 |
| 0.60637066 | 0.519643493 | 0.827912156 | 0.435914684 | 0.517837067 | 0.778035122 | 0.715329954 | 0.453871507 | 0.572824122 | 0.466736169 | 0.411370322 | 0.162698199 |
| 0.617713635 | 0.507129003 | 0.853122425 | 0.468441538 | 0.50412554 | 0.904904253 | 0.719217283 | 0.45866124 | 0.477626903 | 0.479428119 | 0.407453168 | 0.14210341 |
| 0.634125935 | 0.529546632 | 0.834808856 | 0.478722705 | 0.489669353 | 0.784911898 | 0.736743985 | 0.534524356 | 0.546388932 | 0.475748 | 0.40706455 | 0.113205722 |
| 0.572811137 | 0.482539423 | 0.811759797 | 0.495821053 | 0.523472526 | 0.831928791 | 0.718580112 | 0.472414443 | 0.526979239 | 0.468198588 | 0.456504673 | 0.193001779 |
| 0.604793305 | 0.461286049 | 0.823895203 | 0.487681018 | 0.512480291 | 0.852532505 | 0.729030856 | 0.487986579 | 0.50165539 | 0.468233491 | 0.417317304 | 0.127774432 |
| 0.602417254 | 0.577737937 | 0.836779453 | 0.458717276 | 0.556823175 | 0.763619502 | 0.735130565 | 0.500160597 | 0.533319538 | 0.467517696 | 0.403741599 | 0.089628491 |
| 0.608405486 | 0.525636935 | 0.797732092 | 0.47737362 | 0.503573915 | 0.82428308 | 0.762540013 | 0.497164749 | 0.519415658 | 0.469534713 | 0.386221821 | 0.136583145 |
| 0.566503381 | 0.533568578 | 0.811691992 | 0.490976308 | 0.493453688 | 0.832457248 | 0.740796194 | 0.460088305 | 0.544102328 | 0.462021941 | 0.406528879 | 0.154535615 |
| 0.610260583 | 0.53054361 | 0.813493902 | 0.462367846 | 0.492259498 | 0.76646531 | 0.722946158 | 0.485417488 | 0.550167759 | 0.488259553 | 0.409846692 | 0.106268141 |
| 0.647821529 | 0.499159943 | 0.810040038 | 0.478832762 | 0.519727375 | 0.815534311 | 0.722508421 | 0.478148746 | 0.535878222 | 0.45870594 | 0.468648227 | 0.148738583 |
| 0.623582278 | 0.58406213 | 0.804849189 | 0.459156012 | 0.454022812 | 0.837772914 | 0.728365655 | 0.487242912 | 0.535053829 | 0.473036202 | 0.385815838 | 0.149673382 |
| 0.602023286 | 0.547707008 | 0.824640892 | 0.484845729 | 0.495031554 | 0.889664823 | 0.760414943 | 0.483447136 | 0.538216749 | 0.471749358 | 0.383066278 | 0.150475008 |
| 0.636277454 | 0.463828134 | 0.803563746 | 0.463508289 | 0.46308029 | 0.82751248 | 0.735974746 | 0.445334116 | 0.573838309 | 0.464287886 | 0.427149128 | 0.097628447 |
| 0.586932269 | 0.532300407 | 0.816134366 | 0.46178784 | 0.5138795 | 0.778691862 | 0.729457008 | 0.451030199 | 0.475088685 | 0.479267872 | 0.433435921 | 0.099380446 |
| 0.619342102 | 0.502106441 | 0.803038497 | 0.45551773 | 0.504589076 | 0.91091621 | 0.751763427 | 0.475262696 | 0.522167555 | 0.478234521 | 0.407226185 | 0.12678924 |
| 0.557228101 | 0.592635454 | 0.821240107 | 0.445677715 | 0.487648391 | 0.792854341 | 0.724392565 | 0.456289185 | 0.534638448 | 0.459622798 | 0.385251704 | 0.058609906 |
| 0.613787258 | 0.52707829 | 0.817166304 | 0.454489239 | 0.522828576 | 0.833254183 | 0.735959802 | 0.504267673 | 0.469731658 | 0.483708061 | 0.378407151 | 0.091816535 |
| 0.60573358 | 0.474029151 | 0.843294566 | 0.494916903 | 0.498564524 | 0.761155166 | 0.73789888 | 0.49847421 | 0.567119967 | 0.482290943 | 0.400743134 | 0.119905291 |
| 0.604871182 | 0.591761351 | 0.791724224 | 0.478530204 | 0.467752129 | 0.847081671 | 0.731754411 | 0.466770479 | 0.527702015 | 0.480091387 | 0.39541146 | 0.134446139 |
| 0.562570383 | 0.476307074 | 0.785815048 | 0.472440941 | 0.492862785 | 0.796624736 | 0.741548655 | 0.494126873 | 0.549519246 | 0.468202066 | 0.310154528 | 0.153240352 |
| 0.601725243 | 0.503599012 | 0.846183491 | 0.474014118 | 0.535885999 | 0.950173776 | 0.740504025 | 0.543865186 | 0.535490817 | 0.485322049 | 0.382747288 | 0.135192204 |
| 0.595604858 | 0.461702623 | 0.81147993 | 0.478926702 | 0.506652747 | 0.744060268 | 0.734296793 | 0.507500815 | 0.529857971 | 0.482666711 | 0.419303689 | 0.135153278 |
| 0.601630771 | 0.474091378 | 0.80805413 | 0.464038683 | 0.470299394 | 0.889868332 | 0.765141132 | 0.458852683 | 0.511451515 | 0.444235411 | 0.474368125 | 0.164415855 |
| 0.579378633 | 0.50237254 | 0.829271499 | 0.473424113 | 0.492967515 | 0.721997429 | 0.749217964 | 0.468776073 | 0.481035495 | 0.485065235 | 0.399516337 | 0.124991621 |
| 0.620590203 | 0.527293208 | 0.8301148 | 0.43535232 | 0.520419364 | 0.7328838 | 0.771982225 | 0.488511787 | 0.534949517 | 0.488008115 | 0.417848706 | 0.09444839 |
| 0.630640185 | 0.518748999 | 0.810423276 | 0.480230684 | 0.474447478 | 0.88284749 | 0.761952283 | 0.52438967 | 0.526072323 | 0.480885004 | 0.403538612 | 0.116234133 |
| 0.609212349 | 0.494028322 | 0.846011558 | 0.442469897 | 0.497101768 | 0.684000293 | 0.723859383 | 0.504544684 | 0.513550092 | 0.460269325 | 0.387619444 | 0.105356455 |
| 0.608682933 | 0.54787518 | 0.818983216 | 0.496398944 | 0.501994411 | 0.814500589 | 0.742275875 | 0.450936875 | 0.510942165 | 0.446348338 | 0.434936061 | 0.152453688 |
| 0.61631209 | 0.549173013 | 0.812710153 | 0.447295353 | 0.513684559 | 0.839498748 | 0.782857903 | 0.467676857 | 0.531649027 | 0.482911172 | 0.410970461 | 0.102486957 |
| 0.605204806 | 0.510159288 | 0.801125384 | 0.484406404 | 0.502799532 | 0.787366034 | 0.735019538 | 0.517216898 | 0.522031555 | 0.504475531 | 0.37622676 | 0.043968588 |
| 0.625676374 | 0.504382683 | 0.827903496 | 0.501128172 | 0.495372606 | 0.780657442 | 0.740362945 | 0.517295188 | 0.509739039 | 0.488075128 | 0.401682688 | 0.100986865 |
| 0.644462909 | 0.585189854 | 0.786043426 | 0.480708346 | 0.486929562 | 0.891544955 | 0.731307319 | 0.470924789 | 0.502524916 | 0.456540712 | 0.390171456 | 0.119326505 |
| 0.61520996 | 0.551144626 | 0.78572407 | 0.463426922 | 0.495255714 | 0.771363811 | 0.705633312 | 0.470159819 | 0.52176227 | 0.500393712 | 0.379822933 | 0.120355335 |
| 0.553385566 | 0.478994844 | 0.824706437 | 0.476971555 | 0.521728503 | 0.749310709 | 0.71455213 | 0.488067938 | 0.504592969 | 0.463673596 | 0.396400423 | 0.107440249 |
| 0.619454262 | 0.524370607 | 0.83948556 | 0.492386379 | 0.511474947 | 0.772359819 | 0.714457728 | 0.506454286 | 0.542821407 | 0.449601696 | 0.415185769 | 0.128405799 |
| 0.606465887 | 0.43533792 | 0.821668355 | 0.446299648 | 0.503424397 | 0.794123148 | 0.727354331 | 0.501686362 | 0.526197586 | 0.470893026 | 0.410713513 | 0.115202408 |
| 0.661589795 | 0.622044742 | 0.783258875 | 0.476928385 | 0.518713965 | 0.871386765 | 0.734369197 | 0.514180733 | 0.509866563 | 0.473444129 | 0.406449641 | 0.164303732 |
| 0.626617271 | 0.558732098 | 0.831311365 | 0.484630879 | 0.523301433 | 0.837678824 | 0.747940849 | 0.534334325 | 0.499390246 | 0.51603544 | 0.418422063 | 0.114288751 |
| 0.63332636 | 0.551912515 | 0.808165261 | 0.451331555 | 0.548978186 | 0.808395509 | 0.732509282 | 0.47268628 | 0.518246142 | 0.482478121 | 0.448828651 | 0.08391165 |
| 0.641188593 | 0.559107218 | 0.811211151 | 0.474540567 | 0.503596029 | 0.89033552 | 0.758012892 | 0.515359919 | 0.482960281 | 0.473376094 | 0.477307527 | 0.166083384 |
| 0.591453478 | 0.511887638 | 0.764669603 | 0.475706972 | 0.509290366 | 0.873203953 | 0.761360013 | 0.498224083 | 0.482957549 | 0.494281562 | 0.434426669 | 0.153499846 |
| 0.609972065 | 0.477132553 | 0.80765147 | 0.459993575 | 0.501428517 | 0.790036772 | 0.746999899 | 0.437962305 | 0.509385914 | 0.492430822 | 0.40537575 | 0.123882819 |
| 0.632716984 | 0.512058589 | 0.835332083 | 0.443980016 | 0.481544036 | 0.827868484 | 0.722823478 | 0.496021836 | 0.510859676 | 0.480480329 | 0.362961509 | 0.147770297 |
| 0.605524399 | 0.528766669 | 0.836008523 | 0.447887186 | 0.51315103 | 0.81897468 | 0.716285336 | 0.48040205 | 0.549006482 | 0.482453632 | 0.393732556 | 0.143412151 |
| 0.601496529 | 0.472203244 | 0.823373085 | 0.450417726 | 0.521217001 | 0.813387124 | 0.74390909 | 0.510408056 | 0.530226089 | 0.482598483 | 0.470584139 | 0.08959894 |
| 0.585111248 | 0.558463418 | 0.78281013 | 0.466503098 | 0.509394873 | 0.893170175 | 0.760498912 | 0.462606788 | 0.53986254 | 0.490436212 | 0.420159583 | 0.111067736 |
| 0.602383766 | 0.590429077 | 0.811132258 | 0.489699524 | 0.500937672 | 0.820003175 | 0.730454325 | 0.475413006 | 0.508115396 | 0.48626891 | 0.386336541 | 0.167550722 |
| 0.578405868 | 0.512942922 | 0.816238824 | 0.462855406 | 0.539187084 | 0.817173138 | 0.746045608 | 0.455562905 | 0.537699341 | 0.477644297 | 0.446509051 | 0.129741657 |
| 0.66669497 | 0.527429883 | 0.81816455 | 0.474645392 | 0.49650386 | 0.795474687 | 0.735770892 | 0.47017979 | 0.532409416 | 0.474825347 | 0.3800815 | 0.163150968 |
| 0.627686768 | 0.520317671 | 0.786272383 | 0.450450753 | 0.500583516 | 0.629867866 | 0.703430932 | 0.467165939 | 0.521446713 | 0.494037729 | 0.49288361 | 0.119936724 |
| 0.610962226 | 0.612353897 | 0.819120153 | 0.47027293 | 0.490994954 | 0.7781014 | 0.708988083 | 0.520465662 | 0.504256118 | 0.484172503 | 0.44610617 | 0.203439644 |
| 0.6302596 | 0.596054988 | 0.760141894 | 0.425359159 | 0.53239823 | 0.846306362 | 0.72663059 | 0.50418054 | 0.497077045 | 0.500348354 | 0.407854016 | 0.136742601 |
| 0.606361131 | 0.558241776 | 0.81468503 | 0.483559311 | 0.560132828 | 0.836836172 | 0.756000527 | 0.49474479 | 0.484149473 | 0.48171735 | 0.488226054 | 0.165933155 |
| 0.625535067 | 0.476980809 | 0.810982486 | 0.498622747 | 0.544141483 | 0.823946678 | 0.736211265 | 0.519848565 | 0.538032565 | 0.493299075 | 0.417708542 | 0.13635961 |
| 0.630674557 | 0.517533075 | 0.831788093 | 0.484261648 | 0.510248051 | 0.890163351 | 0.739112712 | 0.526991168 | 0.555931943 | 0.471106405 | 0.386482792 | 0.125970408 |
| 0.605122263 | 0.55534376 | 0.809353194 | 0.441910511 | 0.479711591 | 0.849724251 | 0.738154849 | 0.474676006 | 0.543880228 | 0.491508273 | 0.390047404 | 0.089694958 |
| 0.605752269 | 0.562606428 | 0.82114281 | 0.447414625 | 0.505438794 | 0.796197603 | 0.714026794 | 0.456932673 | 0.530057234 | 0.479230524 | 0.407529432 | 0.145792427 |
| 0.624347555 | 0.524702673 | 0.823875353 | 0.454016416 | 0.49156979 | 0.756446235 | 0.745465665 | 0.511346751 | 0.51054175 | 0.507918079 | 0.418680623 | 0.156648794 |
| 0.638288213 | 0.605287685 | 0.827258478 | 0.435441232 | 0.53325583 | 0.867126717 | 0.762662834 | 0.500319913 | 0.496663005 | 0.50380522 | 0.513453586 | 0.082977467 |
| 0.618257671 | 0.483806288 | 0.816116134 | 0.442935482 | 0.501595395 | 0.837773973 | 0.757737075 | 0.46200153 | 0.543853175 | 0.492695227 | 0.43125316 | 0.109560554 |
| 0.609589448 | 0.518855049 | 0.835360207 | 0.475439437 | 0.51842074 | 0.875346631 | 0.748529117 | 0.503502207 | 0.524812479 | 0.446460174 | 0.426051973 | 0.155565781 |
| 0.6436926 | 0.544892697 | 0.835862442 | 0.477767387 | 0.46271038 | 0.869651095 | 0.752265584 | 0.496060554 | 0.505588337 | 0.440163704 | 0.417658808 | 0.250355384 |
| 0.627988255 | 0.525704211 | 0.838673048 | 0.458051675 | 0.46317336 | 0.837075745 | 0.742300572 | 0.46213033 | 0.523489475 | 0.469065029 | 0.422943533 | 0.13461326 |
| 0.606360342 | 0.515615971 | 0.824900559 | 0.478403678 | 0.491691289 | 0.779907514 | 0.720050827 | 0.469724007 | 0.542585879 | 0.474077122 | 0.422561604 | 0.1155446 |
| 0.58626504 | 0.458380932 | 0.814107614 | 0.45022111 | 0.516866205 | 0.829708865 | 0.744690407 | 0.495707217 | 0.529052794 | 0.502374373 | 0.453654075 | 0.09311741 |
| 0.572656446 | 0.433289831 | 0.825881459 | 0.450761997 | 0.4650975 | 0.743565295 | 0.699181773 | 0.455297786 | 0.541428487 | 0.477233226 | 0.422450176 | 0.120747755 |
| 0.626059347 | 0.546219463 | 0.812825133 | 0.471991904 | 0.52054754 | 0.843453165 | 0.757847553 | 0.486074405 | 0.517916901 | 0.485050843 | 0.476750512 | 0.060435527 |
| 0.649605417 | 0.615140794 | 0.827847182 | 0.46400484 | 0.498911076 | 0.795181512 | 0.749172989 | 0.543676206 | 0.534994288 | 0.46689033 | 0.363082544 | 0.192016095 |
| 0.586633565 | 0.480759436 | 0.815875435 | 0.473314668 | 0.521927799 | 0.823477307 | 0.741859607 | 0.497522397 | 0.52937052 | 0.488592613 | 0.41305693 | 0.135489347 |
| 0.591935798 | 0.536229758 | 0.795760134 | 0.443031266 | 0.541332225 | 0.815647718 | 0.725539017 | 0.494524452 | 0.53204147 | 0.491610407 | 0.406279705 | 0.065318403 |
| 0.605873416 | 0.524145661 | 0.786906672 | 0.459676221 | 0.536276298 | 0.778638431 | 0.730344036 | 0.445988461 | 0.533194992 | 0.513218282 | 0.464491585 | 0.097844589 |
| 0.622878212 | 0.472518993 | 0.860552507 | 0.449687041 | 0.511891591 | 0.791595798 | 0.716616463 | 0.491927275 | 0.51435486 | 0.503375178 | 0.448811307 | 0.10519013 |
| 0.5877901 | 0.490822815 | 0.811800063 | 0.427950132 | 0.565750882 | 0.841181525 | 0.732324846 | 0.419412717 | 0.545052229 | 0.46234103 | 0.44994585 | 0.050610033 |
| 0.676433919 | 0.499762948 | 0.838922952 | 0.475190694 | 0.502660767 | 0.989649917 | 0.77164947 | 0.555474523 | 0.486569498 | 0.455154043 | 0.432918946 | 0.157145677 |
| 0.618044086 | 0.625721957 | 0.833172127 | 0.441080275 | 0.495718967 | 0.837842576 | 0.727229439 | 0.490225607 | 0.530115948 | 0.472892981 | 0.4196657 | 0.145342859 |
| 0.594259239 | 0.507884706 | 0.820125745 | 0.486666976 | 0.554570595 | 0.867129066 | 0.75433566 | 0.485885849 | 0.53977715 | 0.479082318 | 0.40675937 | 0.123217116 |
| 0.625622561 | 0.495669561 | 0.835732959 | 0.50758483 | 0.485939289 | 0.848744658 | 0.740435951 | 0.509001603 | 0.551714045 | 0.457842844 | 0.37379712 | 0.112988927 |
| 0.636266291 | 0.567358844 | 0.823677109 | 0.475046622 | 0.542216742 | 0.878380134 | 0.75623316 | 0.471898913 | 0.558188589 | 0.490717111 | 0.400499452 | 0.098424756 |
| 0.60889371 | 0.488766242 | 0.826168204 | 0.426952104 | 0.464477936 | 0.81618847 | 0.738752576 | 0.483807653 | 0.500955641 | 0.493667963 | 0.44582938 | 0.096703499 |
| 0.609505164 | 0.468825811 | 0.784731638 | 0.48651099 | 0.550278658 | 0.861456542 | 0.724679421 | 0.474912406 | 0.568424259 | 0.511679166 | 0.447827942 | 0.130183205 |
| 0.626472072 | 0.555721466 | 0.806728887 | 0.442365002 | 0.546497544 | 0.927076703 | 0.739189621 | 0.493860817 | 0.487496993 | 0.47829784 | 0.449357494 | 0.063582434 |
| 0.604752345 | 0.574938263 | 0.804927391 | 0.448563563 | 0.472128594 | 0.852610085 | 0.736104065 | 0.49362149 | 0.523437604 | 0.480245862 | 0.404622861 | 0.176748785 |
| 0.600432602 | 0.451565428 | 0.826809002 | 0.443460559 | 0.466507704 | 0.745154165 | 0.719550301 | 0.466669704 | 0.529609205 | 0.494422125 | 0.406739736 | 0.117415915 |
| 0.617130337 | 0.49506714 | 0.825144753 | 0.460401369 | 0.508533396 | 0.869680417 | 0.761642755 | 0.500542585 | 0.518436579 | 0.491560515 | 0.447370639 | 0.144657967 |
| 0.629066127 | 0.552805551 | 0.825216608 | 0.493835257 | 0.499272047 | 0.810850482 | 0.748699454 | 0.557932947 | 0.540800531 | 0.474345569 | 0.447384327 | 0.106831194 |
| 0.564354705 | 0.45457415 | 0.793412464 | 0.48190141 | 0.476618329 | 0.72903797 | 0.716656591 | 0.447727981 | 0.537647549 | 0.460153359 | 0.392726086 | 0.145265319 |
| 0.564892552 | 0.446183107 | 0.78826837 | 0.480572709 | 0.54205764 | 0.805163588 | 0.72324583 | 0.474536752 | 0.547226234 | 0.514999627 | 0.426917339 | 0.119382034 |
| 0.594909257 | 0.445641413 | 0.837259589 | 0.477875316 | 0.468455846 | 0.823162418 | 0.74299894 | 0.417047243 | 0.503854461 | 0.458729008 | 0.413320803 | 0.158295059 |
| 0.608124162 | 0.510853022 | 0.838103299 | 0.478726653 | 0.487110809 | 0.897508416 | 0.772253807 | 0.47336053 | 0.52143007 | 0.445985625 | 0.418515575 | 0.152461048 |
| 0.61322108 | 0.541008947 | 0.809763825 | 0.448882905 | 0.534314931 | 0.842561028 | 0.739145512 | 0.492882739 | 0.515336382 | 0.48673884 | 0.463261238 | 0.14140487 |
| 0.632477304 | 0.553992169 | 0.819581335 | 0.458501321 | 0.517028822 | 0.84945667 | 0.770079433 | 0.502831462 | 0.513715773 | 0.513345433 | 0.462719261 | 0.097738664 |
| 0.615837507 | 0.542084941 | 0.843060514 | 0.440195101 | 0.478665667 | 0.807681824 | 0.718988543 | 0.474129844 | 0.507481556 | 0.474569799 | 0.457080478 | 0.096300027 |
| 0.625565561 | 0.458195594 | 0.803500514 | 0.442598547 | 0.459567598 | 0.719260195 | 0.714542877 | 0.479086172 | 0.499003185 | 0.487217189 | 0.41165391 | 0.131013754 |
| 0.652553983 | 0.57705216 | 0.824803759 | 0.484652582 | 0.5062855 | 0.916968684 | 0.738998112 | 0.494538024 | 0.47449628 | 0.467074731 | 0.378108475 | 0.219348519 |
| 0.629807936 | 0.527911498 | 0.823356946 | 0.42707274 | 0.544414907 | 0.79902873 | 0.70431131 | 0.506443625 | 0.546983317 | 0.475074722 | 0.470501895 | 0.181171835 |
| 0.589936802 | 0.50485819 | 0.81945876 | 0.468842697 | 0.484631731 | 0.837106896 | 0.739450524 | 0.434515556 | 0.561383442 | 0.468668088 | 0.400353388 | 0.063508381 |
| 0.619311935 | 0.430061577 | 0.824587328 | 0.478920008 | 0.477779814 | 0.807583515 | 0.722598151 | 0.490545094 | 0.524552653 | 0.486561216 | 0.428866508 | 0.125230334 |
| 0.69374202 | 0.635691641 | 0.854134132 | 0.461972399 | 0.444901801 | 0.745002398 | 0.703640695 | 0.500605423 | 0.493161262 | 0.470215503 | 0.387032043 | 0.152962602 |
| 0.607692468 | 0.490242265 | 0.803491618 | 0.479860916 | 0.470360623 | 0.821210288 | 0.71013404 | 0.482502641 | 0.53329756 | 0.466926791 | 0.397980265 | 0.108460301 |
| 0.603346603 | 0.5039219 | 0.83719893 | 0.482627889 | 0.507876085 | 0.896313915 | 0.746313564 | 0.470849485 | 0.538904246 | 0.46167057 | 0.425009636 | 0.096955005 |
| 0.551900689 | 0.465076784 | 0.786124998 | 0.455458823 | 0.482301615 | 0.70776039 | 0.71016403 | 0.473561759 | 0.548469512 | 0.469186279 | 0.379414759 | 0.159389454 |
| 0.60903979 | 0.515377508 | 0.810306166 | 0.450586011 | 0.483301319 | 0.735490034 | 0.738658249 | 0.455143314 | 0.501098595 | 0.441163872 | 0.443830356 | 0.085732813 |
| 0.612916754 | 0.457601816 | 0.823642976 | 0.450050752 | 0.514190293 | 0.805526324 | 0.724353446 | 0.479124697 | 0.544265727 | 0.497361266 | 0.453355635 | 0.158880554 |
| 0.615453321 | 0.549478334 | 0.796606836 | 0.472948024 | 0.490498679 | 0.841295021 | 0.729810628 | 0.423409069 | 0.517665498 | 0.477125409 | 0.408822623 | 0.132176314 |
| 0.579090188 | 0.518761071 | 0.803145746 | 0.472949996 | 0.502888265 | 0.82254677 | 0.741520003 | 0.464670765 | 0.531675957 | 0.480760859 | 0.410671159 | 0.122875606 |
| 0.61019396 | 0.44878069 | 0.8221546 | 0.463545804 | 0.510742411 | 0.783113937 | 0.728645674 | 0.494948907 | 0.530664016 | 0.486872297 | 0.377555272 | 0.115332861 |
| 0.611590907 | 0.486127772 | 0.819873332 | 0.445799588 | 0.540606982 | 0.804875797 | 0.746568637 | 0.486631931 | 0.534721266 | 0.498593202 | 0.464667194 | 0.128268477 |
| 0.663224798 | 0.555032192 | 0.849127559 | 0.457964807 | 0.532678757 | 0.943811078 | 0.759819075 | 0.506239208 | 0.533941892 | 0.469023547 | 0.399410086 | 0.113511819 |
| 0.583306108 | 0.462629149 | 0.781430432 | 0.468515822 | 0.504565166 | 0.800385356 | 0.739492722 | 0.456790141 | 0.547895825 | 0.508919292 | 0.418185472 | 0.112218833 |
| 0.621651933 | 0.558880419 | 0.794496003 | 0.468824314 | 0.478885249 | 0.71739462 | 0.732569218 | 0.456673981 | 0.536424218 | 0.478538487 | 0.389167817 | 0.129451489 |
| 0.572712539 | 0.415830844 | 0.811798325 | 0.441359471 | 0.514773917 | 0.770737481 | 0.713763585 | 0.462539118 | 0.508258769 | 0.460440467 | 0.480747529 | 0.171247768 |
| 0.645780996 | 0.590584555 | 0.811090316 | 0.463244941 | 0.509925137 | 0.756720549 | 0.745540089 | 0.49259848 | 0.520154702 | 0.472868181 | 0.450146399 | 0.102251897 |
| 0.587277878 | 0.434978782 | 0.806933172 | 0.459656923 | 0.482362512 | 0.768423699 | 0.736628366 | 0.465957549 | 0.533885534 | 0.486890508 | 0.428963724 | 0.104650705 |
| 0.593885316 | 0.542992218 | 0.801094715 | 0.434404593 | 0.498510932 | 0.759159199 | 0.739066133 | 0.476135149 | 0.512248248 | 0.468314848 | 0.408232548 | 0.131024164 |
| 0.57635091 | 0.474869799 | 0.822272433 | 0.49174237 | 0.524780061 | 0.786516223 | 0.759357467 | 0.463203692 | 0.520203414 | 0.473076892 | 0.404419704 | 0.164669126 |
| 0.629808946 | 0.54818874 | 0.833183602 | 0.478275394 | 0.475114277 | 0.835104034 | 0.704651515 | 0.497631754 | 0.51627174 | 0.455135218 | 0.409893321 | 0.108675326 |
| 0.633701744 | 0.571371726 | 0.816227531 | 0.46382189 | 0.512610441 | 0.834579334 | 0.755453679 | 0.520726301 | 0.484139054 | 0.481637524 | 0.415932239 | 0.113183829 |
| 0.638355421 | 0.534293838 | 0.827677732 | 0.453776999 | 0.500403445 | 0.795990666 | 0.733991139 | 0.446800253 | 0.531677659 | 0.471710594 | 0.440556737 | 0.099844822 |
| 0.573853688 | 0.484521126 | 0.788450986 | 0.461569331 | 0.492072635 | 0.790970316 | 0.758532982 | 0.460801154 | 0.528037155 | 0.506969933 | 0.442400204 | 0.139175286 |
| 0.642391286 | 0.531659548 | 0.78898422 | 0.441025689 | 0.530506829 | 0.81942022 | 0.747251058 | 0.46929767 | 0.54252469 | 0.507017245 | 0.425619463 | 0.143168643 |
| 0.60259462 | 0.479911326 | 0.808777836 | 0.446553511 | 0.534009857 | 0.737630851 | 0.708187097 | 0.500806984 | 0.567306102 | 0.49562415 | 0.441964998 | 0.11981964 |
| 0.600797753 | 0.474142327 | 0.832550759 | 0.4664432 | 0.494988802 | 0.779078805 | 0.742922819 | 0.460641678 | 0.521226047 | 0.470761444 | 0.42029626 | 0.106625707 |
| 0.583012617 | 0.504923621 | 0.817555109 | 0.453429975 | 0.498354901 | 0.877446438 | 0.740977392 | 0.491852843 | 0.526092064 | 0.461339033 | 0.456139822 | 0.108350597 |
| 0.598662162 | 0.466140416 | 0.829575241 | 0.457123792 | 0.511700847 | 0.781643961 | 0.727929406 | 0.501248019 | 0.532384679 | 0.442101443 | 0.433968953 | 0.116795825 |
| 0.603524649 | 0.583395078 | 0.815932254 | 0.438913397 | 0.487759192 | 0.793805596 | 0.717822163 | 0.467465335 | 0.520849267 | 0.474476559 | 0.419504847 | 0.098780498 |
| 0.646337013 | 0.570978249 | 0.779973108 | 0.451482885 | 0.546090699 | 0.790467167 | 0.717944868 | 0.4705328 | 0.536657586 | 0.512760031 | 0.422172149 | 0.092866415 |
| 0.61959727 | 0.544874967 | 0.800267156 | 0.454199555 | 0.510845263 | 0.824723784 | 0.725202386 | 0.502166832 | 0.524539914 | 0.485514395 | 0.367565884 | 0.09163689 |
| 0.55776354 | 0.522175097 | 0.781996106 | 0.433021962 | 0.488186047 | 0.781575014 | 0.733635106 | 0.388676856 | 0.52858192 | 0.501810884 | 0.435157592 | 0.179702891 |
| 0.569745156 | 0.455275532 | 0.775521272 | 0.42470398 | 0.487361878 | 0.63614719 | 0.749514301 | 0.477582265 | 0.552552013 | 0.509890836 | 0.388055689 | 0.152906072 |
| 0.661510324 | 0.623733842 | 0.806573718 | 0.441518329 | 0.515267724 | 0.790985096 | 0.763940977 | 0.461377885 | 0.514406132 | 0.481832983 | 0.382815234 | 0.189923905 |
| 0.669478167 | 0.575504288 | 0.819562032 | 0.483741879 | 0.497017079 | 0.856732096 | 0.73448834 | 0.502437604 | 0.548549537 | 0.481451938 | 0.385878534 | 0.118899397 |
| 0.653493125 | 0.558651751 | 0.791322247 | 0.409169262 | 0.515147041 | 0.864868489 | 0.769042698 | 0.471835135 | 0.500726199 | 0.512072271 | 0.389430972 | 0.108032069 |
| 0.6220583 | 0.50376916 | 0.842659961 | 0.456633038 | 0.498169361 | 0.812734264 | 0.750210924 | 0.528308835 | 0.530110727 | 0.480333191 | 0.392830748 | 0.116880816 |
| 0.664527578 | 0.524858037 | 0.839080207 | 0.497868626 | 0.50147525 | 0.949482231 | 0.772960809 | 0.50011594 | 0.551067498 | 0.466028673 | 0.408635682 | 0.17279331 |
| 0.639909402 | 0.557990106 | 0.819662671 | 0.450600258 | 0.526658213 | 0.790810945 | 0.746385446 | 0.487584021 | 0.506891889 | 0.498903774 | 0.441802634 | 0.130307544 |
| 0.631838235 | 0.486292013 | 0.827925846 | 0.468882488 | 0.492632279 | 0.839977861 | 0.749420313 | 0.497439961 | 0.549554099 | 0.495012052 | 0.408550478 | 0.103919964 |
| 0.614647629 | 0.416186469 | 0.803811159 | 0.503570919 | 0.52584844 | 0.796047147 | 0.72561947 | 0.540423568 | 0.533416462 | 0.451101196 | 0.42716437 | 0.173242468 |
| 0.651270129 | 0.563915079 | 0.841868947 | 0.474421777 | 0.525526021 | 0.807550955 | 0.749476465 | 0.468708364 | 0.509938048 | 0.476428879 | 0.404082162 | 0.15456472 |
| 0.627330833 | 0.473611527 | 0.820399713 | 0.472000329 | 0.515519065 | 0.813321971 | 0.747830584 | 0.504701483 | 0.557078124 | 0.481117204 | 0.46070831 | 0.184993024 |
| 0.660901731 | 0.537441006 | 0.831023973 | 0.455222136 | 0.505390593 | 0.91193763 | 0.750684746 | 0.521698337 | 0.540402552 | 0.471886205 | 0.454412534 | 0.110462854 |
| 0.594575871 | 0.466796188 | 0.818621692 | 0.440464949 | 0.498497231 | 0.771331942 | 0.716114506 | 0.470874115 | 0.540592952 | 0.461191533 | 0.385947293 | 0.183546352 |
| 0.617485494 | 0.569486533 | 0.784331025 | 0.424058612 | 0.519026057 | 0.807228708 | 0.710408406 | 0.444741618 | 0.48817748 | 0.497575411 | 0.427721923 | 0.093786473 |
| 0.624038256 | 0.546625823 | 0.82947424 | 0.495069453 | 0.508579939 | 0.75970143 | 0.758018882 | 0.511453363 | 0.508823766 | 0.481632723 | 0.428571211 | 0.197852073 |
| 0.579353396 | 0.436640927 | 0.818851214 | 0.468162718 | 0.492314669 | 0.76205042 | 0.740011844 | 0.48634648 | 0.536633725 | 0.498030877 | 0.435011747 | 0.13444437 |
| 0.576246977 | 0.428859467 | 0.809372229 | 0.491667452 | 0.475601249 | 0.757388282 | 0.710128822 | 0.440231196 | 0.562548601 | 0.484045118 | 0.407034066 | 0.152781061 |
| 0.631067314 | 0.583354823 | 0.819402821 | 0.45439663 | 0.525680631 | 0.815119821 | 0.764335725 | 0.467003194 | 0.515488818 | 0.468678291 | 0.465688255 | 0.205424291 |
| 0.626970444 | 0.45501394 | 0.832736706 | 0.458639525 | 0.519556163 | 0.841485427 | 0.752817531 | 0.51203318 | 0.485299844 | 0.46861673 | 0.399110143 | 0.17950951 |
| 0.628897335 | 0.502078936 | 0.802741637 | 0.477404156 | 0.511674236 | 0.991645212 | 0.768138442 | 0.450639103 | 0.501949317 | 0.46442623 | 0.448525795 | 0.192565879 |
| 0.631062929 | 0.559564816 | 0.833247931 | 0.475222476 | 0.486089654 | 0.766379119 | 0.759487301 | 0.523408279 | 0.523671122 | 0.472932534 | 0.431101873 | 0.188508078 |
| 0.610226478 | 0.549934782 | 0.830631795 | 0.441254179 | 0.516769765 | 0.84280112 | 0.748969458 | 0.514294675 | 0.508399287 | 0.476208051 | 0.427590534 | 0.191683524 |
| 0.620706791 | 0.545336216 | 0.828011512 | 0.479590402 | 0.508182737 | 0.888536044 | 0.761521703 | 0.485022791 | 0.49143073 | 0.475602254 | 0.442331967 | 0.155593188 |
| 0.641017697 | 0.555620196 | 0.828707186 | 0.511375238 | 0.515082488 | 0.787448951 | 0.752682093 | 0.493790057 | 0.498008481 | 0.454808853 | 0.389125241 | 0.170363518 |
| 0.572641763 | 0.415066084 | 0.823250425 | 0.495082744 | 0.491484489 | 0.734348716 | 0.754979852 | 0.494652906 | 0.556594174 | 0.474393915 | 0.412447591 | 0.136307778 |
| 0.640864694 | 0.544312764 | 0.82225656 | 0.474671523 | 0.496400958 | 0.796419496 | 0.765016476 | 0.470405292 | 0.492819634 | 0.468710155 | 0.428979925 | 0.182673532 |
| 0.579034401 | 0.529982711 | 0.80762661 | 0.447362522 | 0.51732075 | 0.859328727 | 0.758648182 | 0.50228357 | 0.530532634 | 0.510030196 | 0.494264112 | 0.115013288 |
| 0.614232309 | 0.486365024 | 0.825049847 | 0.460039262 | 0.507763864 | 0.70561868 | 0.727740145 | 0.486745538 | 0.537464463 | 0.502153389 | 0.414267336 | 0.151688005 |
| 0.611590314 | 0.50857133 | 0.808797774 | 0.46899789 | 0.528432095 | 0.921795549 | 0.778844492 | 0.514154817 | 0.520851696 | 0.484483137 | 0.464559115 | 0.07505583 |
| 0.613607456 | 0.492200515 | 0.835903551 | 0.481305862 | 0.509566922 | 0.799856432 | 0.782816237 | 0.501373243 | 0.518793457 | 0.494140618 | 0.422317388 | 0.120164943 |
| 0.628642118 | 0.561711023 | 0.852865112 | 0.461192355 | 0.500734264 | 0.796963477 | 0.734297565 | 0.56067151 | 0.515779275 | 0.470100918 | 0.456872647 | 0.116088115 |
| 0.609173302 | 0.50055315 | 0.831036001 | 0.482302642 | 0.502253169 | 0.832777256 | 0.744126299 | 0.494526165 | 0.500830794 | 0.481051464 | 0.416756372 | 0.124438149 |
| 0.63050384 | 0.555418286 | 0.811315512 | 0.472287463 | 0.476467525 | 0.736478189 | 0.741820577 | 0.471903524 | 0.52036561 | 0.48241429 | 0.371586252 | 0.130518879 |
| 0.645676164 | 0.55237826 | 0.840805907 | 0.474262106 | 0.491383223 | 0.824936287 | 0.753299763 | 0.498682274 | 0.522557074 | 0.468378559 | 0.406425201 | 0.096511756 |
| 0.626751374 | 0.530350055 | 0.795529844 | 0.432300671 | 0.516265989 | 0.808272092 | 0.701299319 | 0.426679979 | 0.498311374 | 0.497785675 | 0.396983986 | 0.089907325 |
| 0.614536558 | 0.483679747 | 0.847828903 | 0.467256418 | 0.486563585 | 0.868100575 | 0.729671182 | 0.488322041 | 0.51833604 | 0.450146301 | 0.417457992 | 0.118612856 |
| 0.624096595 | 0.486995536 | 0.835792099 | 0.460187303 | 0.527361875 | 0.885556852 | 0.748283184 | 0.540471319 | 0.510320184 | 0.462250594 | 0.384533001 | 0.128575834 |
| 0.623306051 | 0.556641967 | 0.837575186 | 0.499949296 | 0.528617974 | 0.747039206 | 0.742499992 | 0.528130183 | 0.519981885 | 0.487645915 | 0.460717206 | 0.152754516 |
| 0.636053003 | 0.588503028 | 0.851264008 | 0.487512981 | 0.506371121 | 0.827488002 | 0.729420803 | 0.485694838 | 0.524635384 | 0.469149988 | 0.396327516 | 0.095875558 |
| 0.614009807 | 0.505968819 | 0.807905872 | 0.482208585 | 0.548674426 | 0.854868039 | 0.764344471 | 0.458584901 | 0.504106548 | 0.508253152 | 0.417779857 | 0.112898355 |
| 0.568094066 | 0.39425671 | 0.833837066 | 0.465753997 | 0.470132719 | 0.765733335 | 0.728391345 | 0.502121365 | 0.546886025 | 0.474593048 | 0.436878286 | 0.106763147 |
| 0.604689139 | 0.518918521 | 0.838100519 | 0.452249731 | 0.518252672 | 0.901620178 | 0.738863278 | 0.508205382 | 0.50289899 | 0.481756367 | 0.449392155 | 0.144085044 |
| 0.632924116 | 0.491401361 | 0.844734775 | 0.496844225 | 0.500199249 | 0.753000978 | 0.757647198 | 0.533849842 | 0.519999074 | 0.47432065 | 0.445814038 | 0.207539865 |
| 0.657639947 | 0.529913053 | 0.852157165 | 0.491868509 | 0.492773586 | 0.923898095 | 0.723178199 | 0.500066386 | 0.532041826 | 0.45664523 | 0.424867003 | 0.12843074 |
| 0.615299833 | 0.516409085 | 0.787982917 | 0.433564898 | 0.527939625 | 0.790274635 | 0.739738266 | 0.464063733 | 0.536998321 | 0.498379272 | 0.382558029 | 0.123685344 |
| 0.578584308 | 0.50420015 | 0.822170975 | 0.503858406 | 0.508983323 | 0.823219156 | 0.684911564 | 0.468448746 | 0.5318363 | 0.450900336 | 0.420096837 | 0.166596303 |
| 0.597583389 | 0.465913567 | 0.818564918 | 0.468893969 | 0.48698622 | 0.799749725 | 0.719705257 | 0.55266234 | 0.529976425 | 0.492283845 | 0.424402399 | 0.090014828 |
| 0.596793076 | 0.457144693 | 0.831383469 | 0.473037468 | 0.503025977 | 0.905262456 | 0.749034816 | 0.510246362 | 0.538009937 | 0.48902491 | 0.450779471 | 0.105122843 |
| 0.61572703 | 0.6012088 | 0.798716833 | 0.450890574 | 0.502761791 | 0.735881992 | 0.722338037 | 0.462831639 | 0.514570401 | 0.488762841 | 0.426081153 | 0.117609071 |
| 0.590658185 | 0.578895926 | 0.822735305 | 0.485689357 | 0.506098064 | 0.811500784 | 0.770125158 | 0.459297463 | 0.517907965 | 0.464210285 | 0.420412106 | 0.184432053 |
| 0.600858836 | 0.589521835 | 0.808702121 | 0.448696634 | 0.522059994 | 0.825952053 | 0.726332178 | 0.512375108 | 0.524702644 | 0.480190239 | 0.452778623 | 0.13392372 |
| 0.59633655 | 0.54617639 | 0.804492689 | 0.441673525 | 0.512616606 | 0.769703062 | 0.745838514 | 0.498678253 | 0.528199702 | 0.491802563 | 0.395397373 | 0.127734118 |
| 0.601775769 | 0.498386119 | 0.776994376 | 0.440092821 | 0.465981742 | 0.79237033 | 0.729318374 | 0.455164083 | 0.513994724 | 0.488258744 | 0.402896067 | 0.149493429 |
| 0.649134182 | 0.511932298 | 0.832789476 | 0.481301745 | 0.50406388 | 0.865798629 | 0.710109925 | 0.561220699 | 0.540195343 | 0.475447672 | 0.387688126 | 0.109298299 |
| 0.635533419 | 0.546163346 | 0.826799632 | 0.488019973 | 0.483455368 | 0.871831938 | 0.753195093 | 0.510311176 | 0.532371044 | 0.493444157 | 0.391081983 | 0.10210593 |
| 0.649052894 | 0.626371517 | 0.791589316 | 0.431471696 | 0.53733342 | 0.793969959 | 0.714757135 | 0.524976158 | 0.530052606 | 0.459533916 | 0.378399614 | 0.201493192 |
| 0.6361553 | 0.522472786 | 0.838628821 | 0.47708431 | 0.519605447 | 0.857614142 | 0.759437949 | 0.505908144 | 0.531004794 | 0.491595657 | 0.38493522 | 0.049288493 |
| 0.642862716 | 0.522000202 | 0.850677598 | 0.481415146 | 0.507136513 | 0.859348108 | 0.742017886 | 0.525068361 | 0.540545006 | 0.467218603 | 0.434951986 | 0.149371649 |
| 0.641276689 | 0.510197835 | 0.834903902 | 0.477993975 | 0.514107054 | 0.917503337 | 0.751353602 | 0.489714899 | 0.497915351 | 0.492366183 | 0.417308286 | 0.094085229 |
| 0.59582733 | 0.506872674 | 0.843795001 | 0.486395769 | 0.503632072 | 0.812185698 | 0.715588215 | 0.560970226 | 0.511321563 | 0.487802555 | 0.401733943 | 0.138407106 |
| 0.653895071 | 0.64084657 | 0.842873606 | 0.487147628 | 0.515444587 | 0.780033013 | 0.731299178 | 0.471618931 | 0.513483045 | 0.474411756 | 0.435932395 | 0.155619793 |
| 0.662089498 | 0.52176092 | 0.824376262 | 0.477286647 | 0.525927395 | 0.79411974 | 0.727299722 | 0.501745557 | 0.553284332 | 0.487413926 | 0.375053308 | 0.070713637 |
| 0.593002318 | 0.532813986 | 0.79472179 | 0.451986069 | 0.476112218 | 0.792843755 | 0.724974283 | 0.419181888 | 0.519511275 | 0.463043436 | 0.410959431 | 0.118016195 |
| 0.61524392 | 0.523250894 | 0.838980725 | 0.477542081 | 0.501214469 | 0.817484461 | 0.725793512 | 0.506987257 | 0.523270155 | 0.468611511 | 0.444167189 | 0.145138433 |
| 0.578111714 | 0.435643678 | 0.834333801 | 0.493658867 | 0.493814976 | 0.763525856 | 0.742297186 | 0.483276091 | 0.532739227 | 0.468257731 | 0.43188242 | 0.143276312 |
| 0.614867975 | 0.511037884 | 0.816088251 | 0.486380076 | 0.513510421 | 0.789520112 | 0.727279989 | 0.520977339 | 0.527300301 | 0.455530843 | 0.403502824 | 0.126903252 |
| 0.596577285 | 0.498646266 | 0.809354342 | 0.491226419 | 0.503567591 | 0.706821106 | 0.713955387 | 0.522152472 | 0.544158316 | 0.489537856 | 0.453320295 | 0.091496207 |
| 0.597368925 | 0.446334417 | 0.823570446 | 0.468527861 | 0.484490839 | 0.806491943 | 0.707479415 | 0.50159269 | 0.547243941 | 0.48176635 | 0.422561794 | 0.06074641 |
| 0.61792852 | 0.44926433 | 0.829295858 | 0.46990401 | 0.485457661 | 0.831865709 | 0.692548549 | 0.545257292 | 0.555182531 | 0.491984754 | 0.410617503 | 0.073055219 |
| 0.615977736 | 0.453065993 | 0.826741506 | 0.41992597 | 0.50007329 | 0.838554787 | 0.715648865 | 0.517731564 | 0.540523776 | 0.459240658 | 0.434049399 | 0.054964456 |
| 0.688485242 | 0.580236376 | 0.818343343 | 0.464190525 | 0.498982031 | 0.798408924 | 0.729588695 | 0.533198961 | 0.533492545 | 0.46466089 | 0.394024385 | 0.167442137 |
| 0.624168708 | 0.534103695 | 0.841293473 | 0.487840822 | 0.517940325 | 0.873115164 | 0.729879682 | 0.454220382 | 0.526857367 | 0.46467304 | 0.373806435 | 0.119789535 |
| 0.566765389 | 0.410714993 | 0.815735317 | 0.449201425 | 0.476528048 | 0.67271468 | 0.698083281 | 0.445008917 | 0.517844832 | 0.456556872 | 0.436298104 | 0.111514861 |
| 0.600713215 | 0.480274603 | 0.830553455 | 0.496331619 | 0.485595384 | 0.877274578 | 0.73887365 | 0.525100051 | 0.512925877 | 0.46556134 | 0.412526732 | 0.092886224 |
| 0.609792921 | 0.499718494 | 0.807094037 | 0.440903684 | 0.446342258 | 0.777940917 | 0.726373695 | 0.412139353 | 0.558198995 | 0.50344205 | 0.374421836 | 0.088899982 |
| 0.582467553 | 0.496329394 | 0.817270594 | 0.448719709 | 0.516866192 | 0.87278048 | 0.733409214 | 0.474875959 | 0.538993229 | 0.488449468 | 0.453134031 | 0.103764841 |
| 0.602270799 | 0.502814552 | 0.820144144 | 0.462875818 | 0.49791823 | 0.899152529 | 0.710483908 | 0.495914967 | 0.531569305 | 0.479370667 | 0.429611894 | 0.134272139 |
| 0.614298801 | 0.454893105 | 0.808523884 | 0.445642722 | 0.546099874 | 0.878679581 | 0.7167434 | 0.495601423 | 0.554632393 | 0.511483961 | 0.454929047 | 0.079782014 |
| 0.602790519 | 0.505590629 | 0.827953258 | 0.467388218 | 0.510933378 | 0.823344038 | 0.723643104 | 0.458275176 | 0.545676865 | 0.448004205 | 0.424519647 | 0.092954078 |
| 0.591335662 | 0.487230984 | 0.787634952 | 0.452225727 | 0.549021612 | 0.735451301 | 0.663147598 | 0.491226741 | 0.533609199 | 0.437364049 | 0.449851548 | 0.168116905 |
| 0.622918314 | 0.528517516 | 0.834677265 | 0.457988736 | 0.496129596 | 0.83019783 | 0.757669426 | 0.488539201 | 0.520729894 | 0.462673965 | 0.401970493 | 0.064472116 |
| 0.597759468 | 0.517919494 | 0.819960844 | 0.470039275 | 0.518424267 | 0.925752944 | 0.747261863 | 0.491169124 | 0.538867834 | 0.47490159 | 0.401345885 | 0.127390982 |
| 0.583965016 | 0.54886524 | 0.815617635 | 0.480203964 | 0.521451073 | 0.872002925 | 0.752661583 | 0.497297698 | 0.540331827 | 0.480426706 | 0.373599553 | 0.130017492 |
| 0.636040629 | 0.581971779 | 0.819106166 | 0.474954141 | 0.546379105 | 0.81999644 | 0.725820252 | 0.455144145 | 0.543334527 | 0.451198465 | 0.396885782 | 0.178147089 |
| 0.601797986 | 0.549492043 | 0.810419458 | 0.451986874 | 0.514741484 | 0.80054691 | 0.7167915 | 0.480887033 | 0.507246398 | 0.451524716 | 0.517857797 | 0.129807347 |
| 0.631724751 | 0.551907423 | 0.796116092 | 0.44814176 | 0.537975353 | 0.880614493 | 0.730648616 | 0.558405975 | 0.510903345 | 0.477464499 | 0.427014819 | 0.06185622 |
| 0.585230931 | 0.558641911 | 0.81424394 | 0.511155486 | 0.523477113 | 0.810578254 | 0.752257282 | 0.429949411 | 0.538788485 | 0.465151772 | 0.398053296 | 0.182055095 |
| 0.608922697 | 0.561329971 | 0.82623051 | 0.463144744 | 0.503680108 | 0.885353469 | 0.72635932 | 0.486850644 | 0.529400575 | 0.46686591 | 0.391575019 | 0.156599622 |
| 0.6230704 | 0.475909267 | 0.848057035 | 0.454470501 | 0.456754788 | 0.774491829 | 0.733864906 | 0.533259625 | 0.531955325 | 0.476250393 | 0.363214289 | 0.114082384 |
| 0.60131821 | 0.542823245 | 0.818187491 | 0.484304885 | 0.524082913 | 0.844509505 | 0.745985954 | 0.502275729 | 0.551810776 | 0.463981744 | 0.341902622 | 0.136524641 |
| 0.60207229 | 0.511381563 | 0.820248695 | 0.44218016 | 0.478750363 | 0.861892124 | 0.750542844 | 0.506783604 | 0.524183964 | 0.4982051 | 0.438966063 | 0.074589555 |
| 0.620968076 | 0.551741351 | 0.827292345 | 0.469920231 | 0.508771036 | 0.851642106 | 0.748969089 | 0.489624156 | 0.538010568 | 0.468964696 | 0.415720644 | 0.118674238 |
| 0.615318426 | 0.525527189 | 0.846031199 | 0.474595984 | 0.526965265 | 0.759247253 | 0.727783188 | 0.530930996 | 0.538298579 | 0.48029981 | 0.414744338 | 0.157809698 |
| 0.609937643 | 0.532457013 | 0.812364201 | 0.470530626 | 0.508115836 | 0.858338958 | 0.745262629 | 0.51305043 | 0.554311786 | 0.476124242 | 0.352816318 | 0.072025756 |
| 0.630953364 | 0.49297078 | 0.822180473 | 0.447290091 | 0.514119015 | 0.837625876 | 0.739340433 | 0.500868354 | 0.508267155 | 0.482253268 | 0.421893178 | 0.115407758 |
| 0.616765769 | 0.537483011 | 0.840665054 | 0.493660716 | 0.530230576 | 0.927888645 | 0.752602266 | 0.490308064 | 0.533709966 | 0.463584147 | 0.444333234 | 0.112338194 |
| 0.634815178 | 0.511007182 | 0.814821714 | 0.480050027 | 0.517892033 | 0.886272131 | 0.747503859 | 0.469407938 | 0.524286428 | 0.498764634 | 0.491946326 | 0.149043765 |
| 0.615154282 | 0.557903961 | 0.830702293 | 0.474393558 | 0.500564433 | 0.829342191 | 0.75314129 | 0.467002688 | 0.517350387 | 0.492406637 | 0.442615919 | 0.13029675 |
| 0.586069038 | 0.496515303 | 0.812772102 | 0.465246013 | 0.43381388 | 0.842750496 | 0.757737982 | 0.490940956 | 0.526099762 | 0.470121767 | 0.441377539 | 0.093230475 |
| 0.616370904 | 0.629425919 | 0.833003127 | 0.480220889 | 0.503412048 | 0.868869339 | 0.729003173 | 0.468966753 | 0.529997426 | 0.482018957 | 0.423655829 | 0.12669771 |
| 0.585459365 | 0.506160229 | 0.821018653 | 0.455953895 | 0.54289834 | 0.862207836 | 0.75938401 | 0.548670824 | 0.514380507 | 0.470766689 | 0.455387077 | 0.08200288 |
| 0.62448774 | 0.471224085 | 0.796454081 | 0.465109559 | 0.515265091 | 0.772462295 | 0.686005882 | 0.527142823 | 0.529138308 | 0.512017645 | 0.40614764 | 0.18519733 |
| 0.583930667 | 0.43020044 | 0.828669317 | 0.442182615 | 0.479836067 | 0.793761471 | 0.735569711 | 0.48785312 | 0.523633871 | 0.47434848 | 0.38118862 | 0.105137063 |
| 0.607899879 | 0.578598301 | 0.813825443 | 0.434040989 | 0.484120577 | 0.914635872 | 0.741556031 | 0.465626426 | 0.518864297 | 0.486316252 | 0.406798289 | 0.089538619 |
| 0.618729938 | 0.532746477 | 0.835993903 | 0.461714894 | 0.51852286 | 0.889937679 | 0.750675193 | 0.490100823 | 0.514378289 | 0.46917324 | 0.377267868 | 0.12202735 |
| 0.598346649 | 0.482624331 | 0.809602987 | 0.446106402 | 0.515201642 | 0.837875645 | 0.748596125 | 0.455471715 | 0.504742614 | 0.487990589 | 0.40523839 | 0.100026358 |
| 0.63615935 | 0.572559318 | 0.830548868 | 0.47029538 | 0.476743096 | 0.8293748 | 0.759894662 | 0.542033161 | 0.499500618 | 0.493267838 | 0.414364369 | 0.137237012 |
| 0.584652261 | 0.473187532 | 0.833113344 | 0.447472457 | 0.484322013 | 0.840127657 | 0.755684357 | 0.495457451 | 0.511452378 | 0.477959338 | 0.378829894 | 0.115012822 |
| 0.632774377 | 0.525165962 | 0.838462632 | 0.432125143 | 0.500454832 | 0.891991263 | 0.761752442 | 0.509624267 | 0.528656882 | 0.477782343 | 0.397950891 | 0.126541966 |
| 0.58906933 | 0.479226904 | 0.826357631 | 0.465464307 | 0.508864066 | 0.767242254 | 0.743249087 | 0.47467809 | 0.503060823 | 0.516163431 | 0.418924504 | 0.112923535 |
| 0.591945494 | 0.513065027 | 0.824085138 | 0.447807945 | 0.470549177 | 0.635024497 | 0.717692915 | 0.538454557 | 0.524000886 | 0.480080613 | 0.383257822 | 0.101448801 |
| 0.603835374 | 0.520155296 | 0.832023158 | 0.445795567 | 0.508387656 | 0.825199672 | 0.753333685 | 0.511512513 | 0.500971325 | 0.466913201 | 0.391697536 | 0.104570712 |
| 0.646662288 | 0.529389015 | 0.834701804 | 0.452789921 | 0.525687591 | 0.931193785 | 0.753103421 | 0.543685275 | 0.519179536 | 0.461993868 | 0.418779754 | 0.072088431 |
| 0.625415153 | 0.507501076 | 0.840849921 | 0.456644937 | 0.544517423 | 0.81735585 | 0.688726601 | 0.513970726 | 0.538188102 | 0.484371153 | 0.48678033 | 0.177287754 |
| 0.631157905 | 0.543968907 | 0.840479455 | 0.421057168 | 0.510563343 | 0.903250628 | 0.760274773 | 0.454146368 | 0.498622121 | 0.472897415 | 0.406904765 | 0.157503477 |
| 0.562978696 | 0.533581394 | 0.813929209 | 0.476981073 | 0.498370514 | 0.819389577 | 0.743217795 | 0.510323158 | 0.54402589 | 0.476719959 | 0.374316787 | 0.110946803 |
| 0.649737188 | 0.533347077 | 0.835648171 | 0.462511189 | 0.479550867 | 0.821980743 | 0.739922175 | 0.528418532 | 0.537714654 | 0.460867095 | 0.427998503 | 0.104495665 |
| 0.635004676 | 0.506041041 | 0.842906658 | 0.460188587 | 0.482404308 | 0.892254242 | 0.776780068 | 0.525890036 | 0.533153397 | 0.487695878 | 0.413134127 | 0.101308314 |
| 0.621455626 | 0.52655135 | 0.822815181 | 0.46932783 | 0.524562803 | 0.889457256 | 0.753617655 | 0.510938265 | 0.516121664 | 0.483217804 | 0.394496284 | 0.147714933 |
| 0.634168453 | 0.477375167 | 0.813093926 | 0.477027605 | 0.523524554 | 0.750379166 | 0.736918921 | 0.512333747 | 0.54715696 | 0.470296993 | 0.399717337 | 0.169880593 |
| 0.605234331 | 0.494281341 | 0.827654343 | 0.459160418 | 0.504478548 | 0.830512942 | 0.744119434 | 0.49300989 | 0.538881306 | 0.473359482 | 0.468749769 | 0.122148431 |
| 0.62052047 | 0.583690066 | 0.817317356 | 0.460984481 | 0.518552009 | 0.871025681 | 0.744148 | 0.461265155 | 0.526992801 | 0.47969264 | 0.439473761 | 0.116278857 |
| 0.64518466 | 0.526711214 | 0.845773305 | 0.463151613 | 0.50219238 | 0.915639319 | 0.759319268 | 0.501348512 | 0.528081659 | 0.445363462 | 0.404016063 | 0.016935367 |
| 0.630776093 | 0.476673505 | 0.830300202 | 0.432376774 | 0.504831117 | 0.857200302 | 0.755672667 | 0.51510011 | 0.554323811 | 0.497366609 | 0.404826186 | 0.070288033 |
| 0.629123301 | 0.474596301 | 0.855034089 | 0.513635044 | 0.505171747 | 0.870100426 | 0.740128007 | 0.503571558 | 0.562225277 | 0.46703211 | 0.34149911 | 0.090398204 |
| 0.620434223 | 0.482164923 | 0.827453265 | 0.449545169 | 0.483963566 | 0.766785489 | 0.74165406 | 0.509329262 | 0.525472737 | 0.465016543 | 0.406614136 | 0.10928235 |
| 0.635570036 | 0.522950913 | 0.831077121 | 0.462628021 | 0.4991923 | 0.924107307 | 0.732452487 | 0.485986454 | 0.516586308 | 0.472151417 | 0.469242323 | 0.192176257 |
| 0.608763698 | 0.586415555 | 0.816783973 | 0.491680124 | 0.520944204 | 0.824956262 | 0.760625501 | 0.526649589 | 0.520572465 | 0.482804386 | 0.449131422 | 0.141700236 |
| 0.597613074 | 0.451746263 | 0.831556238 | 0.454537148 | 0.510870619 | 0.813105065 | 0.740063278 | 0.535980166 | 0.536083479 | 0.482217393 | 0.365990934 | 0.08218315 |
| 0.643075133 | 0.483723013 | 0.841667783 | 0.444234898 | 0.498898241 | 0.849671339 | 0.742817319 | 0.483598214 | 0.518894635 | 0.491433807 | 0.408441754 | 0.137010632 |
| 0.633094951 | 0.574158092 | 0.828141538 | 0.483255867 | 0.525499951 | 0.904246212 | 0.753782654 | 0.514204139 | 0.541549988 | 0.46690667 | 0.419600972 | 0.158071895 |
| 0.583953745 | 0.429185468 | 0.816742274 | 0.460775001 | 0.467728685 | 0.717816143 | 0.711978388 | 0.535935685 | 0.524962257 | 0.485399702 | 0.39072443 | 0.120605161 |
| 0.628762734 | 0.521330964 | 0.789370907 | 0.459577922 | 0.48236623 | 0.860919744 | 0.759260713 | 0.509002399 | 0.517843997 | 0.464624086 | 0.409188832 | 0.110556189 |
| 0.63478767 | 0.495229082 | 0.836014012 | 0.455797606 | 0.521974421 | 0.855101576 | 0.735467263 | 0.536857777 | 0.532857436 | 0.460573062 | 0.431346132 | 0.175042937 |
| 0.624354883 | 0.468088947 | 0.823763569 | 0.513036397 | 0.48670782 | 0.743891161 | 0.746887211 | 0.491132172 | 0.577656988 | 0.453625252 | 0.38346626 | 0.149611175 |
| 0.667776548 | 0.613420039 | 0.840415996 | 0.44740116 | 0.498968709 | 0.803578636 | 0.733431755 | 0.506478781 | 0.53930862 | 0.447998249 | 0.382484899 | 0.127251652 |
| 0.605587161 | 0.522615749 | 0.803849034 | 0.452918493 | 0.501040572 | 0.72673693 | 0.738374682 | 0.448120304 | 0.549117865 | 0.475071849 | 0.409871335 | 0.108269941 |
| 0.620057516 | 0.493434398 | 0.825207827 | 0.448871749 | 0.470882755 | 0.844485261 | 0.742088033 | 0.469529251 | 0.535817021 | 0.470093463 | 0.429949582 | 0.178524608 |
| 0.60643175 | 0.507794538 | 0.832539532 | 0.439451175 | 0.479663239 | 0.765054054 | 0.704625372 | 0.504631221 | 0.516093273 | 0.460864893 | 0.394290073 | 0.132420974 |
| 0.579798746 | 0.482665081 | 0.832856679 | 0.431007711 | 0.509323961 | 0.78016762 | 0.716585065 | 0.503905018 | 0.554105215 | 0.500716053 | 0.43599204 | 0.129821764 |
| 0.558298437 | 0.468390011 | 0.830927527 | 0.504394888 | 0.501543863 | 0.695628038 | 0.694009134 | 0.526610206 | 0.56315247 | 0.451717943 | 0.391967923 | 0.070107831 |
| 0.626370424 | 0.531082047 | 0.818346513 | 0.447185763 | 0.500985954 | 0.888733603 | 0.778656339 | 0.481461704 | 0.529925198 | 0.467265679 | 0.49887463 | 0.073846929 |
| 0.626254963 | 0.548067216 | 0.800203652 | 0.427112457 | 0.448466907 | 0.763142551 | 0.721478249 | 0.492057985 | 0.519701726 | 0.472161037 | 0.416757243 | 0.090744736 |
| 0.62002342 | 0.462130141 | 0.83746236 | 0.451495178 | 0.519001202 | 0.790625941 | 0.758857864 | 0.545963201 | 0.532560248 | 0.477746481 | 0.439715422 | 0.068248996 |
| 0.63310648 | 0.53477469 | 0.818051645 | 0.510546335 | 0.485904561 | 0.810220676 | 0.720427869 | 0.470847027 | 0.532831071 | 0.459277286 | 0.407693329 | 0.121841351 |
| 0.57795577 | 0.503224712 | 0.781248978 | 0.436202524 | 0.452656713 | 0.804642811 | 0.68221262 | 0.42681048 | 0.492991946 | 0.463683359 | 0.445587607 | 0.087234689 |
| 0.586956989 | 0.550200474 | 0.841475094 | 0.461426208 | 0.485084517 | 0.814716234 | 0.70530962 | 0.519430626 | 0.520532021 | 0.473470518 | 0.452092503 | 0.104591297 |
| 0.588225232 | 0.49903553 | 0.809060479 | 0.471099263 | 0.51956019 | 0.905870184 | 0.760935907 | 0.496817805 | 0.523997867 | 0.488323179 | 0.444076474 | 0.112536278 |
| 0.611184122 | 0.540562697 | 0.829673046 | 0.479801645 | 0.502291995 | 0.847709513 | 0.729804583 | 0.549810161 | 0.532299954 | 0.480041285 | 0.400216237 | 0.094201313 |
| 0.627247549 | 0.528864027 | 0.832159035 | 0.453079838 | 0.508913841 | 0.938398592 | 0.763718516 | 0.4984931 | 0.524874739 | 0.475749146 | 0.364230365 | 0.108565621 |
| 0.603787972 | 0.53975489 | 0.811454846 | 0.463782529 | 0.514769259 | 0.87581172 | 0.73424939 | 0.527620877 | 0.50912599 | 0.478798075 | 0.416305685 | 0.108847664 |
| 0.678685977 | 0.554707038 | 0.809754973 | 0.469352771 | 0.520511167 | 0.873950984 | 0.718994224 | 0.477983163 | 0.486271009 | 0.454806163 | 0.42205784 | 0.137942649 |
| 0.589374368 | 0.558854268 | 0.833165341 | 0.508386502 | 0.519213706 | 0.838242921 | 0.758820148 | 0.387102914 | 0.571326738 | 0.481479 | 0.478305779 | 0.195444368 |
| 0.620350525 | 0.584623215 | 0.833050298 | 0.460858201 | 0.493755301 | 0.836141156 | 0.756946477 | 0.50919503 | 0.532655059 | 0.476414589 | 0.424217655 | 0.149628846 |
| 0.622054202 | 0.469864263 | 0.847494447 | 0.474864358 | 0.524458102 | 0.839066548 | 0.728047677 | 0.522195057 | 0.543545962 | 0.460740983 | 0.39522495 | 0.085875218 |
| 0.631246711 | 0.556394116 | 0.825724645 | 0.489057726 | 0.511937009 | 0.879888147 | 0.722989876 | 0.466674651 | 0.560038094 | 0.484525922 | 0.454961149 | 0.195569903 |
| 0.644471372 | 0.547613147 | 0.81735262 | 0.497465926 | 0.518390866 | 0.904942399 | 0.738124861 | 0.484067526 | 0.543905229 | 0.482134125 | 0.501703951 | 0.091677642 |
| 0.615777992 | 0.494235064 | 0.831711685 | 0.495340848 | 0.516986048 | 0.848717331 | 0.746439121 | 0.526733525 | 0.476057126 | 0.465016849 | 0.442515053 | 0.067807816 |
| 0.584047639 | 0.518921091 | 0.82043067 | 0.504566215 | 0.521900296 | 0.78974166 | 0.729439673 | 0.546137599 | 0.513924361 | 0.463902348 | 0.392638066 | 0.104241361 |
| 0.643860787 | 0.581066665 | 0.785211412 | 0.476078526 | 0.515701736 | 0.868791306 | 0.737298337 | 0.525067829 | 0.494097286 | 0.455387795 | 0.460386572 | 0.134197257 |
| 0.597135053 | 0.457086215 | 0.802835563 | 0.449041466 | 0.545017706 | 0.77432664 | 0.729295408 | 0.526231892 | 0.515665333 | 0.475227445 | 0.482471136 | 0.086489529 |
| 0.597095747 | 0.524990507 | 0.818591971 | 0.44477448 | 0.510247661 | 0.77283115 | 0.737271273 | 0.455906809 | 0.545193841 | 0.478628728 | 0.348443577 | 0.14586933 |
| 0.646203246 | 0.602357377 | 0.806126876 | 0.441638038 | 0.50394841 | 0.92134305 | 0.744706668 | 0.506600042 | 0.503412928 | 0.464163836 | 0.435685217 | 0.105429129 |
| 0.627346225 | 0.550504178 | 0.831348349 | 0.461801549 | 0.505848507 | 0.798070557 | 0.757356438 | 0.514032242 | 0.533060169 | 0.467174756 | 0.394412042 | 0.097220581 |
| 0.595240329 | 0.433026292 | 0.821629272 | 0.426778887 | 0.495298476 | 0.750409937 | 0.739564251 | 0.512643943 | 0.529130244 | 0.494047294 | 0.431534552 | 0.114176383 |
| 0.617279453 | 0.516702843 | 0.808862935 | 0.470445236 | 0.503929425 | 0.871781941 | 0.74185364 | 0.400439364 | 0.501960804 | 0.477237045 | 0.440508385 | 0.103567915 |
| 0.620702142 | 0.558458975 | 0.810479068 | 0.47038524 | 0.460540788 | 0.843255724 | 0.716721688 | 0.49011967 | 0.548822322 | 0.46281624 | 0.385730065 | 0.184450988 |
| 0.598583072 | 0.47327285 | 0.818708828 | 0.509073612 | 0.521450149 | 0.902265959 | 0.724206671 | 0.442200903 | 0.537637961 | 0.46283858 | 0.45658244 | 0.116685244 |
| 0.60480009 | 0.472121106 | 0.818400546 | 0.464811782 | 0.513951968 | 0.873747783 | 0.743769325 | 0.496005415 | 0.522051158 | 0.47936889 | 0.420797614 | 0.071955411 |
| 0.58134516 | 0.48065131 | 0.837616184 | 0.492403348 | 0.493311769 | 0.909844405 | 0.731236821 | 0.521622127 | 0.546944644 | 0.450812439 | 0.365631247 | 0.111294502 |
| 0.555675363 | 0.471173364 | 0.81644152 | 0.458151734 | 0.461421395 | 0.71424427 | 0.72806277 | 0.525514584 | 0.540186236 | 0.472230459 | 0.36216084 | 0.127550453 |
| 0.603824844 | 0.552673446 | 0.811297718 | 0.49954453 | 0.46780106 | 0.882236892 | 0.722054963 | 0.527409274 | 0.507102644 | 0.467711798 | 0.394236982 | 0.147962567 |
| 0.628669515 | 0.545940573 | 0.793579598 | 0.466802577 | 0.520838108 | 0.793090254 | 0.739047513 | 0.4126335 | 0.533684713 | 0.466292322 | 0.494402349 | 0.0748619 |
| 0.611322096 | 0.558614878 | 0.81175359 | 0.479068536 | 0.527074327 | 0.766411026 | 0.774559079 | 0.515437528 | 0.552163777 | 0.472623155 | 0.463742532 | 0.130747398 |
| 0.638855406 | 0.520502928 | 0.826018367 | 0.456374257 | 0.531363633 | 0.878539075 | 0.750746909 | 0.496592284 | 0.505246319 | 0.460557538 | 0.471300961 | 0.145549979 |
| 0.560339118 | 0.577666781 | 0.801150266 | 0.457983879 | 0.498859191 | 0.834683402 | 0.743113083 | 0.458511725 | 0.522514386 | 0.483974167 | 0.430512553 | 0 |
| 0.617689773 | 0.577605295 | 0.846322536 | 0.475171327 | 0.460340211 | 0.903540455 | 0.769063401 | 0.492629474 | 0.510194429 | 0.488535594 | 0.402713741 | 0.085966507 |
| 0.635563917 | 0.489075793 | 0.791530635 | 0.513828767 | 0.534848111 | 0.803651961 | 0.75227711 | 0.50117529 | 0.549958305 | 0.480747973 | 0.376128081 | 0.094921351 |
| 0.611014129 | 0.52584369 | 0.798900243 | 0.444770634 | 0.489237876 | 0.809835958 | 0.731033441 | 0.472562455 | 0.517695533 | 0.492522522 | 0.449011064 | 0.124176644 |
| 0.616539371 | 0.479845962 | 0.821027629 | 0.4441218 | 0.519095844 | 0.847420779 | 0.717412332 | 0.544681617 | 0.524355135 | 0.477838915 | 0.374979444 | 0.09094133 |
| 0.5897564 | 0.546886171 | 0.792499082 | 0.428293646 | 0.50459903 | 0.812379274 | 0.743126338 | 0.483469534 | 0.521191813 | 0.502697152 | 0.405625806 | 0.119497486 |
| 0.571023165 | 0.515463683 | 0.773751548 | 0.484175761 | 0.51912038 | 0.757463044 | 0.737289912 | 0.508094504 | 0.504691033 | 0.485169635 | 0.457238254 | 0.199797224 |
| 0.578535596 | 0.500499358 | 0.804725287 | 0.455036167 | 0.524937786 | 0.755427403 | 0.693523741 | 0.496167251 | 0.50964767 | 0.464135042 | 0.437668526 | 0.147401292 |
| 0.642662477 | 0.595475093 | 0.807945034 | 0.477636402 | 0.503879178 | 0.836297574 | 0.732746838 | 0.451632412 | 0.525080263 | 0.472248136 | 0.471680147 | 0.16484596 |
| 0.597274671 | 0.557166255 | 0.816506919 | 0.466921395 | 0.508699502 | 0.855477487 | 0.735593288 | 0.485630115 | 0.514525979 | 0.45441194 | 0.437156657 | 0.111672312 |
| 0.615765793 | 0.579577997 | 0.770467613 | 0.448158336 | 0.536787813 | 0.856389349 | 0.742934032 | 0.503003466 | 0.477322422 | 0.431824745 | 0.473400256 | 0.088504136 |
| 0.60340356 | 0.549120449 | 0.817779078 | 0.467676267 | 0.535257584 | 0.868043059 | 0.743496311 | 0.520302877 | 0.553487171 | 0.476237625 | 0.425582702 | 0.171364855 |
| 0.634442236 | 0.57942193 | 0.816397955 | 0.436302315 | 0.453464209 | 0.805770712 | 0.73627524 | 0.467409285 | 0.534631067 | 0.485959549 | 0.372359031 | 0.145321486 |
| 0.624413634 | 0.541581685 | 0.812154788 | 0.442475242 | 0.497256763 | 0.842723611 | 0.734482002 | 0.456147091 | 0.515070274 | 0.468824027 | 0.408510857 | 0.064420333 |
| 0.582730624 | 0.536878093 | 0.792117721 | 0.440560273 | 0.538290009 | 0.735933658 | 0.70989452 | 0.462306139 | 0.569626259 | 0.49632523 | 0.4196628 | 0.096274231 |
| 0.618509463 | 0.506979001 | 0.842108699 | 0.477356813 | 0.471005996 | 0.850431146 | 0.752485259 | 0.50057227 | 0.53224798 | 0.487940142 | 0.392602613 | 0.121966132 |
| 0.60659011 | 0.517629998 | 0.819976636 | 0.451101428 | 0.504707558 | 0.821768583 | 0.726545203 | 0.516967886 | 0.539474758 | 0.479685186 | 0.437093391 | 0.1074811 |
| 0.627732171 | 0.462323077 | 0.851854416 | 0.426575203 | 0.513397404 | 0.719915107 | 0.723304683 | 0.519950233 | 0.511441756 | 0.491424764 | 0.445284767 | 0.120757518 |
| 0.606407972 | 0.503434561 | 0.813637108 | 0.486400998 | 0.450303256 | 0.768887182 | 0.761756525 | 0.531878359 | 0.52687221 | 0.477456894 | 0.446620789 | 0.228841056 |
| 0.607193098 | 0.530492314 | 0.848600247 | 0.48869346 | 0.507509921 | 0.843274733 | 0.743050379 | 0.492730582 | 0.53829601 | 0.498008886 | 0.432118543 | 0.094636461 |
| 0.59176012 | 0.442305566 | 0.832494157 | 0.503163383 | 0.505463301 | 0.756675501 | 0.710490163 | 0.519801896 | 0.549014832 | 0.471649789 | 0.356698993 | 0.148088836 |
| 0.611166068 | 0.543568417 | 0.791685517 | 0.440260305 | 0.435849053 | 0.763778814 | 0.718114897 | 0.492043391 | 0.511795244 | 0.472383514 | 0.431071863 | 0.170676189 |
| 0.663665092 | 0.557105893 | 0.83559985 | 0.448119473 | 0.526375417 | 0.871239104 | 0.73006218 | 0.496070108 | 0.52089883 | 0.49038267 | 0.476227032 | 0.080232496 |
| 0.603319233 | 0.504853827 | 0.783180203 | 0.44160606 | 0.545402862 | 0.966904696 | 0.777859532 | 0.456145006 | 0.51332004 | 0.498652477 | 0.431430685 | 0.146386085 |
| 0.592319481 | 0.508194262 | 0.833248744 | 0.460787591 | 0.486333957 | 0.813188334 | 0.739509942 | 0.489000279 | 0.501637587 | 0.493261674 | 0.402907772 | 0.250297588 |
| 0.605709572 | 0.563471619 | 0.821263262 | 0.480161881 | 0.524904241 | 0.819720531 | 0.710089377 | 0.488663965 | 0.537002792 | 0.499576475 | 0.446564204 | 0.1044913 |
| 0.620461337 | 0.526314065 | 0.740697298 | 0.47285883 | 0.479516363 | 0.98899877 | 0.753351756 | 0.440029045 | 0.525749544 | 0.49363744 | 0.336661715 | 0.109323933 |
| 0.543349542 | 0.435338651 | 0.777952469 | 0.451236031 | 0.459073935 | 0.70554617 | 0.697656732 | 0.389316748 | 0.504584753 | 0.466279513 | 0.374088814 | 0.132393093 |
| 0.586068394 | 0.483986151 | 0.790487492 | 0.439447711 | 0.528277132 | 0.731787295 | 0.702862707 | 0.460326466 | 0.540466611 | 0.492983167 | 0.425906327 | 0.081870735 |
| 0.606056228 | 0.502020708 | 0.826147776 | 0.48350703 | 0.50941513 | 0.772924457 | 0.733337488 | 0.514891799 | 0.537068828 | 0.457186126 | 0.34466351 | 0.233915015 |
| 0.607066293 | 0.52636204 | 0.799163885 | 0.43292033 | 0.527489592 | 0.810315027 | 0.715098609 | 0.500987903 | 0.514165094 | 0.475674046 | 0.424230743 | 0.166590521 |
| 0.639848716 | 0.546877911 | 0.837295387 | 0.530637917 | 0.49314478 | 0.79821366 | 0.766972409 | 0.545476908 | 0.563248197 | 0.488976169 | 0.403524288 | 0.148033662 |
| 0.629001186 | 0.519051739 | 0.820796837 | 0.475101243 | 0.527963122 | 0.794728429 | 0.759849986 | 0.504605043 | 0.522407411 | 0.470843545 | 0.404187285 | 0.155181629 |
| 0.588107377 | 0.503656592 | 0.815367493 | 0.519832279 | 0.484351911 | 0.778242369 | 0.74641957 | 0.483308426 | 0.582877933 | 0.493593774 | 0.390432651 | 0.108766317 |
| 0.605739182 | 0.532135469 | 0.822533686 | 0.47330135 | 0.528468055 | 0.865494826 | 0.762376953 | 0.466223546 | 0.545424536 | 0.482516129 | 0.403430977 | 0.119352453 |
| 0.612459079 | 0.561170717 | 0.82186942 | 0.48696556 | 0.512705563 | 0.645677711 | 0.693232194 | 0.532839737 | 0.50165287 | 0.448681564 | 0.391160726 | 0.090721051 |
| 0.573206738 | 0.47389257 | 0.804979309 | 0.440405427 | 0.486245269 | 0.763292748 | 0.738464603 | 0.472482605 | 0.525318362 | 0.501053761 | 0.431750677 | 0.12986104 |
| 0.620961077 | 0.451430093 | 0.836091937 | 0.481126793 | 0.485626442 | 0.782398651 | 0.751991366 | 0.488754618 | 0.531758223 | 0.475923192 | 0.404798317 | 0.113634648 |
| 0.576776855 | 0.464321183 | 0.789941584 | 0.468741405 | 0.517763666 | 0.729461883 | 0.718348003 | 0.461645145 | 0.533400696 | 0.513198648 | 0.453219484 | 0.107712761 |
| 0.580597489 | 0.433092473 | 0.814585304 | 0.473133411 | 0.497582831 | 0.760097287 | 0.706503931 | 0.454262211 | 0.543353951 | 0.451680902 | 0.388055095 | 0.066984136 |
| 0.632073152 | 0.511136105 | 0.846051134 | 0.480475837 | 0.480529499 | 0.821081778 | 0.724374095 | 0.503682112 | 0.522700772 | 0.448137581 | 0.404564249 | 0.155825488 |
| 0.607127255 | 0.564788161 | 0.833128741 | 0.458930231 | 0.493125511 | 0.913795996 | 0.761703206 | 0.490552126 | 0.482227578 | 0.476751349 | 0.438007541 | 0.231090229 |
| 0.650535281 | 0.530989136 | 0.836872202 | 0.453662053 | 0.478543295 | 0.741523638 | 0.72847888 | 0.501848225 | 0.539329492 | 0.467432421 | 0.40774938 | 0.14384515 |
| 0.642812045 | 0.520446334 | 0.817046604 | 0.469048713 | 0.519576681 | 0.772251067 | 0.703694763 | 0.485308628 | 0.527943514 | 0.469686297 | 0.441996544 | 0.106188571 |
| 0.611089666 | 0.500697219 | 0.78302245 | 0.44057857 | 0.525324486 | 0.991224254 | 0.768989066 | 0.506908051 | 0.472705914 | 0.491732883 | 0.467411198 | 0.150786099 |
| 0.592677542 | 0.549904049 | 0.818830807 | 0.466792207 | 0.493873897 | 0.873031421 | 0.75176707 | 0.491139043 | 0.498604927 | 0.463565771 | 0.378255364 | 0.198940745 |
| 0.59288369 | 0.475678918 | 0.815440642 | 0.475210532 | 0.473922108 | 0.767822685 | 0.742330437 | 0.471198737 | 0.525932221 | 0.459986816 | 0.390814284 | 0.138976204 |
| 0.608299835 | 0.499725649 | 0.830488514 | 0.477676834 | 0.525019762 | 0.843487923 | 0.740473663 | 0.515589761 | 0.516413602 | 0.477010242 | 0.389892663 | 0.126250049 |
| 0.588468007 | 0.491340454 | 0.802456185 | 0.439793016 | 0.525913778 | 0.727096815 | 0.693942225 | 0.467339391 | 0.537099035 | 0.502850853 | 0.470218254 | 0.081277952 |
| 0.625818891 | 0.442607953 | 0.819414353 | 0.486617337 | 0.529820551 | 0.81444841 | 0.727687462 | 0.499335742 | 0.511553745 | 0.480293044 | 0.436801392 | 0.100149055 |
| 0.687051553 | 0.582101189 | 0.823190005 | 0.494686716 | 0.512608556 | 0.962143696 | 0.752587739 | 0.575212003 | 0.510466417 | 0.453243728 | 0.420879666 | 0.156069507 |
| 0.642195188 | 0.583974905 | 0.82427177 | 0.460761027 | 0.492161458 | 0.810826139 | 0.724620394 | 0.468327359 | 0.574707506 | 0.477334415 | 0.40486872 | 0.124600327 |
| 0.561880922 | 0.466868381 | 0.77893519 | 0.433367025 | 0.539694596 | 0.705038959 | 0.747739831 | 0.419801475 | 0.514430797 | 0.498915725 | 0.417019437 | 0.07822805 |
| 0.54061485 | 0.397153313 | 0.787024908 | 0.424318469 | 0.449594253 | 0.598549275 | 0.698537751 | 0.428723097 | 0.497185364 | 0.460699337 | 0.401671176 | 0.137041086 |
| 0.619066002 | 0.53445764 | 0.842056782 | 0.468845103 | 0.539706104 | 0.819066223 | 0.731524498 | 0.488028636 | 0.51708475 | 0.48572283 | 0.469034385 | 0.09347852 |
| 0.610749225 | 0.486328096 | 0.827905937 | 0.433135799 | 0.550851694 | 0.880958173 | 0.744605889 | 0.532011958 | 0.524304392 | 0.509477932 | 0.463172523 | 0.097537767 |
| 0.594964274 | 0.436688796 | 0.831799021 | 0.477842434 | 0.466364669 | 0.773838354 | 0.730795891 | 0.488698215 | 0.544833401 | 0.471271712 | 0.421175694 | 0.116146042 |
| 0.658423663 | 0.595046193 | 0.797838115 | 0.461433665 | 0.531641856 | 0.892520676 | 0.752929305 | 0.488276447 | 0.522107539 | 0.464067202 | 0.431641893 | 0.148439406 |
| 0.637318774 | 0.57918738 | 0.824938685 | 0.431082158 | 0.55326994 | 0.900703539 | 0.753162858 | 0.51341687 | 0.499540296 | 0.473026904 | 0.384976296 | 0.10070482 |
| 0.619702991 | 0.541003991 | 0.8320823 | 0.499761335 | 0.514695324 | 0.855109779 | 0.753410002 | 0.494041584 | 0.52709879 | 0.476753013 | 0.381485633 | 0.142884696 |
| 0.577216296 | 0.462631748 | 0.805898776 | 0.492765711 | 0.535760592 | 0.779244541 | 0.730352098 | 0.5226933 | 0.563432266 | 0.500109536 | 0.41670005 | 0.100517292 |
| 0.623951476 | 0.569806731 | 0.826364489 | 0.46968863 | 0.537123216 | 0.897712001 | 0.780866628 | 0.468946524 | 0.542399521 | 0.493720047 | 0.410582244 | 0.132195836 |
| 0.626826864 | 0.533262303 | 0.831468869 | 0.470314667 | 0.503289601 | 0.886849749 | 0.755628754 | 0.528606448 | 0.541286511 | 0.498287788 | 0.419307764 | 0.129771475 |
| 0.702788162 | 0.648999728 | 0.841854023 | 0.486358544 | 0.484894044 | 0.855304821 | 0.744154369 | 0.522776535 | 0.506336882 | 0.475112598 | 0.424739103 | 0.116274272 |
| 0.659144935 | 0.532635355 | 0.849755079 | 0.499905168 | 0.506205081 | 0.937591915 | 0.773709031 | 0.538622283 | 0.538921802 | 0.474092101 | 0.431798347 | 0.096906342 |
| 0.631589264 | 0.584933023 | 0.838772364 | 0.462516124 | 0.514545299 | 0.811814428 | 0.7343583 | 0.497615509 | 0.529271573 | 0.496554646 | 0.480556915 | 0.146991 |
| 0.62937178 | 0.565986016 | 0.838881622 | 0.444530635 | 0.533808992 | 0.879196718 | 0.77889757 | 0.480460423 | 0.48260576 | 0.475583248 | 0.443195806 | 0.179574151 |
| 0.578953443 | 0.487284803 | 0.821573361 | 0.430951296 | 0.538646522 | 0.784179063 | 0.747745886 | 0.490366444 | 0.52448685 | 0.497335622 | 0.424059449 | 0.068319103 |
| 0.637531071 | 0.557032366 | 0.847489499 | 0.461733805 | 0.539750863 | 0.860150571 | 0.774117512 | 0.506852809 | 0.531086378 | 0.501091862 | 0.418184066 | 0.117523235 |
| 0.634582199 | 0.525614197 | 0.833823085 | 0.464608538 | 0.506834239 | 0.867728369 | 0.772627465 | 0.511190804 | 0.532910068 | 0.484112453 | 0.450476553 | 0.115937319 |
| 0.639442756 | 0.577458664 | 0.827277912 | 0.493800743 | 0.521609086 | 0.783634454 | 0.73595705 | 0.512593172 | 0.543374944 | 0.474365167 | 0.452308749 | 0.132092444 |
| 0.619324128 | 0.539429931 | 0.830076753 | 0.523484029 | 0.482128141 | 0.828989637 | 0.723087012 | 0.552284891 | 0.539474034 | 0.457032303 | 0.414240467 | 0.196768093 |
| 0.632020169 | 0.502413633 | 0.834466101 | 0.451895286 | 0.491892146 | 0.810307045 | 0.753252604 | 0.517419917 | 0.507834697 | 0.466669762 | 0.423933579 | 0.114552815 |
| 0.576316486 | 0.46915107 | 0.815248543 | 0.479762577 | 0.505317818 | 0.803194269 | 0.697555802 | 0.481173158 | 0.549754139 | 0.487537859 | 0.418227298 | 0.09204825 |
| 0.616044416 | 0.486741482 | 0.818808258 | 0.450013521 | 0.534517591 | 0.829552433 | 0.761590276 | 0.449682146 | 0.506538164 | 0.485408979 | 0.439518569 | 0.101113785 |
| 0.594211763 | 0.523395147 | 0.784306053 | 0.470323132 | 0.506404068 | 0.902844773 | 0.746997775 | 0.49131776 | 0.487423034 | 0.502039099 | 0.431207413 | 0.162321795 |
| 0.62710823 | 0.556762795 | 0.847008983 | 0.493764748 | 0.479258426 | 0.830302966 | 0.703865689 | 0.51822298 | 0.560904056 | 0.46582846 | 0.417604511 | 0.186976822 |
| 0.640432203 | 0.57398412 | 0.811728904 | 0.488338175 | 0.480416051 | 0.796715096 | 0.708148783 | 0.493007591 | 0.550479481 | 0.447407164 | 0.389219352 | 0.158608828 |
| 0.547502523 | 0.415616902 | 0.783609685 | 0.433634621 | 0.454197176 | 0.629351978 | 0.691766053 | 0.446325024 | 0.527499121 | 0.46379397 | 0.389824939 | 0.126146269 |
| 0.645376784 | 0.520143745 | 0.843666256 | 0.457378213 | 0.504475957 | 0.909991112 | 0.772421983 | 0.537707364 | 0.494314136 | 0.476639015 | 0.430665283 | 0.127310518 |
| 0.630239565 | 0.526612561 | 0.832827841 | 0.475776144 | 0.461370154 | 0.824754917 | 0.766300392 | 0.476023349 | 0.528392039 | 0.468471653 | 0.399742439 | 0.177214641 |
| 0.632662984 | 0.547644658 | 0.828037619 | 0.507309359 | 0.463681762 | 0.865318103 | 0.749449894 | 0.534388746 | 0.561023592 | 0.483190476 | 0.388518016 | 0.161748566 |
| 0.671825311 | 0.607351573 | 0.854099195 | 0.469924365 | 0.487796241 | 0.880410171 | 0.766375942 | 0.504382336 | 0.516127819 | 0.486536952 | 0.443045442 | 0.130576081 |
| 0.649266454 | 0.547429382 | 0.813688857 | 0.490852619 | 0.535298857 | 0.923845883 | 0.737332526 | 0.484488973 | 0.55714336 | 0.46011524 | 0.385305564 | 0.105995825 |
| 0.590958799 | 0.590751244 | 0.803260137 | 0.487101315 | 0.507524282 | 0.710273164 | 0.710913413 | 0.469550129 | 0.505433577 | 0.462474458 | 0.407627899 | 0.172403547 |
| 0.607236014 | 0.486432684 | 0.826822937 | 0.443434241 | 0.496125917 | 0.872700373 | 0.759635464 | 0.488910027 | 0.53689493 | 0.468917588 | 0.417164589 | 0.091107431 |
| 0.614371438 | 0.447270177 | 0.801214735 | 0.44917241 | 0.479821747 | 0.73972305 | 0.65727588 | 0.47578568 | 0.511792657 | 0.466979997 | 0.389117407 | 0.154246871 |
| 0.631863738 | 0.539415286 | 0.832184505 | 0.489760612 | 0.510831754 | 0.796831388 | 0.725905275 | 0.508267597 | 0.534021928 | 0.463194299 | 0.436847795 | 0.099516585 |
| 0.604633191 | 0.521106331 | 0.78966466 | 0.454738201 | 0.53509905 | 0.730004422 | 0.727953203 | 0.476408605 | 0.510789352 | 0.502403691 | 0.411297545 | 0.132228232 |
| 0.62234646 | 0.513786649 | 0.820043727 | 0.506970362 | 0.504423357 | 0.796805736 | 0.734049296 | 0.472189156 | 0.55731634 | 0.450041053 | 0.379981214 | 0.065353264 |
| 0.600463597 | 0.474356372 | 0.832390464 | 0.474461059 | 0.488216926 | 0.860510509 | 0.726585515 | 0.483371342 | 0.515276149 | 0.46322299 | 0.437374645 | 0.166236182 |
| 0.61477073 | 0.464091046 | 0.839575119 | 0.484057056 | 0.48911569 | 0.883633592 | 0.72269228 | 0.509904356 | 0.544066156 | 0.470747524 | 0.399634191 | 0.121631558 |
| 0.610825119 | 0.541455426 | 0.813728189 | 0.475769553 | 0.510241664 | 0.861457118 | 0.747762643 | 0.508937847 | 0.498312451 | 0.4559038 | 0.380605241 | 0.131963516 |
| 0.593782966 | 0.459540566 | 0.839784736 | 0.477862056 | 0.488532513 | 0.827487315 | 0.744930405 | 0.457443402 | 0.531670472 | 0.466886921 | 0.427656125 | 0.105058153 |
| 0.624614434 | 0.518191689 | 0.8324576 | 0.476046001 | 0.495172074 | 0.834570079 | 0.74385822 | 0.495798986 | 0.526633552 | 0.500276198 | 0.42267137 | 0.123884635 |
| 0.615508195 | 0.541222282 | 0.825070733 | 0.458752565 | 0.511525985 | 0.874694133 | 0.76599619 | 0.491903655 | 0.549359696 | 0.487881732 | 0.433088701 | 0.119783864 |
| 0.636689931 | 0.523533671 | 0.804839617 | 0.508873014 | 0.505696697 | 0.761921149 | 0.696886582 | 0.505637544 | 0.548305292 | 0.476699864 | 0.40651249 | 0.093120262 |
| 0.672594124 | 0.57718355 | 0.840321138 | 0.455499279 | 0.461356459 | 0.839701604 | 0.736437428 | 0.517944184 | 0.486904372 | 0.474840738 | 0.42599562 | 0.126321113 |
| 0.59039687 | 0.509585142 | 0.782121658 | 0.446963094 | 0.531653267 | 0.947895069 | 0.747804572 | 0.463115212 | 0.551899815 | 0.47954431 | 0.376984396 | 0.14810239 |
| 0.638798591 | 0.509084158 | 0.81884107 | 0.486446648 | 0.498044512 | 0.894844586 | 0.760463866 | 0.542445062 | 0.54468443 | 0.469886033 | 0.434245599 | 0.229540956 |
| 0.581145329 | 0.451203251 | 0.821992566 | 0.476365345 | 0.515436397 | 0.855791086 | 0.754542076 | 0.531120868 | 0.498358843 | 0.476557 | 0.425834716 | 0.184488833 |
| 0.609534722 | 0.452536125 | 0.806940583 | 0.428990247 | 0.507067976 | 0.802982668 | 0.711082817 | 0.462215509 | 0.533363698 | 0.49609234 | 0.420900276 | 0.054302884 |
| 0.628001166 | 0.566176055 | 0.83764036 | 0.469599449 | 0.514735549 | 0.802238899 | 0.729949081 | 0.51940975 | 0.527167171 | 0.468143446 | 0.472932401 | 0.140633052 |
| 0.603750472 | 0.524945892 | 0.794820912 | 0.463278741 | 0.532813224 | 0.806465781 | 0.7536681 | 0.484114518 | 0.538595403 | 0.528972755 | 0.423642402 | 0.131272096 |
| 0.591382426 | 0.412800627 | 0.814233736 | 0.467022881 | 0.493413043 | 0.884968985 | 0.75144279 | 0.468329358 | 0.524132347 | 0.487184645 | 0.399046921 | 0.152226132 |
| 0.624589103 | 0.549510959 | 0.837872618 | 0.478496038 | 0.489814852 | 0.88809195 | 0.76642456 | 0.502276926 | 0.538295558 | 0.47863209 | 0.407165502 | 0.126910992 |
| 0.622180728 | 0.576215586 | 0.824042414 | 0.501684209 | 0.479875598 | 0.809410346 | 0.738859442 | 0.435890884 | 0.510554678 | 0.451107421 | 0.40803105 | 0.126048496 |
| 0.592584992 | 0.586428952 | 0.824603601 | 0.446547114 | 0.552621584 | 0.834719057 | 0.735773364 | 0.486197932 | 0.542608104 | 0.474827339 | 0.448537144 | 0.081662255 |
| 0.613953671 | 0.524322726 | 0.808583717 | 0.456923582 | 0.535931456 | 0.848835139 | 0.732679025 | 0.50243308 | 0.547179496 | 0.496491326 | 0.431009904 | 0.054360821 |
| 0.571984783 | 0.485531685 | 0.809756763 | 0.467040727 | 0.470307246 | 0.762950931 | 0.741916456 | 0.499901361 | 0.497107314 | 0.478508165 | 0.435025501 | 0.156362269 |
| 0.618024568 | 0.437092816 | 0.81849619 | 0.462357012 | 0.509484475 | 0.752314302 | 0.720019357 | 0.527844983 | 0.53926298 | 0.46809074 | 0.399585144 | 0.129217204 |
| 0.575404951 | 0.447291969 | 0.800173183 | 0.443635643 | 0.4979176 | 0.789011997 | 0.722357526 | 0.438255372 | 0.547854515 | 0.49084059 | 0.442300895 | 0.062963326 |
| 0.604426861 | 0.501734881 | 0.838487768 | 0.466049691 | 0.482072918 | 0.793290569 | 0.721883492 | 0.478386029 | 0.505203843 | 0.464118385 | 0.446972351 | 0.107684775 |
| 0.603104282 | 0.471554585 | 0.80898002 | 0.449091712 | 0.541444979 | 0.808245366 | 0.710405408 | 0.461348597 | 0.560660288 | 0.497193749 | 0.408037186 | 0.077960738 |
| 0.630895081 | 0.531884277 | 0.828991581 | 0.456526287 | 0.524148338 | 0.878947041 | 0.759392516 | 0.523722107 | 0.514994106 | 0.470597638 | 0.384556056 | 0.135429523 |
| 0.623583211 | 0.513590819 | 0.836812296 | 0.473557617 | 0.45517395 | 0.856055608 | 0.725813921 | 0.460974201 | 0.543807828 | 0.470299593 | 0.392388557 | 0.165747788 |
| 0.609577823 | 0.551023618 | 0.811297798 | 0.448636895 | 0.46900714 | 0.772686341 | 0.741887376 | 0.478301969 | 0.497745862 | 0.476463604 | 0.408604701 | 0.165607071 |
| 0.573498638 | 0.495805482 | 0.740524957 | 0.438444841 | 0.542701806 | 0.904672661 | 0.749440474 | 0.44192725 | 0.539474327 | 0.489447297 | 0.418942521 | 0.137571492 |
| 0.572765279 | 0.463948727 | 0.806660365 | 0.465430806 | 0.498852264 | 0.860174102 | 0.713776311 | 0.452577731 | 0.524783199 | 0.521870331 | 0.401450822 | 0.077894261 |
| 0.650319596 | 0.57364615 | 0.841340336 | 0.45092963 | 0.522841317 | 0.81448557 | 0.774130033 | 0.510689585 | 0.491330384 | 0.504589484 | 0.420149087 | 0.148007504 |
| 0.611908107 | 0.454885446 | 0.859452148 | 0.494119821 | 0.497268089 | 0.742706778 | 0.712945954 | 0.503199885 | 0.556146102 | 0.438153661 | 0.41965018 | 0.108704511 |
| 0.63207475 | 0.582899241 | 0.82332454 | 0.48509413 | 0.503350672 | 0.813122109 | 0.763871275 | 0.471538457 | 0.526558192 | 0.478828672 | 0.436056966 | 0.119951017 |
| 0.608251653 | 0.473966304 | 0.799793641 | 0.446788324 | 0.493358855 | 0.867335683 | 0.732150746 | 0.467368125 | 0.523037469 | 0.454153238 | 0.404015359 | 0.119332268 |
| 0.584988315 | 0.493063498 | 0.806236496 | 0.465145071 | 0.506841612 | 0.842989669 | 0.709796654 | 0.476152201 | 0.565246693 | 0.51022956 | 0.414041185 | 0.103493272 |
| 0.636158229 | 0.471588593 | 0.822328061 | 0.456847156 | 0.518803425 | 0.777596377 | 0.735961642 | 0.524332073 | 0.524637795 | 0.486130664 | 0.452366766 | 0.116319454 |
| 0.648710744 | 0.526839333 | 0.831225074 | 0.455986239 | 0.474283569 | 0.863635829 | 0.725343738 | 0.538467176 | 0.524492269 | 0.458520341 | 0.4215261 | 0.135663802 |
| 0.602427784 | 0.513403242 | 0.835759355 | 0.467533177 | 0.492388333 | 0.87890777 | 0.761242896 | 0.507358908 | 0.531366156 | 0.474062181 | 0.437645278 | 0.212832268 |
| 0.609990866 | 0.525196963 | 0.778739543 | 0.430310343 | 0.563354718 | 0.744699299 | 0.721761978 | 0.457808694 | 0.541699282 | 0.513757476 | 0.40008526 | 0.116441761 |
| 0.61750108 | 0.487362574 | 0.84709309 | 0.495905078 | 0.50905789 | 0.807219963 | 0.72936991 | 0.533885818 | 0.530071923 | 0.467453771 | 0.41494947 | 0.132945573 |
| 0.60711873 | 0.522335409 | 0.810254239 | 0.49097098 | 0.518810817 | 0.936919493 | 0.76787065 | 0.459708505 | 0.509891025 | 0.465478485 | 0.404287931 | 0.114731775 |
| 0.613186597 | 0.497927414 | 0.831527831 | 0.449521576 | 0.515744718 | 0.754651803 | 0.752106044 | 0.496434646 | 0.547984247 | 0.499054638 | 0.440789207 | 0.087294427 |
| 0.637995546 | 0.49607288 | 0.826119179 | 0.470367831 | 0.513903912 | 0.847729947 | 0.725776144 | 0.482310896 | 0.548069426 | 0.467786042 | 0.382435353 | 0.112424149 |
| 0.634640271 | 0.552057812 | 0.814048895 | 0.447814224 | 0.492022477 | 0.861609698 | 0.739053221 | 0.437986997 | 0.509924291 | 0.500079284 | 0.409378138 | 0.100410257 |
| 0.635110517 | 0.57138285 | 0.823458042 | 0.466660594 | 0.498334838 | 0.830406699 | 0.749347279 | 0.502891595 | 0.526021853 | 0.481885046 | 0.440013673 | 0.144880603 |
| 0.655285557 | 0.548072381 | 0.833189837 | 0.481032202 | 0.488473328 | 0.894813671 | 0.73200168 | 0.52012728 | 0.53785999 | 0.454856723 | 0.422418688 | 0.152031101 |
| 0.63059656 | 0.493494791 | 0.844862607 | 0.473125408 | 0.477901699 | 0.921722018 | 0.73825665 | 0.48957867 | 0.540058999 | 0.474543845 | 0.399303664 | 0.140036578 |
| 0.64162119 | 0.566566136 | 0.83325908 | 0.482109422 | 0.512403927 | 0.919694426 | 0.762636426 | 0.483065376 | 0.534515358 | 0.474176857 | 0.415780896 | 0.138593164 |
| 0.617124963 | 0.575266318 | 0.826420124 | 0.462118259 | 0.468681729 | 0.818512082 | 0.73690199 | 0.503078861 | 0.53615945 | 0.509193457 | 0.434716904 | 0.115280459 |
| 0.650688985 | 0.629697847 | 0.834202457 | 0.44591191 | 0.51608382 | 0.777326069 | 0.757380474 | 0.498265421 | 0.496436448 | 0.486171943 | 0.418560981 | 0.136720292 |
| 0.633025923 | 0.48961485 | 0.81069235 | 0.488213002 | 0.502904353 | 0.924812484 | 0.759931803 | 0.490435951 | 0.560170509 | 0.477680036 | 0.379146037 | 0.105201408 |
| 0.61867366 | 0.653026025 | 0.825573007 | 0.482852258 | 0.509420156 | 0.971522767 | 0.771381101 | 0.506373819 | 0.517084843 | 0.462652272 | 0.420051793 | 0.190387784 |
| 0.640476743 | 0.541841147 | 0.83965652 | 0.466478877 | 0.481799197 | 0.912222529 | 0.74110685 | 0.50004818 | 0.508989728 | 0.466438818 | 0.432607316 | 0.132208225 |
| 0.637867337 | 0.47688465 | 0.845964623 | 0.483505328 | 0.492020679 | 0.887461581 | 0.7632288 | 0.529905718 | 0.557718392 | 0.457631281 | 0.426963792 | 0.164496384 |
| 0.630341764 | 0.55712495 | 0.814456753 | 0.422387596 | 0.531872075 | 0.768741813 | 0.742788497 | 0.521608737 | 0.491165644 | 0.514446406 | 0.486184634 | 0.16183237 |
| 0.585676435 | 0.437933161 | 0.810502663 | 0.442688846 | 0.511497134 | 0.816912376 | 0.698372472 | 0.508967345 | 0.529711042 | 0.476034546 | 0.454335486 | 0.055023236 |
| 0.625704872 | 0.558383794 | 0.795147439 | 0.455867165 | 0.517344265 | 0.822425168 | 0.703155547 | 0.570740282 | 0.544633661 | 0.466793851 | 0.442024384 | 0.222158964 |
| 0.599716079 | 0.561382865 | 0.788416543 | 0.439371054 | 0.523377597 | 0.762341166 | 0.758070176 | 0.465128967 | 0.538729251 | 0.488568291 | 0.434117056 | 0.131277289 |
| 0.654125921 | 0.59324297 | 0.843535284 | 0.471634454 | 0.494099565 | 0.859054521 | 0.745242759 | 0.525072479 | 0.523881353 | 0.469563893 | 0.429669841 | 0.164522222 |
| 0.656199998 | 0.497559042 | 0.832312541 | 0.494065859 | 0.498179579 | 0.964800179 | 0.756734834 | 0.589744442 | 0.554073864 | 0.477748288 | 0.413009458 | 0.153587008 |
| 0.670013424 | 0.576190378 | 0.84355673 | 0.454069379 | 0.512723281 | 0.95351524 | 0.748457599 | 0.485104886 | 0.538326054 | 0.460573042 | 0.418909603 | 0.100164629 |
| 0.617681838 | 0.530747404 | 0.81942167 | 0.475945718 | 0.499491185 | 0.826076784 | 0.751382281 | 0.502700343 | 0.535970508 | 0.463086316 | 0.395670536 | 0.131976784 |
| 0.635653932 | 0.5100175 | 0.831552625 | 0.476248348 | 0.533117432 | 0.886268439 | 0.744260356 | 0.446400064 | 0.523059755 | 0.483576618 | 0.445299791 | 0.119336682 |
| 0.632022836 | 0.558746931 | 0.79707158 | 0.456624242 | 0.559904635 | 0.762896002 | 0.713474551 | 0.469370617 | 0.533506981 | 0.499427012 | 0.438983393 | 0.084706174 |
| 0.612655578 | 0.511487584 | 0.804849408 | 0.438445208 | 0.505857595 | 0.847078289 | 0.722170555 | 0.435940694 | 0.537238464 | 0.489724155 | 0.381096236 | 0.074061048 |
| 0.59138076 | 0.457124457 | 0.830305221 | 0.480758054 | 0.469221835 | 0.855597844 | 0.741004742 | 0.534196707 | 0.540348003 | 0.46017295 | 0.446448945 | 0.150024422 |
| 0.584830447 | 0.405777725 | 0.815324633 | 0.433210661 | 0.510362586 | 0.721165788 | 0.715220953 | 0.447265078 | 0.517868819 | 0.47690699 | 0.41456336 | 0.093379966 |
| 0.617589883 | 0.542644938 | 0.812276701 | 0.459624938 | 0.548959139 | 0.907292723 | 0.772603751 | 0.505814756 | 0.524126318 | 0.474109283 | 0.369371577 | 0.110539327 |
| 0.651869121 | 0.522301211 | 0.811909996 | 0.455173274 | 0.520509271 | 0.957068029 | 0.730812268 | 0.498496898 | 0.524869609 | 0.48805649 | 0.4006241 | 0.124859957 |
| 0.618775319 | 0.502337089 | 0.792455233 | 0.460237713 | 0.540811452 | 0.797166362 | 0.73645136 | 0.461553092 | 0.499619512 | 0.496887662 | 0.425888445 | 0.101455875 |
| 0.599429287 | 0.541651209 | 0.830561098 | 0.478237535 | 0.54847448 | 0.891019498 | 0.747240675 | 0.479682925 | 0.529188924 | 0.452964107 | 0.424859523 | 0.158680184 |
| 0.621107826 | 0.529393685 | 0.826622131 | 0.473834539 | 0.491902755 | 0.787296157 | 0.753987559 | 0.504113861 | 0.538891833 | 0.477569558 | 0.445113486 | 0.08307331 |
| 0.603033206 | 0.484392782 | 0.81257384 | 0.468186284 | 0.510822864 | 0.820056899 | 0.725448904 | 0.514013968 | 0.504870881 | 0.468183383 | 0.43017214 | 0.079139538 |
| 0.590662675 | 0.494720788 | 0.793632988 | 0.450072348 | 0.508218202 | 0.807629866 | 0.740129926 | 0.4852305 | 0.54078552 | 0.491378232 | 0.395007647 | 0.129278868 |
| 0.614610108 | 0.523552293 | 0.812500223 | 0.42927265 | 0.503043683 | 0.74757404 | 0.706612421 | 0.511864592 | 0.51541423 | 0.456556872 | 0.377839909 | 0.096790638 |
| 0.644656968 | 0.54911565 | 0.836744465 | 0.47074896 | 0.482514737 | 0.802734783 | 0.716917666 | 0.521358352 | 0.530952543 | 0.452172379 | 0.398358901 | 0.145081919 |
| 0.609067438 | 0.458570013 | 0.822661408 | 0.465187645 | 0.50202309 | 0.871717041 | 0.731411154 | 0.476434623 | 0.54353912 | 0.462170686 | 0.424836038 | 0.126214382 |
| 0.610480545 | 0.535124366 | 0.83061601 | 0.456776527 | 0.479472398 | 0.775888034 | 0.726853905 | 0.46382139 | 0.514404828 | 0.45951145 | 0.393776943 | 0.123920054 |
| 0.586741212 | 0.473626816 | 0.778023152 | 0.466711163 | 0.508508848 | 0.74461789 | 0.74301017 | 0.460354458 | 0.533343795 | 0.476763707 | 0.441072361 | 0.164706846 |
| 0.610375357 | 0.52127381 | 0.778008034 | 0.409563012 | 0.547493631 | 0.778647046 | 0.725365622 | 0.454012312 | 0.520185752 | 0.518492477 | 0.353090994 | 0.107168111 |
| 0.599712044 | 0.530517815 | 0.820193303 | 0.484346364 | 0.499813723 | 0.872431981 | 0.718650013 | 0.503442895 | 0.533030651 | 0.484562298 | 0.417513602 | 0.141929879 |
| 0.623517356 | 0.548713027 | 0.832680065 | 0.464956667 | 0.529296382 | 0.877241817 | 0.714507932 | 0.493936553 | 0.533050136 | 0.485713791 | 0.397835099 | 0.108627601 |
| 0.627806864 | 0.603611723 | 0.807126454 | 0.462845919 | 0.489895049 | 0.807858416 | 0.742998001 | 0.430736346 | 0.502357527 | 0.479485805 | 0.411286382 | 0.165335844 |
| 0.642380857 | 0.57342495 | 0.846133511 | 0.488859728 | 0.504220133 | 0.888614533 | 0.75895136 | 0.48299259 | 0.505306986 | 0.465651892 | 0.406956035 | 0.128207671 |
| 0.606816266 | 0.526801678 | 0.811167364 | 0.477430511 | 0.497176301 | 0.832670725 | 0.740326189 | 0.476164441 | 0.501373399 | 0.465313553 | 0.445148173 | 0.100566563 |
| 0.60995843 | 0.499598066 | 0.784345155 | 0.432445208 | 0.545375634 | 0.774401177 | 0.717341834 | 0.431322771 | 0.53226566 | 0.499455638 | 0.365823567 | 0.098645271 |
| 0.638418806 | 0.598637251 | 0.783990968 | 0.434055218 | 0.497232825 | 0.853851571 | 0.715775094 | 0.476152912 | 0.538671336 | 0.495148704 | 0.398156992 | 0.136476014 |
| 0.598108599 | 0.528793757 | 0.779091976 | 0.425714041 | 0.528417326 | 0.751321179 | 0.691303092 | 0.465801146 | 0.526506665 | 0.506956161 | 0.40825249 | 0.097039197 |
| 0.634930354 | 0.543391837 | 0.824455286 | 0.44302339 | 0.515132177 | 0.858725422 | 0.746114031 | 0.504733346 | 0.521377404 | 0.485889182 | 0.406893208 | 0.155099164 |
| 0.582868101 | 0.44829496 | 0.787203425 | 0.415872124 | 0.507830483 | 0.802107112 | 0.72703633 | 0.452680836 | 0.532981698 | 0.513669146 | 0.419914494 | 0.130297543 |
| 0.614201931 | 0.493584495 | 0.800657437 | 0.42726078 | 0.49034242 | 0.757937395 | 0.708070817 | 0.423424465 | 0.511411961 | 0.490762333 | 0.376851352 | 0.140021401 |
| 0.61259912 | 0.501049853 | 0.786741078 | 0.446437143 | 0.508506192 | 0.804180483 | 0.721515407 | 0.475171236 | 0.536667688 | 0.494502978 | 0.355258965 | 0.129390403 |
| 0.624192733 | 0.560694602 | 0.841139911 | 0.471092755 | 0.531299148 | 0.835405075 | 0.76138661 | 0.518408593 | 0.528531219 | 0.476722534 | 0.393450118 | 0.169610618 |
| 0.654658081 | 0.606236036 | 0.83361085 | 0.483157783 | 0.505645229 | 0.805328777 | 0.760459843 | 0.482198952 | 0.537225572 | 0.489433312 | 0.400754164 | 0.094761483 |
| 0.667738442 | 0.569603602 | 0.82744612 | 0.464154754 | 0.523684993 | 0.88109775 | 0.778992604 | 0.554305054 | 0.528709508 | 0.478093541 | 0.445201154 | 0.145554609 |
| 0.626492851 | 0.534865517 | 0.821010547 | 0.478499299 | 0.509180832 | 0.872778401 | 0.75426708 | 0.510480468 | 0.513512789 | 0.471348757 | 0.428798154 | 0.151174554 |
| 0.620510005 | 0.451652435 | 0.829968823 | 0.477549484 | 0.511771929 | 0.833342853 | 0.747323607 | 0.487207672 | 0.517044381 | 0.477791069 | 0.393268055 | 0.140325825 |
| 0.635999744 | 0.520862029 | 0.834851952 | 0.4909657 | 0.518302569 | 0.854172352 | 0.753308826 | 0.462215987 | 0.512938054 | 0.47914324 | 0.391601832 | 0.112920486 |
| 0.604541326 | 0.467069929 | 0.832534377 | 0.504333073 | 0.454623829 | 0.807076658 | 0.751313241 | 0.464703313 | 0.514537105 | 0.482549991 | 0.359986563 | 0.177112946 |
| 0.639214879 | 0.558219709 | 0.84967851 | 0.491504835 | 0.515416308 | 0.973439579 | 0.770591497 | 0.481199307 | 0.532191703 | 0.442039671 | 0.438614135 | 0.181829043 |
| 0.597724032 | 0.555746163 | 0.824928408 | 0.481067805 | 0.523517064 | 0.882039204 | 0.745343783 | 0.502160599 | 0.519803575 | 0.453403926 | 0.453349154 | 0.178760568 |
| 0.633296841 | 0.504297143 | 0.805222712 | 0.437180833 | 0.496834743 | 0.834283328 | 0.758308348 | 0.447075152 | 0.513086843 | 0.489588445 | 0.392367827 | 0.137413998 |
| 0.60993006 | 0.629613242 | 0.80670189 | 0.491930141 | 0.524603581 | 0.757513276 | 0.751529751 | 0.463592754 | 0.513698989 | 0.506078991 | 0.42905428 | 0.138758232 |
| 0.644245198 | 0.547267693 | 0.835556869 | 0.48021212 | 0.508094497 | 0.866245662 | 0.762236 | 0.459096264 | 0.510648166 | 0.486929603 | 0.449225525 | 0.18282527 |
| 0.654952774 | 0.562030325 | 0.819111538 | 0.466547844 | 0.535708037 | 0.890406807 | 0.774327913 | 0.500962053 | 0.528040731 | 0.510486779 | 0.394998586 | 0.133529478 |
| 0.621924613 | 0.535046888 | 0.82885822 | 0.464755255 | 0.504127509 | 0.843350382 | 0.755076304 | 0.471000629 | 0.493763294 | 0.478099198 | 0.439695391 | 0.182457393 |
| 0.681111262 | 0.599775864 | 0.83387258 | 0.456418757 | 0.50726677 | 0.807764769 | 0.760812594 | 0.525833701 | 0.485957241 | 0.443271632 | 0.441781673 | 0.168008637 |
| 0.599330699 | 0.432407889 | 0.79558653 | 0.452855118 | 0.478994136 | 0.720664591 | 0.718551909 | 0.482434816 | 0.491765745 | 0.490585956 | 0.399460692 | 0.146575006 |
| 0.620454347 | 0.544764671 | 0.827044604 | 0.463624177 | 0.491527062 | 0.809048706 | 0.752990746 | 0.454291942 | 0.494010016 | 0.467943325 | 0.426977059 | 0.170932064 |
| 0.607042757 | 0.532799683 | 0.823852153 | 0.45567437 | 0.490433892 | 0.854567265 | 0.736255877 | 0.490289692 | 0.486305054 | 0.458496961 | 0.43264824 | 0.192115742 |
| 0.621493615 | 0.522668704 | 0.81933169 | 0.488287896 | 0.513462305 | 0.762324843 | 0.751730342 | 0.461284155 | 0.499457646 | 0.457020713 | 0.454342455 | 0.215231093 |
| 0.644208456 | 0.618217639 | 0.823444029 | 0.478493226 | 0.526842638 | 0.767224258 | 0.744206 | 0.465243015 | 0.494996222 | 0.443881396 | 0.423442725 | 0.204037542 |
| 0.595204246 | 0.502281999 | 0.825620626 | 0.50681513 | 0.515057668 | 0.805372774 | 0.750174502 | 0.513863524 | 0.536435847 | 0.535450831 | 0.442129107 | 0.103282234 |
| 0.616255547 | 0.501251219 | 0.809226574 | 0.459775102 | 0.495658221 | 0.820208819 | 0.744875184 | 0.45874999 | 0.491988726 | 0.47955113 | 0.432460614 | 0.192369963 |
| 0.672323996 | 0.588405163 | 0.838793323 | 0.488374804 | 0.5143069 | 0.879564094 | 0.760511948 | 0.45539441 | 0.489799905 | 0.484569088 | 0.417442658 | 0.112005248 |
| 0.628990497 | 0.565137505 | 0.821587359 | 0.465551694 | 0.525184879 | 0.909324759 | 0.777894723 | 0.483016089 | 0.525550473 | 0.489139689 | 0.454479558 | 0.139671241 |
| 0.637555524 | 0.457215587 | 0.844980698 | 0.474578771 | 0.534600873 | 0.946741312 | 0.789727885 | 0.514990493 | 0.490557854 | 0.466729811 | 0.403567685 | 0.119518516 |
| 0.621343321 | 0.57263096 | 0.84751726 | 0.469441284 | 0.507378376 | 0.892116285 | 0.782915929 | 0.510381824 | 0.506990154 | 0.477196969 | 0.428205525 | 0.088061797 |
| 0.59096491 | 0.445606209 | 0.812858863 | 0.457595068 | 0.503125702 | 0.74492486 | 0.715011121 | 0.480818766 | 0.501271803 | 0.497546242 | 0.422972409 | 0.109160918 |
| 0.60323388 | 0.527776306 | 0.821201202 | 0.485162371 | 0.514055267 | 0.820538956 | 0.725562282 | 0.505156773 | 0.512059048 | 0.480945959 | 0.414836571 | 0.136970914 |
| 0.628994995 | 0.510040696 | 0.808314619 | 0.452510979 | 0.505347165 | 0.837491371 | 0.733226322 | 0.456210331 | 0.543966847 | 0.507424332 | 0.414771574 | 0.113605224 |
| 0.631154936 | 0.532982589 | 0.800122631 | 0.444076673 | 0.508446187 | 0.81893209 | 0.714568269 | 0.452948412 | 0.49255686 | 0.475876186 | 0.460229798 | 0.158965167 |
| 0.641228571 | 0.499258364 | 0.808997344 | 0.457182964 | 0.486313984 | 0.914034701 | 0.729190064 | 0.458334578 | 0.54215206 | 0.476102106 | 0.418721409 | 0.050622269 |
| 0.619554757 | 0.508075874 | 0.828751781 | 0.485108248 | 0.507614053 | 0.861660755 | 0.74452479 | 0.520952649 | 0.52324746 | 0.486492524 | 0.434291569 | 0.150656846 |
| 0.605601329 | 0.57606696 | 0.827213069 | 0.488200627 | 0.520066881 | 0.818054055 | 0.747034327 | 0.542927754 | 0.55615172 | 0.485884161 | 0.419067357 | 0.123251421 |
| 0.614933874 | 0.464980316 | 0.843605449 | 0.474700943 | 0.481453637 | 0.828040158 | 0.745245022 | 0.520953766 | 0.514905596 | 0.464096893 | 0.415450814 | 0.096854033 |
| 0.61482806 | 0.511366261 | 0.835632122 | 0.473401982 | 0.513794385 | 0.853048807 | 0.748824472 | 0.47089474 | 0.499327262 | 0.487522159 | 0.429762345 | 0.130190488 |
| 0.625986255 | 0.530631761 | 0.850010965 | 0.493766348 | 0.523301087 | 0.885175082 | 0.74396735 | 0.488354171 | 0.51584079 | 0.485662836 | 0.395029119 | 0.143795479 |
| 0.645013382 | 0.492978616 | 0.832924762 | 0.452537961 | 0.513232039 | 0.892571733 | 0.75212591 | 0.53878844 | 0.503385833 | 0.50001602 | 0.457592488 | 0.145675147 |
| 0.593539146 | 0.48074514 | 0.83594685 | 0.480818808 | 0.512402461 | 0.757214128 | 0.745709424 | 0.4950028 | 0.543487304 | 0.475011754 | 0.462048381 | 0.128499731 |
| 0.633447743 | 0.486918658 | 0.849555938 | 0.483740634 | 0.509693257 | 0.932110085 | 0.782800375 | 0.527865132 | 0.499542229 | 0.453392882 | 0.43686856 | 0.142987446 |
| 0.623501156 | 0.463601868 | 0.856830863 | 0.484864715 | 0.517715908 | 0.848677688 | 0.754539309 | 0.512948121 | 0.507466788 | 0.469016194 | 0.446624929 | 0.135664537 |
| 0.627269723 | 0.451114678 | 0.789943991 | 0.44166085 | 0.505153652 | 0.795771416 | 0.715246902 | 0.447768567 | 0.519182035 | 0.475807534 | 0.409263155 | 0.129613024 |
| 0.648435249 | 0.568691194 | 0.844595131 | 0.500524999 | 0.540062975 | 0.866463475 | 0.754792063 | 0.558924564 | 0.544242898 | 0.452752752 | 0.43243111 | 0.175846112 |
| 0.650614021 | 0.595275973 | 0.852865866 | 0.490248295 | 0.469590208 | 0.891290293 | 0.722151213 | 0.507820117 | 0.530510089 | 0.471778717 | 0.371917234 | 0.10518848 |
| 0.570948361 | 0.4324771 | 0.813905297 | 0.444401647 | 0.446868746 | 0.698104675 | 0.683047692 | 0.534351289 | 0.542306861 | 0.484758859 | 0.420578123 | 0.09067137 |
| 0.627293996 | 0.565146457 | 0.820418722 | 0.45186778 | 0.4994348 | 0.888534449 | 0.748633224 | 0.496575099 | 0.529549527 | 0.470995148 | 0.431667463 | 0.177938888 |
| 0.656241404 | 0.558565077 | 0.848749908 | 0.462308025 | 0.5202775 | 0.896984122 | 0.754788781 | 0.562931345 | 0.533296073 | 0.459392602 | 0.360189936 | 0.166935791 |
| 0.601785822 | 0.574164704 | 0.825523148 | 0.4750481 | 0.517274644 | 0.886068872 | 0.764686917 | 0.446841302 | 0.514894224 | 0.447408577 | 0.385898744 | 0.160021801 |
| 0.623750418 | 0.575334424 | 0.815294306 | 0.495650664 | 0.520827985 | 0.862288773 | 0.751411207 | 0.494901381 | 0.539986422 | 0.458390153 | 0.375380961 | 0.200912925 |
| 0.633993995 | 0.519578853 | 0.802729296 | 0.426301121 | 0.503739839 | 0.873790662 | 0.72743258 | 0.488398317 | 0.513920691 | 0.478250653 | 0.441764227 | 0.129232587 |
| 0.611719428 | 0.524740674 | 0.825613955 | 0.479299802 | 0.519220447 | 0.78333983 | 0.714613287 | 0.46344739 | 0.554196268 | 0.48731461 | 0.403239746 | 0.042747696 |
| 0.630541742 | 0.490306226 | 0.780489368 | 0.444462909 | 0.525414797 | 0.991802479 | 0.747428054 | 0.455521961 | 0.52456758 | 0.497382022 | 0.393793656 | 0.112494326 |
| 0.567363033 | 0.554252623 | 0.816956744 | 0.467204348 | 0.490622962 | 0.844619592 | 0.730775917 | 0.434827017 | 0.515224584 | 0.450787667 | 0.422440327 | 0.238510808 |
| 0.643610892 | 0.565208364 | 0.841994885 | 0.459653564 | 0.516817375 | 0.797720134 | 0.725580586 | 0.568784786 | 0.520355584 | 0.46687569 | 0.398646585 | 0.176136958 |
| 0.627906869 | 0.537620917 | 0.838603759 | 0.47288085 | 0.521267715 | 0.857349946 | 0.751762731 | 0.503413566 | 0.529800486 | 0.494193943 | 0.429665655 | 0.113897374 |
| 0.590014456 | 0.48382251 | 0.830505685 | 0.463927544 | 0.522311052 | 0.714246631 | 0.700018836 | 0.472224289 | 0.542592803 | 0.478496697 | 0.438323446 | 0.101147406 |
| 0.606589538 | 0.524550187 | 0.844115397 | 0.486798141 | 0.507147985 | 0.82203189 | 0.73131308 | 0.52256294 | 0.546774842 | 0.464448191 | 0.427346652 | 0.11238885 |
| 0.608681162 | 0.495799987 | 0.816430345 | 0.473999045 | 0.512993275 | 0.791751789 | 0.725521994 | 0.491343362 | 0.543050515 | 0.501542245 | 0.424368506 | 0.080070451 |
| 0.601595266 | 0.452056803 | 0.814908266 | 0.464550821 | 0.512313762 | 0.740124424 | 0.725513866 | 0.492827035 | 0.537572514 | 0.488311723 | 0.436168509 | 0.08229402 |
| 0.651991217 | 0.541733083 | 0.798789954 | 0.467841172 | 0.497153167 | 0.803421315 | 0.725416859 | 0.495290977 | 0.537760779 | 0.494455944 | 0.438709251 | 0.115125407 |
| 0.590492355 | 0.47336403 | 0.836874366 | 0.464514343 | 0.497521588 | 0.812528299 | 0.706763436 | 0.516895836 | 0.538335868 | 0.459809123 | 0.412248646 | 0.107248814 |
| 0.629207915 | 0.469647109 | 0.81897699 | 0.490850529 | 0.496499127 | 0.861406961 | 0.731984698 | 0.516014933 | 0.536459123 | 0.485289631 | 0.424665789 | 0.103414293 |
| 0.607037793 | 0.509646671 | 0.810110538 | 0.499114384 | 0.476790405 | 0.819647027 | 0.722787053 | 0.500871716 | 0.541595986 | 0.472867235 | 0.403480168 | 0.094866747 |
| 0.607771152 | 0.46140346 | 0.803829393 | 0.461231788 | 0.527937461 | 1 | 0.751404426 | 0.45003454 | 0.49507701 | 0.486465514 | 0.380589358 | 0.103923417 |
| 0.611704698 | 0.521326271 | 0.82744752 | 0.486051352 | 0.488491605 | 0.831903176 | 0.732687081 | 0.500028001 | 0.531362994 | 0.458133566 | 0.385747882 | 0.124061469 |
| 0.622246601 | 0.514252105 | 0.815377895 | 0.483692959 | 0.492237654 | 0.878688863 | 0.699113515 | 0.506485772 | 0.564128566 | 0.513280728 | 0.436464335 | 0.045006677 |
| 0.63337907 | 0.51538093 | 0.836643532 | 0.452792962 | 0.477796325 | 0.80964521 | 0.709323294 | 0.465886734 | 0.533054615 | 0.485001136 | 0.440297056 | 0.056079683 |
| 0.624011761 | 0.555948734 | 0.792074286 | 0.44128399 | 0.504777808 | 0.912675303 | 0.753037267 | 0.493664145 | 0.517058223 | 0.471799311 | 0.398068882 | 0.204147908 |
| 0.616630438 | 0.509401696 | 0.834186174 | 0.507987738 | 0.491492429 | 0.793195746 | 0.703602979 | 0.515573619 | 0.539680165 | 0.485921123 | 0.451905403 | 0.131494774 |
| 0.64805304 | 0.514435085 | 0.831449644 | 0.471720575 | 0.492397926 | 0.803199624 | 0.698457783 | 0.520761698 | 0.567894088 | 0.492092789 | 0.409901168 | 0.1252814 |
| 0.617090693 | 0.547595414 | 0.837106368 | 0.47533518 | 0.511385962 | 0.88295451 | 0.731291551 | 0.521054146 | 0.488592256 | 0.453427219 | 0.356019548 | 0.127663889 |
| 0.602663865 | 0.525184942 | 0.819974673 | 0.466649387 | 0.503487053 | 0.901146676 | 0.751089058 | 0.476383189 | 0.563576102 | 0.513119253 | 0.409484443 | 0.075947116 |
| 0.617811676 | 0.522048184 | 0.782281808 | 0.49611213 | 0.519975315 | 0.971116607 | 0.743276194 | 0.428934858 | 0.505485229 | 0.490855919 | 0.365142739 | 0.104078224 |
| 0.604130349 | 0.440259644 | 0.807514735 | 0.456891846 | 0.501622348 | 0.894158892 | 0.708034054 | 0.43807528 | 0.52665986 | 0.49506504 | 0.457983563 | 0.07161216 |
| 0.623335263 | 0.465072843 | 0.839200565 | 0.449140909 | 0.479163927 | 0.877560324 | 0.736453339 | 0.526033115 | 0.513614577 | 0.482081693 | 0.430895093 | 0.101877949 |
| 0.555556055 | 0.510226394 | 0.812053538 | 0.452245299 | 0.508180251 | 0.778582668 | 0.719613492 | 0.44104497 | 0.506300298 | 0.449367635 | 0.403338124 | 0.149485904 |

Con:Normal; treat:Coronary artery disease(CAD)
