# Supplementary material for: Identification of molecular subtypes of coronary artery disease based on ferroptosis- and necroptosis-related genes
Source: Front Genet. 2022 Sep 20;13:870222. doi: 10.3389/fgene.2022.870222 (PMC9531137; doi:10.3389/fgene.2022.870222)
Supplement: Supplementary file 5 [file Table3.docx]

**Supplement Table3.** The consensus clustering approach to identify two subgroups (clusterA and cluster B) based on the expression of the 25 significant Ferroptosis- and Necroptosis-related DEGs.

| Samples | Cluster |
| --- | --- |
| GSM308600 | A |
| GSM308601 | B |
| GSM308603 | A |
| GSM308604 | B |
| GSM308605 | A |
| GSM308606 | A |
| GSM308607 | A |
| GSM308608 | A |
| GSM308609 | A |
| GSM308610 | B |
| GSM308611 | A |
| GSM308613 | B |
| GSM308614 | B |
| GSM308615 | B |
| GSM308616 | B |
| GSM308617 | B |
| GSM308618 | A |
| GSM308620 | A |
| GSM308622 | B |
| GSM308623 | A |
| GSM308625 | B |
| GSM308626 | A |
| GSM308627 | B |
| GSM308629 | B |
| GSM308630 | B |
| GSM308631 | A |
| GSM308632 | B |
| GSM308634 | B |
| GSM308637 | B |
| GSM308639 | A |
| GSM308640 | B |
| GSM308641 | B |
| GSM308642 | B |
| GSM308644 | A |
| GSM308645 | B |
| GSM308646 | A |
| GSM308647 | B |
| GSM308650 | A |
| GSM308652 | B |
| GSM308653 | B |
| GSM308655 | A |
| GSM308657 | B |
| GSM308658 | A |
| GSM308659 | A |
| GSM308664 | B |
| GSM308666 | B |
| GSM308667 | B |
| GSM308668 | A |
| GSM308669 | B |
| GSM308670 | B |
| GSM308671 | B |
| GSM308672 | A |
| GSM308673 | B |
| GSM308674 | B |
| GSM308675 | B |
| GSM308676 | B |
| GSM308678 | A |
| GSM308679 | B |
| GSM308681 | B |
| GSM308685 | B |
| GSM308686 | B |
| GSM308688 | B |
| GSM308689 | A |
| GSM308690 | B |
| GSM308691 | A |
| GSM308692 | A |
| GSM308693 | B |
| GSM308694 | B |
| GSM308695 | A |
| GSM308696 | B |
| GSM308697 | B |
| GSM308699 | A |
| GSM308700 | A |
| GSM308703 | A |
| GSM308704 | B |
| GSM308705 | A |
| GSM308707 | A |
| GSM308708 | A |
| GSM308710 | A |
| GSM308711 | B |
| GSM308715 | A |
| GSM308717 | B |
| GSM308718 | B |
| GSM308719 | A |
| GSM308723 | A |
| GSM308724 | B |
| GSM308725 | A |
| GSM308727 | B |
| GSM308734 | A |
| GSM308736 | B |
| GSM308737 | B |
| GSM308741 | B |
| GSM308742 | A |
| GSM308743 | A |
| GSM308745 | B |
| GSM308747 | A |
| GSM308752 | B |
| GSM308754 | B |
| GSM308755 | B |
| GSM308756 | B |
| GSM308759 | B |
| GSM308773 | B |
| GSM308775 | B |
| GSM308776 | B |
| GSM308778 | A |
| GSM308780 | B |
| GSM308781 | A |
| GSM308782 | A |
| GSM308783 | B |
| GSM308784 | B |
| GSM518690 | B |
| GSM518691 | B |
| GSM518692 | B |
| GSM518693 | B |
| GSM518694 | B |
| GSM518695 | B |
| GSM518696 | B |
| GSM518697 | B |
| GSM518698 | A |
| GSM518699 | A |
| GSM518700 | B |
| GSM518701 | B |
| GSM518702 | A |
| GSM518703 | B |
| GSM518704 | B |
| GSM518705 | A |
| GSM518706 | A |
| GSM518707 | B |
| GSM518708 | B |
| GSM518709 | B |
| GSM518710 | A |
| GSM518711 | B |
| GSM518712 | B |
| GSM518713 | B |
| GSM518714 | B |
| GSM518715 | B |
| GSM518716 | B |
| GSM518717 | A |
| GSM518718 | B |
| GSM518719 | B |
| GSM518720 | A |
| GSM518721 | B |
| GSM518722 | B |
| GSM518723 | A |
| GSM518724 | B |
| GSM518725 | B |
| GSM518726 | A |
| GSM518727 | B |
| GSM518728 | B |
| GSM518729 | B |
| GSM518730 | B |
| GSM518731 | A |
| GSM518732 | B |
| GSM518733 | A |
| GSM518734 | A |
| GSM518735 | A |
| GSM518736 | B |
| GSM518737 | A |
| GSM518738 | B |
| GSM518739 | A |
| GSM518740 | A |
| GSM518741 | B |
| GSM518742 | A |
| GSM518743 | B |
| GSM518744 | A |
| GSM518745 | A |
| GSM518746 | A |
| GSM518747 | A |
| GSM518748 | B |
| GSM518749 | B |
| GSM518750 | B |
| GSM518751 | A |
| GSM518752 | B |
| GSM518753 | B |
| GSM518754 | B |
| GSM518755 | A |
| GSM518756 | B |
| GSM518757 | A |
| GSM518758 | A |
| GSM518759 | A |
| GSM518760 | B |
| GSM518761 | B |
| GSM518762 | B |
| GSM518763 | B |
| GSM518764 | B |
| GSM518765 | B |
| GSM518766 | B |
| GSM518767 | A |
| GSM518768 | A |
| GSM518769 | B |
| GSM518770 | A |
| GSM518771 | B |
| GSM518772 | B |
| GSM518773 | A |
| GSM518774 | A |
| GSM518775 | A |
| GSM518776 | B |
| GSM518777 | B |
| GSM518778 | A |
| GSM518779 | B |
| GSM518780 | A |
| GSM518781 | B |
| GSM518782 | B |
| GSM518783 | A |
| GSM518784 | B |
| GSM518785 | B |
| GSM518786 | B |
| GSM518787 | B |
| GSM518788 | B |
| GSM518789 | B |
| GSM518790 | A |
| GSM518791 | B |
| GSM518792 | B |
| GSM518793 | A |
| GSM518794 | B |
| GSM518795 | A |
| GSM518796 | B |
| GSM518797 | B |
| GSM518798 | B |
| GSM518799 | B |
| GSM518800 | B |
| GSM518801 | A |
| GSM518802 | A |
| GSM518803 | B |
| GSM518804 | B |
| GSM518805 | A |
| GSM518806 | B |
| GSM518807 | B |
| GSM518808 | B |
| GSM518809 | B |
| GSM518810 | A |
| GSM518811 | A |
| GSM518812 | A |
| GSM518813 | B |
| GSM518814 | B |
| GSM518815 | A |
| GSM518816 | A |
| GSM518817 | A |
| GSM518818 | B |
| GSM518819 | B |
| GSM518820 | B |
| GSM518821 | A |
| GSM518822 | B |
| GSM518823 | A |
| GSM518824 | A |
| GSM518825 | A |
| GSM518826 | A |
| GSM518827 | A |
| GSM518828 | B |
| GSM518829 | B |
| GSM518830 | B |
| GSM518831 | B |
| GSM518832 | A |
| GSM518885 | A |
| GSM518887 | B |
| GSM518889 | A |
| GSM518891 | A |
| GSM518893 | B |
| GSM518895 | B |
| GSM518897 | B |
| GSM518899 | B |
| GSM518901 | B |
| GSM518903 | B |
| GSM518905 | A |
| GSM518907 | B |
| GSM518909 | B |
| GSM518911 | A |
| GSM518913 | A |
| GSM518915 | A |
| GSM518917 | B |
| GSM518919 | B |
| GSM518921 | B |
| GSM518923 | B |
| GSM518925 | A |
| GSM518927 | B |
| GSM518929 | B |
| GSM518931 | B |
| GSM518933 | A |
| GSM518935 | B |
| GSM518937 | A |
| GSM518939 | A |
| GSM518941 | B |
| GSM518943 | A |
| GSM518945 | B |
| GSM518947 | A |
| GSM518949 | A |
| GSM518951 | A |
| GSM518953 | A |
| GSM518955 | A |
| GSM518957 | A |
| GSM518959 | A |
| GSM518961 | A |
| GSM518963 | B |
| GSM518965 | B |
| GSM518967 | A |
| GSM518969 | B |
| GSM518971 | B |
| GSM518973 | B |
| GSM518975 | A |
| GSM518977 | A |
| GSM518979 | A |
| GSM518981 | A |
| GSM518983 | A |
| GSM518985 | B |
| GSM518987 | B |
| GSM518989 | B |
| GSM518991 | B |
| GSM518993 | A |
| GSM518995 | B |
| GSM518997 | B |
| GSM518999 | B |
| GSM519001 | A |
| GSM519003 | A |
| GSM519005 | A |
| GSM519007 | B |
| GSM519009 | A |
| GSM519011 | A |
| GSM519013 | B |
| GSM519015 | B |
| GSM519017 | A |
| GSM519019 | B |
| GSM519021 | B |
| GSM519023 | A |
| GSM519025 | B |
| GSM519027 | A |
| GSM519029 | B |
| GSM519031 | B |
| GSM519033 | A |
| GSM519035 | B |
| GSM519037 | B |
| GSM519039 | B |
| GSM519041 | B |
| GSM519043 | B |
| GSM519045 | B |
| GSM519047 | B |
| GSM519049 | A |
| GSM519051 | B |
| GSM519053 | B |
| GSM519055 | B |
| GSM519057 | A |
| GSM519059 | B |
| GSM519061 | B |
| GSM519063 | B |
| GSM519065 | A |
| GSM519067 | B |
| GSM519069 | B |
| GSM519071 | A |
| GSM519073 | B |
| GSM519075 | A |
| GSM519077 | B |
| GSM519079 | B |
| GSM519081 | B |
