# Supplementary material for: Identification of molecular subtypes of coronary artery disease based on ferroptosis- and necroptosis-related genes
Source: Front Genet. 2022 Sep 20;13:870222. doi: 10.3389/fgene.2022.870222 (PMC9531137; doi:10.3389/fgene.2022.870222)
Supplement: Supplementary file 6 [file DataSheet2.docx]

Supplementary Material

# Supplementary Figures and Tables

## Supplementary Figures

**Supplementary Figure 1.** Differences in the abundances of infiltrating immune cells between 11 high and low ferroptosis- and necroptosis-related gene expression groups. (A) *CBS*. (B) *FADD*. (C) *FLT*. (D) *ITPK1*. (E) *MAP3K5*. (F) *MAPK14*. (G) *NCF2*. (H) *PGD*. (I) *STAT3*. (J) *TLR4*. (K) *TNFSF10*.

## Supplementary Tables

**Supplementary Table 1.** Ferroptosis- and necroptosis-related genes.

**Supplementary Table 2.** Information on the characteristics of the GSE12288, GSE20680, GSE20681, and [GSE](https://www.ncbi.nlm.nih.gov/geo/query/acc.cgi?acc=GSE68506)180083 datasets.

**Supplementary Table 3.** The consensus clustering approach to identify two subgroups (cluster A and cluster B) based on the expression of the 25 significant ferroptosis- and necroptosis-related DEGs.

**Supplementary Table 4.** GO and KEGG enrichment analysis of DEGs.

**Supplementary Table 5.** Abundances of immune cells in CAD samples, as calculated using ssGSEA.

**Supplementary Table 6.** The consensus clustering approach to identify two subgroups (cluster A and cluster B) based on the expression of four subgroup‐specific genes.

**Supplementary Table 7.** Scores for the four subgroup-specific genes in CAD samples, as calculated using PCA.
