# Supplementary material for: Identification of molecular subtypes of coronary artery disease based on ferroptosis- and necroptosis-related genes
Source: Front Genet. 2022 Sep 20;13:870222. doi: 10.3389/fgene.2022.870222 (PMC9531137; doi:10.3389/fgene.2022.870222)
Supplement: Supplementary file 7 [file Table4.docx]

**Supplement Table4.** GO and KEGG enrichment analysis of DEGs.

| Function enrichment analysis | ONTOLOGY | ID | Description | GeneRatio | pvalue | p.adjust | qvalue | geneID | Count |
| --- | --- | --- | --- | --- | --- | --- | --- | --- | --- |
| GO | up-regulated genes in cluster A | | | | | | | | |
|  | BP | GO:0043312 | neutrophil degranulation | 15/55 | 5.31E-12 | 3.39E-09 | 2.76E-09 | MMP9/ARG1/MGAM/ORM1/PGLYRP1/QPCT/FCGR3B/IGF2R/ANXA3/MME/TNFAIP6/PYGL/FPR2/MMP25/CXCL1 | 15 |
|  | BP | GO:0002283 | neutrophil activation involved in immune response | 15/55 | 5.79E-12 | 3.39E-09 | 2.76E-09 | MMP9/ARG1/MGAM/ORM1/PGLYRP1/QPCT/FCGR3B/IGF2R/ANXA3/MME/TNFAIP6/PYGL/FPR2/MMP25/CXCL1 | 15 |
|  | BP | GO:0002446 | neutrophil mediated immunity | 15/55 | 7.95E-12 | 3.39E-09 | 2.76E-09 | MMP9/ARG1/MGAM/ORM1/PGLYRP1/QPCT/FCGR3B/IGF2R/ANXA3/MME/TNFAIP6/PYGL/FPR2/MMP25/CXCL1 | 15 |
|  | BP | GO:0042119 | neutrophil activation | 15/55 | 8.18E-12 | 3.39E-09 | 2.76E-09 | MMP9/ARG1/MGAM/ORM1/PGLYRP1/QPCT/FCGR3B/IGF2R/ANXA3/MME/TNFAIP6/PYGL/FPR2/MMP25/CXCL1 | 15 |
|  | BP | GO:0051384 | response to glucocorticoid | 5/55 | 4.55E-05 | 0.01510068 | 0.012292866 | ARG1/DUSP1/ADM/ANXA3/FOS | 5 |
|  | BP | GO:0031960 | response to corticosteroid | 5/55 | 8.00E-05 | 0.022118435 | 0.018005744 | ARG1/DUSP1/ADM/ANXA3/FOS | 5 |
|  | BP | GO:0071496 | cellular response to external stimulus | 6/55 | 0.000244672 | 0.057987339 | 0.047205201 | CBS/FOS/KCNJ2/TLR4/RALB/INHBB | 6 |
|  | CC | GO:1904724 | tertiary granule lumen | 6/55 | 9.85E-09 | 9.04E-07 | 8.11E-07 | MMP9/ORM1/PGLYRP1/QPCT/TNFAIP6/CXCL1 | 6 |
|  | CC | GO:0042581 | specific granule | 8/55 | 1.50E-08 | 9.04E-07 | 8.11E-07 | ARG1/ORM1/PGLYRP1/QPCT/ANXA3/FPR2/MMP25/CXCL1 | 8 |
|  | CC | GO:0070820 | tertiary granule | 8/55 | 1.82E-08 | 9.04E-07 | 8.11E-07 | MMP9/MGAM/ORM1/PGLYRP1/QPCT/TNFAIP6/FPR2/CXCL1 | 8 |
|  | CC | GO:0035580 | specific granule lumen | 5/55 | 8.44E-07 | 3.15E-05 | 2.82E-05 | ARG1/ORM1/PGLYRP1/QPCT/CXCL1 | 5 |
|  | CC | GO:0101002 | ficolin-1-rich granule | 6/55 | 1.32E-05 | 0.000392558 | 0.000352207 | MMP9/MGAM/QPCT/TNFAIP6/PYGL/FPR2 | 6 |
|  | CC | GO:0030667 | secretory granule membrane | 6/55 | 0.000211025 | 0.005161114 | 0.0046306 | MGAM/FCGR3B/IGF2R/MME/FPR2/MMP25 | 6 |
|  | CC | GO:0034774 | secretory granule lumen | 6/55 | 0.000282439 | 0.005161114 | 0.0046306 | ARG1/ORM1/PGLYRP1/QPCT/PYGL/CXCL1 | 6 |
|  | CC | GO:0060205 | cytoplasmic vesicle lumen | 6/55 | 0.000301723 | 0.005161114 | 0.0046306 | ARG1/ORM1/PGLYRP1/QPCT/PYGL/CXCL1 | 6 |
|  | CC | GO:0031983 | vesicle lumen | 6/55 | 0.000311745 | 0.005161114 | 0.0046306 | ARG1/ORM1/PGLYRP1/QPCT/PYGL/CXCL1 | 6 |
|  | CC | GO:1904813 | ficolin-1-rich granule lumen | 4/55 | 0.000411859 | 0.006136701 | 0.005505906 | MMP9/QPCT/TNFAIP6/PYGL | 4 |
|  | MF | GO:0038187 | pattern recognition receptor activity | 3/55 | 4.33E-05 | 0.009664138 | 0.007709414 | CLEC4E/PGLYRP1/TLR4 | 3 |
|  | up-regulated genes in cluster B | | | | | | | | |
|  | BP | GO:0050851 | antigen receptor-mediated signaling pathway | 7/30 | 5.87E-07 | 0.000368813 | 0.000284986 | MS4A1/LCK/CD3D/IGHD/CD79B/IGHM/CD3G | 7 |
|  | BP | GO:0050853 | B cell receptor signaling pathway | 5/30 | 2.07E-06 | 0.000651353 | 0.000503308 | MS4A1/LCK/IGHD/CD79B/IGHM | 5 |
|  | BP | GO:0002429 | immune response-activating cell surface receptor signaling pathway | 7/30 | 8.22E-06 | 0.001290003 | 0.000996801 | MS4A1/LCK/CD3D/IGHD/CD79B/IGHM/CD3G | 7 |
|  | BP | GO:0002757 | immune response-activating signal transduction | 7/30 | 8.22E-06 | 0.001290003 | 0.000996801 | MS4A1/LCK/CD3D/IGHD/CD79B/IGHM/CD3G | 7 |
|  | BP | GO:0030098 | lymphocyte differentiation | 6/30 | 1.81E-05 | 0.002274554 | 0.001757575 | MS4A1/LCK/CD3D/TCF7/CD79B/CD3G | 6 |
|  | BP | GO:1903131 | mononuclear cell differentiation | 6/30 | 3.93E-05 | 0.004116218 | 0.003180651 | MS4A1/LCK/CD3D/TCF7/CD79B/CD3G | 6 |
|  | BP | GO:0045059 | positive thymic T cell selection | 2/30 | 0.000219913 | 0.019729293 | 0.015245062 | CD3D/CD3G | 2 |
|  | BP | GO:0045061 | thymic T cell selection | 2/30 | 0.00055384 | 0.041351428 | 0.031952746 | CD3D/CD3G | 2 |
|  | BP | GO:0030217 | T cell differentiation | 4/30 | 0.000592616 | 0.041351428 | 0.031952746 | LCK/CD3D/TCF7/CD3G | 4 |
|  | BP | GO:0042110 | T cell activation | 5/30 | 0.00083182 | 0.052238275 | 0.040365144 | LCK/CD3D/TCF7/ICOS/CD3G | 5 |
|  | CC | GO:0009897 | external side of plasma membrane | 6/30 | 1.89E-05 | 0.001489833 | 0.001032263 | MS4A1/CD3D/IGHD/CD79B/IGHM/CD3G | 6 |
|  | CC | GO:0019814 | immunoglobulin complex | 3/30 | 0.001721701 | 0.059566823 | 0.041272149 | IGHD/CD79B/IGHM | 3 |
|  | CC | GO:0044391 | ribosomal subunit | 3/30 | 0.002376093 | 0.059566823 | 0.041272149 | RPL36/MRPS33/RPL34 | 3 |
|  | CC | GO:0022625 | cytosolic large ribosomal subunit | 2/30 | 0.003016042 | 0.059566823 | 0.041272149 | RPL36/RPL34 | 2 |
|  | CC | GO:0005840 | ribosome | 3/30 | 0.004960516 | 0.071527707 | 0.049559504 | RPL36/MRPS33/RPL34 | 3 |
|  | CC | GO:0042571 | immunoglobulin complex, circulating | 2/30 | 0.005432484 | 0.071527707 | 0.049559504 | IGHD/IGHM | 2 |
|  | MF | GO:0042608 | T cell receptor binding | 2/30 | 0.000105495 | 0.010338507 | 0.007440171 | LCK/CD3G | 2 |
|  | up-regulated genes in cluster A | | | | | | | | |
| KEGG | hsa05146 |  | Amoebiasis | 4/37 | 0.001131508 | 0.116862407 | 0.101898878 | ARG1/IL1R2/TLR4/CXCL1 | 4 |
|  | hsa04915 |  | Estrogen signaling pathway | 4/37 | 0.003426351 | 0.116862407 | 0.101898878 | MMP9/KRT23/FOS/SP1 | 4 |
|  | hsa05418 |  | Fluid shear stress and atherosclerosis | 4/37 | 0.003516452 | 0.116862407 | 0.101898878 | MMP9/IL1R2/DUSP1/FOS | 4 |
|  | hsa05140 |  | Leishmaniasis | 3/37 | 0.005067295 | 0.116862407 | 0.101898878 | FCGR3B/FOS/TLR4 | 3 |
|  | hsa05323 |  | Rheumatoid arthritis | 3/37 | 0.008551723 | 0.116862407 | 0.101898878 | FOS/TLR4/CXCL1 | 3 |
|  | hsa04657 |  | IL-17 signaling pathway | 3/37 | 0.008806175 | 0.116862407 | 0.101898878 | MMP9/FOS/CXCL1 | 3 |
|  | hsa05150 |  | Staphylococcus aureus infection | 3/37 | 0.009328382 | 0.116862407 | 0.101898878 | FCGR3B/KRT23/FPR2 | 3 |
|  | hsa04621 |  | NOD-like receptor signaling pathway | 4/37 | 0.009433844 | 0.116862407 | 0.101898878 | TLR4/RNASEL/NAMPT/CXCL1 | 4 |
|  | hsa01522 |  | Endocrine resistance | 3/37 | 0.009868438 | 0.116862407 | 0.101898878 | MMP9/FOS/SP1 | 3 |
|  | hsa04060 |  | Cytokine-cytokine receptor interaction | 5/37 | 0.010316419 | 0.116862407 | 0.101898878 | IL1R2/TNFRSF10C/OSM/CXCL1/INHBB | 5 |
|  | hsa04613 |  | Neutrophil extracellular trap formation | 4/37 | 0.010530769 | 0.116862407 | 0.101898878 | FCGR3B/TLR4/AQP9/FPR2 | 4 |
|  | hsa05202 |  | Transcriptional misregulation in cancer | 4/37 | 0.010914095 | 0.116862407 | 0.101898878 | MMP9/IL1R2/BCL6/SP1 | 4 |
|  | hsa00500 |  | Starch and sucrose metabolism | 2/37 | 0.01156882 | 0.116862407 | 0.101898878 | MGAM/PYGL | 2 |
|  | hsa04928 |  | Parathyroid hormone synthesis, secretion and action | 3/37 | 0.012209505 | 0.116862407 | 0.101898878 | FOS/MMP25/SP1 | 3 |
|  | hsa04668 |  | TNF signaling pathway | 3/37 | 0.014158059 | 0.118596396 | 0.10341084 | MMP9/FOS/CXCL1 | 3 |
|  | hsa00260 |  | Glycine, serine and threonine metabolism | 2/37 | 0.014160764 | 0.118596396 | 0.10341084 | CBS/AOC3 | 2 |
|  | hsa05417 |  | Lipid and atherosclerosis | 4/37 | 0.01598622 | 0.126009031 | 0.109874332 | MMP9/FOS/TLR4/CXCL1 | 4 |
|  | hsa05134 |  | Legionellosis | 2/37 | 0.027607263 | 0.205520737 | 0.179205042 | TLR4/CXCL1 | 2 |
|  | hsa05161 |  | Hepatitis B | 3/37 | 0.037008869 | 0.261009915 | 0.227589164 | MMP9/FOS/TLR4 | 3 |
|  | hsa01230 |  | Biosynthesis of amino acids | 2/37 | 0.045617327 | 0.280343203 | 0.24444694 | ARG1/CBS | 2 |
|  | hsa04971 |  | Gastric acid secretion | 2/37 | 0.046718198 | 0.280343203 | 0.24444694 | KCNJ15/KCNJ2 | 2 |
|  | hsa05133 |  | Pertussis | 2/37 | 0.046718198 | 0.280343203 | 0.24444694 | FOS/TLR4 | 2 |
|  | hsa05152 |  | Tuberculosis | 3/37 | 0.048118609 | 0.280343203 | 0.24444694 | CLEC4E/FCGR3B/TLR4 | 3 |
|  | up-regulated genes in cluster B | | | | | | | | |
|  | hsa04660 |  | T cell receptor signaling pathway | 4/16 | 4.12E-05 | 0.001782493 | 0.001527851 | LCK/CD3D/ICOS/CD3G | 4 |
|  | hsa05340 |  | Primary immunodeficiency | 3/16 | 5.09E-05 | 0.001782493 | 0.001527851 | LCK/CD3D/ICOS | 3 |
|  | hsa05235 |  | PD-L1 expression and PD-1 checkpoint pathway in cancer | 3/16 | 0.000644747 | 0.012317585 | 0.01055793 | LCK/CD3D/CD3G | 3 |
|  | hsa04658 |  | Th1 and Th2 cell differentiation | 3/16 | 0.000710394 | 0.012317585 | 0.01055793 | LCK/CD3D/CD3G | 3 |
|  | hsa04640 |  | Hematopoietic cell lineage | 3/16 | 0.000879827 | 0.012317585 | 0.01055793 | MS4A1/CD3D/CD3G | 3 |
|  | hsa04659 |  | Th17 cell differentiation | 3/16 | 0.001132838 | 0.013216443 | 0.01132838 | LCK/CD3D/CD3G | 3 |
|  | hsa05166 |  | Human T-cell leukemia virus 1 infection | 3/16 | 0.00869992 | 0.086999197 | 0.07457074 | LCK/CD3D/CD3G | 3 |
|  | hsa05142 |  | Chagas disease | 2/16 | 0.016750409 | 0.135156012 | 0.11584801 | CD3D/CD3G | 2 |
|  | hsa04064 |  | NF-kappa B signaling pathway | 2/16 | 0.017377201 | 0.135156012 | 0.11584801 | LCK/BIRC3 | 2 |
|  | hsa05162 |  | Measles | 2/16 | 0.029896293 | 0.20927405 | 0.179377757 | CD3D/CD3G | 2 |
|  | hsa04390 |  | Hippo signaling pathway | 2/16 | 0.037395664 | 0.220686069 | 0.189159488 | TCF7/BIRC3 | 2 |
|  | hsa03010 |  | Ribosome | 2/16 | 0.037831898 | 0.220686069 | 0.189159488 | RPL36/RPL34 | 2 |
|  | hsa03430 |  | Mismatch repair | 1/16 | 0.044453108 | 0.239362888 | 0.20516819 | LIG1 | 1 |
| GO:Gene Ontology; CC:Cellular Component; BP:Biological Process; MF:Molecular Function; KEGG:Kyoto Encyclopedia of Genes and Genomes | | | | | | | | | |
