# Supplementary material for: Identification of molecular subtypes of coronary artery disease based on ferroptosis- and necroptosis-related genes
Source: Front Genet. 2022 Sep 20;13:870222. doi: 10.3389/fgene.2022.870222 (PMC9531137; doi:10.3389/fgene.2022.870222)
Supplement: Supplementary file 8 [file Table6.docx]

**Supplement Table6.** The consensus clustering approach to identify two subgroups (geneclusterA and genecluster B) based on the expression of the 4 Subgroup‐specific genes.

|  |  |  |  |
| --- | --- | --- | --- |
|  | Samples | geneCluster |  |
|  | GSM308600 | A |  |
|  | GSM308601 | B |  |
|  | GSM308603 | A |  |
|  | GSM308604 | B |  |
|  | GSM308605 | A |  |
|  | GSM308606 | A |  |
|  | GSM308607 | A |  |
|  | GSM308608 | A |  |
|  | GSM308609 | B |  |
|  | GSM308610 | B |  |
|  | GSM308611 | A |  |
|  | GSM308613 | B |  |
|  | GSM308614 | B |  |
|  | GSM308615 | B |  |
|  | GSM308616 | B |  |
|  | GSM308617 | B |  |
|  | GSM308618 | B |  |
|  | GSM308620 | B |  |
|  | GSM308622 | B |  |
|  | GSM308623 | B |  |
|  | GSM308625 | B |  |
|  | GSM308626 | A |  |
|  | GSM308627 | B |  |
|  | GSM308629 | B |  |
|  | GSM308630 | B |  |
|  | GSM308631 | B |  |
|  | GSM308632 | B |  |
|  | GSM308634 | B |  |
|  | GSM308637 | B |  |
|  | GSM308639 | A |  |
|  | GSM308640 | B |  |
|  | GSM308641 | B |  |
|  | GSM308642 | B |  |
|  | GSM308644 | A |  |
|  | GSM308645 | B |  |
|  | GSM308646 | A |  |
|  | GSM308647 | B |  |
|  | GSM308650 | A |  |
|  | GSM308652 | B |  |
|  | GSM308653 | B |  |
|  | GSM308655 | A |  |
|  | GSM308657 | B |  |
|  | GSM308658 | B |  |
|  | GSM308659 | A |  |
|  | GSM308664 | B |  |
|  | GSM308666 | B |  |
|  | GSM308667 | B |  |
|  | GSM308668 | A |  |
|  | GSM308669 | B |  |
|  | GSM308670 | B |  |
|  | GSM308671 | B |  |
|  | GSM308672 | B |  |
|  | GSM308673 | B |  |
|  | GSM308674 | B |  |
|  | GSM308675 | B |  |
|  | GSM308676 | B |  |
|  | GSM308678 | A |  |
|  | GSM308679 | B |  |
|  | GSM308681 | B |  |
|  | GSM308685 | B |  |
|  | GSM308686 | B |  |
|  | GSM308688 | B |  |
|  | GSM308689 | A |  |
|  | GSM308690 | B |  |
|  | GSM308691 | A |  |
|  | GSM308692 | A |  |
|  | GSM308693 | B |  |
|  | GSM308694 | A |  |
|  | GSM308695 | B |  |
|  | GSM308696 | B |  |
|  | GSM308697 | B |  |
|  | GSM308699 | A |  |
|  | GSM308700 | A |  |
|  | GSM308703 | A |  |
|  | GSM308704 | B |  |
|  | GSM308705 | A |  |
|  | GSM308707 | A |  |
|  | GSM308708 | A |  |
|  | GSM308710 | B |  |
|  | GSM308711 | B |  |
|  | GSM308715 | A |  |
|  | GSM308717 | B |  |
|  | GSM308718 | B |  |
|  | GSM308719 | B |  |
|  | GSM308723 | A |  |
|  | GSM308724 | A |  |
|  | GSM308725 | A |  |
|  | GSM308727 | B |  |
|  | GSM308734 | B |  |
|  | GSM308736 | B |  |
|  | GSM308737 | B |  |
|  | GSM308741 | B |  |
|  | GSM308742 | A |  |
|  | GSM308743 | A |  |
|  | GSM308745 | B |  |
|  | GSM308747 | A |  |
|  | GSM308752 | B |  |
|  | GSM308754 | B |  |
|  | GSM308755 | B |  |
|  | GSM308756 | B |  |
|  | GSM308759 | B |  |
|  | GSM308773 | B |  |
|  | GSM308775 | A |  |
|  | GSM308776 | B |  |
|  | GSM308778 | A |  |
|  | GSM308780 | B |  |
|  | GSM308781 | A |  |
|  | GSM308782 | B |  |
|  | GSM308783 | B |  |
|  | GSM308784 | B |  |
|  | GSM518690 | B |  |
|  | GSM518691 | B |  |
|  | GSM518692 | B |  |
|  | GSM518693 | B |  |
|  | GSM518694 | B |  |
|  | GSM518695 | B |  |
|  | GSM518696 | B |  |
|  | GSM518697 | B |  |
|  | GSM518698 | B |  |
|  | GSM518699 | A |  |
|  | GSM518700 | B |  |
|  | GSM518701 | B |  |
|  | GSM518702 | A |  |
|  | GSM518703 | B |  |
|  | GSM518704 | B |  |
|  | GSM518705 | A |  |
|  | GSM518706 | B |  |
|  | GSM518707 | B |  |
|  | GSM518708 | B |  |
|  | GSM518709 | B |  |
|  | GSM518710 | A |  |
|  | GSM518711 | B |  |
|  | GSM518712 | B |  |
|  | GSM518713 | B |  |
|  | GSM518714 | B |  |
|  | GSM518715 | B |  |
|  | GSM518716 | B |  |
|  | GSM518717 | A |  |
|  | GSM518718 | B |  |
|  | GSM518719 | B |  |
|  | GSM518720 | A |  |
|  | GSM518721 | A |  |
|  | GSM518722 | B |  |
|  | GSM518723 | B |  |
|  | GSM518724 | B |  |
|  | GSM518725 | B |  |
|  | GSM518726 | A |  |
|  | GSM518727 | B |  |
|  | GSM518728 | B |  |
|  | GSM518729 | B |  |
|  | GSM518730 | B |  |
|  | GSM518731 | A |  |
|  | GSM518732 | B |  |
|  | GSM518733 | A |  |
|  | GSM518734 | A |  |
|  | GSM518735 | A |  |
|  | GSM518736 | B |  |
|  | GSM518737 | A |  |
|  | GSM518738 | B |  |
|  | GSM518739 | A |  |
|  | GSM518740 | A |  |
|  | GSM518741 | B |  |
|  | GSM518742 | A |  |
|  | GSM518743 | B |  |
|  | GSM518744 | A |  |
|  | GSM518745 | A |  |
|  | GSM518746 | A |  |
|  | GSM518747 | A |  |
|  | GSM518748 | B |  |
|  | GSM518749 | B |  |
|  | GSM518750 | B |  |
|  | GSM518751 | A |  |
|  | GSM518752 | B |  |
|  | GSM518753 | B |  |
|  | GSM518754 | B |  |
|  | GSM518755 | A |  |
|  | GSM518756 | B |  |
|  | GSM518757 | B |  |
|  | GSM518758 | A |  |
|  | GSM518759 | A |  |
|  | GSM518760 | B |  |
|  | GSM518761 | B |  |
|  | GSM518762 | B |  |
|  | GSM518763 | B |  |
|  | GSM518764 | B |  |
|  | GSM518765 | B |  |
|  | GSM518766 | A |  |
|  | GSM518767 | A |  |
|  | GSM518768 | A |  |
|  | GSM518769 | B |  |
|  | GSM518770 | B |  |
|  | GSM518771 | B |  |
|  | GSM518772 | B |  |
|  | GSM518773 | B |  |
|  | GSM518774 | A |  |
|  | GSM518775 | A |  |
|  | GSM518776 | B |  |
|  | GSM518777 | B |  |
|  | GSM518778 | B |  |
|  | GSM518779 | B |  |
|  | GSM518780 | A |  |
|  | GSM518781 | B |  |
|  | GSM518782 | B |  |
|  | GSM518783 | A |  |
|  | GSM518784 | B |  |
|  | GSM518785 | B |  |
|  | GSM518786 | B |  |
|  | GSM518787 | B |  |
|  | GSM518788 | B |  |
|  | GSM518789 | B |  |
|  | GSM518790 | A |  |
|  | GSM518791 | B |  |
|  | GSM518792 | B |  |
|  | GSM518793 | A |  |
|  | GSM518794 | B |  |
|  | GSM518795 | B |  |
|  | GSM518796 | B |  |
|  | GSM518797 | B |  |
|  | GSM518798 | B |  |
|  | GSM518799 | B |  |
|  | GSM518800 | B |  |
|  | GSM518801 | B |  |
|  | GSM518802 | A |  |
|  | GSM518803 | B |  |
|  | GSM518804 | B |  |
|  | GSM518805 | A |  |
|  | GSM518806 | B |  |
|  | GSM518807 | B |  |
|  | GSM518808 | A |  |
|  | GSM518809 | B |  |
|  | GSM518810 | A |  |
|  | GSM518811 | B |  |
|  | GSM518812 | A |  |
|  | GSM518813 | B |  |
|  | GSM518814 | B |  |
|  | GSM518815 | A |  |
|  | GSM518816 | A |  |
|  | GSM518817 | A |  |
|  | GSM518818 | B |  |
|  | GSM518819 | B |  |
|  | GSM518820 | B |  |
|  | GSM518821 | B |  |
|  | GSM518822 | B |  |
|  | GSM518823 | A |  |
|  | GSM518824 | A |  |
|  | GSM518825 | A |  |
|  | GSM518826 | B |  |
|  | GSM518827 | A |  |
|  | GSM518828 | B |  |
|  | GSM518829 | A |  |
|  | GSM518830 | B |  |
|  | GSM518831 | B |  |
|  | GSM518832 | A |  |
|  | GSM518885 | A |  |
|  | GSM518887 | B |  |
|  | GSM518889 | A |  |
|  | GSM518891 | B |  |
|  | GSM518893 | A |  |
|  | GSM518895 | A |  |
|  | GSM518897 | B |  |
|  | GSM518899 | B |  |
|  | GSM518901 | B |  |
|  | GSM518903 | B |  |
|  | GSM518905 | A |  |
|  | GSM518907 | B |  |
|  | GSM518909 | B |  |
|  | GSM518911 | B |  |
|  | GSM518913 | A |  |
|  | GSM518915 | A |  |
|  | GSM518917 | B |  |
|  | GSM518919 | B |  |
|  | GSM518921 | A |  |
|  | GSM518923 | B |  |
|  | GSM518925 | A |  |
|  | GSM518927 | B |  |
|  | GSM518929 | B |  |
|  | GSM518931 | B |  |
|  | GSM518933 | A |  |
|  | GSM518935 | A |  |
|  | GSM518937 | A |  |
|  | GSM518939 | A |  |
|  | GSM518941 | B |  |
|  | GSM518943 | A |  |
|  | GSM518945 | B |  |
|  | GSM518947 | A |  |
|  | GSM518949 | A |  |
|  | GSM518951 | A |  |
|  | GSM518953 | A |  |
|  | GSM518955 | A |  |
|  | GSM518957 | A |  |
|  | GSM518959 | A |  |
|  | GSM518961 | A |  |
|  | GSM518963 | A |  |
|  | GSM518965 | B |  |
|  | GSM518967 | A |  |
|  | GSM518969 | B |  |
|  | GSM518971 | A |  |
|  | GSM518973 | B |  |
|  | GSM518975 | A |  |
|  | GSM518977 | B |  |
|  | GSM518979 | A |  |
|  | GSM518981 | A |  |
|  | GSM518983 | A |  |
|  | GSM518985 | B |  |
|  | GSM518987 | B |  |
|  | GSM518989 | B |  |
|  | GSM518991 | B |  |
|  | GSM518993 | A |  |
|  | GSM518995 | B |  |
|  | GSM518997 | B |  |
|  | GSM518999 | B |  |
|  | GSM519001 | A |  |
|  | GSM519003 | A |  |
|  | GSM519005 | A |  |
|  | GSM519007 | B |  |
|  | GSM519009 | A |  |
|  | GSM519011 | B |  |
|  | GSM519013 | B |  |
|  | GSM519015 | B |  |
|  | GSM519017 | A |  |
|  | GSM519019 | B |  |
|  | GSM519021 | B |  |
|  | GSM519023 | A |  |
|  | GSM519025 | A |  |
|  | GSM519027 | A |  |
|  | GSM519029 | B |  |
|  | GSM519031 | B |  |
|  | GSM519033 | A |  |
|  | GSM519035 | B |  |
|  | GSM519037 | B |  |
|  | GSM519039 | B |  |
|  | GSM519041 | B |  |
|  | GSM519043 | B |  |
|  | GSM519045 | B |  |
|  | GSM519047 | B |  |
|  | GSM519049 | A |  |
|  | GSM519051 | B |  |
|  | GSM519053 | B |  |
|  | GSM519055 | B |  |
|  | GSM519057 | A |  |
|  | GSM519059 | B |  |
|  | GSM519061 | B |  |
|  | GSM519063 | B |  |
|  | GSM519065 | B |  |
|  | GSM519067 | B |  |
|  | GSM519069 | B |  |
|  | GSM519071 | A |  |
|  | GSM519073 | B |  |
|  | GSM519075 | A |  |
|  | GSM519077 | B |  |
|  | GSM519079 | B |  |
|  | GSM519081 | B |  |
|  |  |  |  |
